# The indispensable contribution of s38 protein to ovarian-eggshell morphogenesis in *Drosophila melanogaster*

**DOI:** 10.1038/s41598-018-34532-2

**Published:** 2018-10-31

**Authors:** Athanassios D. Velentzas, Panagiotis D. Velentzas, Stamatia A. Katarachia, Athanasios K. Anagnostopoulos, Niki E. Sagioglou, Eleni V. Thanou, Maria M. Tsioka, Vassiliki E. Mpakou, Zoe Kollia, Vassilios E. Gavriil, Issidora S. Papassideri, George Th. Tsangaris, Alkiviadis-Constantinos Cefalas, Evangelia Sarantopoulou, Dimitrios J. Stravopodis

**Affiliations:** 1 Section of Cell Biology and Biophysics, Department of Biology, School of Science, National and Kapodistrian University of Athens (NKUA), Athens, Greece; 2 Systems Biology Center, Biomedical Research Foundation of the Academy of Athens (BRFAA), Athens, Greece; 3 Theoretical and Physical Chemistry Institute, National Hellenic Research Foundation (NHRF), Athens, Greece; 4 Present Address: Department of Cancer Biology, Medical School, University of Massachusetts, Worcester, Massachusetts (MA) USA

## Abstract

*Drosophila* chorion represents a remarkable model system for the *in vivo* study of complex extracellular-matrix architectures. For its organization and structure, s38 protein is considered as a component of major importance, since it is synthesized and secreted during early choriogenesis. However, there is no evidence that proves its essential, or redundant, role in chorion biogenesis. Hence, we show that targeted downregulation of s38 protein, specifically in the ovarian follicle-cell compartment, via employment of an RNAi-mediated strategy, causes generation of diverse dysmorphic phenotypes, regarding eggshell’s regionally and radially specialized structures. Downregulation of s38 protein severely impairs fly’s fertility and is unable to be compensated by the s36 homologous family member, thus unveiling s38 protein’s essential contribution to chorion’s assembly and function. Altogether, s38 acts as a key skeletal protein being critically implicated in the patterning establishment of a highly structured tripartite endochorion. Furthermore, it seems that s38 loss may sensitize choriogenesis to stochastic variation in its coordination and timing.

## Introduction

*Drosophila* oogenesis represents a vital developmental process often used as a biological platform for the *in vivo* analysis of gene regulation, cell differentiation, cell migration, cell death and tissue morphogenesis^1,2^. *Drosophila* eggs arise from individual structural and functional units called follicles, or otherwise egg chambers^3^. Each follicle proceeds through 14 anatomically and morphologically distinct stages of development^4^ (20 according to Margaritis LH, 1986^5^) and contains 16 germ-line cells (15 nurse cells and 1 oocyte) surrounded by a monolayer of approximately 650 somatic epithelial cells, called follicle cells^2,6^. During the developmental stages 11–14, in a process known as choriogenesis, peripherally organized groups of columnar follicle cells synthesize chorion proteins and secrete eggshell components onto the oocyte’s surface, where they assemble to form the multi-layered chorion^1,7^. Besides its protective role in the developing embryo, the mature eggshell displays highly organized regional complexity with specialized structures, such as the micropyle (permits the fertilization of the egg), the dorsal appendages (facilitate gas exchange for the embryo) and the operculum (a weakened region for the release of larva from its protective shell)^3,7–9^. The eggshell also exhibits highly structured radial complexity, which is best portrayed in the main body region of the follicle and consists of five architecturally distinct and successive layers that encompass the oocyte. These are: (a) the innermost vitelline membrane (~300 nm), which appears as a continuous granular layer without prominent substructures; (b) the lipid wax layer; (c) the inner chorionic layer or ICL (~40–50 nm), (d) the endochorion (~500–700 nm) and (e) the outermost, adjacent to the follicle-cell layer, thin and amorphous non-proteinaceous exochorion (~300–500 nm)^5,7,10,11^. Endochorion is a tripartite layer comprised of floor and roof structures separated by numerous pillars, which leave air-spaces in-between them, allowing eggs to facilitate gas exchange^7^. The ICL, endochorion and exochorion layers are jointly referred to as chorion^7^.

Molecular and developmental analysis have revealed the existence of at least 20 structural proteins (6 major ones) in the eggshell of *Drosophila melanogaster*. Genes encoding the major chorion proteins are clustered in two chromosomal loci of the fly genome^9,12,13^. The first cluster is located at the 7F1–7F1 cytogenetic region of X chromosome and contains, among others, the two major *s36* and *s38* chorion genes, which are expressed early during the eggshell formation course, mainly at stages 11–12. The other major chorion genes *s15*, *s16*, *s18* and *s19* are mapped at the cytological location 66D12-66D12 of the 3^rd^ chromosome, and are mainly expressed at the later developmental stages 13–14^7,9,14–19^. These two chorion-gene clusters are temporally regulated and selectively amplified, in order to enable the sequential synthesis of chorion-protein amounts required during the short 5 h-period of choriogenesis process^16,19^. This rapid synthesis is controlled by genomic regions (known as *Drosophila* amplicons in follicle cells) that undergo extensive re-replication, prior to transcription, to increase the DNA copy number. Amplification levels of the major chorion genes mapped on the X chromosome are up to approximately 18–20x (folds), whereas the respective ones of the 3^rd^ chromosome cluster are estimated at 60–80x^12,20,21^.

Hitherto, apart from the s36 protein, the *in vivo* role of the other -five- major chorion proteins in *Drosophila* has not been elucidated through gene-targeted strategies, such as RNAi-mediated gene-specific silencing^15^. Although several female-sterile mutants with substantial disruption of the endochorion and underproduction of all six major chorion proteins have been previously produced, they proved to only reduce the amplification rather than the transcription activity of the genes involved^12,22–24^. One characteristic mutation isolated following X-ray mutagenesis is the *ocelliless* (*oc*), which involves a small chromosome inversion (*In*(1)7F1,2-8A1,2) with a distal break-point located 1–3 Kb upstream from the *s36* and *s38* chorion genes^7,16,25,26^. The *oc* mutation causes a *cis*-acting reduction of s36 and s38 protein contents, and results in the formation of abnormal eggshell and failure of fly embryo to develop^12,18,26^. However, the obtained *oc* phenotype is pleiotropic. *oc* name derives from the observation that homozygote mutant flies totally lack the ocelli (little eyes, in Latin) organs. Moreover, *oc* mutant animals exhibit an abnormal pattern of bristles in the ocellar region of the head, while female flies usually lack parovaria, an accessory gland of the reproductive tract^25,27^. These observations likely suggest that the *oc* genomic lesion, besides the *s36* and *s38* genes, simultaneously alters the functional capacity of other essential for *Drosophila* physiology genes. Other mutations, such as the *cor-36* and *Cp36*^*dec2-1*^, which have been cytogenetically mapped at the respective regions 7E10-8A4 and 7E10-8A5 of the X chromosome, proved to cause structural abnormalities in the endochorionic layer and lead to a fragile chorion^7,28–31^. However, these chromosomal regions contain 40 and 41 genes respectively, with only 5 of them [*Cp7Fa*, *Cp7Fb*, *Cp7Fc*, *Cp36* (*s36*) and *Cp38* (*s38*)] being classified as *bona fide* chorion genes^32^.

Therefore, since there is no convincing evidence that the aforementioned phenotypes are associated with a chorion gene-specific deregulation, we have herein aimed to examine, after suitable employment of the GAL4/UAS binary genetic system and the RNAi-based technology^33^, the effects of follicle cell-specific *s38*-gene silencing in chorion biogenesis. Interestingly, the generation of diverse dysmorphic phenotypes, regarding follicle’s regionally and radially specialized structures, in the s38-targeted flies, strongly suggests a critical role for s38 protein in choriogenic patterning, while s38 loss seems to sensitize choriogenesis to stochastic variation regarding its coordination and timing.

## Results

### RNAi-mediated suppression of *s38*-gene expression, specifically in the follicle-cell compartment, leads to generation of s38-deficient ovaries in *D. melanogaster*

We have recently reported that -ovarian- follicle cell-specific downregulation of s36 protein results in deformed and severely impaired endochorion, which mechanistically causes female infertility^15^. To the same direction, we have herein attempted to illuminate the architectural role of s38 protein [being produced by two alternatively spliced RNA transcripts (RA and RB)] in the patterning establishment of chorion’s regional specialization and radial complexity. Hence, by applying the GAL4/UAS and RNAi genetic technologies^34^, the biological effects of *s38* gene-specific expression silencing, exclusively in the follicle-cell compartment of *D. melanogaster* developing ovary, were thoroughly examined. Even though there are two s38-RNAi (GD and KK) stock lines available (with no reported “off-targets”), the majority of our experiments were performed with the s38-RNAi KK stock line, due to the genetically improved nature of the KK stock collection^35^. Nevertheless, the s38-RNAi GD stock line was also used for the independent confirmation of the obtained follicle dysmorphias.

**Figure 1:**
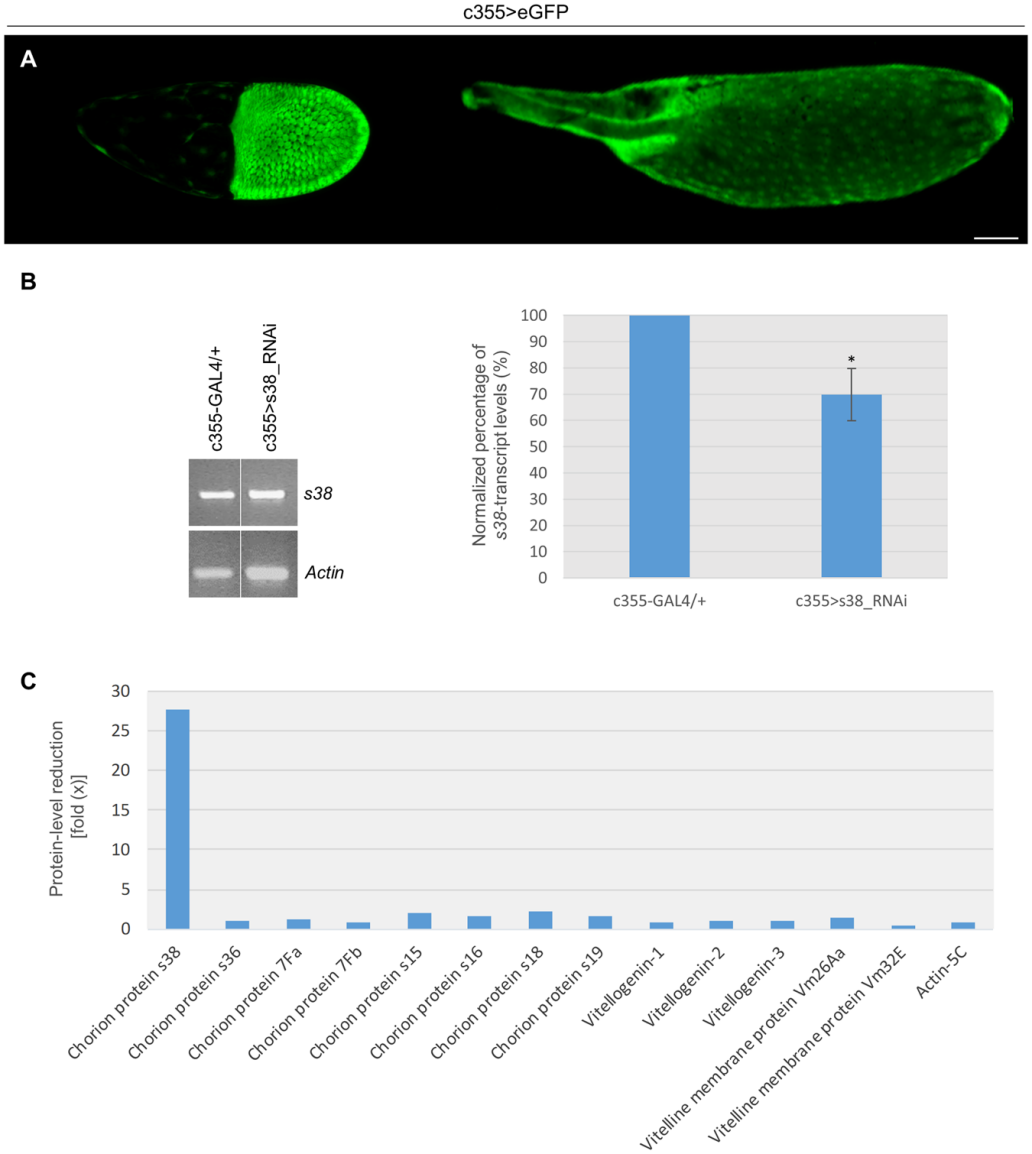
RNAi-mediated targeting of *s38* gene specifically in the follicle-cell population of *D. melanogaster* generates ovaries with severely reduced levels of s38 protein. (**A**) Confocal laser scanning microscopy (CLSM) images of representative stage-10 (left panel) and stage-14 (right panel) follicles derived from c355 > eGFP transgenic control flies, specifically expressing the eGFP reporter protein in their follicle cells. Number of follicles examined: 89. (**B**) The *s38*-transcript contents in s38-targeted (c355 > s38_RNAi) ovaries are presented with an -average- reduction of 30%, as compared to control (c355-GAL4/+) ones, via employment of an RT-PCR protocol and utilization of *Actin* as gene of reference. (**C**) Graphical presentation of Mascot score-based comparative measurements of relative protein abundance (presented in fold: “x”), in between s38-downregulated (Table S2) and control^38^ ovaries. Scale Bar: 50 μm. *p < 0.05.

The follicle cell-specific patterning of GAL4-driver’s promoter activity, throughout late oogenesis, is demonstrated in Fig. 1A, whereat two characteristic follicles, one of stage 10 (left panel) and one of stage 14 (right panel), with strong GFP expression in their follicle-cell populations, are presented via confocal-laser-scanning-microscopy imaging. Next, ovaries derived from control (c355-GAL4/+) and s38-downregulated (c355 > s38_RNAi) transgenic flies were examined for the detection of both *s38*-gene transcript (RA and RB) contents, through employment of RT-PCR protocols. Interestingly, despite the -developmentally regulated- strong (18–20x) gene-amplification program of the X-chromosome cluster, which significantly increases the DNA copy number^20,21^, the *s38*-transcript(s) abundance proved to be quantitatively reduced at an average percentage of 30% in the s38-targeted, as compared to control, ovaries (Fig. 1B {PCR products derived from both *s38-RA* and *s38-RB* RNA transcripts} and data not shown {PCR fragments reflecting *s38-RB*-specific transcript levels}), thus indicating the *s38* gene-silencing efficiency of our GAL4/UAS-s38_RNAi transgenic platform in fly ovary.

Then, since no specific validated antibody against the *Drosophila* s38 protein is yet available, a nLC-MS/MS (n: nano) proteomics technology was suitably applied for mapping the proteomic landscape of s38-targeted ovaries (c355 > s38_RNAi). Such an approach offers the advantage of providing, within the same analysis settings, data regarding both upregulation and downregulation changes at the protein level, which can occur in the absence of changes at the RNA level^36^. Ovaries were selected over laid eggs for protein extraction, since the vitelline membrane and the chorion are additionally stabilized by tyrosine-based (covalent-bond) cross-linking upon ovulation, a process that renders eggshell proteins completely insoluble in laid eggs and derived extracts^7,37^. Obtained data from nLC-MS/MS analysis and following processing of the 17,274 generated unique peptides, through the *D. melanogaster* reference proteome, identified 2,123 distinct proteins (Table S2). Taking advantage of a previously described nLC-MS/MS-based proteomic dataset of *Drosophila* control (c355-GAL4/+) ovaries^38^, we further conducted comparative measurements of protein abundance (Fig. 1C), using Mascot-score value as the evaluation criterion of differential quantification^36^. This analysis revealed that while all major proteins critically implicated in vitellogenesis (Vitellogenin 1–3, Vm26Aa and Vm32E) or in diverse structural (e.g. Actin-5C) networks either remained unaffected or were subjected to small changes in their total contents, an approximately 27x reduction of the s38-protein levels was observed in ovaries derived from the c355 > s38_RNAi transgenic flies (Fig. 1C). Remarkably, the other known -major- chorion proteins, Cp7Fa, Cp7Fb, s15, s16, s18, s19 and s36, were presented with insignificant quantity changes in their respective protein contents, in between control and s38-targeted ovaries (Fig. 1C), thus indicating that downregulation of s38 protein does not affect the expression profiles of the other chorion protein-family members. Intriguingly, the moderate reduction of *s38*-gene transcript(s) abundance (Fig. 1B), as compared to the strong one of s38-protein level (Fig. 1C), specifically in s38-targeted ovaries, underscores the activation of an RNAi-dependent mechanism that functions via more translational repression than transcript degradation of specific RNA targets, as previously reported for other systems^39^. Conclusively, our RT-PCR and nLC-MS/MS proteomics analysis demonstrate the efficient disruption of *s38*-gene expression in fly ovary, and the critical contribution of follicle-cell compartment to s38-protein synthesis and secretion.

### Follicle cell-specific downregulation of s38 protein results in production of laid eggs characterized by diverse types of dysmorphic dorsal appendages

**Figure 2:**
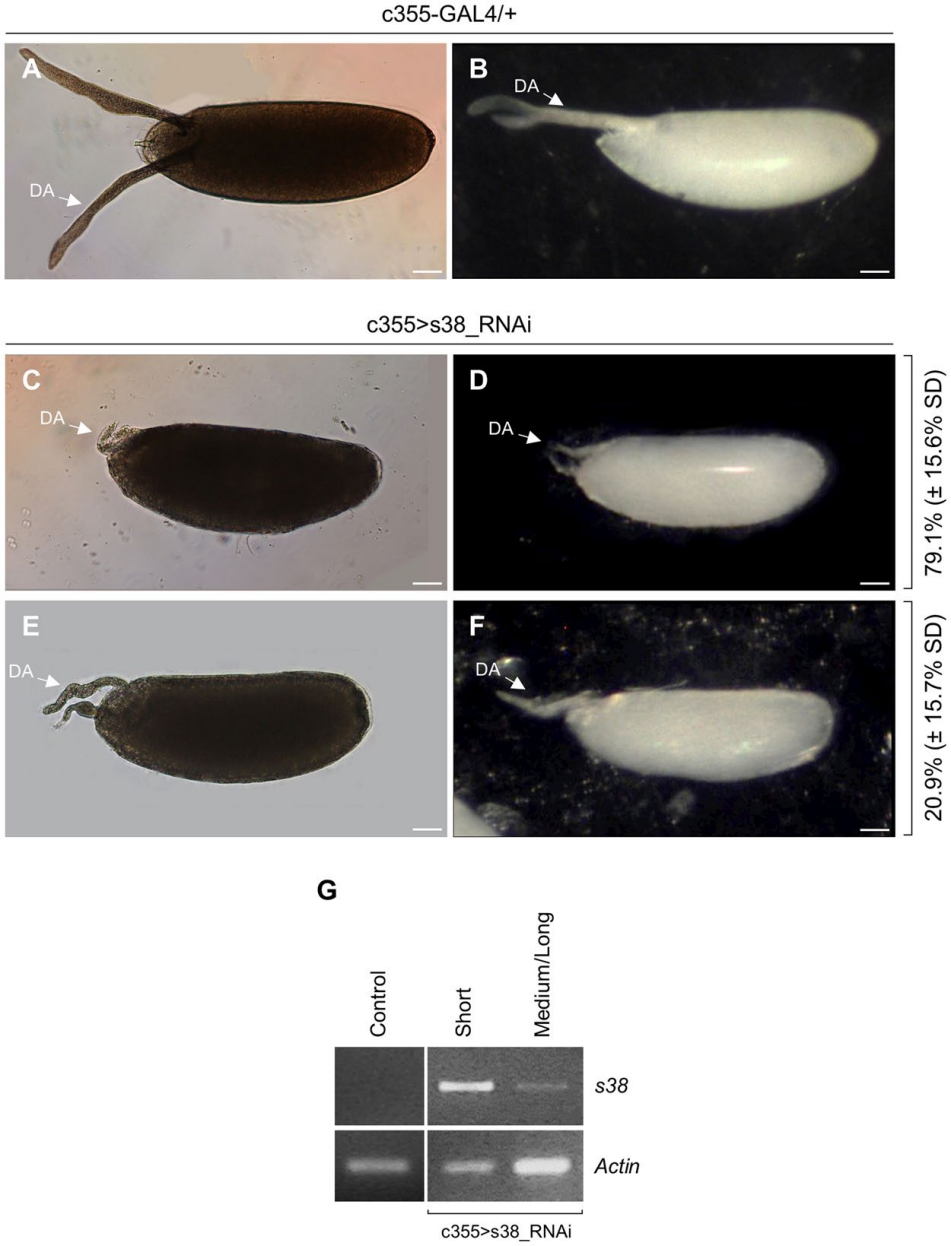
The s38-protein lack essentially affects the length and morphology of dorsal appendages in *Drosophila* eggs. (**A**,**B**) The morphology of control (c355-GAL4/+) laid eggs is characterized by two prominent dorsal appendages, as illustrated through a (**A**) light microscope and (**B**) stereomicroscope. (**C**,**D**) Characteristic s38-targeted (c355 > s38_RNAi) laid eggs with very short and thin dorsal appendages, and a glassy external surface (**D**). (**E**,**F**) Representative s38-downregulated laid eggs with comparatively longer but still dysmorphic dorsal appendages. Number of eggs examined: control = 291, s38-downregulated = 713. (**G**) *s38* transcript-level quantification of control (c355-GAL4/+) and s38-targeted (c355 > s38_RNAi) follicles carrying either short or medium/long in length dorsal appendages, via employment of an RT-PCR protocol. *Actin* served as gene of reference. DA: Dorsal Appendage(s). Scale Bars: 50 μm.

**Figure 3:**
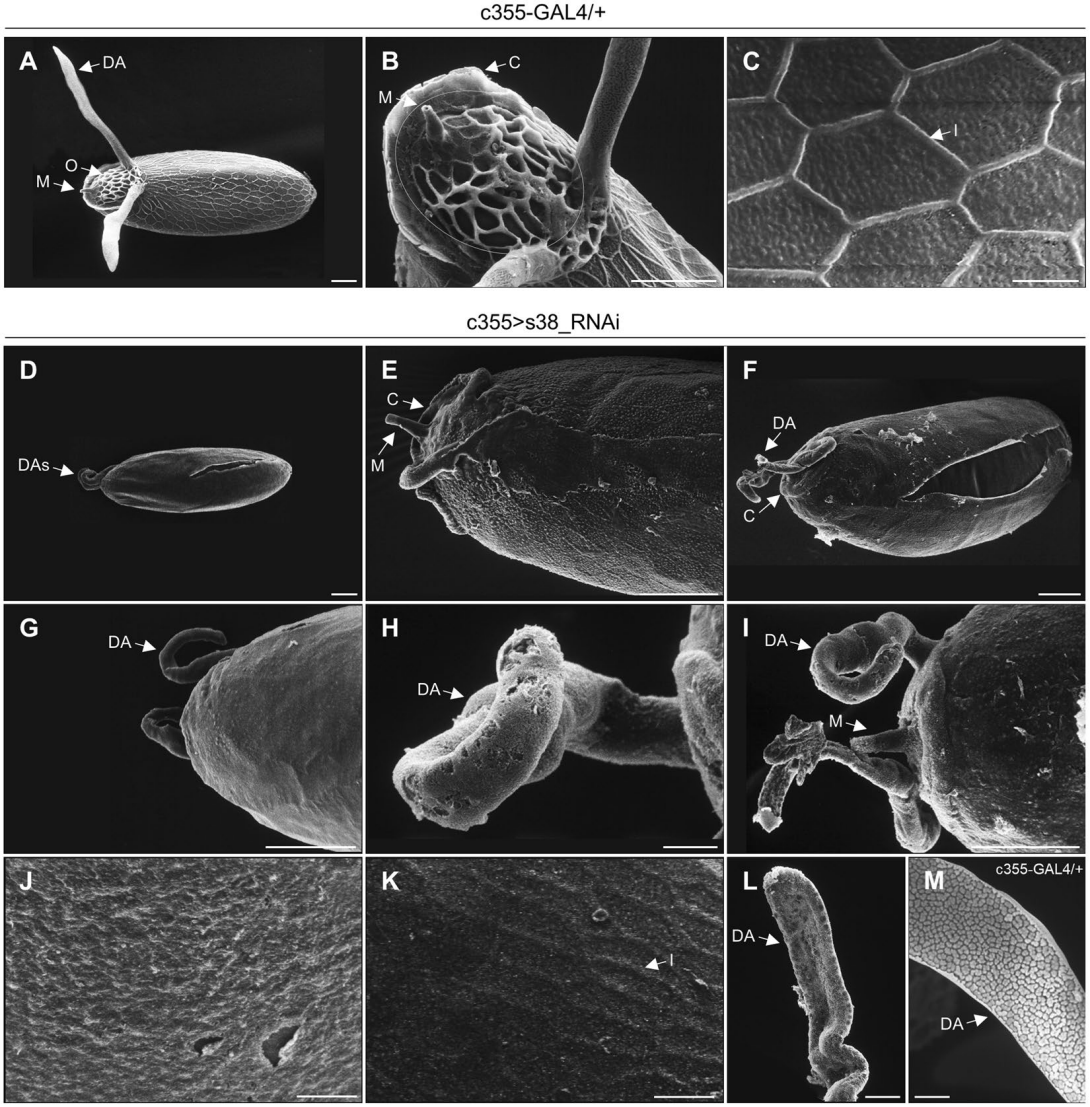
Targeted downregulation of s38 protein in the ovarian follicle cells disrupts the patterning establishment of chorion’s regional specialization. (**A**–**C**) Scanning-electron-microscopy images of representative control (c355-GAL4/+) laid eggs. (**A**) Far-off view of the general egg morphology, featuring the two typically shaped and sized dorsal appendages. (**B**) The operculum region (orange-circled area) with the characteristic pattern of follicle-cell imprints on its surface and a morphologically undamaged micropyle. (**C**) High magnification scanning-electron-microscopy image of main-body egg’s surface, illustrating the characteristic hexagonal follicular imprints. (**D**–**L**) Scanning electron micrographs of representative s38-downregulated (c355 > s38_RNAi) laid eggs revealing diverse eggshell malformations. (**D**–**G**) Low magnification images of eggs being characterized by dysmorphic dorsal appendages with pleiotropic pathology, and variable sizes and shapes. (**H**,**I**) Close-up views of representative highly dysmorphic, thin and coiled dorsal appendages with abnormal tips. (**J**) The main-body surface morphology of an egg carrying short in length dorsal appendages is structured by a smooth and fragile eggshell without follicular imprints. (**K**,**L**) Eggs with longer but still abnormal in size dorsal appendages are presented with some follicular imprints in their main-body surface, albeit bearing much thinner ridges (**K**), while they encompass some typical features of physiological dorsal appendages (**L**). (**M**) A typical dorsal appendage with a paddle-shaped tip from a control laid egg, exhibiting discrete plaques on its dorsal surface. Number of eggs examined: control = 139, s38-downregulated = 271. DA: Dorsal Appendage(s), O: Operculum, M: Micropyle, C: Collar and I: Imprint. Scale Bars: 50 μm in (**A**,**B**,**D**–**G** and **I**) 10 μm in (**C**,**H** and **J**–**M**).

Control (c355-GAL4/+) laid eggs, as they are observed through light microscopy, are presented by carrying two long dorsal appendages, with paddle-shaped tips, protruding by approximately 50% of egg’s length beyond the anterior end. Dorsal appendages function as gills for the developing embryo, when it is submerged in water or rotting fruit (Fig. 2A,B). In contrast, the s38-downregulated (c355 > s38_RNAi) laid eggs are characterized by coiled, highly dysmorphic dorsal appendages of diverse lengths, which are considerably reduced in both thickness and length, and also lack the typical paddle-shaped tips (Fig. 2C–F). The defects of dorsal appendages in s38-targeted ovaries significantly vary among eggs. Surprisingly, their length ranges from notably short (Fig. 2C,D), observed in approximately 80% of the laid eggs examined, to comparatively longer (Fig. 2E,F), but always shorter than the one of control follicles (Fig. 2A,B). Furthermore, eggs bearing short in length dorsal appendages seem to be more severely affected by the s38-protein downregulation, since they prove to contain a thinner and more fragile eggshell that lacks the characteristic pentagonal and hexagonal imprints on its external surface (Fig. 3D), giving eggs a glassy appearance (Fig. 2D). On the other hand, s38-targeted eggs with dysmorphic but longer in length dorsal appendages tend to correlate with a comparatively more normal shape and still manage to contain some follicular imprints (Figs 2F, 3E). To independently validate the obtained diverse phenotypes, we also examined the s38-RNAi GD stock line. Similar aberrations were observed in these s38-targeted follicles (Fig. S1A–E), confirming that egg dysmorphia derives specifically from the s38-protein lack.

To examine the contribution of *s38*-transcript levels (as they are determined by the RNAi-mediated gene-silencing process) to the variable dysmorphic phenotypes obtained, we, next, employed an RT-PCR strategy, using as templates RNA preparations derived from control and s38-targeted, late-stage 14, follicles. Interestingly, c355 > s38_RNAi follicles with short in length dorsal appendages are presented with significantly increased contents of *s38*-gene transcripts (RA and RB), as compared to the ones of (s38-downregulated) follicles with long(er) in length dorsal appendages (Fig. 2G). Control (c355-GAL4/+) late-stage 14 follicles proved to lack detectable *s38*-transcription activity (Fig. 2G), in accordance with previous reports. s38 is considered as an early chorion protein that mainly functions in endochorion synthesis and its cognate gene remains transcriptionally active till stage 13^9,14,40^. Therefore, the elevated *s38*-transcript contents in s38-downregulated (late-stage 14) follicles with short in length dorsal appendages may represent a counterbalance mechanism of follicle cells to synthesize and secrete, in a temporally aberrant manner, the adequate s38-protein amounts required for normal chorion biogenesis. The RNAi machinery-induced translational repression of *s38*-transcript species (Fig. 1B,C) likely compels targeted follicle cells to enhance and prolong the *s38*-gene transcription activities till the end of choriogenesis (late-stage 14). This may serve as a defense effort of the follicle cells to produce small quantities of the s38 protein to even partly rescue chorion’s architectural structure.

Given the important role of a rigid eggshell in egg’s elliptical shape during late oogenesis^41^, we, next, examined the impact of s38 lack on the maintenance of egg’s elongated shape. Interestingly, both length (Fig. S2A) and length to width ratio (Fig. S2B) were notably reduced in the s38-targeted laid eggs, as compared to the control ones, indicating the essential contribution of s38 protein to eggshell’s rigidity. Specifically, the median length of s38-targeted (c355 > s38_RNAi) laid eggs (excluding dorsal-appendages measurement) is approximately 475 μm, as compared to the one of control (c355-GAL4/+) eggs, whose median length is approximately measured at 525 μm (Fig. S2A). Similarly, the length to width ratio was also decreased from an approximate value of 2.8 (control eggs) to a 2.5 one (s38-downregulated eggs) (Fig. S2B).

### The essential role of s38 protein in the patterning establishment of chorion’s regional specialization and radial complexity

Scanning-electron-microscopy (SEM) analysis of representative control (c355-GAL4/+) laid eggs unveils the highly specialized structures formed at both the anterior and posterior follicle poles (Fig. 3A,B). In accordance with previous reports, the dorsal part of anterior pole carries the operculum area (Fig. 3B), which contains the micropyle, the two long dorsal appendages and the collar, a raised specialized transition zone at its periphery, which breaks open to permit hatching of the larva^5^. The operculum area is characterized by a unique and distinct pattern of follicle-cell imprints (Fig. 3B), which reflect the expression of a highly developed endochorionic-roof network in this region^11^. Micropyle (Fig. 3B), with an external eccentric canal and an internal eccentric paracrystalline region^42,43^, is a narrow channel that functions as the sperm entry point during fertilization. Dorsal appendages (Fig. 3A,B,M) are two narrow cylindrical stalks with flattened paddles at their tips and exhibit two distinct surfaces: a dorsal one covered with porous plaques similar to endochorionic floor and a ventral one analogous to endochorionic-roof network^5,11^. In the main body of laid-egg’s surface, the most prominent structures are pentagonal and hexagonal imprints, which are visible on the eggshell only after follicle cells degenerate and slough off, during oviposition (Fig. 3C).

Similar examination of laid eggs derived from s38-downregulated (c355 > s38_RNAi) ovaries revealed eggshells with diverse morphological abnormalities (Fig. 3D–L). The most prominent defect is related to the size of dorsal appendages. In agreement with the light microscopy-extracted phenotypes (Fig. 2C–F), the s38-deficient laid eggs can be roughly categorized into two major groups: (a) eggs with very small dorsal appendages (Fig. 3D,G) and (b) eggs with comparatively larger dorsal appendages (Fig. 3E,F,H,I,L), with some of the second group showing typical features of control dorsal appendages (Fig. 3L, as compared to 3M). Regardless of the size, all laid eggs from s38-downregulated flies, as compared to control ones, are presented to contain short, thin and coiled dorsal appendages, with no clear orientation, and to also lack the characteristic paddle-shaped tips (Fig. 3D–I). The operculum area shows the same extent of severe malformations in all examined eggs being laid by s38-targeted flies, with the most prominent ones being the poorly developed collar and the complete loss of follicle cell-imprint pattern on its surface, which strongly suggests an underdeveloped endochorion in this region (Fig. 3D–G,I). In terms of the fine surface morphology of main-body eggshell, the s38-downregulated laid eggs, in comparison to control ones, prove to be smoother, carrying abnormally thin and fragile eggshell (Fig. 3D–G), while in the vast majority of them the follicular imprints are entirely missing (Fig. 3D,G,J). Interestingly, s38-targeted laid eggs with -comparatively- longer/larger dorsal appendages seem to contain some regionally distinct imprints on their surface, albeit they bear much thinner ridges (Fig. 3E,F,K). In conclusion, lack of s38 protein likely renders regional-specialization patterning more sensitive to stochastic variation in the coordination and timing of morphogenetic events.

**Figure 4:**
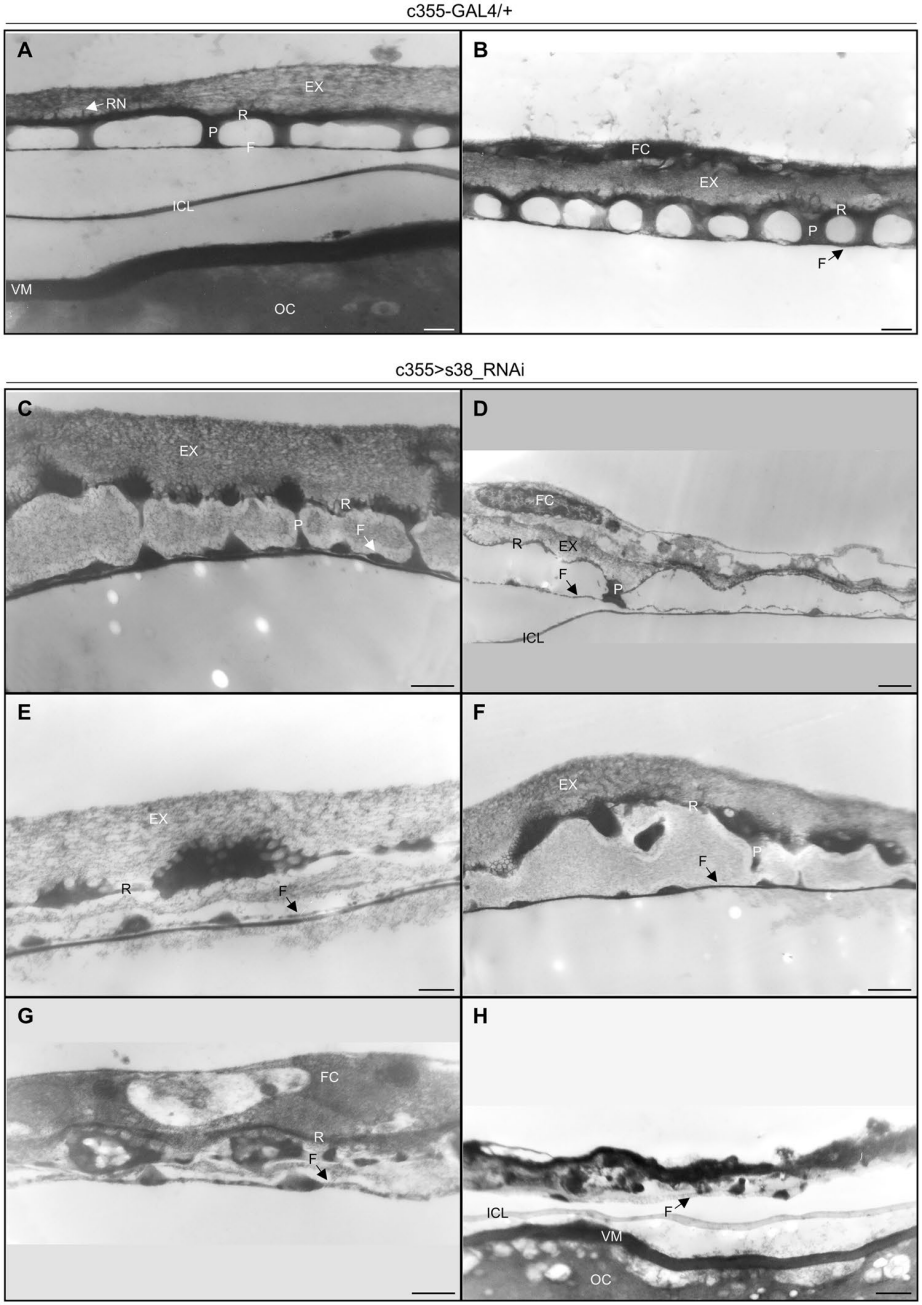
Lack of s38 protein causes disintegration of chorion’s radial complexity. (**A**,**B**) Transmission-electron-microscopy images of main-body eggshell from control (c355-GAL4/+) stage-14 follicles, illustrating all typical structures of the tripartite endochorion. (**C**–**H**) Transmission electron micrographs of eggshell’s ultrastructural organization in s38-downregulated (c355 > s38_RNAi) follicles revealing phenotypic variation of endochorion’s dysmorphia. (**C**–**F**) Unpredictable and stochastic endochorionic assemblies, bearing some architectural elements of a typical endochorion, albeit with thinner fragmented floor and roof structures, less prominent pillars, and larger in size cavities full of loose material (as compared to control ones). (**G**) Completely disintegrated endochorion structure, containing randomly arranged clumps of endochorionic material. (**H**) Follicle with entirely collapsed endochorion, but without detectable morphological deformities in the ICL or vitelline membrane. Number of follicles examined: control = 9, s38-downregulated = 17. OC: Oocyte, VM: Vitelline Membrane, ICL: Inner Chorionic Layer, F: Floor, P: Pillar(s), R: Roof, EX: Exochorion, RN: Roof Network and FC: Follicle Cell(s). Scale Bars: 0.5 μm.

Since eggshell-surface defects vary notably among eggs of the same genotype (c355 > s38_RNAi), we, next, investigated, via a transmission-electron-microscopy (TEM) protocol, the presumable disruption of eggshell’s radial complexity. Ultrathin cross sections in the main body of control (c355-GAL4/+) stage-14 follicles revealed the typical eggshell’s radial complexity (Fig. 4A,B). Specifically, the tripartite endochorion [consisted of the thin floor (F), the vertical pillars (P) and the thick roof (R)] is located between the innermost chorionic layer (ICL) and the outermost non-proteinaceous exochorion (EX) (Fig. 4A,B). The prominent cavities between floor and roof layers, created by the vertical pillars, facilitate gas exchange. The roof of endochorion is anchored into the exochorion through characteristic protrusions, forming the roof network (RN). External reticulum projections from the roof of endochorion, termed ridges, mark the borders between adjacent follicle cells. Finally, the homogeneous, electron dense, vitelline membrane (VM) is located underneath the ICL and very close to the oocyte (Fig. 4A).

A similar analysis of the main-body region from nearly mature s38-targeted (c355 > s38_RNAi) follicles demonstrated a chorion’s radial complexity that proved considerably dissimilar in structure [as compared to the normal one (Fig. 4A,B)], varying from almost typical (Fig. 4C) to completely pathological (Fig. 4G,H) ones, among the examined follicles. Although chorion’s ultrastructural morphology illustrated in Fig. 4C contains a tripartite endochorion, its roof obtains, in different areas, either a comparatively thinner, or an aggregated and cluttered organization. Pillars are moderately underdeveloped with rather reduced contents of endochorionic material (Fig. 4C), as opposed to the ones of control follicles (Fig. 4A,B). Moreover, their cavities lose the typical canonical shape, become larger and accumulate loose material (Fig. 4C). Nevertheless, the vast majority of s38-downregulated follicles are presented with severe malformations in endochorion’s structural integrity (Fig. 4D–H). Specifically, a number of diverse dysmorphic phenotypes are obtained in response to s38-protein lack, with all of them being characterized by a prominent disintegration of chorion’s structural architecture. Depending on the -distinct- type of dysmorphia, (a) the very thin floor can be either fragmented or aberrantly organized by uneven chorion deposition, (b) the pillars can be poorly developed or replaced by large irregular clumps of endochorionic material, and (c) the roof can significantly become thinner and/or obtain a discontinuous organization (Fig. 4D–F). The indispensable contribution of s38 protein to patterning establishment of eggshell’s radial complexity is even more convincingly demonstrated in Fig. 4G,H, which describe the complete collapse of endochorion and the total failure of exochorion to be properly assembled, in the absence of s38 protein from follicle-cell compartment. Floor and pillars are replaced by small clumps of endochorionic material, and only a very thin roof can be identified (Fig. 4G,H). In contrast to endochorion, the ICL seems unaffected, thus suggesting a non-essential, or redundant, role for s38 protein in its biogenesis. Moreover, even in the s38-targeted follicles with severe chorion malformations, no obvious morphological and functional aberrations in the vitelline membrane could be ever observed (Figs 4H, S3). Taken together, we herein suggest that s38 protein serves as an essential structural component of chorion’s radial specialization. Also, its absence seems to sensitize the radial-complexity patterning to stochastic variation in the timing and coordination of endochorion’s assembly.

### Atomic force microscopy-mediated assessment of surface deformity and fragility in s38-targeted eggshells

**Figure 5:**
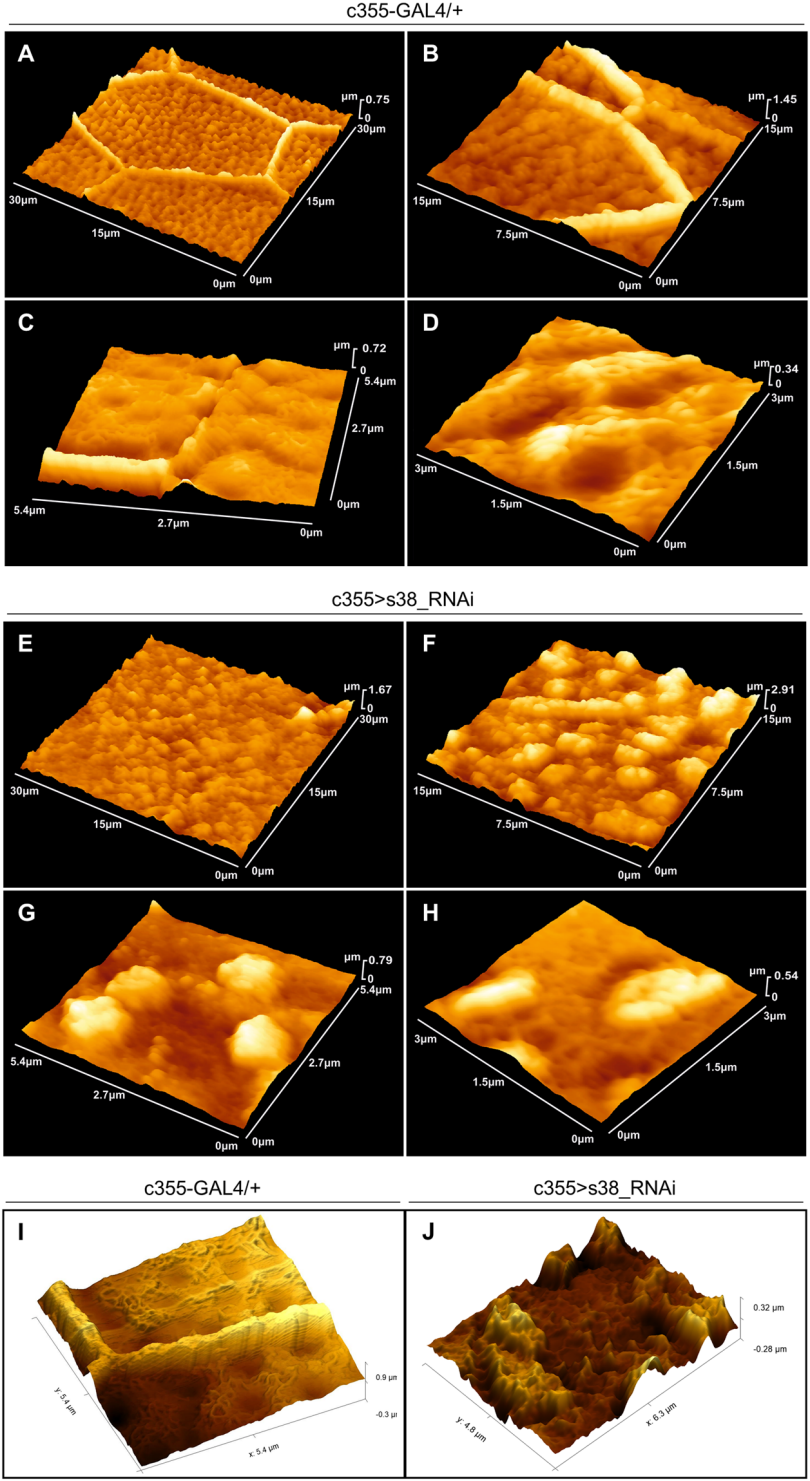
AFM-mediated topography of surface deformities in s38-targeted laid eggs. (**A**–**D**) Atomic-force-microscopy (AFM) imaging of control (c355-GAL4/+) (laid-egg) eggshell-surface topography, in increasing magnifications, illustrating the characteristic follicular-imprint hexagonal pattern (**A**–**C**). (**D**) Inside view of a hexagonal area, where, in a canonical distribution, dimple-like structures are self-assembled in a hill-and-valley arrangement. (**E**–**H**) AFM analysis of s38-downregulated (c355 > s38_RNAi) eggs, in increasing magnifications, unveils a severely disorganized eggshell surface that lacks the typical follicular imprints. In addition, the hill-and-valley/dimple patterns of control eggs are replaced by unevenly distributed granular structures. (**I**,**J**) Enhanced images of control (**I**) and s38-targeted (**J**) eggs, using the Gwyddion visualization software, demonstrating the fibrillary organized surface of control egg, within its hexagonal area (**I**), in contrast to the randomly organized granular surface of a representative s38-targeted (**J**) one. Number of eggs examined: control = 8, s38-downregulated = 14.

Atomic-force-microscopy (AFM) analysis is able to provide high-resolution quantification data of eggshell’s topography and, as it is carried out in environmental conditions, offers a more real-life view of egg-surface characteristics. Surface topography from 30 × 30 μm^2^ area of a representative control (c355-GAL4/+) egg (Fig. 5A) demonstrates the typical surface pattern of *D. melanogaster* eggshell, mainly defined by arrays of regular hexagonal (and pentagonal) formations, the so-called follicular imprints, being created from chorionic material secreted by the above lying follicle cells. Follicular imprints, as illustrated at higher-magnification images, vary in length, and consist of solenoid-like structures of approximately 1.00–1.50 μm in width and 400–800 nm in height (Fig. 5B,C,I). Within each hexagon (and pentagon) area, in a rather canonical distribution, dimple-like structures of an approximately 600–800 nm mean diameter are self-assembled, in a hill-and-valley arrangement (Fig. 5D,I). In contrast, a representative AFM surface-topography image of a typical s38-targeted (c355 > s38_RNAi) laid egg, with 30 × 30 μm^2^ scan area, unveils a disorganized surface that lacks the characteristic hexagonal (and pentagonal) pattern (Fig. 5E). Furthermore, the typical hill-and-valley/dimple pattern of control eggs is missing, and only parts of solenoid-like structures are randomly arranged, together with unevenly distributed granular formations (Fig. 5F–H,J).

**Figure 6:**
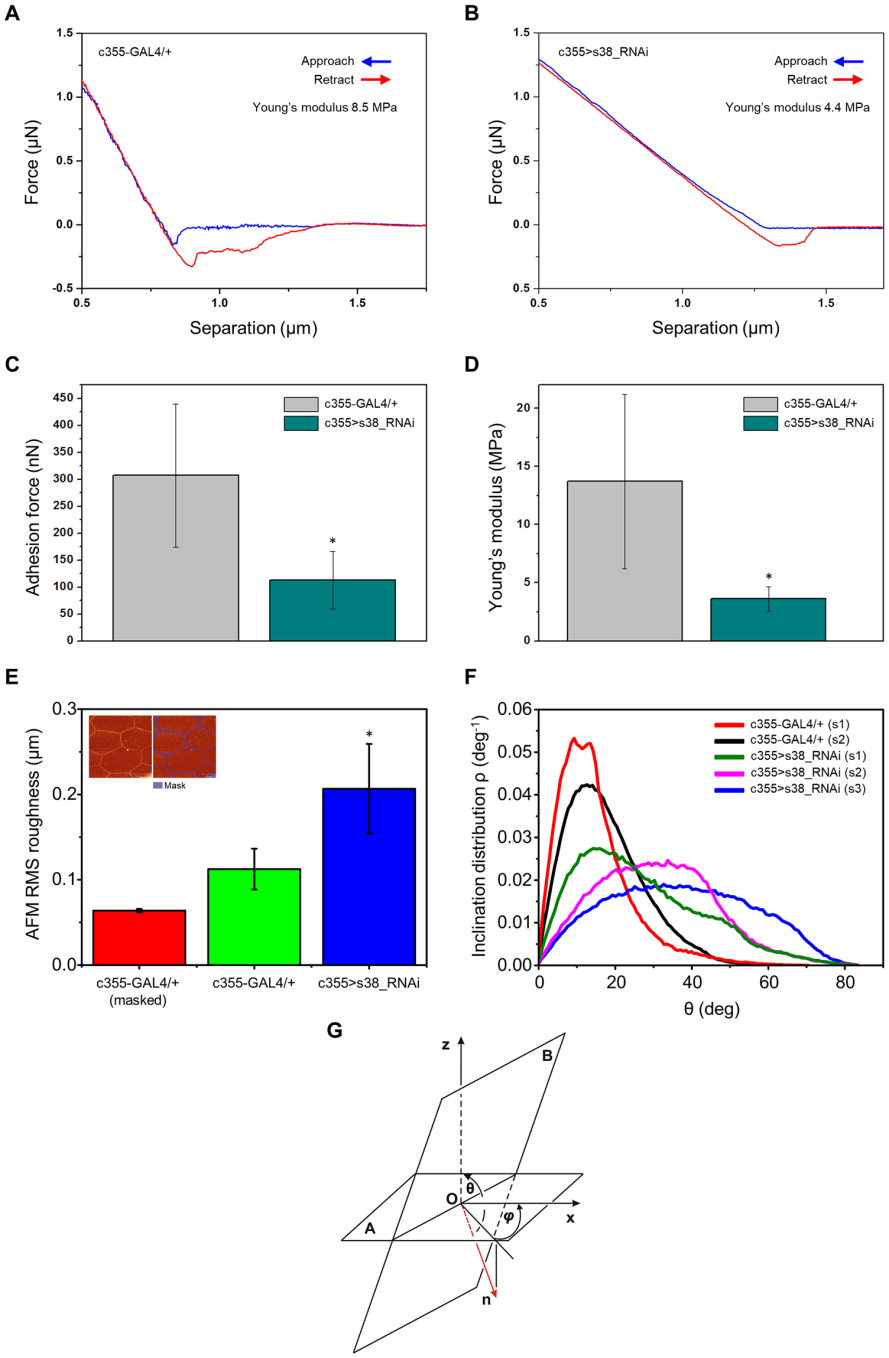
AFM-based quantification of eggshell-surface mechanical parameters in the presence or absence of s38 protein. (**A**,**B**) Typical approach (blue) and retraction (red) force-distance curves of a representative (**A**) control (c355-GAL4/+) and (**B**) s38-targeted (c355 > s38_RNAi) laid egg. (**C**,**D**) Bar-charts presenting the mean values of (**C**) adhesion force and (**D**) Young’s modulus of control and s38-downregulated laid eggs. (**E**) AFM-dependent average RMS roughness of masked control (red bar), unmasked control (green bar) and s38-targeted (blue bar) laid eggs. (**F**) Inclination distribution ρ for angles θ from 0 to 90° in AFM images. (**G**) Schematic layout displaying the angle θ between the z axis and the unit-vector $\overrightarrow{n}$ of one pixel, at the point O. Number of eggs examined: control = 8, s38-downregulated = 14. s: sample. *p < 0.05.

Nanoindentation represents an approach-retract mode of AFM technology that can reliably evaluate cell-surface compliance and elasticity. Typical force-distance (F-D) curves in the main-body region of control (c355-GAL4/+) and s38-downregulated (c355 > s38_RNAi) eggs are illustrated in Fig. 6A,B, respectively. The obtained elastic modulus values for a typical control and s38-downregulated egg were 8.5 and 4.4 MPa, respectively (Fig. 6A,B). Furthermore, the mean adhesion-force values for control and s38-targeted eggs were 318 and 138 nN, respectively (Fig. 6C). These findings indicate a role of s38 protein in the maintenance of surface robustness. The elastic modulus and adhesion force, measured on three different s38-targeted eggs, at six distinct positions, ranged between 2.6 and 5.1 MPa, and 58 and 203 nN, respectively, while the mean elastic-modulus and adhesion-force values with sample standard deviations (mean ± SSD) were 3.6 ± 1.1 MPa and 113.3 ± 53.3 nN, respectively (Fig. 6C,D). For comparison reasons, the elastic-modulus and adhesion-force values of control eggs are also presented (Fig. 6C,D). These data reveal a nearly 3x reduction of both mean elasticity and adhesion-force values between control and s38-downregulated eggs. This indicates that the ability of s38-downregulated eggs to resist deformation from mechanical stress is significantly limited, as compared to the one of control eggs, and also suggests that lack of s38 protein causes degeneration of egg’s surface structure, likely altering its biochemical composition.

The area RMS (Root Mean Square) roughness surface-parameter values were also measured for both control and s38-downregulated eggs. RMS is defined as ${R}_{ms}=\sqrt{\frac{1}{N}{\sum }_{i=1}^{N}|{Z}_{i}-\bar{Z}|}$, where N is the number of pixels of AFM image, Z_i_ the height in the i^th^ pixel and $\overline{Z\,}$ the mean height of the AFM image. The s38-targeted eggs are presented with considerably higher mean RMS roughness (blue bar) and high RMS standard-deviation values (indicating the significant roughness variability among s38-targeted eggs), as compared to the mean RMS values of unmasked (green bar) and masked (red bar) control eggs (Fig. 6E). Masked control values (red bar) represent RMS values derived from control eggs (green bar) after the subtraction of follicular imprints (see purple lines in inset of Fig. 6E).

To further demonstrate the architectural meddling caused by s38-protein lack in egg-surface structures (e.g. hexagonal/pentagonal -ordered- arrays for control and hill-like -cluttered- formations for s38-targeted eggs), the inclination-angle distributions were quantified for both control and s38-downregulated eggs (Fig. 6F), using the Gwyddion software^44^. The polar angle θ between the horizontal plane (A) and the “central derivative plane” in every pixel at a point O (B) (Fig. 6G) is related to the surface-profile gradient $\overrightarrow{u}=(\frac{dz}{dx},\frac{dz}{dy})$ via the equation $\,\theta ={\tan }^{-1}|\overrightarrow{u}|$, where $\,\overrightarrow{n}=\frac{(\frac{dz}{dx},\frac{dz}{dy})}{|(\frac{dz}{dx},\frac{dz}{dy})|}$. The polar angle θ is always positive and rises with a slope $|(\frac{dz}{dx},\frac{dz}{dy})|$, while the integral $\int \rho \,(\theta )d\theta$ from $\theta \in [0,\frac{\pi }{2}]$ is normalized to one and $\rho =\frac{Number\,of\,counts}{\int (Number\,of\,counts)d\theta }$. Figure 6F shows that lack of follicular imprints in the s38-downregulated -laid- eggs leads to notably wider inclination-angle distributions, as compared to the ones of control eggs that are being characterized by the unique hexagonal (and pentagonal) pattern on their surface. It seems that control egg’s surface is typified by a structural surface regularity, whereas the s38-targeted one is defined by random patterns of atypical and de-oriented structures. Altogether, perturbation in the inclination angle-distribution profiling may be critically associated with the disruption of physical interactions between chorion’s external surface and adjacent follicle-cell layer, likely leading to impaired post-fertilization development.

### Specific targeting of *s38* gene in the ovarian follicle-cell compartment causes severe reduction of fly’s fecundity

**Figure 7:**
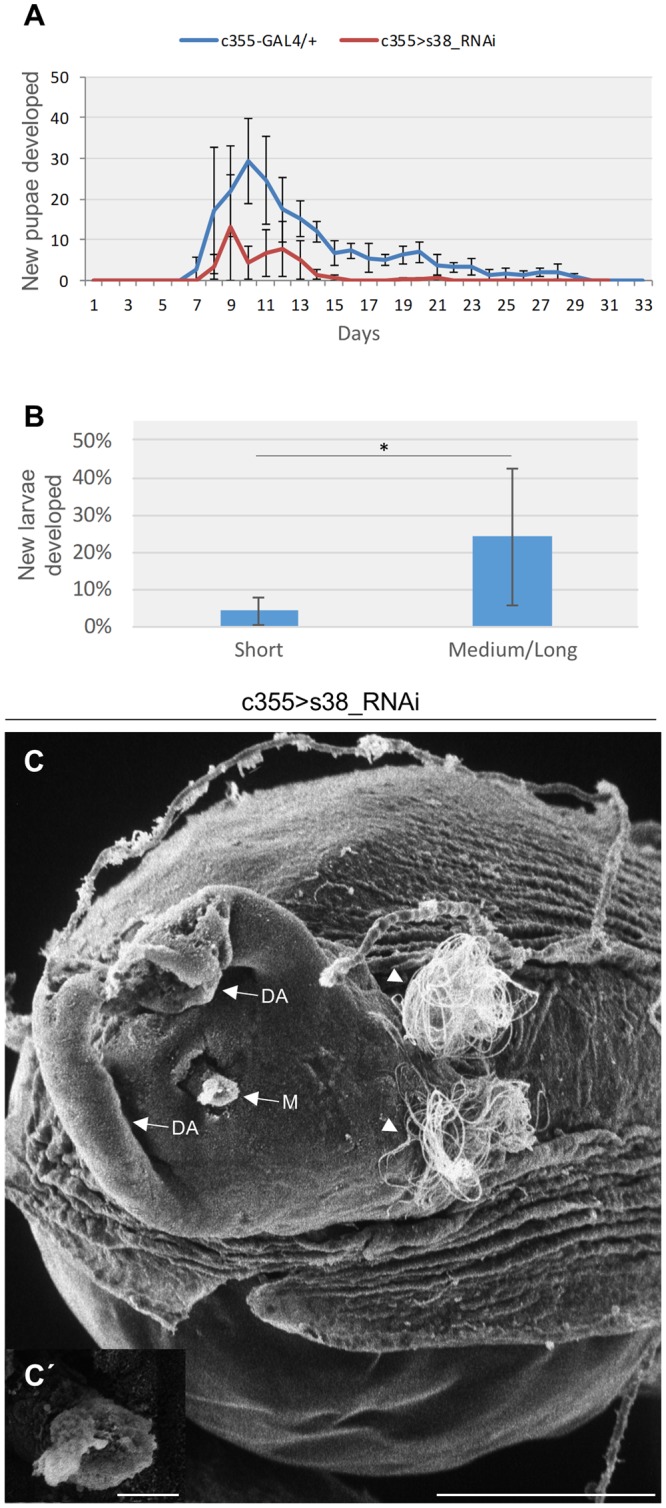
Follicle-cell cluster-specific downregulation of s38 protein severely harms fly’s fertility. (**A**) Eclosion-efficiency graphs of both control (c355-GAL4/+) (n = 60) and s38-downregulated (c355 > s38_RNAi) (n = 60) flies, for a thirty-days period, illustrating the severe reduction of fecundity capacity in s38-targeted flies. (**B**) Bar-chart presenting the eclosion-efficiency percentage of different egg categories with respect to their dorsal-appendages length. Laid eggs with (comparatively) longer in length (but still dysmorphic) dorsal appendages (n = 76) are strongly associated with increased percentage of hatching competence, as compared to eggs carrying short in length (and dysmorphic) dorsal appendages (n = 147). (**C**) Scanning electron micrograph of the operculum area of a representative s38-targeted laid egg, bearing a micropyle with blocked sperm opening and spermatozoa (white arrowheads) on its surface. (C′) Higher-magnification image of the clogged micropyle. M: Micropyle and DAs: Dorsal Appendages. Scale Bars: 50 μm in C; 5 μm in C′. *p < 0.05.

Our data clearly demonstrate that lack of s38 protein in *D. melanogaster* ovary results in flies that lay eggs with severely deformed regionally and radially specialized structures, mainly due to an impaired endochorion deposition (Figs 3, 4). Hence, to further examine the impact of s38 lack-induced chorionic disintegration on fecundity competence, we, next, proceeded to perform hatchability assays. In contrast to control (c355-GAL4/+) flies, the s38-targeted (c355 > s38_RNAi) ones (KK and GD stock lines) were presented with a significantly reduced eclosion efficiency (Figs 7A, S1F). Due to the variability in the structural defects among s38-downregulated -laid- eggs, the eclosion capacity of eggs carrying different length of dorsal appendages was individually measured (Fig. 7B). Interestingly, approximately 95% of the oviposited eggs with short length of dorsal appendages could not hatch, in contrast to eggs with malformed but comparatively longer in length dorsal appendages, which exhibited an average 25% hatching efficiency (Fig. 7B).

The herein observed s38 lack-dependent reduction of hatching competence could derive from failure in egg fertilization. In accordance, scanning electron micrographs of the micropyle region from representative s38-targeted -laid- eggs revealed a clogged micropyle (Fig. 7C,C′). Moreover, at the anterior part of the egg examined, spermatozoa (arrowheads) that failed to pass through the defective micropyle can also be observed (Fig. 7C). Micropyle is a specialized narrow channel at the anterior tip of the egg with a clear sperm opening (Fig. 3B) that facilitates sperm entry during fertilization, an indispensable process for the egg to complete meiosis and produce the zygote. Therefore, it seems that impaired hatching, in the absence of s38 protein, may likely derive from failure in egg fertilization, most probably due to endochorionic collapse within the micropyle that ultimately results in its structural block and functional obstruction.

## Discussion

*Drosophila* eggshell possesses a vital role in embryo’s protection and physiology, and its patterning represents a remarkable *in vivo* model system for processes engaged in the assembly of complex extracellular-matrix architectures^1,7,9^. Hitherto, all mutational studies on *Drosophila* chorion-protein functions, excluding our recent publication in which the s36 protein was specifically depleted from the follicle-cell population of developing egg chamber^15^, described the disruption of either chorionic-amplification-control elements or genes mapped onto a 16-band chromosomal region of the X chromosome, which contains approximately 40 genes, with 5 of them (*Cp7Fa*, *Cp7Fb*, *Cp7Fc*, *Cp36/s36* and *Cp38/s38*) being considered as chorion genes^7,26–29,31^.

Therefore, since all the previously published mutational studies, except the s36-related one^15^, failed to specifically target the chorion genes of interest, but simultaneously affected several other genes, we have herein employed the GAL4/UAS binary genetic system, combined with the RNAi technology^33,45^, to specifically downregulate the s38 protein in the follicle-cell compartment of *D. melanogaster* ovary. Our data reveal that RNAi-mediated targeting of *s38*-gene expression in fly ovary results in dysmorphic follicles being characterized by variable structural eggshell defects. Laid eggs from s38-deficient flies, based on the size of their dorsal appendages, can be roughly categorized into two major groups: (a) eggs with very small dorsal appendages and (b) eggs with comparatively larger dorsal appendages. The external surface morphology of both types of laid eggs (compared to control ones) is generally presented with: rounder and shorter oocytes, thin and fragile eggshells, short, thin and collapsed dorsal appendages, dysmorphic operculum areas and underdeveloped or no follicular imprints. Interestingly, laid eggs carrying short dorsal appendages seem to be more severely affected by the s38-protein downregulation compared to eggs with dysmorphic but longer in length dorsal appendages, which tend to correlate with some typical features of control eggs. For example, laid eggs with short dorsal appendages are characterized by thinner eggshell, as indicated by the lack of follicular imprints on their external surface, when compared to eggs with longer in length dorsal appendages that still manage to contain some follicular imprints.

The ultrastructural analysis of s38-downregulated stage-14 follicles revealed a considerably dissimilar structural radial complexity among them, varying from partly typical to completely pathological one. Nevertheless, all the examined follicles feature disrupted endochorion that clearly diverges from the typical tripartite structure, suggesting the indispensable role of s38 protein in architectural formation of a scaffold-like framework, which is likely required for other chorion proteins to be properly bound onto it, thus successfully molding and completing fly’s chorion body. Moreover, in follicles carrying the most severe phenotypes, the ridges that physiologically project from the roof network and mark the borders between adjacent follicle cells, thus creating the visible imprints on the external surface, are severely underdeveloped, justifying the lack of follicular imprints in the s38-downregulated laid eggs. Dorsal appendages are composed internally of branched endochorion, while the operculum area contains prominent cell imprints, as a result of the highly developed endochorionic-roof network in this region^5,10,11^. The endochorionic origin of these structures explains the highly dysmorphic phenotypes of dorsal appendages and operculum area detected in the s38-targeted laid eggs, thus indicating the essential role of s38 protein in their biogenesis and function.

Endochorion is architecturally structured during the developmental stages 11–13 of oogenesis^5,7^, in the course of which transcription, RNA contents and protein synthesis derived from the *s36* and *s38* genes are at maximal levels^9,14,40,46^. This temporal synchronization between *s38*-gene expression and endochorion synthesis likely suggests the pivotal contribution of s38 protein to endochorion biogenesis. Interestingly, laid eggs carrying short dorsal appendages appear with severely disrupted endochorion-based structures, suggesting critically low s38-protein quantities in these follicles. Most importantly, it seems that the s36 homologous family-member protein cannot act as a functionally equivalent chorion component to compensate for the s38 lack, while different quantities of s38 protein must decisively control the development of diverse pathological phenotypes herein described. Hence, the reduction of s38-protein levels below a minimal threshold is sufficient to fully disrupt the architectural structure and organization of ovarian endochorion. In contrast, the maintenance of s38-protein contents above a critical threshold causes formation of comparatively milder defects. In control follicles, during the late choriogenic stage 14 of oogenesis, the *s38* gene is transcriptionally quiescent^9,14,40,46^. Surprisingly, stage-14 follicles carrying dysmorphic but comparatively longer dorsal appendages, derived from s38-targeted ovaries, are presented with an elevated activity of *s38*-gene transcription, which becomes even stronger in follicles with short in length dorsal appendages. Therefore, we herein suggest that s38 protein, after succeeding its optimal levels, most likely outside the follicular compartment, could act as a critical regulatory switch to turn off the *s38*-gene transcription itself, constituting a decisive component of a negative feedback loop of signaling, specifically operating in stage-14 ovarian follicles. Reduction in s38-protein synthesis and secretion probably attenuates the robustness of this negative feedback loop and de-represses *s38*-gene transcription activity. The elevated *s38*-RNA-transcript levels detected in stage-14 s38-downregulated follicles may represent a counterbalance mechanism of follicle cells, in order them to synthesize and secrete the adequate (or, even optimal) amounts of s38 protein that are typically required for chorion’s normal biogenesis. Interestingly, RNAi-mediated targeting of s38 protein specifically in regionally specialized follicle-cell populations, such as the ones controlling micropyle and dorsal-appendages morphogenesis (using suitable GAL4 genetic drivers), proved insufficient to cause detectable dysmorphias (data not shown), thus indicating enrichment of s38-protein quantities in the targeted areas by s38-protein transfer from adjacent unaffected cells.

The biochemical process that governs the three-dimensional assembly and patterning organization of a number of proteins to a chorionic architectural structure likely entails inherent randomness, since it typically involves molecules compelled to thermally diffuse in extracellular-matrix micro-environments. However, stochasticity-buffering mechanisms must function, even in fluctuating micro-environments, to ensure developmental precision and robustness. High levels of gene expression and/or efficient operation of negative feedback loops (e.g. a protein encoded by a gene negatively controls its own transcription) can reduce the effects derived from stochastic gene expression^47,48^. Hence, the presumable ability of s38 protein to provide developmentally regulated (stage 14) signals for its own gene repression (negative feedback loop) and the strong *s38*-gene expression being mechanistically induced during early choriogenesis (e.g. due to a gene-specific amplification/re-replication process) may not only alleviate -putative- quantitative differences among different follicle cells in the same or different follicles, but also guarantee that the s38 protein will be synthesized and secreted in adequate amounts required for an architecturally sole and robust chorion structure. Functionally expanding a previous report that describes the competence of macromolecular crowding to reduce gene-expression stochasticity (noise) by limiting transcription-factor diffusion^49^, we herein suggest that s38 protein serves as a regulator of chorion-patterning stochasticity, likely acting as an inducer of chorion-protein crowding and repressor of chorion-protein diffusion. If so, the RNAi-mediated reduction of s38-protein content below a certain threshold can sensitize choriogenesis to stochastic variation in morphogenetic events and also disable or weaken negative-feedback-loop’s strength, leading to the diverse dysmorphias obtained and the different *s38*-gene transcription activities observed between dysmorphic follicles carrying short or comparatively longer dorsal appendages. Interestingly, a similar type of incomplete penetrance has been recently described, suggesting a role of Idgfs (Imaginal disc growth factors) in the stochastic variation regarding coordination and timing of events during dorsal-appendages morphogenesis^50^.

Regarding the morphogenetic importance of s38-protein downregulation in other chorion structures, such as the innermost chorionic layer and the exochorion, no detectable defects in the s38-targeted follicles could be observed, suggesting a non-essential, or redundant, role of s38 protein in their assembly and architecture. In accordance, the fibrous exochorion is synthesized during the developmental stage 14 of oogenesis^5^, which is characterized by strong expression of the 3^rd^ -and not X (carries the *s36* and *s38* genes)- chromosome cluster-chorion genes^9,14,40,46^. Intriguingly, RNAi-mediated targeting of the developmentally medium/late-chorion genes *s15*, *s16*, *s18* and *s19*, of the 3^rd^-chromosome cluster, in ovarian follicle cells, caused production of eggs with insignificant defects in their chorion structure and patterning (data not shown), indicating their non-essential, or redundant, role in chorion biogenesis. However, the alternative scenario of inefficient silencing could not be excluded, since these genes are developmentally subjected to an approximately 60–80 round-amplification mechanism^12,20,21^.

Data derived from *Cp36*^*dec2-1*^ mutation-based^28^ and chorion-immunolocalization^51^ studies have previously suggested a structural role for the s38-chorion protein in vitelline-membrane architecture. Furthermore, a consistent fraction of eggs derived from *Cp36*^*dec2-1*^ mutant flies burst upon brief exposure to bleach, while the remaining ones demonstrate significant uptake of the neutral-red dye^28,52^. However, in our experiments, no statistically significant differences could be observed in egg rupture or neutral-red-dye penetration between control and s38-targeted (de-chorionated) eggs (Fig. S3), thus indicating the non-essential (or redundant) role of s38 protein in vitelline-membrane assembly and function.

Aiming at measuring chorion’s physical properties and especially mechanical strength, an AFM-dependent approach was herein employed. Force-distance curves and adhesion-force values presented a nearly 3x reduction in the mechanical strength of s38-downregulated -laid- eggs, as compared to control ones. It should be noted that local-surface structural changes significantly alter the values of adhesion forces applied. Specifically, the adhesion-force value of an AFM tip in contact with poor density-packed surfaces, structurally disordered and short self-assembled chains is smaller than the one of well-packed and rigid surfaces, with long self-assembled molecular chains^53^. The observed inability of s38-downregulated eggs to resist deformation induced by mechanical stress might be, among others, associated with defective eggshell hardening, likely due to inefficient and/or aberrant formation of di- and tri-tyrosine covalent bonds among chorion proteins^15,54^. Inclination (slope) distribution is a robust statistical method to investigate surface auto-correlation for finding periodic structures prevalent in the surface topography, and to also quantify pattern orientation and asymmetry. It is additionally used to correlate variation in the topography with other parameters, such as energy flow in a surface at the nano-scale level^55^. Interestingly, control -laid- eggs, typified by hexagonal/pentagonal follicular imprints on their surface, are presented with relatively narrow inclination-angle distributions. In contrast, s38-targeted -laid- eggs, due to the lack of periodic structures and pattern orientations on their surface, appear with comparatively wider inclination-angle distributions, thus indicating an altered chorion’s biochemical composition. Among others, it may be the s38 lack-induced asymmetry of surface topography that renders ovarian eggshell a fragile and mechanically vulnerable structure. Loss of follicular imprints and surface symmetry could severely compromise the mechano-transduction pathways presumably controlling fly’s oogenesis.

With respect to systemic malfunctions at the organism level, s38-targeted flies carrying severely dysmorphic follicles (e.g. with short dorsal appendages), generated by the strong downregulation of s38-chorion protein, are presented with impaired fertility, most likely caused by defects in micropyle’s integrity and therefore egg-fertilization failure, as previously reported for s36-depleted flies^15^. Micropyle consists of an inner part structured by a protrusion of vitelline membrane and an outer part composed of a chorionic protrusion that contains a canal through which the spermatozoon enters the egg^42^. The chorionic part of micropyle entails a rather spongy and fibrous endochorion, and its biogenesis commences at stage 12 of follicle’s development^42,43^. Taken together, it seems that in s38-downregulated follicles the lack of solid and robust endochorionic framework results in collapse of chorion material inside the micropylar pore, causing its structural blockage and thus leading to fertilization failure, similarly to the mechanism previously demonstrated for the s36-deficient flies^15^. This is further supported by the finding that s38-targeted -laid- eggs carrying short dorsal appendages, and thus severely disrupted endochorion-based structures, are associated with reduced levels of eclosion, as compared to -laid- dysmorphic eggs with long(er) in length dorsal appendages. In accordance, we have recently described the inability of spermatozoa to pass through micropyle in eggs being depleted of the s36-chorion protein^56^, which is developmentally co-expressed with the s38 one, and its absence also results in severe endochorionic malformation and impaired fertility^15^.

Conclusively, our findings unearth the essential contribution of s38 protein to eggshell patterning in *D. melanogaster* during late oogenesis. s38 proves to serve as a major architectural protein, orchestrating and structuring the molecular assembly and organization of a highly ordered tripartite endochorionic scaffold, which critically supports chorion’s regional specialization and radial complexity. Biogenesis of a mechanically robust and functionally effective endochorion requires the precise spatial and temporal coordination of *s38*-gene expression. Downward deviation from the optimal level of s38 protein causes pathogenic variation in chorion structure. Therefore, it may be the loss of s38 protein that sensitizes ovarian choriogenesis to stochastic variation in its coordination and timing.

## Materials and Methods

### *Drosophila melanogaster* strain stocks and culturing conditions

The *D. melanogaster* transgenic fly strains P{w[+mW.hs] = GawB}c355, w[1118] (BL: 3750) and y[*] w[*]; P{w[+mC] = UAS-2xEGFP} AH2 (BL: 6874) were obtained from Bloomington *Drosophila* Stock Center (NIH P40OD018537) (IN, USA). The *D. melanogaster* transgenic fly strains UAS-s38_RNAi (Transformant IDs: 106008 and 41344) were provided by Vienna *Drosophila* Resource Center (Vienna, Austria)^57^. Fly stocks were maintained at 25 °C and fed on standard diet (6.4% rice flour, 5% tomato paste, 3.2% sugar, 0.8% yeast, 0.8% agar, 0.4% ethanol and 0.4% propionic acid).

### RT-PCR

Control and s38-targeted female transgenic flies were dissected in *Drosophila* Ringer’s buffer and stage 14 follicles were hand-collected from isolated ovaries. Total RNA was extracted following a Trizol-based protocol (Molecular Research Center Inc., OH, USA), according to manufacturer’s instructions. One microgram of RNA was reverse transcribed, using an oligo-[dT]_12–18_ primer and the MMLV reverse transcriptase (Thermo Fisher Scientific Inc.-Life Technologies-Invitrogen, MA, USA). Produced cDNA was amplified by PCR with a Bio-Rad T100 Thermocycler (Bio-Rad, CA, USA), using *s38* gene-specific oligonucleotide primers (Table S1). *Actin* served as gene of reference (control). PCR fragments were resolved in 2% agarose gels, according to standard procedures. RT-PCR assays were repeated three times, using independent fly crosses.

### Proteοmics: peptide generation and liquid chromatography-tandem mass spectrometry (LC-MS/MS)

Total-protein samples were prepared from thirty pairs of s38-targeted (c355 > s38_RNAi) fly ovaries. Protein purification and processing for tryptic-peptide generation and extraction were carried out as previously described^38^. Produced peptides were analyzed using an LTQ Orbitrap Elite instrument (Thermo Scientific, IL, USA), with the mass spectrometer being coupled to a Dionex Ultimate 3000 HPLC system. The extracted ion chromatogram was further processed using the Proteome Discoverer software (Thermo Scientific, IL, USA) and the Sequest search engine. The database chosen for protein identification searches was the *D. melanogaster* reference proteome from UniProt 2.16. Identification criteria included a precursor-mass tolerance of 10 ppm and a fragment-mass tolerance of 0.05 Da. Trypsin was selected as the cleavage enzyme with a maximum of “0” missed-cleavage parameter. A false-discovery rate-threshold of 0.5% ensured the reliability of protein identification procedure.

### Hatchability assay

Five pairs of three-day-old adult flies were transferred into fresh media and remained there for 12 h to mate, and lay eggs. Hatching efficiency was quantified by comparing the number of laid eggs (as measured by a stereomicroscope) with the number of third-instar larvae developed. Experiments were performed three different times, using independent genetic crosses.

### Egg collection and scoring

Control and s38-targeted female flies were fattened on wet yeast overnight and, then, placed in apple agar plates for 2 h. Eggs were, next, collected and scored for eggshell abnormalities in a BMS stereomicroscope, model 74958. For egg size measurements, collected eggs were placed on a glass slide with 1x PBS and imaged under a Nikon Eclipse TE2000S microscope (Nikon, Tokyo, Japan).

### Light microscopy (LM)

Transgenic follicles, over-expressing the eGFP protein, were visualized under a Nikon confocal laser scanning microscope (CLSM), model Digital Eclipse C1 (Nikon, Tokyo, Japan). For the neutral-red staining assay, de-chorionated follicles were incubated for 10 min at room temperature with a solution of 5 mg/ml neutral red (Sigma-Aldrich, MI, USA) in 1x PBS, subsequently washed five times with 1x PBS containing 0.05% Triton X-100 and finally visualized under a BMS stereomicroscope. LM imaging was repeated three times, using independent fly crosses.

### Scanning electron microscopy (SEM)

Surface structural architecture of laid transgenic eggs was visualized through SEM technology. Preparation of samples for SEM analysis was performed as previously described^15^. Next, samples were subjected to a critical-point drying (Samdri 780 A; Tousimis, MD, USA) process, attached on aluminum stubs, coated with gold-palladium (60:40) in a sputter-coating apparatus (Samsputter 2a; Tousimis, MD, USA) and finally visualized under a Philips 515 scanning electron microscope. SEM-imaging experiments were performed three times, using independent genetic crosses.

### Transmission electron microscopy (TEM)

*D. melanogaster* transgenic follicles were processed for TEM analysis as previously described^15^. Ultrathin sections were mounted on uncoated copper grids, stained with uranyl acetate and lead citrate, and viewed under a Philips EM300 transmission electron microscope. TEM-imaging experiments were repeated three different times, collecting data from independent fly crosses.

### Atomic force microscopy (AFM)

For a closer to an atomic resolution mapping of the morphological and mechanical landscape of *D. melanogaster* egg surface, an atomic force microscope (di Innova; Bruker, CA, USA), with resolution of a few nanometers, was suitably engaged. AFM imaging and surface analysis of the ovarian eggshell were performed at ambient conditions, for both control (c355-GAL4/+) and s38-downregulated (c355 > s38_RNAi) laid eggs. The AFM-surface morphology of the control samples was probed in tapping mode, using a 40 nN/nm stiff silicon cantilever (Bruker MPP-11123-10) at 300 kHz, while for the s38-downregulated eggs, surface analysis was carried out in contact mode using a softer silicon nitride MLCT cantilever with 0.03 nN/nm at 15 kHz (“D” tip). Cantilever’s stiffness was obtained by applying the thermal tune mode of the instrument. High-resolution surface images were captured at a scanning rate of 0.5 Hz. AFM imaging was carried out on different sample regions and scan sizes, for both control and s38-downregulated samples. Image processing and data acquisition were performed using the SPMLab v7.0 software.

To probe mechanical properties, like elasticity (Young’s modulus) and adhesion force, of both control and s38-targeted ovarian-eggshell types, force-distance (F-D, indentation versus loading force) curves were recorded on several points, regularly distributed over a selected area away from the edges of each examined egg. The stiffness variation was identified via elastic modulus, using the same cantilever type (Bruker MPP-11123-10) for both control and s38-downregulated laid eggs. Young’s modulus values were provided by fitting the contact-region data of the retract F-D curve to the Hertz-Sneddon model^58^ and applying a hysteresis correction on force-curve pairs. Of note, it was assumed that (a) during the contact phase, the cantilever deflection was balanced smoothly by the cantilever forces exerted on the tip from each sample (repulsive interaction) and (b) excessive strains from the tip that have the capacity to induce damage on the surface of each sample were limited. In the retracting phase, the tip was undetached, until the point where the pulling force could overcome the adhesion force of the tip to the surface. Consequently, the “AFM pull-off force”, during the jump-off-contact stage, was used to extract the adhesion force at a nanoscale level. Commonly, the Poisson’s ratio for biological materials lies in the interval from 0.2 to 0.5 (0.5, for perfectly uncompressible materials). Since the actual Poisson’s ratio for control and s38-targeted eggshells was unknown, and given that the experiments were conducted in dry air, the value of 0.2 was used for both sample types. AFM-imaging experiments were carried out three times, collecting data from independent genetic crosses.

### Statistical analysis

The Statistical Package for Social Sciences (IBM SPSS v23.0 for Windows IBM Corp., NY, USA) was used for the statistical analysis. For Figs 1B, 2(C–F), 6(C–E) and 7B, the results were presented as mean ± SSD (Sample Standard Deviation), and the significance meaning of s38 downregulation was evaluated using one-way ANOVA. For Fig. S2, after testing all variables for normal distribution profile, the in between groups differences were evaluated by Mann-Whitney analysis. Significance was accepted at a *p* value of less than 0.05.

## Electronic supplementary material

Supplementary Information
