# Supplementary material for: The indispensable contribution of s38 protein to ovarian-eggshell morphogenesis in Drosophila melanogaster
Source: Sci Rep. 2018 Oct 31;8:16103. doi: 10.1038/s41598-018-34532-2 (PMC6208399; doi:10.1038/s41598-018-34532-2)
Supplement: Supplementary file 1 — Supplementary Information [file 41598_2018_34532_MOESM1_ESM.pdf]

Supplementary Information

The indispensable contribution of s38 protein to ovarian-eggshell morphogenesis in *Drosophila melanogaster*

Athanassios D. Velentzas[1], Panagiotis D. Velentzas[1,a], Stamatia A. Katarachia[1], Athanasios K. Anagnostopoulos[2], Niki E. Sagioglou[1], Eleni V. Thanou[1], Maria M. Tsioka[1], Vassiliki E. Mpakou[1], Zoe Kollia[3], Vassilios E. Gavriil[3], Issidora S. Papassideri[1], George Th. Tsangaris[2], Alkiviadis-Constantinos Cefalas[3], Evangelia Sarantopoulou[3] and Dimitrios J. Stravopodis[1,*]

[1]Section of Cell Biology and Biophysics, Department of Biology, School of Science, National and Kapodistrian University of Athens (NKUA), Athens, Greece

[2]Systems Biology Center, Biomedical Research Foundation of the Academy of Athens (BRFAA), Athens, Greece

[3]Theoretical and Physical Chemistry Institute, National Hellenic Research Foundation (NHRF), Athens, Greece

[a]Current Address: Department of Cancer Biology, Medical School, University of Massachusetts, Worcester, Massachusetts (MA), USA

[*]**Corresponding Author**:

Dimitrios J. Stravopodis, *BSc*, *MSc*, *PhD*, Assistant Professor, Section of Cell Biology and Biophysics, Department of Biology, School of Science, National and Kapodistrian University of Athens (NKUA), Panepistimiopolis, Zografou 157 84, Athens, Greece (GR). Tel.: +30 210 727 4105, Fax: +30 210 727 4742, E-mail: dstravop@biol.uoa.gr

Figure S1

Independent validation of eggs' diverse phenotypes with the use of s38-RNAi GD stock line. (A) Far-off view light-microscopy image of several s38-targeted (c355>s38GD_RNAi) eggs being characterized by diverse dysmorphias in their dorsal appendages. (B, C) Representative s38GD-downregulated eggs with short and thin dorsal appendages that also lack the typical paddle-shaped tips. (D, E) Eggs derived from s38GD-targeted flies with comparatively longer, but always shorter, dorsal appendages, as compared to the ones of control eggs. Total number of eggs examined from the c355>s38GD_RNAi flies: 368. (F) Eclosion-efficiency graphs of both control (c355-GAL4/+) (n=60) and s38-downregulated (c355>s38GD_RNAi) (n=60) flies, for a thirty-days period. DA: Dorsal Appendage(s). Scale Bars: 50 μm.

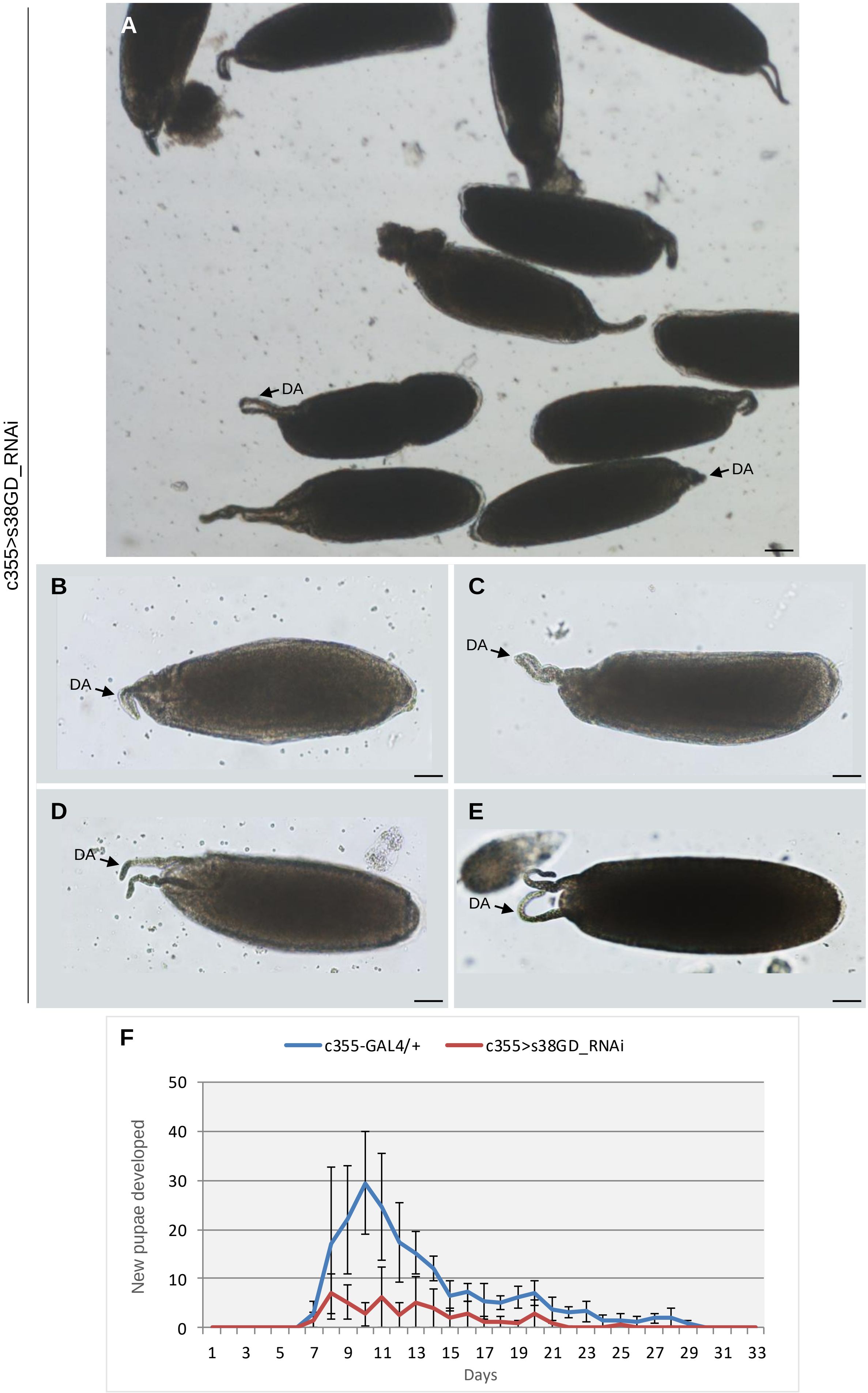
c355>s38GD_RNAi
A
DA
DA
B
DA
C
DA
D
DA
E
DA
F
c355-GAL4/+
c355>s38GD_RNAi
New pupae developed
0
10
20
30
40
50
1
3
5
7
9
11
13
15
17
19
21
23
25
27
29
31
33
Days

Figure S2

The importance of s38 protein in the maintenance of egg's elliptical shape. Box-plot graphical presentations of the main-body egg (A) length and (B) length to width ratio (excluding the dorsal appendages) between control (n=121) and s38-targeted (n=133) laid eggs. *: $p < 0.05$.

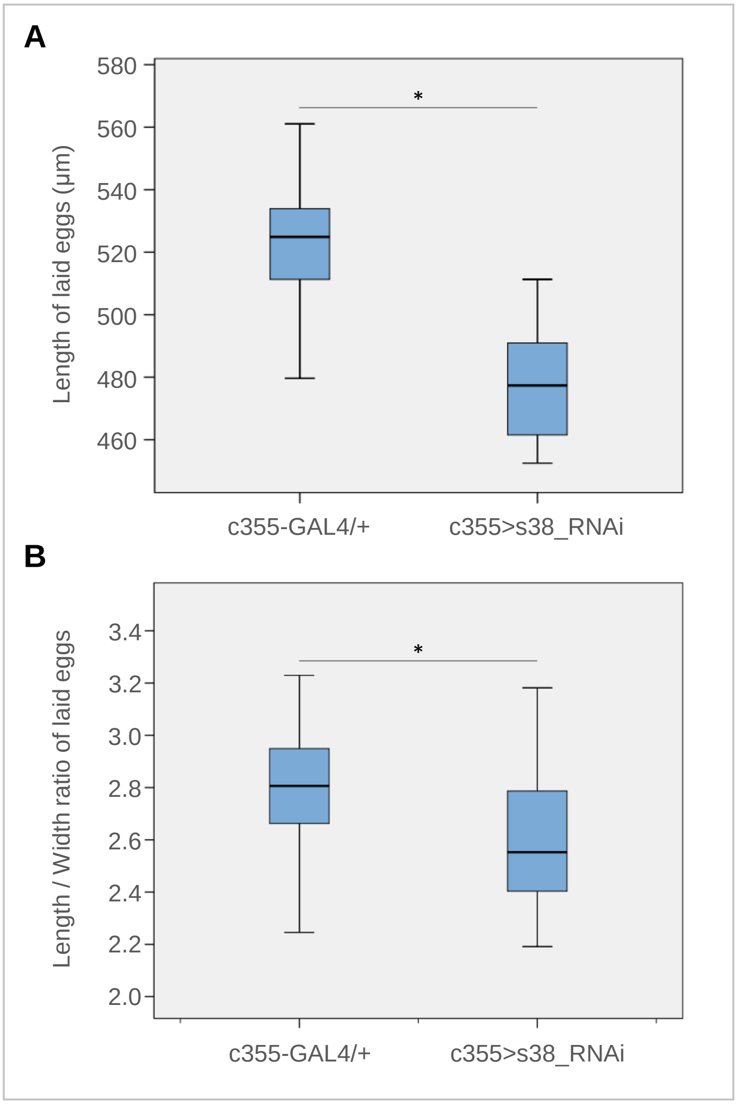
A
Length of laid eggs (µm)
580
560
540
520
500
480
460
*
c355-GAL4/+
c355>s38_RNAi
B
Length / Width ratio of laid eggs
3.4
3.2
3.0
2.8
2.6
2.4
2.2
2.0
*
c355-GAL4/+
c355>s38_RNAi

Figure S3

s38-targeted eggs appear with unaffected vitelline-membrane permeability. Light-microscopy images of (A) control (c355-GAL4/+) (n=102) and (B) s38-downregulated (c355>s38_RNAi) (n=138) laid de-chorionated eggs, after their incubation with neutral red, demonstrating their impermeability to the dye. E: Embryo. Scale Bars: 50 μm.

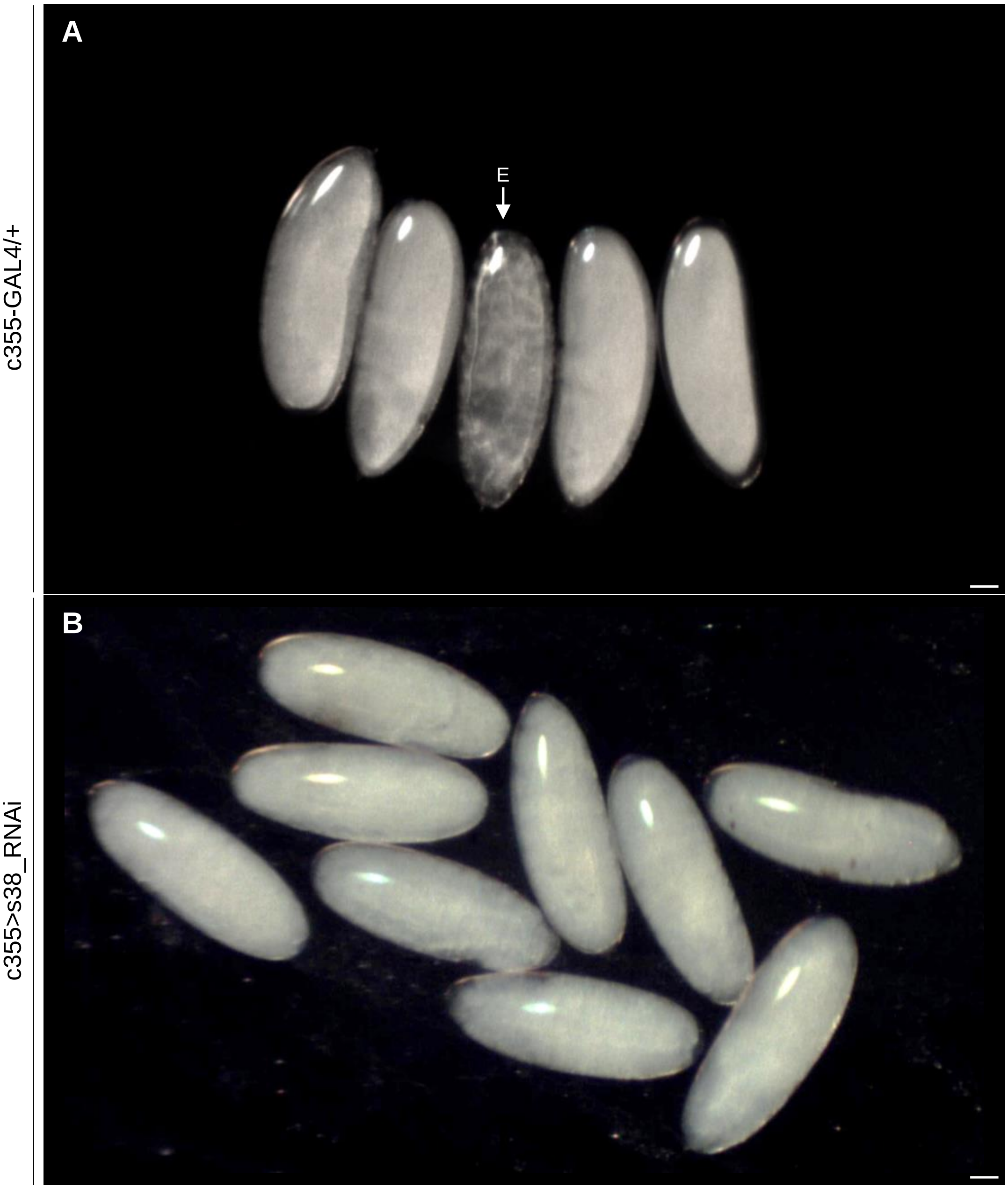
A
E
B
c355-GAL4/+
c355>s38_RNAi

Supplementary Table S1

Gene name, oligonucleotide-primer sequence (F: forward; R: reverse), molecular size (bp) and annealing temperature (Ta) of the amplified PCR products for the, herein, examined genes.

Gene Name	Primer Sequence	Molecular Size (bp) of PCR Product	Ta
Chorion protein 38 Transcript variants A and B (CG11213)	F: 5′-CGA GAT CGA CCT ACA TTT G-3′	213	53ºC
	R: 5′-ATT CGA GAG CAC CAG CAC C-3′		
Chorion protein 38 Transcript variant B (CG11213)	F: 5′-TTA CGG ATT GGA TGA CTG CTG-3′	228	54ºC
	R: 5′-GCA AAG AAA CCT GAG TGC CAC-3′		

Supplementary Table S2

Protein library (n=2,123 members) of s38-downregulated (c355>s38_RNAi) *D. melanogaster* ovaries, specifying, for each identified protein, the following parameters: (A) UniProt accession number, (B) name and description, (C) Mascot score and (D) sequence coverage. Additional important features of the nLC-MS/MS proteomics analysis, such as the number of unique peptides, the number of amino-acid residues, the molecular weight (kDa) and the isoelectric point, are also described.

Accession	Description	Score	Coverage	# Proteins	# Unique Peptides	# Peptides	# PSMs	Area	# AAs	MW [kDa]	calc. pI
P02844	Vitellogenin-2 OS=Drosophila melanogaster GN=Yp2 PE=1 SV=2 - [VIT2_DROME]	579.43	52.04	2	22	23	329	1.523E10	442	49.6	7.96
P02828	Heat shock protein 83 OS=Drosophila melanogaster GN=Hsp83 PE=1 SV=1 - [HSP83_DROME]	563.00	51.46	3	40	43	269	2.667E9	717	81.8	5.02
P11147	Heat shock 70 kDa protein cognate 4 OS=Drosophila melanogaster GN=Hsc70-4 PE=1 SV=3 - [HSP7D_DROME]	473.94	60.06	6	32	41	218	3.569E9	651	71.1	5.52
P02843	Vitellogenin-1 OS=Drosophila melanogaster GN=Yp1 PE=1 SV=1 - [VIT1_DROME]	471.47	63.10	1	23	24	316	1.734E10	439	48.7	7.69
M9NFS1	Terribly reduced optic lobes, isoform AQ OS=Drosophila melanogaster GN=trol PE=4 SV=2 - [M9NFS1_DROME]	315.49	22.77	18	5	70	163	2.703E8	4299	476.3	4.88
M9NGK3	Terribly reduced optic lobes, isoform AL OS=Drosophila melanogaster GN=trol PE=4 SV=2 - [M9NGK3_DROME]	315.32	24.81	4	1	66	160	2.703E8	3728	412.5	4.92
P18169	Defective chorion-1 protein, FC125 isoform OS=Drosophila melanogaster GN=dec-1 PE=2 SV=2 - [DEC11_DROME]	314.34	44.29	6	34	34	113	1.086E9	1208	137.4	5.29
E1JHJ3	Myosin heavy chain, isoform O OS=Drosophila melanogaster GN=Mhc PE=4 SV=1 - [E1JHJ3_DROME]	277.96	34.91	5	3	68	127	3.583E8	1962	224.4	6.19
M9ND95	Myosin heavy chain, isoform U OS=Drosophila melanogaster GN=Mhc PE=4 SV=1 - [M9ND95_DROME]	269.67	34.22	4	2	67	125	3.583E8	1949	222.8	6.25
P10987	Actin-5C OS=Drosophila melanogaster GN=Act5C PE=1 SV=4 - [ACT1_DROME]	240.17	60.64	1	1	20	164	6.286E9	376	41.8	5.48
P02572	Actin-42A OS=Drosophila melanogaster GN=Act42A PE=1 SV=3 - [ACT2_DROME]	238.63	52.39	1	1	17	161	6.286E9	376	41.8	5.48
M9PF14	Trailer hitch, isoform C OS=Drosophila melanogaster GN=tral PE=4 SV=1 - [M9PF14_DROME]	233.98	49.85	2	0	22	84	1.403E9	656	69.7	9.48
Q05825	ATP synthase subunit beta, mitochondrial OS=Drosophila melanogaster GN=ATPsyn-beta PE=2 SV=3 - [ATPB_DROME]	233.47	40.59	3	16	16	99	1.533E9	505	54.1	5.27
P29844	Heat shock 70 kDa protein cognate 3 OS=Drosophila melanogaster GN=Hsc70-3 PE=2 SV=2 - [HSP7C_DROME]	231.65	47.41	2	26	31	110	3.040E9	656	72.2	5.36
Q9VTZ0	GH08269p OS=Drosophila melanogaster GN=tral PE=1 SV=3 - [Q9VTZ0_DROME]	230.64	49.54	2	1	22	84	1.403E9	652	69.3	9.54
M9PFF9	Trailer hitch, isoform D OS=Drosophila melanogaster GN=tral PE=4 SV=1 - [M9PFF9_DROME]	209.85	48.76	2	1	18	73	1.403E9	646	68.7	9.54
P07182	Chorion protein S36 OS=Drosophila melanogaster GN=Cp36 PE=3 SV=2 - [CH36_DROME]	207.98	33.45	1	9	9	75	1.329E9	284	29.9	8.69
P06607	Vitellogenin-3 OS=Drosophila melanogaster GN=Yp3 PE=1 SV=1 - [VIT3_DROME]	203.85	60.95	1	23	23	178	1.064E10	420	46.1	8.50
E1JGP0	Actin 57B, isoform C OS=Drosophila melanogaster GN=Act57B PE=2 SV=1 - [E1JGP0_DROME]	199.54	58.33	2	1	18	127	6.286E9	360	40.2	5.83
P10981	Actin-87E OS=Drosophila melanogaster GN=Act87E PE=1 SV=1 - [ACT5_DROME]	196.17	55.85	3	1	18	126	6.286E9	376	41.8	5.48
P13060	Elongation factor 2 OS=Drosophila melanogaster GN=EF2 PE=1 SV=4 - [EF2_DROME]	195.05	47.04	1	33	33	117	3.173E9	844	94.4	6.60
P05389	60S acidic ribosomal protein P2 OS=Drosophila melanogaster GN=RpLP2 PE=1 SV=1 - [RLA2_DROME]	188.97	53.98	1	8	8	87	9.276E8	113	11.7	4.70
P08736	Elongation factor 1-alpha 1 OS=Drosophila melanogaster GN=Ef1alpha48D PE=1 SV=2 - [EF1A1_DROME]	185.42	53.13	2	22	22	121	1.434E10	463	50.3	9.07
Q7KN62	Transitional endoplasmic reticulum ATPase TER94 OS=Drosophila melanogaster GN=TER94 PE=1 SV=1 - [TERA_DROME]	182.86	47.82	2	34	34	90	4.569E8	801	88.8	5.35
O18640	Guanine nucleotide-binding protein subunit beta-like protein OS=Drosophila melanogaster GN=Rack1 PE=1 SV=2 - [GBLP_DROME]	182.62	63.21	2	19	20	106	1.892E9	318	35.6	7.47
P09180	60S ribosomal protein L4 OS=Drosophila melanogaster GN=RpL4 PE=1 SV=2 - [RL4_DROME]	170.23	47.13	2	19	19	68	1.571E9	401	45.0	11.47
O02649	60 kDa heat shock protein, mitochondrial OS=Drosophila melanogaster GN=Hsp60 PE=1 SV=3 - [CH60_DROME]	167.65	58.12	2	30	30	75	6.141E8	573	60.8	5.49
P19889	60S acidic ribosomal protein P0 OS=Drosophila melanogaster GN=RpLP0 PE=1 SV=1 - [RLA0_DROME]	161.75	56.78	1	18	18	74	2.164E9	317	34.2	6.95
Q24560	Tubulin beta-1 chain OS=Drosophila melanogaster GN=betaTub56D PE=1 SV=2 - [TBB1_DROME]	161.36	39.60	5	10	15	79	1.711E9	447	50.1	4.86
Q9VMA3	Protein cup OS=Drosophila melanogaster GN=cup PE=1 SV=3 - [CUP_DROME]	158.73	40.91	1	25	25	71	2.825E8	1117	125.6	8.85
P54399	Protein disulfide-isomerase OS=Drosophila melanogaster GN=Pdi PE=2 SV=1 - [PDI_DROME]	154.01	55.44	1	22	22	103	6.349E8	496	55.7	4.82
P07186	Chorion protein S19 OS=Drosophila melanogaster GN=Cp19 PE=2 SV=1 - [CH19_DROME]	153.87	67.63	1	7	7	60	1.047E9	173	18.5	9.36
Q06559	40S ribosomal protein S3 OS=Drosophila melanogaster GN=RpS3 PE=1 SV=1 - [RS3_DROME]	146.34	71.54	1	21	21	97	1.915E9	246	27.5	9.39
Q9W0S7	LD20211p OS=Drosophila melanogaster GN=Tudor-SN PE=1 SV=1 - [Q9W0S7_DROME]	146.14	33.80	1	28	28	71	3.364E8	926	103.0	8.05
P35381	ATP synthase subunit alpha, mitochondrial OS=Drosophila melanogaster GN=blw PE=1 SV=2 - [ATPA_DROME]	144.14	46.74	3	30	30	76	1.177E9	552	59.4	9.01
P14130	40S ribosomal protein S14 OS=Drosophila melanogaster GN=RpS14a PE=1 SV=1 - [RS14_DROME]	140.13	48.34	1	9	9	50	2.630E9	151	16.3	10.35
O62619	Pyruvate kinase OS=Drosophila melanogaster GN=PyK PE=2 SV=2 - [KPYK_DROME]	139.63	47.65	1	21	21	75	3.277E8	533	57.4	7.44
P21187	Polyadenylate-binding protein OS=Drosophila melanogaster GN=pAbp PE=1 SV=3 - [PABP_DROME]	139.13	43.38	1	19	19	62	1.006E9	634	69.9	9.31
Q9VIE8	Aconitase, isoform B OS=Drosophila melanogaster GN=Acon PE=2 SV=2 - [Q9VIE8_DROME]	138.54	41.68	2	24	24	71	2.296E8	787	85.3	8.24
Q9VUC1	CG6603-PA, isoform A OS=Drosophila melanogaster GN=Hsc70Cb PE=2 SV=1 - [Q9VUC1_DROME]	131.19	44.15	2	26	26	63	3.379E8	804	88.4	5.43
Q7K3J0	CG8258 OS=Drosophila melanogaster GN=CG8258 PE=2 SV=1 - [Q7K3J0_DROME]	128.11	47.44	1	22	22	67	3.894E8	546	59.4	5.31
Q868Z9	Papilin OS=Drosophila melanogaster GN=Ppn PE=1 SV=2 - [PPN_DROME]	127.49	16.32	1	31	31	70	2.530E8	2898	312.8	4.58
Q9W392	CG7033 OS=Drosophila melanogaster GN=CG7033 PE=2 SV=2 - [Q9W392_DROME]	127.30	50.65	2	21	21	55	6.323E8	535	58.0	5.85
Q9VQL7	CG3523, isoform A OS=Drosophila melanogaster GN=CG3523 PE=4 SV=1 - [Q9VQL7_DROME]	123.09	18.50	2	33	33	63	1.466E8	2438	266.3	6.37
Q9V496	Apolipophorins OS=Drosophila melanogaster GN=Rfabg PE=1 SV=2 - [APLP_DROME]	122.86	17.22	1	50	50	76	1.827E8	3351	372.4	7.97
Q9VHP0	ATP-dependent RNA helicase bel OS=Drosophila melanogaster GN=bel PE=1 SV=1 - [DDX3_DROME]	122.53	40.23	2	22	24	62	1.804E8	798	85.0	7.53
P15357	Ubiquitin-40S ribosomal protein S27a OS=Drosophila melanogaster GN=RpS27A PE=1 SV=2 - [RS27A_DROME]	122.13	60.26	4	3	9	57	2.693E9	156	17.9	9.77
P46223	60S ribosomal protein L7a OS=Drosophila melanogaster GN=RpL7A PE=1 SV=2 - [RL7A_DROME]	121.32	37.27	1	16	16	52	2.749E9	271	30.7	10.71
Q8IPM3	Growl, isoform B (Fragment) OS=Drosophila melanogaster GN=lost PE=4 SV=2 - [Q8IPM3_DROME]	118.83	43.93	2	22	22	53	4.917E8	535	58.9	9.32
Q7KSU6	CG8036, isoform D OS=Drosophila melanogaster GN=CG8036 PE=4 SV=1 - [Q7KSU6_DROME]	118.61	33.79	3	20	20	51	6.747E8	580	63.0	6.80

Accession	Description	Score	Coverage	# Proteins	# Unique Peptides	# Peptides	# PSMs	Area	# AAs	MW [kDa]	calc. pI
P92177	14-3-3 protein epsilon OS=Drosophila melanogaster GN=14-3-3epsilon PE=1 SV=2 - [1433E_DROME]	118.54	60.69	1	17	19	62	1.049E9	262	29.8	4.78
Q9VHL2	LD47396p OS=Drosophila melanogaster GN=Tcp-1eta PE=2 SV=2 - [Q9VHL2_DROME]	118.39	49.45	1	24	24	70	5.049E8	544	59.3	6.34
Q7KM15	Beta NAC homolog OS=Drosophila melanogaster GN=bic PE=2 SV=1 - [Q7KM15_DROME]	115.15	81.07	2	15	15	43	9.985E8	169	17.7	7.49
Q9VFT4	AT27578p OS=Drosophila melanogaster GN=rin PE=1 SV=1 - [Q9VFT4_DROME]	114.98	29.71	1	16	16	47	4.305E8	690	74.9	7.37
Q9V3P0	Peroxiredoxin 1 OS=Drosophila melanogaster GN=Jafrac1 PE=1 SV=1 - [PRDX1_DROME]	113.94	45.36	1	10	10	54	6.034E8	194	21.7	5.71
Q8T0L3	GH24511p OS=Drosophila melanogaster GN=Uba1 PE=2 SV=1 - [Q8T0L3_DROME]	113.81	29.55	1	25	25	61	2.122E8	1191	130.7	5.29
O61380	Eukaryotic translation initiation factor 4G, isoform A OS=Drosophila melanogaster GN=eIF4G PE=2 SV=3 - [O61380_DROME]	112.18	28.99	2	31	31	63	1.722E8	1666	183.8	7.83
Q9NJH0	Elongation factor 1-gamma OS=Drosophila melanogaster GN=Ef1gamma PE=2 SV=2 - [EF1G_DROME]	112.11	34.57	1	15	15	50	1.554E9	431	48.9	7.05
P07487	Glyceraldehyde-3-phosphate dehydrogenase 2 OS=Drosophila melanogaster GN=Gapdh2 PE=1 SV=2 - [G3P2_DROME]	111.78	51.51	2	4	14	56	2.189E9	332	35.3	8.44
P06603	Tubulin alpha-1 chain OS=Drosophila melanogaster GN=alphaTub84B PE=2 SV=1 - [TBA1_DROME]	111.54	33.11	3	11	11	61	7.185E8	450	49.9	5.14
P98163	Putative vitellogenin receptor OS=Drosophila melanogaster GN=yl PE=1 SV=2 - [YL_DROME]	110.69	17.24	1	22	22	54	1.734E8	1984	219.4	6.20
Q02748	Eukaryotic initiation factor 4A OS=Drosophila melanogaster GN=eIF-4a PE=1 SV=3 - [IF4A_DROME]	109.59	42.43	1	17	19	61	1.216E9	403	45.8	5.66
P48605	T-complex protein 1 subunit gamma OS=Drosophila melanogaster GN=Cctgamma PE=2 SV=2 - [TCPG_DROME]	109.07	49.08	2	25	25	59	4.988E8	544	59.4	6.80
Q8MLY8	40S ribosomal protein S8 OS=Drosophila melanogaster GN=RpS8 PE=1 SV=1 - [RS8_DROME]	108.71	49.04	1	11	11	45	1.846E9	208	23.7	10.48
O16797	60S ribosomal protein L3 OS=Drosophila melanogaster GN=RpL3 PE=1 SV=3 - [RL3_DROME]	106.89	50.00	1	22	22	61	1.583E9	416	46.9	10.24
Q9VS34	60S ribosomal protein L18 OS=Drosophila melanogaster GN=RpL18 PE=1 SV=1 - [RL18_DROME]	103.79	34.57	1	5	5	49	8.581E8	188	21.7	11.53
P31009	40S ribosomal protein S2 OS=Drosophila melanogaster GN=RpS2 PE=1 SV=2 - [RS2_DROME]	103.29	47.19	1	13	13	52	1.898E9	267	28.9	10.15
P23128	Putative ATP-dependent RNA helicase me31b OS=Drosophila melanogaster GN=me31B PE=1 SV=3 - [DDX6_DROME]	102.45	41.61	1	17	17	55	9.704E8	459	51.9	7.64
E1JHR5	Enolase, isoform F OS=Drosophila melanogaster GN=Eno PE=3 SV=1 - [E1JHR5_DROME]	102.12	52.42	2	21	21	56	1.146E9	433	46.6	6.55
P07486	Glyceraldehyde-3-phosphate dehydrogenase 1 OS=Drosophila melanogaster GN=Gapdh1 PE=2 SV=2 - [G3P1_DROME]	100.30	51.20	2	3	13	53	1.947E9	332	35.3	8.18
Q9VEB1	CG7998 OS=Drosophila melanogaster GN=Mdh2 PE=2 SV=1 - [Q9VEB1_DROME]	100.15	33.93	1	12	12	40	5.276E8	336	35.3	9.11
X2JC80	40S ribosomal protein SA OS=Drosophila melanogaster GN=sta PE=3 SV=1 - [X2JC80_DROME]	99.99	39.63	2	9	9	45	1.460E9	270	30.2	4.87
P02517	Heat shock protein 26 OS=Drosophila melanogaster GN=Hsp26 PE=1 SV=2 - [HSP26_DROME]	98.32	62.02	1	9	9	52	7.528E8	208	23.0	7.56
Q9VEG6	Chorion peroxidase OS=Drosophila melanogaster GN=Pxt PE=2 SV=3 - [PERC_DROME]	98.11	35.60	1	19	19	44	2.585E8	809	90.5	6.90
Q9VN25	Eukaryotic translation initiation factor 3 subunit A OS=Drosophila melanogaster GN=eIF3-S10 PE=1 SV=1 - [EIF3A_DROME]	98.03	22.37	1	26	26	43	2.052E8	1140	133.8	9.01
P00334	Alcohol dehydrogenase OS=Drosophila melanogaster GN=Adh PE=1 SV=2 - [ADH_DROME]	96.86	51.95	1	9	9	50	6.400E8	256	27.7	7.96
Q24251	ATP synthase subunit d, mitochondrial OS=Drosophila melanogaster GN=ATPsyn-d PE=2 SV=2 - [ATP5H_DROME]	95.37	56.74	1	11	11	40	3.785E8	178	20.2	6.55
P18101	Ubiquitin-60S ribosomal protein L40 OS=Drosophila melanogaster GN=RpL40 PE=1 SV=2 - [RL40_DROME]	93.97	49.22	4	1	7	48	2.693E9	128	14.7	9.83
Q960D3	SD06613p OS=Drosophila melanogaster GN=vig2 PE=2 SV=1 - [Q960D3_DROME]	91.80	49.51	2	18	18	38	3.932E8	412	45.2	9.41
P48159	60S ribosomal protein L23 OS=Drosophila melanogaster GN=RpL23 PE=1 SV=2 - [RL23_DROME]	91.80	50.00	1	7	7	33	8.824E8	140	14.9	10.83
O96827	Probable elongation factor 1-beta OS=Drosophila melanogaster GN=Ef1beta PE=1 SV=3 - [EF1B_DROME]	90.28	66.22	1	10	10	43	1.372E9	222	24.2	4.58
Q9VFF0	CG3731, isoform A OS=Drosophila melanogaster GN=CG3731 PE=2 SV=2 - [Q9VFF0_DROME]	90.25	37.66	1	12	12	36	2.755E8	470	51.8	6.00
Q7KKI0	GM12270p OS=Drosophila melanogaster GN=Cct5 PE=2 SV=1 - [Q7KKI0_DROME]	89.56	40.22	2	18	19	53	2.366E8	542	59.2	6.25
X2JB48	Stress-sensitive B, isoform E OS=Drosophila melanogaster GN=sesB PE=3 SV=1 - [X2JB48_DROME]	88.76	54.18	3	19	19	79	1.483E9	299	32.9	9.77
Q9VPN5	CG2720-PA OS=Drosophila melanogaster GN=Hop PE=2 SV=1 - [Q9VPN5_DROME]	88.33	38.57	1	17	17	47	2.412E8	490	55.7	6.81
Q00174	Laminin subunit alpha OS=Drosophila melanogaster GN=LanA PE=1 SV=2 - [LAMA_DROME]	87.11	12.96	1	31	31	49	8.606E7	3712	410.9	5.22
P55935	40S ribosomal protein S9 OS=Drosophila melanogaster GN=RpS9 PE=1 SV=2 - [RS9_DROME]	86.54	46.67	2	14	14	50	9.425E8	195	22.6	10.61
O16043	Anon1A4 OS=Drosophila melanogaster GN=Df31 PE=2 SV=1 - [O16043_DROME]	86.31	85.79	1	15	15	39	7.204E8	183	18.8	4.27
P25007	Peptidyl-prolyl cis-trans isomerase OS=Drosophila melanogaster GN=Cyp1 PE=1 SV=2 - [PPIA_DROME]	86.06	59.03	8	13	13	46	2.524E9	227	24.7	9.22
Q9V3P6	26S proteasome non-ATPase regulatory subunit 1 OS=Drosophila melanogaster GN=Rpn2 PE=1 SV=1 - [PSMD1_DROME]	86.04	22.94	1	17	17	40	3.105E8	1020	113.1	5.19
P55830	40S ribosomal protein S3a OS=Drosophila melanogaster GN=RpS3A PE=1 SV=4 - [RS3A_DROME]	85.74	58.58	1	17	17	74	2.122E9	268	30.3	9.61
Q9W1B9	RE28824p OS=Drosophila melanogaster GN=RpL12 PE=1 SV=1 - [Q9W1B9_DROME]	85.62	46.06	1	9	9	41	2.356E9	165	17.7	9.07
P08570	60S acidic ribosomal protein P1 OS=Drosophila melanogaster GN=RpLP1 PE=1 SV=2 - [RLA1_DROME]	85.02	73.21	1	6	6	43	2.469E9	112	11.5	4.41
Q9VWG3	40S ribosomal protein S10b OS=Drosophila melanogaster GN=RpS10b PE=1 SV=2 - [RS10B_DROME]	84.74	48.13	2	8	8	36	7.661E8	160	17.9	9.80
P50887	60S ribosomal protein L22 OS=Drosophila melanogaster GN=RpL22 PE=1 SV=2 - [RL22_DROME]	84.08	50.17	1	13	13	28	1.915E9	299	30.6	10.11
P84040	Histone H4 OS=Drosophila melanogaster GN=His4 PE=1 SV=2 - [H4_DROME]	82.10	52.43	1	7	7	40	2.261E9	103	11.4	11.36
P41126	60S ribosomal protein L13 OS=Drosophila melanogaster GN=RpL13 PE=1 SV=1 - [RL13_DROME]	81.43	43.58	1	13	13	46	1.199E9	218	24.9	10.99
P07764	Fructose-bisphosphate aldolase OS=Drosophila melanogaster GN=Ald PE=1 SV=5 - [ALF_DROME]	80.92	50.42	2	16	16	46	3.827E8	361	39.0	7.40
Q9W237	40S ribosomal protein S16 OS=Drosophila melanogaster GN=RpS16 PE=1 SV=1 - [RS16_DROME]	80.53	55.41	1	10	10	41	1.014E9	148	16.8	10.17
P24156	Protein l(2)37Cc OS=Drosophila melanogaster GN=l(2)37Cc PE=2 SV=2 - [L2CC_DROME]	80.45	50.36	1	11	11	40	2.237E8	276	30.4	5.73

Accession	Description	Score	Coverage	# Proteins	# Unique Peptides	# Peptides	# PSMs	Area	# AAs	MW [kDa]	calc. pI
P13469	DNA-binding protein modulo OS=Drosophila melanogaster GN=mod PE=1 SV=2 - [MODU_DROME]	79.99	39.30	1	21	21	45	5.979E8	542	60.3	5.43
P36241	60S ribosomal protein L19 OS=Drosophila melanogaster GN=RpL19 PE=1 SV=2 - [RL19_DROME]	79.44	35.47	1	9	9	31	8.729E8	203	24.0	11.00
P09052	ATP-dependent RNA helicase vasa, isoform A OS=Drosophila melanogaster GN=vas PE=1 SV=3 - [VASA1_DROME]	79.24	25.57	2	12	13	33	2.134E8	661	72.3	5.66
Q9VTP4	60S ribosomal protein L10a-2 OS=Drosophila melanogaster GN=RpL10Ab PE=1 SV=2 - [R10AB_DROME]	78.92	43.32	1	11	11	54	1.924E9	217	24.3	9.86
P07185	Chorion protein S15 OS=Drosophila melanogaster GN=Cp15 PE=3 SV=1 - [CH15_DROME]	78.71	67.83	1	5	5	29	4.166E8	115	12.0	8.92
M9PI17	CG6084, isoform D OS=Drosophila melanogaster GN=CG6084 PE=4 SV=1 - [M9PI17_DROME]	78.08	40.51	5	11	11	42	4.817E8	316	35.9	6.80
P32100	60S ribosomal protein L7 OS=Drosophila melanogaster GN=RpL7 PE=1 SV=2 - [RL7_DROME]	77.50	39.68	1	14	14	42	1.640E9	252	29.5	10.89
Q9VFV9	DnaJ-like-2, isoform A OS=Drosophila melanogaster GN=Droj2 PE=2 SV=1 - [Q9VFV9_DROME]	77.38	41.69	1	14	14	34	1.637E8	403	45.2	6.48
Q9VV75	AT02348p OS=Drosophila melanogaster GN=CG4169 PE=2 SV=1 - [Q9VV75_DROME]	76.79	44.55	1	12	12	28	2.747E8	440	45.4	9.44
Q9W1G7	LD21576p OS=Drosophila melanogaster GN=Nap1 PE=2 SV=1 - [Q9W1G7_DROME]	76.36	29.19	1	11	11	37	8.630E8	370	42.7	4.79
Q8IP94	CG5353-PB, isoform B OS=Drosophila melanogaster GN=Aats-thr PE=3 SV=1 - [Q8IP94_DROME]	75.92	22.32	2	12	12	40	1.005E8	690	79.3	6.90
Q7KN75	Dodeca-satellite-binding protein 1, isoform A OS=Drosophila melanogaster GN=Dp1 PE=2 SV=1 - [Q7KN75_DROME]	74.95	24.75	1	23	23	40	1.724E8	1301	144.2	6.20
P15215	Laminin subunit gamma-1 OS=Drosophila melanogaster GN=LanB2 PE=2 SV=2 - [LAMC1_DROME]	73.86	26.78	1	29	29	44	2.402E8	1639	182.2	5.38
Q9VN44	FI07923p OS=Drosophila melanogaster GN=Karybeta3 PE=2 SV=1 - [Q9VN44_DROME]	73.04	21.18	1	17	17	45	3.307E8	1105	123.5	4.73
P29845	Heat shock 70 kDa protein cognate 5 OS=Drosophila melanogaster GN=Hsc70-5 PE=1 SV=2 - [HSP7E_DROME]	72.88	38.48	1	24	25	39	2.807E8	686	74.0	6.35
Q59E58	Zipper, isoform C OS=Drosophila melanogaster GN=zip PE=4 SV=1 - [Q59E58_DROME]	72.73	15.27	2	1	24	41	1.044E8	1971	226.8	5.47
Q9GU68	Eukaryotic translation initiation factor 5A OS=Drosophila melanogaster GN=eIF-5A PE=2 SV=2 - [IF5A_DROME]	72.37	51.57	1	12	12	47	1.990E9	159	17.6	5.15
P08879	Nucleoside diphosphate kinase OS=Drosophila melanogaster GN=awd PE=1 SV=3 - [NDKA_DROME]	71.70	44.44	1	6	6	32	1.403E9	153	17.2	8.12
Q9VHR8	Dipeptidyl peptidase 3 OS=Drosophila melanogaster GN=DppIII PE=2 SV=2 - [DPP3_DROME]	70.92	33.08	1	22	22	47	3.114E8	786	89.1	6.43
Q9VBU7	Nup358 OS=Drosophila melanogaster GN=Nup358 PE=4 SV=2 - [Q9VBU7_DROME]	70.27	15.36	1	26	26	34	1.776E8	2695	296.2	6.09
Q99323	Myosin heavy chain, non-muscle OS=Drosophila melanogaster GN=zip PE=1 SV=2 - [MYSN_DROME]	70.26	14.44	1	1	24	39	1.044E8	2057	236.5	5.68
P29327	40S ribosomal protein S6 OS=Drosophila melanogaster GN=RpS6 PE=1 SV=1 - [RS6_DROME]	70.18	34.68	2	11	11	36	1.291E9	248	28.4	10.74
Q94920	Voltage-dependent anion-selective channel OS=Drosophila melanogaster GN=porin PE=1 SV=3 - [VDAC_DROME]	69.37	47.87	1	12	12	34	1.018E9	282	30.5	6.96
P48149	40S ribosomal protein S15Aa OS=Drosophila melanogaster GN=RpS15Aa PE=1 SV=2 - [RS15A_DROME]	69.04	45.38	2	7	7	37	9.556E8	130	14.8	9.80
Q9VQ61	Aspartate aminotransferase OS=Drosophila melanogaster GN=Got2 PE=3 SV=1 - [Q9VQ61_DROME]	68.74	27.12	2	10	10	29	3.053E8	424	47.2	8.78
Q9VM69	CG10206-PA OS=Drosophila melanogaster GN=nop5 PE=2 SV=2 - [Q9VM69_DROME]	68.18	33.86	1	13	13	33	6.835E7	511	57.1	8.59
Q3YMU0	Protein disulfide-isomerase (Fragment) OS=Drosophila melanogaster GN=ERp60 PE=2 SV=1 - [Q3YMU0_DROME]	67.64	34.36	1	15	15	39	7.557E8	489	55.3	5.87
P12613	T-complex protein 1 subunit alpha OS=Drosophila melanogaster GN=T-cp1 PE=2 SV=2 - [TCPA_DROME]	67.61	31.24	1	15	15	41	5.611E8	557	59.5	6.39
Q9VMV5	Viking, isoform A OS=Drosophila melanogaster GN=vkg PE=4 SV=1 - [Q9VMV5_DROME]	67.43	11.29	1	17	17	31	1.470E8	1940	193.7	6.43
Q01604	Phosphoglycerate kinase OS=Drosophila melanogaster GN=Pgk PE=2 SV=2 - [PGK_DROME]	65.99	41.93	1	13	13	35	1.793E8	415	43.8	7.42
Q9VW54	26S proteasome regulatory complex subunit p97 OS=Drosophila melanogaster GN=Rpn1 PE=2 SV=2 - [Q9VW54_DROME]	65.87	19.80	1	16	16	34	1.891E8	919	102.2	5.85
B7YZQ7	Nucleosome remodeling factor-38kD, isoform B OS=Drosophila melanogaster GN=Nurf-38 PE=4 SV=1 - [B7YZQ7_DROME]	65.70	68.62	3	16	16	39	2.760E8	290	32.6	5.68
Q9VL78	FK506-binding protein 59 OS=Drosophila melanogaster GN=FKBP59 PE=1 SV=1 - [FKB59_DROME]	65.64	43.28	1	19	19	39	4.250E8	439	48.8	5.41
Q9VWV6	Transferrin OS=Drosophila melanogaster GN=Tsf1 PE=3 SV=1 - [Q9VWV6_DROME]	64.18	40.56	1	19	19	37	3.159E8	641	71.8	7.06
Q27415	Nucleoplasmin-like protein OS=Drosophila melanogaster GN=Nlp PE=1 SV=1 - [NLP_DROME]	63.96	36.18	1	5	5	30	7.930E8	152	17.0	4.61
Q8IPE8	CG4389-PB, isoform B OS=Drosophila melanogaster GN=Mtpalpha PE=3 SV=1 - [Q8IPE8_DROME]	63.76	25.54	4	15	15	36	2.552E8	744	79.6	8.85
Q9XTL9	Glycogen phosphorylase OS=Drosophila melanogaster GN=GlyP PE=2 SV=2 - [PYG_DROME]	63.59	22.99	1	17	17	36	1.137E8	844	96.9	6.52
P41042	40S ribosomal protein S4 OS=Drosophila melanogaster GN=RpS4 PE=1 SV=2 - [RS4_DROME]	63.31	51.72	1	14	14	56	1.531E9	261	29.1	10.18
Q9VLC5	Aldehyde dehydrogenase OS=Drosophila melanogaster GN=Aldh PE=3 SV=1 - [Q9VLC5_DROME]	62.75	29.81	1	12	12	33	2.349E8	520	57.0	6.80
P22977	Chorion protein S16 OS=Drosophila melanogaster GN=Cp16 PE=2 SV=2 - [CH16_DROME]	62.65	44.20	1	6	6	28	3.261E8	138	14.3	7.81
P29843	Heat shock 70 kDa protein cognate 1 OS=Drosophila melanogaster GN=Hsc70-1 PE=1 SV=1 - [HSP7A_DROME]	62.17	14.98	3	1	7	30	1.626E9	641	70.6	5.49
Q95029	Cathepsin L OS=Drosophila melanogaster GN=Cp1 PE=2 SV=2 - [CATL_DROME]	62.10	35.85	1	12	12	34	3.090E8	371	41.6	7.21
Q7JR58	CG6543, isoform A OS=Drosophila melanogaster GN=CG6543 PE=2 SV=1 - [Q7JR58_DROME]	61.72	41.36	1	9	9	30	1.929E8	295	31.6	8.63
A1ZAX1	Eukaryotic translation initiation factor 3 subunit C OS=Drosophila melanogaster GN=eIF3-S8 PE=1 SV=1 - [EIF3C_DROME]	61.57	21.43	1	19	19	40	1.324E8	910	105.6	6.06
Q9VAY2	Glycoprotein 93 OS=Drosophila melanogaster GN=Gp93 PE=2 SV=1 - [Q9VAY2_DROME]	61.29	19.57	1	11	13	29	1.754E8	787	90.2	5.02
P29310	14-3-3 protein zeta OS=Drosophila melanogaster GN=14-3-3zeta PE=1 SV=1 - [1433Z_DROME]	61.08	57.66	1	10	12	33	6.428E8	248	28.2	4.88
O44081	H/ACA ribonucleoprotein complex subunit 4 OS=Drosophila melanogaster GN=Nop60B PE=1 SV=1 - [DKC1_DROME]	60.63	30.12	1	14	14	29	2.994E8	508	56.8	9.28
Q9VAC4	CG7911 OS=Drosophila melanogaster GN=CG7911 PE=4 SV=1 - [Q9VAC4_DROME]	60.40	49.36	1	5	5	22	7.632E8	156	17.4	4.56
P02518	Heat shock protein 27 OS=Drosophila melanogaster GN=Hsp27 PE=1 SV=2 - [HSP27_DROME]	60.28	46.01	1	9	9	27	7.528E8	213	23.6	7.44
P41093	60S ribosomal protein L18a OS=Drosophila melanogaster GN=RpL18A PE=1 SV=1 - [RL18A_DROME]	60.15	44.07	1	11	11	48	2.454E9	177	21.0	10.62

Accession	Description	Score	Coverage	# Proteins	# Unique Peptides	# Peptides	# PSMs	Area	# AAs	MW [kDa]	calc. pI
Q86BM5	A kinase anchor protein 200, isoform G OS=Drosophila melanogaster GN=Akap200 PE=4 SV=1 - [Q86BM5_DROME]	59.99	19.35	3	13	13	23	2.337E8	837	88.1	4.22
Q9VK60	CG6180 OS=Drosophila melanogaster GN=CG6180 PE=2 SV=2 - [Q9VK60_DROME]	59.41	30.35	1	5	5	20	3.527E8	257	28.7	8.82
Q9VJ19	CG10652-PA, isoform A OS=Drosophila melanogaster GN=RpL30 PE=1 SV=1 - [Q9VJ19_DROME]	59.10	66.67	1	7	7	21	8.778E8	111	12.2	9.58
O18404	3-hydroxyacyl-CoA dehydrogenase type-2 OS=Drosophila melanogaster GN=scu PE=1 SV=1 - [HCD2_DROME]	58.84	65.10	2	12	12	30	1.978E8	255	26.9	9.03
A8JNP2	Arginine kinase, isoform E OS=Drosophila melanogaster GN=Argk PE=3 SV=1 - [A8JNP2_DROME]	57.86	36.53	2	12	12	34	2.448E8	375	42.2	6.32
Q9W5R8	60S ribosomal protein L5 OS=Drosophila melanogaster GN=RpL5 PE=1 SV=2 - [RL5_DROME]	57.50	29.43	1	10	10	32	2.382E9	299	34.0	9.77
P52295	Importin subunit alpha OS=Drosophila melanogaster GN=Pen PE=1 SV=2 - [IMA_DROME]	57.34	34.48	1	11	11	24	3.548E8	522	57.8	5.35
P11046	Laminin subunit beta-1 OS=Drosophila melanogaster GN=LanB1 PE=1 SV=4 - [LAMB1_DROME]	57.15	13.03	1	14	14	24	1.446E8	1788	198.2	5.33
Q9V3U6	26-29kD-proteinase OS=Drosophila melanogaster GN=26-29-p PE=2 SV=1 - [Q9V3U6_DROME]	57.04	21.68	1	11	11	28	8.716E8	549	62.1	6.74
P39018	40S ribosomal protein S19a OS=Drosophila melanogaster GN=RpS19a PE=1 SV=3 - [RS19A_DROME]	56.82	62.18	1	13	13	31	2.165E9	156	17.3	10.11
Q9V6K1	Tripeptidyl-peptidase 2 OS=Drosophila melanogaster GN=TppII PE=1 SV=2 - [TPP2_DROME]	56.40	17.42	1	21	21	37	1.790E8	1441	158.6	7.59
Q8MST5	Beta-Tubulin at 97EF, isoform B OS=Drosophila melanogaster GN=betaTub97EF PE=2 SV=1 - [Q8MST5_DROME]	56.37	12.04	2	1	6	27	3.933E8	457	51.2	4.86
Q9I7D3	Caprin homolog OS=Drosophila melanogaster GN=Capr PE=1 SV=1 - [CAPR1_DROME]	56.24	24.66	1	16	16	29	2.425E8	961	103.5	6.51
Q9VVU1	CG3902-PA OS=Drosophila melanogaster GN=CG3902-RA PE=2 SV=1 - [Q9VVU1_DROME]	56.13	27.54	1	9	9	30	2.052E8	414	45.3	6.71
P08928	Lamin Dm0 OS=Drosophila melanogaster GN=Lam PE=1 SV=4 - [LAM0_DROME]	55.85	37.78	1	16	16	25	9.324E7	622	71.3	6.47
Q9W0A8	FI01658p OS=Drosophila melanogaster GN=RpL23A PE=1 SV=1 - [Q9W0A8_DROME]	55.39	31.05	1	10	10	34	2.118E9	277	29.4	10.95
P54397	39 kDa FK506-binding nuclear protein OS=Drosophila melanogaster GN=FK506-bp1 PE=1 SV=2 - [FKB39_DROME]	54.86	26.61	1	11	11	35	5.196E8	357	39.3	4.79
Q0KHS7	Lipid storage droplets surface-binding protein OS=Drosophila melanogaster GN=Lsd-2 PE=3 SV=1 - [Q0KHS7_DROME]	53.50	50.15	2	1	13	26	2.904E8	335	36.3	8.79
P41094	40S ribosomal protein S18 OS=Drosophila melanogaster GN=RpS18 PE=1 SV=1 - [RS18_DROME]	53.40	61.84	1	14	14	27	1.303E9	152	17.6	10.48
Q9VJ86	Bicoid stability factor OS=Drosophila melanogaster GN=bsf PE=2 SV=2 - [Q9VJ86_DROME]	53.06	15.79	1	19	20	26	9.390E7	1412	157.2	7.14
P02255	Histone H1 OS=Drosophila melanogaster GN=His1 PE=1 SV=1 - [H1_DROME]	53.03	32.42	5	10	10	21	7.980E8	256	26.3	10.54
P22700	Calcium-transporting ATPase sarcoplasmic/endoplasmic reticulum type OS=Drosophila melanogaster GN=Ca-P60A PE=1 SV=2 - [ATC1_DROME]	52.97	15.78	1	12	12	24	8.185E7	1020	111.6	5.41
Q9VEH0	Aluminum tubes, isoform A OS=Drosophila melanogaster GN=alt PE=4 SV=1 - [Q9VEH0_DROME]	52.83	24.58	2	14	14	23	1.554E8	842	95.0	7.09
P48809	Heterogeneous nuclear ribonucleoprotein 27C OS=Drosophila melanogaster GN=Hrb27C PE=1 SV=2 - [RB27C_DROME]	52.72	26.84	1	8	8	20	1.977E8	421	44.7	6.80
A8JUV4	Vitelline membrane-like OS=Drosophila melanogaster GN=Vml PE=4 SV=1 - [A8JUV4_DROME]	52.60	16.61	1	5	5	37	5.395E8	578	56.1	7.93
Q9VC18	CG11089 OS=Drosophila melanogaster GN=CG11089 PE=2 SV=1 - [Q9VC18_DROME]	52.24	30.85	1	14	14	27	1.581E8	590	63.3	7.87
Q0E8E8	MIP08013p1 OS=Drosophila melanogaster GN=Mpcp PE=2 SV=1 - [Q0E8E8_DROME]	51.65	32.30	2	10	10	32	3.887E8	356	38.8	8.88
P29742	Clathrin heavy chain OS=Drosophila melanogaster GN=Chc PE=1 SV=1 - [CLH_DROME]	51.55	13.35	1	18	18	33	1.537E8	1678	191.1	5.72
E1JJ68	Rm62, isoform H OS=Drosophila melanogaster GN=Rm62 PE=3 SV=1 - [E1JJ68_DROME]	50.82	44.17	2	16	19	38	1.954E8	575	62.4	9.57
Q24208	Eukaryotic translation initiation factor 2 subunit 3 OS=Drosophila melanogaster GN=eIF-2gamma PE=2 SV=1 - [IF2G_DROME]	50.81	33.47	2	14	14	25	2.639E8	475	51.5	8.73
Q9VZS5	60S ribosomal protein L28 OS=Drosophila melanogaster GN=RpL28 PE=1 SV=1 - [RL28_DROME]	50.59	56.25	1	10	10	26	7.826E9	144	16.0	11.09
P61851	Superoxide dismutase [Cu-Zn] OS=Drosophila melanogaster GN=Sod PE=1 SV=2 - [SODC_DROME]	50.58	54.90	2	6	6	20	4.312E8	153	15.7	6.11
Q9W1V3	rRNA 2'-O-methyltransferase fibrillarin OS=Drosophila melanogaster GN=Fib PE=2 SV=1 - [FBRL_DROME]	49.52	38.37	1	12	12	32	2.681E8	344	34.6	10.29
Q0E9B6	40S ribosomal protein S11 OS=Drosophila melanogaster GN=RpS11 PE=1 SV=1 - [RS11_DROME]	49.26	46.45	2	8	8	24	1.379E9	155	18.1	10.93
Q9VXY7	Lipid storage droplets surface-binding protein 2 OS=Drosophila melanogaster GN=Lsd-2 PE=1 SV=1 - [LSD2_DROME]	49.23	52.56	3	2	14	28	2.904E8	352	38.2	8.41
O61231	60S ribosomal protein L10 OS=Drosophila melanogaster GN=RpL10 PE=1 SV=1 - [RL10_DROME]	49.00	45.87	1	13	13	32	1.324E9	218	25.5	9.85
O97428	CG4944-PA, isoform A OS=Drosophila melanogaster GN=cib PE=1 SV=1 - [O97428_DROME]	48.80	48.84	2	5	5	18	1.919E8	129	14.4	5.21
Q9VKX2	Malate dehydrogenase OS=Drosophila melanogaster GN=Mdh1 PE=2 SV=2 - [Q9VKX2_DROME]	48.67	37.39	1	11	11	28	5.214E8	337	36.0	7.39
P17704	40S ribosomal protein S17 OS=Drosophila melanogaster GN=RpS17 PE=1 SV=2 - [RS17_DROME]	48.67	47.33	1	6	6	22	8.419E8	131	15.3	9.94
P55841	60S ribosomal protein L14 OS=Drosophila melanogaster GN=RpL14 PE=1 SV=1 - [RL14_DROME]	48.32	40.96	1	8	8	21	1.469E9	166	19.2	11.18
O02195	Eukaryotic translation initiation factor 3 subunit I OS=Drosophila melanogaster GN=Trip1 PE=1 SV=1 - [EIF3I_DROME]	48.09	40.80	1	8	8	22	2.830E8	326	36.1	5.34
E2QCF1	ATP citrate lyase, isoform F OS=Drosophila melanogaster GN=ATPCL PE=4 SV=1 - [E2QCF1_DROME]	47.78	11.05	2	9	9	22	5.649E7	1095	119.7	7.12
E1JH02	Lingerer, isoform E OS=Drosophila melanogaster GN=lig PE=4 SV=1 - [E1JH02_DROME]	47.52	17.87	2	14	14	32	1.562E8	1332	134.5	6.92
Q9W029	CG5717-PA OS=Drosophila melanogaster GN=yellow-g PE=2 SV=1 - [Q9W029_DROME]	46.15	31.30	1	11	11	20	2.518E8	393	43.0	7.80
Q9V3G1	60S ribosomal protein L8 OS=Drosophila melanogaster GN=RpL8 PE=1 SV=1 - [RL8_DROME]	45.98	46.88	1	11	11	25	1.779E9	256	27.9	11.15
Q9VK69	T-complex protein 1 subunit delta OS=Drosophila melanogaster GN=CG5525 PE=2 SV=1 - [Q9VK69_DROME]	45.56	46.72	1	18	19	33	4.374E8	533	57.1	7.56
A1ZAB5	Protein clueless OS=Drosophila melanogaster GN=clu PE=1 SV=1 - [CLU_DROME]	45.33	15.40	1	17	17	23	7.350E7	1448	160.8	6.60
Q961R8	GH09263p OS=Drosophila melanogaster GN=Aats-gly PE=2 SV=1 - [Q961R8_DROME]	45.28	20.32	3	11	11	24	1.333E8	679	75.8	6.40
Q9VXQ5	GH13725p OS=Drosophila melanogaster GN=Tcp-1zeta PE=2 SV=1 - [Q9VXQ5_DROME]	45.13	30.77	1	14	14	32	2.487E8	533	58.2	6.62
Q94511	NADH-ubiquinone oxidoreductase 75 kDa subunit, mitochondrial OS=Drosophila melanogaster GN=ND75 PE=2 SV=3 - [NDUS1_DROME]	44.80	21.20	1	12	12	19	1.017E8	731	78.6	6.84

Accession	Description	Score	Coverage	# Proteins	# Unique Peptides	# Peptides	# PSMs	Area	# AAs	MW [kDa]	calc. pI
Q9W028	IP19120p OS=Drosophila melanogaster GN=yellow-g2 PE=2 SV=1 - [Q9W028_DROME]	44.55	30.63	1	8	8	21	1.410E8	382	42.5	9.11
Q9V5C6	Proteasome subunit alpha type-3 OS=Drosophila melanogaster GN=Prosalpha7 PE=1 SV=1 - [PSA3_DROME]	44.47	28.85	7	5	6	23	1.826E8	253	27.7	5.69
Q9VAW5	La-related protein 1 OS=Drosophila melanogaster GN=larp PE=1 SV=5 - [LARP_DROME]	44.37	16.92	1	17	17	24	8.740E7	1673	178.0	9.60
P11449	Vitelline membrane protein Vm26Aa OS=Drosophila melanogaster GN=Vm26Aa PE=2 SV=1 - [VTU1_DROME]	44.05	16.31	1	2	2	26	2.952E9	141	14.3	7.75
Q9VNE9	60S ribosomal protein L13a OS=Drosophila melanogaster GN=RpL13A PE=1 SV=1 - [RL13A_DROME]	43.94	33.17	1	9	9	28	3.592E8	205	23.6	11.03
P41374	Eukaryotic translation initiation factor 2 subunit 1 OS=Drosophila melanogaster GN=eIF-2alpha PE=2 SV=1 - [IF2A_DROME]	43.87	42.23	1	11	11	27	1.278E8	341	38.6	4.94
Q7K2G1	Proteasomal ubiquitin receptor ADRM1 homolog OS=Drosophila melanogaster GN=Rpn13 PE=1 SV=1 - [ADRM1_DROME]	43.82	33.16	1	8	8	17	7.140E7	389	42.0	5.64
Q9VCK0	Eukaryotic translation initiation factor 3 subunit D-1 OS=Drosophila melanogaster GN=eIF-3p66 PE=1 SV=1 - [EI3D1_DROME]	43.74	20.89	1	8	8	20	1.685E8	560	63.8	7.02
Q9VZ23	GTP-binding nuclear protein Ran OS=Drosophila melanogaster GN=ran PE=1 SV=1 - [RAN_DROME]	43.73	48.15	1	11	11	30	4.769E8	216	24.7	7.81
Q9VVA4	CG9674, isoform A OS=Drosophila melanogaster GN=CG9674 PE=2 SV=2 - [Q9VVA4_DROME]	43.46	11.64	3	16	16	26	8.201E7	2114	231.9	6.42
P46415	Alcohol dehydrogenase class-3 OS=Drosophila melanogaster GN=Fdh PE=1 SV=3 - [ADHX_DROME]	43.34	21.37	1	6	6	22	1.542E8	379	40.4	6.71
P06606	Tubulin alpha-4 chain OS=Drosophila melanogaster GN=alphaTub67C PE=3 SV=1 - [TBA4_DROME]	43.31	30.52	1	10	10	23	1.419E8	462	51.1	5.26
Q8T8R1	CCHC-type zinc finger protein CG3800 OS=Drosophila melanogaster GN=CG3800 PE=1 SV=1 - [Y3800_DROME]	43.21	53.33	1	8	8	16	9.602E8	165	17.6	8.65
P12881	Proteasome subunit alpha type-1 OS=Drosophila melanogaster GN=Prosalpha6 PE=1 SV=1 - [PSA1_DROME]	43.06	41.94	1	9	9	22	3.814E8	279	31.0	6.55
Q9W227	Peptidyl-prolyl cis-trans isomerase OS=Drosophila melanogaster GN=CG2852 PE=2 SV=1 - [Q9W227_DROME]	42.41	57.07	1	13	13	31	5.491E8	205	22.2	8.75
Q9V9W2	Ribosomal protein L6, isoform A OS=Drosophila melanogaster GN=RpL6 PE=1 SV=1 - [Q9V9W2_DROME]	42.05	29.63	2	12	12	24	1.091E9	243	27.7	10.68
P48375	12 kDa FK506-binding protein OS=Drosophila melanogaster GN=FK506-bp2 PE=3 SV=2 - [FKB12_DROME]	41.94	41.67	1	4	4	15	5.469E8	108	11.7	8.13
Q9VLM8	Alanine--tRNA ligase, cytoplasmic OS=Drosophila melanogaster GN=Aats-ala PE=2 SV=1 - [SYAC_DROME]	41.91	22.05	1	17	17	28	1.119E8	966	107.7	6.13
Q8MZI3	CG10077, isoform D OS=Drosophila melanogaster GN=CG10077 PE=2 SV=1 - [Q8MZI3_DROME]	41.73	11.74	2	4	7	19	7.545E7	818	88.2	9.36
Q9VRP2	CG10576, isoform A OS=Drosophila melanogaster GN=CG10576 PE=4 SV=1 - [Q9VRP2_DROME]	41.66	31.20	1	11	11	23	6.504E8	391	42.7	7.11
Q9VKI8	CG6287-PA OS=Drosophila melanogaster GN=CG6287 PE=2 SV=1 - [Q9VKI8_DROME]	41.24	46.39	1	12	12	33	4.454E8	332	35.2	7.36
P07184	Chorion protein S18 OS=Drosophila melanogaster GN=Cp18 PE=2 SV=2 - [CH18_DROME]	41.09	44.19	1	6	6	19	3.355E8	172	17.2	9.32
P25843	Profilin OS=Drosophila melanogaster GN=chic PE=1 SV=1 - [PROF_DROME]	41.08	59.52	1	4	4	15	1.187E8	126	13.7	5.41
P54385	Glutamate dehydrogenase, mitochondrial OS=Drosophila melanogaster GN=Gdh PE=1 SV=2 - [DHE3_DROME]	40.42	23.49	2	12	12	25	1.847E8	562	62.5	8.27
Q24407	ATP synthase-coupling factor 6, mitochondrial OS=Drosophila melanogaster GN=ATPsyn-Cf6 PE=3 SV=1 - [ATP5J_DROME]	40.31	30.19	1	3	3	17	1.589E8	106	11.9	9.04
Q9VVL7	Dihydrolipoyl dehydrogenase OS=Drosophila melanogaster GN=CG7430 PE=2 SV=1 - [Q9VVL7_DROME]	40.11	21.03	1	9	9	21	2.798E8	504	53.1	6.87
O01666	ATP synthase subunit gamma, mitochondrial OS=Drosophila melanogaster GN=ATPsyn-gamma PE=2 SV=2 - [ATPG_DROME]	40.03	35.69	1	9	9	20	3.231E8	297	32.9	9.22
E1JIR4	Na pump alpha subunit, isoform I OS=Drosophila melanogaster GN=Atpalpha PE=3 SV=1 - [E1JIR4_DROME]	39.88	23.85	3	18	18	26	1.303E8	1002	111.1	5.49
Q9VQL1	CG17259, isoform A OS=Drosophila melanogaster GN=CG17259 PE=2 SV=1 - [Q9VQL1_DROME]	39.68	32.34	1	11	11	21	3.054E8	501	56.4	6.49
Q9VJY6	60S ribosomal protein L24 OS=Drosophila melanogaster GN=RpL24 PE=1 SV=1 - [RL24_DROME]	39.64	31.61	1	7	7	21	1.300E8	155	17.5	11.06
Q9VWH4	Probable isocitrate dehydrogenase [NAD] subunit alpha, mitochondrial OS=Drosophila melanogaster GN=l(1)G0156 PE=2 SV=1 - [IDH3A_DROME]	39.64	31.03	2	12	12	28	1.697E8	377	40.8	7.36
P08120	Collagen alpha-1(IV) chain OS=Drosophila melanogaster GN=Cg25C PE=2 SV=3 - [CO4A1_DROME]	39.47	3.88	1	5	5	18	1.802E8	1779	174.2	8.60
M9PD08	Ef1alpha-like factor, isoform C OS=Drosophila melanogaster GN=Elf PE=4 SV=1 - [M9PD08_DROME]	39.44	25.45	2	11	11	24	1.108E8	495	55.3	7.20
A1Z7H3	Lethal (2) 44DEa, isoform C OS=Drosophila melanogaster GN=Acsl PE=4 SV=1 - [A1Z7H3_DROME]	39.12	20.79	3	10	10	20	5.376E7	707	79.4	8.44
A1Z6L9	FI21274p1 OS=Drosophila melanogaster GN=Trap1 PE=2 SV=1 - [A1Z6L9_DROME]	38.56	16.93	1	8	9	20	4.563E8	691	77.9	7.59
Q9VAN7	GH13304p OS=Drosophila melanogaster GN=Pglym78 PE=2 SV=2 - [Q9VAN7_DROME]	38.52	38.43	2	7	7	19	1.620E8	255	28.6	6.89
P09491	Tropomyosin-2 OS=Drosophila melanogaster GN=Tm2 PE=2 SV=1 - [TPM2_DROME]	38.49	34.86	1	8	10	16	2.455E8	284	33.0	4.83
P40304	Proteasome subunit beta type-1 OS=Drosophila melanogaster GN=Prosbeta6 PE=2 SV=2 - [PSB1_DROME]	38.17	34.04	1	6	6	28	3.720E8	235	25.8	6.54
Q0E993	Valyl-tRNA synthetase, isoform A OS=Drosophila melanogaster GN=Aats-val PE=3 SV=1 - [Q0E993_DROME]	38.16	15.25	1	14	14	21	1.041E8	1049	118.2	6.65
Q27331	V-type proton ATPase catalytic subunit A isoform 2 OS=Drosophila melanogaster GN=Vha68-2 PE=1 SV=2 - [VATA2_DROME]	38.12	17.10	2	3	8	19	7.456E7	614	68.3	5.34
Q8T3U2	40S ribosomal protein S23 OS=Drosophila melanogaster GN=RpS23 PE=1 SV=1 - [RS23_DROME]	37.96	48.25	1	6	6	20	7.268E8	143	16.0	10.59
Q7KLV9	26S proteasome non-ATPase regulatory subunit 11 OS=Drosophila melanogaster GN=Rpn6 PE=1 SV=1 - [PSD11_DROME]	37.82	31.04	1	10	10	25	2.480E8	422	47.2	5.88
P48588	40S ribosomal protein S25 OS=Drosophila melanogaster GN=RpS25 PE=1 SV=3 - [RS25_DROME]	37.75	34.19	1	5	5	23	3.184E9	117	13.2	10.27
P45594	Cofilin/actin-depolymerizing factor homolog OS=Drosophila melanogaster GN=tsr PE=2 SV=1 - [CADF_DROME]	37.70	41.89	1	6	6	25	9.034E8	148	17.1	7.17
P29613	Triosephosphate isomerase OS=Drosophila melanogaster GN=Tpi PE=1 SV=3 - [TPIS_DROME]	37.60	38.87	1	8	8	25	4.492E8	247	26.6	6.00
Q7K0E6	Aspartyl-tRNA synthetase OS=Drosophila melanogaster GN=Aats-asp PE=2 SV=1 - [Q7K0E6_DROME]	37.57	30.51	1	10	10	22	1.616E8	531	59.0	6.81
P05990	CAD protein OS=Drosophila melanogaster GN=r PE=1 SV=3 - [PYR1_DROME]	37.53	7.82	1	15	15	23	1.329E8	2224	246.5	6.64
Q27580	Adenosylhomocysteinase OS=Drosophila melanogaster GN=Ahcy13 PE=2 SV=2 - [SAHH_DROME]	37.36	45.14	2	14	14	28	3.695E8	432	47.3	6.20
X2JE34	RNA-binding protein 2, isoform E OS=Drosophila melanogaster GN=Rbp2 PE=4 SV=1 - [X2JE34_DROME]	37.32	38.17	5	8	8	17	1.298E8	317	34.2	9.29
P31409	V-type proton ATPase subunit B OS=Drosophila melanogaster GN=Vha55 PE=2 SV=1 - [VATB_DROME]	37.30	25.31	1	11	11	19	2.164E8	490	54.5	5.40

Accession	Description	Score	Coverage	# Proteins	# Unique Peptides	# Peptides	# PSMs	Area	# AAs	MW [kDa]	calc. pI
P08985	Histone H2A.v OS=Drosophila melanogaster GN=His2Av PE=1 SV=2 - [H2AV_DROME]	37.19	29.08	1	4	6	16	6.799E8	141	15.0	10.24
Q7K1Q6	CG42351, isoform B OS=Drosophila melanogaster GN=Jabba PE=2 SV=1 - [Q7K1Q6_DROME]	37.12	30.94	4	8	8	15	3.361E8	320	35.9	5.29
Q9VWD9	LD38919p OS=Drosophila melanogaster GN=Ubqn PE=1 SV=1 - [Q9VWD9_DROME]	37.00	22.67	1	8	8	14	2.227E7	547	58.8	5.11
Q8MT58	CG17337 OS=Drosophila melanogaster GN=CG17337 PE=2 SV=1 - [Q8MT58_DROME]	36.98	23.01	1	9	9	21	1.899E8	478	53.1	5.66
M9PFW8	CG32165, isoform B OS=Drosophila melanogaster GN=CG32165 PE=4 SV=1 - [M9PFW8_DROME]	36.78	7.84	4	7	7	22	8.981E7	1059	118.8	4.81
Q7KMQ0	26S proteasome regulatory complex subunit p48B OS=Drosophila melanogaster GN=Rpt1 PE=2 SV=1 - [Q7KMQ0_DROME]	36.78	30.02	1	11	11	27	9.451E7	433	48.5	6.04
Q9VKW5	CG5355 OS=Drosophila melanogaster GN=CG5355 PE=2 SV=2 - [Q9VKW5_DROME]	36.77	19.58	2	12	12	19	1.594E8	756	86.3	6.19
Q9VMU4	60S ribosomal protein L37a OS=Drosophila melanogaster GN=RpL37A PE=1 SV=3 - [RL37A_DROME]	36.72	55.43	1	6	6	19	3.777E8	92	10.3	10.61
Q9VGM2	CG6783, isoform B OS=Drosophila melanogaster GN=fabp PE=2 SV=1 - [Q9VGM2_DROME]	36.62	63.08	3	9	9	17	8.621E8	130	14.5	5.66
Q9VSA3	Probable medium-chain specific acyl-CoA dehydrogenase, mitochondrial OS=Drosophila melanogaster GN=CG12262 PE=1 SV=1 - [ACADM_DROME]	36.61	32.70	1	10	10	20	1.428E8	419	45.8	7.94
Q94518	Nascent polypeptide-associated complex subunit alpha OS=Drosophila melanogaster GN=Nacalpha PE=1 SV=2 - [NACA_DROME]	36.23	41.94	1	5	5	14	3.199E8	217	23.0	4.74
P46222	60S ribosomal protein L11 OS=Drosophila melanogaster GN=RpL11 PE=1 SV=2 - [RL11_DROME]	36.19	29.35	1	6	6	16	9.558E8	184	21.1	10.02
Q7KUB0	Isocitrate dehydrogenase [NADP] OS=Drosophila melanogaster GN=Idh PE=3 SV=1 - [Q7KUB0_DROME]	36.13	33.41	5	12	12	31	3.424E8	416	46.6	6.74
Q9VA91	40S ribosomal protein S7 OS=Drosophila melanogaster GN=RpS7 PE=1 SV=1 - [RS7_DROME]	36.09	43.81	2	10	10	25	1.920E9	194	22.2	9.80
A1ZBB4	CG30122, isoform B OS=Drosophila melanogaster GN=CG30122 PE=4 SV=1 - [A1ZBB4_DROME]	36.07	14.00	4	9	9	14	3.289E7	1271	140.4	5.01
Q9I7K6	Protein NASP homolog OS=Drosophila melanogaster GN=CG8223 PE=1 SV=1 - [NASP_DROME]	35.96	32.52	1	7	7	16	1.849E8	492	51.9	4.39
Q9VNX6	LD06749p OS=Drosophila melanogaster GN=Nopp140 PE=2 SV=3 - [Q9VNX6_DROME]	35.55	9.17	4	7	7	13	5.041E7	720	72.2	9.04
Q9VFQ9	Dipeptidase B, isoform A OS=Drosophila melanogaster GN=Dip-B PE=2 SV=2 - [Q9VFQ9_DROME]	35.21	19.49	1	7	7	15	8.675E7	508	55.5	6.76
O17445	60S ribosomal protein L15 OS=Drosophila melanogaster GN=RpL15 PE=1 SV=1 - [RL15_DROME]	35.13	38.73	1	9	9	19	1.274E9	204	24.3	11.47
Q9U3Z7	NHP2-like protein 1 homolog OS=Drosophila melanogaster GN=hoip PE=2 SV=1 - [NH2L1_DROME]	35.06	42.52	1	4	4	14	1.093E9	127	13.9	7.09
Q94516	ATP synthase subunit b, mitochondrial OS=Drosophila melanogaster GN=ATPsyn-b PE=2 SV=2 - [AT5F1_DROME]	34.68	20.99	1	5	5	16	1.549E8	243	27.3	8.72
Q7KW39	Probable methylmalonate-semialdehyde dehydrogenase [acylating], mitochondrial OS=Drosophila melanogaster GN=CG17896 PE=2 SV=1 - [MMSA_DROME]	34.51	24.81	1	10	10	16	1.601E8	520	55.9	8.35
Q9VIX7	CG15825-PB, isoform B OS=Drosophila melanogaster GN=fon PE=2 SV=1 - [Q9VIX7_DROME]	34.40	15.08	3	5	5	10	1.756E8	577	58.0	6.96
Q9VL18	Probable elongation factor 1-delta OS=Drosophila melanogaster GN=eEF1delta PE=1 SV=1 - [EF1D_DROME]	34.39	49.61	1	12	12	20	3.986E8	256	28.9	4.87
Q9VVU2	CG6846-PA OS=Drosophila melanogaster GN=RpL26 PE=1 SV=1 - [Q9VVU2_DROME]	34.33	53.69	2	10	10	17	1.070E9	149	17.3	10.95
Q9V8M5	Probable 3-hydroxyisobutyrate dehydrogenase, mitochondrial OS=Drosophila melanogaster GN=CG15093 PE=2 SV=2 - [3HIDH_DROME]	34.32	22.53	1	5	5	16	6.805E7	324	33.9	8.13
M9NFR5	Ypsilon schachtel, isoform B OS=Drosophila melanogaster GN=yps PE=4 SV=1 - [M9NFR5_DROME]	34.09	34.41	2	7	7	13	3.420E8	340	37.0	10.01
Q9VKM1	Protein piwi OS=Drosophila melanogaster GN=piwi PE=1 SV=1 - [PIWI_DROME]	33.62	17.32	1	13	13	17	1.699E8	843	97.1	9.57
Q9VSD6	D-Importin 7/RanBP7 OS=Drosophila melanogaster GN=msk PE=2 SV=1 - [Q9VSD6_DROME]	33.61	13.35	10	10	10	18	2.328E8	1049	119.2	4.86
B3LF78	Fmr1, isoform F OS=Drosophila melanogaster GN=Fmr1 PE=2 SV=1 - [B3LF78_DROME]	33.53	20.90	2	11	11	14	1.045E8	646	71.9	9.13
Q8IPB1	Deoxyuridine triphosphatase, isoform B OS=Drosophila melanogaster GN=dUTPase PE=2 SV=1 - [Q8IPB1_DROME]	33.20	43.10	2	7	7	15	4.329E8	174	18.5	5.43
Q9VT04	MIP04528p OS=Drosophila melanogaster GN=path PE=2 SV=1 - [Q9VT04_DROME]	33.04	10.83	2	3	3	13	4.782E7	471	52.0	7.71
Q08473	RNA-binding protein squid OS=Drosophila melanogaster GN=sqd PE=1 SV=3 - [SQD_DROME]	33.00	18.90	1	5	5	16	2.982E8	344	36.2	7.46
Q9W3M7	CG10777, isoform B OS=Drosophila melanogaster GN=CG10777 PE=2 SV=1 - [Q9W3M7_DROME]	32.95	15.03	1	7	9	16	2.995E7	945	100.4	9.14
Q9VHC7	FI21236p1 OS=Drosophila melanogaster GN=rump PE=2 SV=1 - [Q9VHC7_DROME]	32.93	9.97	1	4	4	12	7.924E7	632	66.7	7.85
M9NG39	Protein on ecdysone puffs, isoform D OS=Drosophila melanogaster GN=Pep PE=4 SV=1 - [M9NG39_DROME]	32.91	16.59	2	9	9	14	9.432E7	693	75.6	5.44
Q8IRQ5	LP02262p OS=Drosophila melanogaster GN=l(1)G0255 PE=2 SV=1 - [Q8IRQ5_DROME]	32.89	19.91	4	7	7	14	4.471E7	467	50.4	7.53
P29413	Calreticulin OS=Drosophila melanogaster GN=Crc PE=1 SV=2 - [CALR_DROME]	32.73	34.73	1	11	11	16	2.496E8	406	46.8	4.58
Q9XZ57	Eb1, isoform E OS=Drosophila melanogaster GN=Eb1 PE=2 SV=1 - [Q9XZ57_DROME]	32.43	31.72	3	6	6	16	1.289E8	290	32.5	5.34
Q9W0B8	Coatomer subunit alpha OS=Drosophila melanogaster GN=alphaCOP PE=2 SV=1 - [Q9W0B8_DROME]	32.39	7.86	1	6	6	16	3.791E7	1234	139.2	7.65
O18332	FI01544p OS=Drosophila melanogaster GN=Rab1 PE=2 SV=1 - [O18332_DROME]	32.25	38.54	22	5	7	16	1.054E8	205	22.7	5.47
Q9I7P8	CG4365, isoform B OS=Drosophila melanogaster GN=CG4365 PE=3 SV=1 - [Q9I7P8_DROME]	31.68	29.89	3	7	7	16	1.462E8	271	30.3	6.07
Q8SWR8	Ataxin-2 homolog OS=Drosophila melanogaster GN=Atx2 PE=1 SV=1 - [ATX2_DROME]	31.50	13.56	1	9	9	12	1.275E8	1084	117.5	8.91
Q9VNE2	Protein krasavietz OS=Drosophila melanogaster GN=kra PE=1 SV=1 - [PKRA_DROME]	31.44	16.59	1	6	6	12	6.858E7	422	49.2	5.74
Q9I7R0	CG18815, isoform A OS=Drosophila melanogaster GN=CG18815 PE=2 SV=1 - [Q9I7R0_DROME]	31.39	49.54	3	7	7	21	1.037E8	216	23.1	6.28
B7Z0X1	Serine hydroxymethyltransferase OS=Drosophila melanogaster GN=CG3011 PE=3 SV=1 - [B7Z0X1_DROME]	31.23	20.99	2	9	9	19	1.506E8	467	51.0	7.72
O97102	Small ubiquitin-related modifier OS=Drosophila melanogaster GN=smt3 PE=1 SV=1 - [O97102_DROME]	31.11	44.44	1	5	5	15	5.774E8	90	10.1	5.45
Q9VQR2	CG8844 protein OS=Drosophila melanogaster GN=Pdsw PE=2 SV=1 - [Q9VQR2_DROME]	31.05	25.79	1	3	3	9	3.567E7	159	18.9	7.01
Q9VHE5	RH48056p OS=Drosophila melanogaster GN=RpL34b PE=2 SV=1 - [Q9VHE5_DROME]	30.96	38.10	1	2	8	19	7.204E8	168	18.4	11.52
Q94522	Succinyl-CoA ligase [ADP/GDP-forming] subunit alpha, mitochondrial OS=Drosophila melanogaster GN=Scsalpha PE=2 SV=3 - [SUCA_DROME]	30.95	24.70	2	7	7	16	2.304E8	328	34.4	8.98

Accession	Description	Score	Coverage	# Proteins	# Unique Peptides	# Peptides	# PSMs	Area	# AAs	MW [kDa]	calc. pI
P91938	Thioredoxin reductase 1, mitochondrial OS=Drosophila melanogaster GN=Trxr-1 PE=1 SV=2 - [TRXR1_DROME]	30.92	16.95	2	9	9	21	4.125E8	596	64.3	7.91
F0JAN1	Ade5, isoform B OS=Drosophila melanogaster GN=ade5 PE=2 SV=1 - [F0JAN1_DROME]	30.78	14.25	2	5	5	13	8.281E7	428	47.1	8.38
Q9VGS2	Translationally-controlled tumor protein homolog OS=Drosophila melanogaster GN=Tctp PE=1 SV=1 - [TCTP_DROME]	30.73	53.49	1	8	8	19	2.765E8	172	19.6	4.81
Q9VGW7	Arginine methyltransferase 1 OS=Drosophila melanogaster GN=Art1 PE=2 SV=1 - [Q9VGW7_DROME]	30.61	25.00	1	7	7	12	1.741E8	376	42.8	5.15
Q9VPX7	Adenylyl cyclase-associated protein OS=Drosophila melanogaster GN=capt PE=3 SV=2 - [Q9VPX7_DROME]	30.59	29.95	2	9	9	15	1.475E8	424	45.6	7.06
Q7KN94	Walrus, isoform A OS=Drosophila melanogaster GN=wal PE=2 SV=1 - [Q7KN94_DROME]	30.42	27.88	1	5	5	13	5.389E7	330	34.2	8.32
P50882	60S ribosomal protein L9 OS=Drosophila melanogaster GN=RpL9 PE=1 SV=2 - [RL9_DROME]	30.39	54.21	1	10	10	22	4.690E8	190	21.4	9.69
Q9V3V6	26S proteasome regulatory complex subunit p50 OS=Drosophila melanogaster GN=Rpt5 PE=2 SV=1 - [Q9V3V6_DROME]	30.35	28.74	1	9	9	17	3.149E8	428	47.8	5.34
P84051	Histone H2A OS=Drosophila melanogaster GN=His2A PE=1 SV=2 - [H2A_DROME]	30.32	42.74	2	3	5	15	6.577E8	124	13.4	10.73
Q24186	40S ribosomal protein S5a OS=Drosophila melanogaster GN=RpS5a PE=1 SV=1 - [RS5A_DROME]	30.19	28.95	1	2	9	13	8.616E8	228	25.4	8.63
Q9VRL0	CG4769, isoform A OS=Drosophila melanogaster GN=CG4769 PE=2 SV=1 - [Q9VRL0_DROME]	30.14	27.36	1	7	7	22	1.971E8	307	33.7	8.53
Q9V434	Asparaginyl-tRNA synthetase, isoform A OS=Drosophila melanogaster GN=Aats-asn PE=2 SV=1 - [Q9V434_DROME]	30.14	13.44	1	7	7	17	2.022E7	558	63.9	5.96
Q9VNB9	RE17737p OS=Drosophila melanogaster GN=RpL35A PE=1 SV=1 - [Q9VNB9_DROME]	30.07	51.59	1	14	14	22	6.352E8	157	17.6	11.12
Q7KU82	MIP20544p OS=Drosophila melanogaster GN=smid PE=2 SV=2 - [Q7KU82_DROME]	29.79	11.21	4	6	6	13	3.527E7	910	100.2	5.27
P17210	Kinesin heavy chain OS=Drosophila melanogaster GN=Khc PE=1 SV=2 - [KINH_DROME]	29.54	11.38	1	9	9	12	9.697E7	975	110.3	5.77
P02283	Histone H2B OS=Drosophila melanogaster GN=His2B PE=1 SV=2 - [H2B_DROME]	29.52	45.53	1	6	6	24	1.426E9	123	13.7	10.35
Q9VQF7	Bacchus, isoform B OS=Drosophila melanogaster GN=Bacc PE=2 SV=1 - [Q9VQF7_DROME]	29.38	34.21	1	5	5	14	3.439E8	152	15.5	4.04
Q9W401	Probable citrate synthase, mitochondrial OS=Drosophila melanogaster GN=kdn PE=2 SV=1 - [CISY_DROME]	29.27	24.78	1	10	10	23	1.417E8	464	51.5	8.81
Q8MSW0	Isoleucyl-tRNA synthetase, isoform A OS=Drosophila melanogaster GN=Aats-ile PE=2 SV=1 - [Q8MSW0_DROME]	29.08	16.44	1	18	18	23	9.836E7	1229	141.0	7.52
Q9VZU7	Ubiquitin carboxyl-terminal hydrolase OS=Drosophila melanogaster GN=CG12082 PE=2 SV=1 - [Q9VZU7_DROME]	28.99	14.27	1	9	9	17	3.097E7	827	92.0	5.49
M9PHG2	Moesin, isoform M OS=Drosophila melanogaster GN=Moe PE=4 SV=1 - [M9PHG2_DROME]	28.98	25.51	3	12	12	17	1.548E8	584	68.8	5.99
M9PEJ8	Rhea, isoform H OS=Drosophila melanogaster GN=rhea PE=4 SV=1 - [M9PEJ8_DROME]	28.87	5.36	4	8	8	11	2.421E7	2689	291.4	6.06
Q9W2M4	CG10527 OS=Drosophila melanogaster GN=CG10527 PE=4 SV=1 - [Q9W2M4_DROME]	28.49	20.95	1	4	4	12	5.337E7	296	31.5	4.77
Q7KVQ0	Probable H/ACA ribonucleoprotein complex subunit 1 OS=Drosophila melanogaster GN=CG4038 PE=1 SV=1 - [GAR1_DROME]	28.32	32.49	1	6	6	18	2.572E8	237	22.7	11.19
Q95WY3	FI04781p OS=Drosophila melanogaster GN=Nop56 PE=2 SV=1 - [Q95WY3_DROME]	28.23	23.19	1	9	9	15	2.452E8	496	54.8	9.28
P28668	Bifunctional glutamate/proline--tRNA ligase OS=Drosophila melanogaster GN=Aats-glupro PE=1 SV=2 - [SYEP_DROME]	28.23	11.32	1	15	15	21	9.768E7	1714	189.3	8.63
Q9VXR5	CG9281, isoform B OS=Drosophila melanogaster GN=CG9281 PE=2 SV=1 - [Q9VXR5_DROME]	28.16	19.97	1	11	11	18	1.027E8	611	69.5	7.53
Q7JWQ7	CG3074, isoform A OS=Drosophila melanogaster GN=Swim PE=2 SV=1 - [Q7JWQ7_DROME]	27.91	18.79	1	6	6	11	5.832E7	431	48.8	7.08
P48602	V-type proton ATPase catalytic subunit A isoform 1 OS=Drosophila melanogaster GN=Vha68-1 PE=2 SV=2 - [VATA1_DROME]	27.77	12.54	2	1	6	12	6.245E7	614	68.3	5.29
P13395	Spectrin alpha chain OS=Drosophila melanogaster GN=alpha-Spec PE=1 SV=2 - [SPTCA_DROME]	27.70	6.67	4	13	13	19	3.886E7	2415	278.1	5.20
A1ZBE9	CG15100 OS=Drosophila melanogaster GN=CG15100 PE=3 SV=1 - [A1ZBE9_DROME]	27.65	12.23	1	9	9	14	8.335E7	1022	112.4	8.51
Q9VU35	CG11267-PA OS=Drosophila melanogaster GN=CG11267 PE=2 SV=1 - [Q9VU35_DROME]	27.63	58.25	1	6	6	18	7.072E8	103	11.0	9.09
Q9V3K3	RuvB-like helicase 2 OS=Drosophila melanogaster GN=rept PE=1 SV=1 - [RUVB2_DROME]	27.42	10.19	1	5	5	11	1.713E7	481	53.5	5.85
Q9VT32	CG6767, isoform A OS=Drosophila melanogaster GN=CG6767 PE=2 SV=2 - [Q9VT32_DROME]	27.39	17.14	6	5	5	13	1.057E8	350	38.3	7.44
Q9XZJ4	Proteasome subunit alpha type-6 OS=Drosophila melanogaster GN=Prosalpha1 PE=1 SV=2 - [PSA6_DROME]	27.34	36.48	1	10	10	17	2.972E8	244	27.1	7.66
Q9VB46	FI18644p1 OS=Drosophila melanogaster GN=Hmu PE=2 SV=1 - [Q9VB46_DROME]	27.31	19.34	1	8	8	15	5.158E7	579	63.4	9.35
Q5BI73	Chorion protein a at 7F OS=Drosophila melanogaster GN=Cp7Fa PE=2 SV=1 - [Q5BI73_DROME]	27.21	47.78	1	10	10	20	2.084E8	293	32.2	5.64
Q9VBU9	40S ribosomal protein S27 OS=Drosophila melanogaster GN=RpS27 PE=1 SV=1 - [Q9VBU9_DROME]	27.17	41.67	1	5	5	19	9.340E8	84	9.4	9.52
Q01637	Uridine 5'-monophosphate synthase OS=Drosophila melanogaster GN=r-l PE=2 SV=2 - [UMPS_DROME]	27.16	11.36	1	4	4	15	1.522E8	493	53.4	7.25
Q9VNF3	CG12171 OS=Drosophila melanogaster GN=CG12171 PE=2 SV=1 - [Q9VNF3_DROME]	27.12	26.85	1	4	6	14	4.675E7	257	26.9	7.50
Q7JXZ2	Eip55E OS=Drosophila melanogaster GN=Eip55E PE=2 SV=1 - [Q7JXZ2_DROME]	27.11	22.14	1	7	7	13	5.361E7	393	43.0	7.62
Q9VCZ8	Probable prefoldin subunit 5 OS=Drosophila melanogaster GN=CG7048 PE=2 SV=1 - [PFD5_DROME]	27.09	33.33	1	4	4	12	3.957E7	168	19.1	7.33
Q8SXM8	Lysine--tRNA ligase OS=Drosophila melanogaster GN=Aats-lys PE=2 SV=1 - [Q8SXM8_DROME]	27.02	18.29	2	9	9	11	5.309E7	574	64.6	6.51
M9PE40	Ubiquitin carboxyl-terminal hydrolase OS=Drosophila melanogaster GN=Uch PE=3 SV=1 - [M9PE40_DROME]	26.84	25.89	2	4	4	11	3.624E8	224	25.4	5.49
Q9VBH8	FI02850p OS=Drosophila melanogaster GN=RpL34a PE=1 SV=2 - [Q9VBH8_DROME]	26.78	30.86	1	1	7	17	7.204E8	162	18.1	11.44
C4NYP8	Hsc70-interacting protein 1 OS=Drosophila melanogaster GN=HIP PE=1 SV=2 - [F10A1_DROME]	26.48	17.77	1	6	6	13	1.726E8	377	41.0	5.35
P25161	Probable 26S proteasome non-ATPase regulatory subunit 3 OS=Drosophila melanogaster GN=Rpn3 PE=2 SV=1 - [PSMD3_DROME]	26.31	18.42	1	9	9	13	1.110E8	494	56.0	9.00
Q7PLL3	Eukaryotic initiation factor 4B OS=Drosophila melanogaster GN=eIF-4B PE=2 SV=1 - [Q7PLL3_DROME]	26.22	21.79	4	7	7	12	1.134E8	459	52.2	6.96
Q24009	Protein bicaudal C OS=Drosophila melanogaster GN=BicC PE=1 SV=2 - [BICC_DROME]	26.21	12.49	1	6	6	10	1.047E8	905	97.8	8.66
Q9VFE4	40S ribosomal protein S5b OS=Drosophila melanogaster GN=RpS5b PE=2 SV=1 - [RS5B_DROME]	26.18	28.70	1	2	9	15	1.190E9	230	25.7	8.54

Accession	Description	Score	Coverage	# Proteins	# Unique Peptides	# Peptides	# PSMs	Area	# AAs	MW [kDa]	calc. pI
P37276	Dynein heavy chain, cytoplasmic OS=Drosophila melanogaster GN=Dhc64C PE=2 SV=2 - [DYHC_DROME]	26.10	5.07	7	18	18	18	3.085E8	4639	529.9	6.33
P49630	60S ribosomal protein L36 OS=Drosophila melanogaster GN=RpL36 PE=1 SV=1 - [RL36_DROME]	26.05	38.26	1	6	6	15	9.665E8	115	13.5	11.37
Q95083	Proteasome subunit alpha type-5 OS=Drosophila melanogaster GN=Prosalpha5 PE=2 SV=2 - [PSA5_DROME]	26.03	34.43	1	7	7	13	1.847E8	244	26.9	4.98
Q9VJ39	Leukotriene A(4) hydrolase OS=Drosophila melanogaster GN=CG10602 PE=3 SV=3 - [Q9VJ39_DROME]	25.96	20.55	4	9	9	13	9.398E7	613	68.4	5.50
P17336	Catalase OS=Drosophila melanogaster GN=Cat PE=1 SV=2 - [CATA_DROME]	25.90	25.69	1	8	8	15	4.561E7	506	57.1	8.18
Q9VE79	CG14309 OS=Drosophila melanogaster GN=CG14309 PE=4 SV=2 - [Q9VE79_DROME]	25.77	14.08	1	10	10	14	7.002E7	966	110.5	8.16
O76927	40S ribosomal protein S21 OS=Drosophila melanogaster GN=RpS21 PE=1 SV=1 - [RS21_DROME]	25.77	32.53	1	4	4	10	6.754E8	83	9.2	5.91
A0AQH0	Proteasome subunit beta type OS=Drosophila melanogaster GN=Prosbeta1 PE=2 SV=1 - [A0AQH0_DROME]	25.75	39.73	1	5	5	12	6.242E7	224	24.2	5.25
Q9VH95	Uncharacterized protein CG16817 OS=Drosophila melanogaster GN=CG16817 PE=1 SV=1 - [YC17_DROME]	25.66	17.93	1	5	5	15	1.609E8	184	20.7	4.60
Q9W3F6	CG1440, isoform A OS=Drosophila melanogaster GN=CG1440 PE=2 SV=2 - [Q9W3F6_DROME]	25.29	19.47	4	8	8	14	6.931E7	488	55.1	6.01
Q0E924	LD46344p OS=Drosophila melanogaster GN=Phb2 PE=2 SV=2 - [Q0E924_DROME]	25.20	31.10	3	9	9	19	3.461E8	299	33.0	9.54
Q9VL70	CG4600-PA OS=Drosophila melanogaster GN=yip2 PE=2 SV=1 - [Q9VL70_DROME]	24.81	27.89	1	7	7	11	5.174E7	398	41.6	8.51
P25171	Regulator of chromosome condensation OS=Drosophila melanogaster GN=Rcc1 PE=1 SV=2 - [RCC1_DROME]	24.75	16.64	1	5	5	10	5.232E7	547	58.8	7.94
Q9W4M4	CG15570 OS=Drosophila melanogaster GN=CG15570 PE=4 SV=1 - [Q9W4M4_DROME]	24.74	6.48	1	6	6	10	1.642E8	1357	136.8	5.38
P48598	Eukaryotic translation initiation factor 4E OS=Drosophila melanogaster GN=eIF-4E PE=1 SV=1 - [IF4E_DROME]	24.69	33.20	1	6	6	15	3.721E8	259	29.2	6.24
O18413	26S protease regulatory subunit 8 OS=Drosophila melanogaster GN=Rpt6 PE=1 SV=2 - [PRS8_DROME]	24.61	20.74	1	1	7	12	8.638E7	405	45.8	8.41
Q9VUQ5	Protein argonaute-2 OS=Drosophila melanogaster GN=AGO2 PE=1 SV=3 - [AGO2_DROME]	24.47	21.00	2	6	6	9	6.570E7	1214	136.8	9.61
O97477	Inositol-3-phosphate synthase OS=Drosophila melanogaster GN=Inos PE=1 SV=1 - [INO1_DROME]	24.40	16.11	1	7	7	13	9.587E7	565	62.2	6.23
O46067	CG2918, isoform B OS=Drosophila melanogaster GN=EG:25E8.1 PE=2 SV=1 - [O46067_DROME]	24.35	10.18	1	6	6	12	1.923E7	923	103.4	5.12
Q9VDL2	LD40493p OS=Drosophila melanogaster GN=TFAM PE=2 SV=1 - [Q9VDL2_DROME]	24.33	29.57	2	7	7	11	6.814E7	257	29.9	9.99
Q9V895	Acidic leucine-rich nuclear phosphoprotein 32 family member A OS=Drosophila melanogaster GN=Anp32a PE=2 SV=1 - [AN32A_DROME]	24.32	21.84	2	5	5	11	1.219E8	261	29.2	4.21
P52029	Glucose-6-phosphate isomerase OS=Drosophila melanogaster GN=Pgi PE=2 SV=2 - [G6PI_DROME]	24.28	14.70	1	5	5	17	1.456E8	558	62.3	7.12
O77410	Eukaryotic translation initiation factor 3 subunit E OS=Drosophila melanogaster GN=eIF3-S6 PE=2 SV=1 - [EIF3E_DROME]	24.21	17.01	1	7	7	14	2.093E8	435	51.1	6.79
Q9VMB9	CG11015-PA OS=Drosophila melanogaster GN=CoVb PE=2 SV=1 - [Q9VMB9_DROME]	24.17	31.67	1	3	3	17	1.800E8	120	13.6	8.69
Q8SXC2	CG6950, isoform A OS=Drosophila melanogaster GN=CG6950-RC PE=2 SV=1 - [Q8SXC2_DROME]	24.12	8.00	1	2	2	8	4.781E7	450	51.2	7.72
Q7KRU8	Ferritin OS=Drosophila melanogaster GN=Fer1HCH PE=2 SV=1 - [Q7KRU8_DROME]	24.09	20.49	2	3	3	11	2.973E7	205	23.1	5.87
Q7K4L8	CG7878 OS=Drosophila melanogaster GN=CG7878 PE=2 SV=1 - [Q7K4L8_DROME]	24.08	6.97	1	3	4	11	3.483E7	703	78.5	6.70
P35415	Paramyosin, long form OS=Drosophila melanogaster GN=Prm PE=1 SV=1 - [MYSP1_DROME]	24.06	12.97	2	9	9	13	1.546E8	879	102.3	5.58
M9MS06	Alpha actinin, isoform D OS=Drosophila melanogaster GN=Actn PE=4 SV=1 - [M9MS06_DROME]	24.04	10.39	4	8	8	13	3.183E7	895	103.8	5.64
P17917	Proliferating cell nuclear antigen OS=Drosophila melanogaster GN=PCNA PE=1 SV=2 - [PCNA_DROME]	23.91	37.69	1	8	8	16	2.180E8	260	28.8	4.81
Q8INM3	Protein slender lobes OS=Drosophila melanogaster GN=sle PE=1 SV=1 - [SLE_DROME]	23.90	9.72	2	10	10	12	7.738E7	1420	158.6	5.24
P05205	Heterochromatin protein 1 OS=Drosophila melanogaster GN=Su(var)205 PE=1 SV=2 - [HP1_DROME]	23.87	16.99	1	2	2	11	7.786E7	206	23.2	5.08
A4UZW2	S-adenosylmethionine synthase OS=Drosophila melanogaster GN=Sam-S PE=3 SV=1 - [A4UZW2_DROME]	23.75	18.38	11	6	6	16	1.024E8	408	44.8	6.54
Q9W1H8	GH13256p OS=Drosophila melanogaster GN=Thiolase PE=2 SV=1 - [Q9W1H8_DROME]	23.73	13.65	1	6	6	14	1.225E8	469	50.6	9.14
Q9W229	40S ribosomal protein S24 OS=Drosophila melanogaster GN=RpS24 PE=1 SV=1 - [Q9W229_DROME]	23.72	17.56	1	3	3	12	3.963E8	131	15.0	11.22
P06754	Tropomyosin-1, isoforms 9A/A/B OS=Drosophila melanogaster GN=Tm1 PE=2 SV=2 - [TPM1_DROME]	23.59	42.48	5	4	16	19	2.686E8	339	39.3	4.94
Q9VA54	Rpt6R OS=Drosophila melanogaster GN=Rpt6R PE=2 SV=1 - [Q9VA54_DROME]	23.58	20.80	1	1	7	12	7.400E7	399	45.1	7.97
Q9VZ69	CG1691-PA, isoform A OS=Drosophila melanogaster GN=Imp PE=2 SV=2 - [Q9VZ69_DROME]	23.49	14.66	5	7	7	12	1.320E8	566	62.1	9.61
Q8I0J1	CG1910, isoform B OS=Drosophila melanogaster GN=CG1910 PE=2 SV=1 - [Q8I0J1_DROME]	23.42	23.01	3	7	7	10	5.806E7	439	45.9	4.49
Q9VQR8	CG33123, isoform A OS=Drosophila melanogaster GN=CG33123 PE=3 SV=2 - [Q9VQR8_DROME]	23.12	18.70	1	15	15	21	1.349E8	1182	134.8	7.64
Q9V455	Importin subunit alpha OS=Drosophila melanogaster GN=Kap-alpha3 PE=2 SV=1 - [Q9V455_DROME]	23.00	11.67	1	4	4	7	4.770E7	514	57.0	5.26
P56538	Eukaryotic translation initiation factor 6 OS=Drosophila melanogaster GN=eIF6 PE=2 SV=3 - [IF6_DROME]	22.96	37.96	1	5	5	11	1.242E8	245	26.5	4.73
A1ZBJ2	CG7461 OS=Drosophila melanogaster GN=CG7461 PE=3 SV=2 - [A1ZBJ2_DROME]	22.93	16.43	1	9	9	11	7.726E7	627	68.3	7.77
P53777	Muscle LIM protein 1 OS=Drosophila melanogaster GN=Mlp60A PE=2 SV=1 - [MLP1_DROME]	22.73	51.09	2	3	4	8	2.858E7	92	10.0	8.24
Q9V3G7	26S proteasome non-ATPase regulatory subunit 6 OS=Drosophila melanogaster GN=Rpn7 PE=2 SV=1 - [PSMD6_DROME]	22.40	25.71	10	8	8	14	1.297E8	389	45.4	6.48
Q9VSR5	CG5012-PA OS=Drosophila melanogaster GN=mRpL12 PE=2 SV=1 - [Q9VSR5_DROME]	22.25	42.31	1	7	7	15	2.604E8	182	19.6	8.75
P04359	60S ribosomal protein L32 OS=Drosophila melanogaster GN=RpL32 PE=1 SV=3 - [RL32_DROME]	22.20	32.84	1	5	5	14	8.653E8	134	16.0	11.40
Q9VAC1	CG7920, isoform A OS=Drosophila melanogaster GN=CG7920 PE=2 SV=1 - [Q9VAC1_DROME]	22.19	22.43	2	8	8	15	2.023E8	477	51.8	8.12
P36179	Serine/threonine-protein phosphatase PP2A 65 kDa regulatory subunit OS=Drosophila melanogaster GN=Pp2A-29B PE=2 SV=4 - [2AAA_DROME]	22.17	20.30	3	10	10	13	1.705E8	591	65.4	5.05
Q9V4E0	CG1970, isoform B OS=Drosophila melanogaster GN=CG1970-RA PE=2 SV=2 - [Q9V4E0_DROME]	22.06	13.68	1	4	4	13	2.788E7	468	52.9	6.67

Accession	Description	Score	Coverage	# Proteins	# Unique Peptides	# Peptides	# PSMs	Area	# AAs	MW [kDa]	calc. pI
P48601	26S protease regulatory subunit 4 OS=Drosophila melanogaster GN=Rpt2 PE=1 SV=2 - [PRS4_DROME]	22.04	23.92	1	8	8	17	1.786E8	439	49.3	6.58
Q9VHI8	CG11980, isoform A OS=Drosophila melanogaster GN=CG11980 PE=4 SV=2 - [Q9VHI8_DROME]	22.01	15.36	3	4	4	8	1.482E8	345	39.2	6.54
Q9VAN0	Probable phosphoserine aminotransferase OS=Drosophila melanogaster GN=CG11899 PE=2 SV=1 - [SERC_DROME]	21.99	23.08	1	7	7	17	8.777E7	364	39.5	8.41
Q27294	RNA-binding protein cabeza OS=Drosophila melanogaster GN=caz PE=2 SV=2 - [CAZ_DROME]	21.92	23.56	3	5	5	10	6.650E7	399	38.8	9.36
Q27889	Serine/threonine-protein phosphatase 2B catalytic subunit 2 OS=Drosophila melanogaster GN=Pp2B-14D PE=1 SV=2 - [PP2B2_DROME]	21.92	15.79	3	7	7	11	6.819E7	570	63.1	5.88
Q9VHJ8	SkpA associated protein, isoform A OS=Drosophila melanogaster GN=skap PE=3 SV=2 - [Q9VHJ8_DROME]	21.85	16.14	2	6	6	11	1.264E8	502	54.8	8.66
Q9VLK2	Ribosomal L1 domain-containing protein CG13096 OS=Drosophila melanogaster GN=CG13096 PE=1 SV=1 - [Y3096_DROME]	21.84	14.24	1	8	8	11	1.267E8	681	74.3	8.51
Q9W125	CG3683 protein OS=Drosophila melanogaster GN=NDUFA8 PE=2 SV=1 - [Q9W125_DROME]	21.57	28.57	1	4	4	10	3.968E7	175	19.8	6.87
P84029	Cytochrome c-2 OS=Drosophila melanogaster GN=Cyt-c-p PE=1 SV=2 - [CYC2_DROME]	21.56	37.96	1	5	5	15	2.454E8	108	11.7	9.57
Q9Y125	BcDNA.GH08312 OS=Drosophila melanogaster GN=Sap-r PE=2 SV=1 - [Q9Y125_DROME]	21.52	10.81	2	9	9	15	1.307E8	953	105.9	4.97
M9PHR2	Upheld, isoform O OS=Drosophila melanogaster GN=up PE=4 SV=1 - [M9PHR2_DROME]	21.51	17.63	8	6	6	11	5.304E7	397	47.5	4.67
Q9W414	Regulatory particle triple-A ATPase 4 OS=Drosophila melanogaster GN=Rpt4 PE=3 SV=3 - [Q9W414_DROME]	21.34	23.43	2	9	9	15	7.905E7	397	44.9	8.07
Q9V9M7	RE62581p OS=Drosophila melanogaster GN=RpL21 PE=1 SV=1 - [Q9V9M7_DROME]	21.21	25.79	1	5	5	12	4.555E8	159	18.5	10.55
Q8SYJ2	CG32230, isoform B OS=Drosophila melanogaster GN=CG32230 PE=2 SV=1 - [Q8SYJ2_DROME]	21.19	37.35	1	4	4	11	1.358E8	83	9.4	9.26
Q9VEN1	Filamin-A OS=Drosophila melanogaster GN=cher PE=1 SV=2 - [FLNA_DROME]	21.18	7.51	4	13	13	15	3.189E7	2210	239.0	6.11
P22769	Proteasome subunit alpha type-7-1 OS=Drosophila melanogaster GN=Prosalpha4 PE=1 SV=2 - [PSA71_DROME]	20.89	27.71	2	7	7	11	1.769E8	249	28.0	8.12
A1ZAH8	Ribosomal protein S15, isoform B OS=Drosophila melanogaster GN=RpS15 PE=2 SV=1 - [A1ZAH8_DROME]	20.78	22.45	2	4	4	12	7.219E8	147	17.0	10.33
M9PE74	No circadian temperature entrainment, isoform D OS=Drosophila melanogaster GN=nocte PE=4 SV=1 - [M9PE74_DROME]	20.71	8.33	2	10	10	11	3.260E7	2305	234.9	9.25
P22058	Chromosomal protein D1 OS=Drosophila melanogaster GN=D1 PE=1 SV=3 - [CPD1_DROME]	20.55	25.92	1	7	7	11	1.055E8	355	37.0	6.64
Q9W3N9	CG10932, isoform A OS=Drosophila melanogaster GN=CG10932 PE=2 SV=1 - [Q9W3N9_DROME]	20.54	20.49	1	5	5	8	3.444E7	410	43.4	8.68
M9PCG1	CG5261, isoform E OS=Drosophila melanogaster GN=CG5261 PE=3 SV=1 - [M9PCG1_DROME]	20.53	19.56	5	9	9	16	5.529E7	496	52.8	9.55
P20477	Glutamine synthetase 1, mitochondrial OS=Drosophila melanogaster GN=Gs1 PE=2 SV=3 - [GLNA1_DROME]	20.35	22.31	1	4	4	10	6.535E7	399	44.4	6.46
Q9VN50	Eukaryotic translation initiation factor 3 subunit F-1 OS=Drosophila melanogaster GN=eIF3-S5-1 PE=2 SV=1 - [EI3F1_DROME]	20.23	22.50	1	4	4	8	5.906E7	280	31.1	6.52
Q9W2X6	Lethal (1) G0230, isoform A OS=Drosophila melanogaster GN=l(1)G0230 PE=2 SV=1 - [Q9W2X6_DROME]	20.21	42.68	1	6	6	10	2.411E8	157	16.7	5.99
Q9VZF9	Cuticular protein 64Ad OS=Drosophila melanogaster GN=Cpr64Ad PE=2 SV=2 - [Q9VZF9_DROME]	20.20	28.34	1	4	4	9	1.419E7	247	24.3	5.45
Q9VJ43	LD10783p OS=Drosophila melanogaster GN=ScpX PE=2 SV=1 - [Q9VJ43_DROME]	20.17	13.05	2	5	5	8	6.785E7	544	59.0	7.78
P80455	40S ribosomal protein S12 OS=Drosophila melanogaster GN=RpS12 PE=1 SV=2 - [RS12_DROME]	19.98	51.80	1	7	7	13	4.998E8	139	15.2	6.38
Q9VR96	IP02009p OS=Drosophila melanogaster GN=Obp19c PE=2 SV=1 - [Q9VR96_DROME]	19.87	29.71	1	4	4	9	1.660E8	175	19.4	9.07
Q9W0Y1	Troponin C-akin-1 protein OS=Drosophila melanogaster GN=Tina-1 PE=2 SV=1 - [TINA1_DROME]	19.81	26.35	2	3	3	10	1.463E7	167	19.0	7.44
A1Z9E3	Elongation factor Tu OS=Drosophila melanogaster GN=EfTuM PE=3 SV=1 - [A1Z9E3_DROME]	19.78	16.16	1	5	5	10	1.362E8	489	54.0	8.03
Q9U9Q4	Eukaryotic translation initiation factor 3 subunit H OS=Drosophila melanogaster GN=eIF-3p40 PE=2 SV=2 - [EIF3H_DROME]	19.69	19.23	1	6	6	13	1.294E8	338	38.4	6.10
Q9VV60	LD21116p OS=Drosophila melanogaster GN=Aats-tyr PE=2 SV=1 - [Q9VV60_DROME]	19.61	22.86	1	10	10	11	1.921E8	525	58.1	6.87
Q9W3Y3	CG3226-PA OS=Drosophila melanogaster GN=CG3226 PE=2 SV=1 - [Q9W3Y3_DROME]	19.60	19.57	1	4	4	10	7.897E7	230	25.8	6.96
Q9XZU1	Exportin-2 OS=Drosophila melanogaster GN=Cas PE=2 SV=2 - [XPO2_DROME]	19.46	6.15	1	5	5	10	1.242E8	975	110.1	5.82
Q24492	Replication protein A 70 kDa DNA-binding subunit OS=Drosophila melanogaster GN=RpA-70 PE=1 SV=1 - [RFA1_DROME]	19.42	18.74	1	11	11	13	2.297E8	603	66.6	6.76
Q9VLB7	GDP dissociation inhibitor, isoform A OS=Drosophila melanogaster GN=Gdi PE=2 SV=1 - [Q9VLB7_DROME]	19.37	26.64	1	11	11	26	3.900E8	443	49.9	5.72
Q9W3W8	60S ribosomal protein L17 OS=Drosophila melanogaster GN=RpL17 PE=1 SV=1 - [RL17_DROME]	19.31	20.97	1	6	6	8	5.997E7	186	21.6	10.30
Q9VKM3	CG6105-PA OS=Drosophila melanogaster GN=l(2)06225 PE=2 SV=1 - [Q9VKM3_DROME]	19.30	15.15	1	1	1	8	1.497E8	99	10.9	9.76
Q9VTY2	CG10638, isoform A OS=Drosophila melanogaster GN=CG10638 PE=4 SV=1 - [Q9VTY2_DROME]	19.27	16.72	2	4	4	12	1.093E8	317	35.8	6.11
P32234	GTP-binding protein 128up OS=Drosophila melanogaster GN=128up PE=2 SV=2 - [128UP_DROME]	19.19	22.83	1	6	6	11	4.471E7	368	41.1	8.60
Q9VVC5	Neural conserved at 73EF, isoform A OS=Drosophila melanogaster GN=Nc73EF PE=2 SV=2 - [Q9VVC5_DROME]	19.14	5.56	3	4	4	11	3.570E7	1008	112.5	6.89
Q27268	ATP-dependent RNA helicase WM6 OS=Drosophila melanogaster GN=Hel25E PE=1 SV=1 - [DX39B_DROME]	19.05	16.04	2	7	7	12	1.835E8	424	48.6	5.66
Q9VL89	Methionine aminopeptidase 2 OS=Drosophila melanogaster GN=und PE=2 SV=1 - [Q9VL89_DROME]	19.03	23.44	1	10	10	14	1.637E8	448	49.8	6.68
Q960M4	LD45324p OS=Drosophila melanogaster GN=Prx5 PE=2 SV=1 - [Q960M4_DROME]	19.02	39.47	1	5	5	11	9.516E7	190	19.9	8.68
Q9VB10	CG5590 OS=Drosophila melanogaster GN=CG5590 PE=2 SV=1 - [Q9VB10_DROME]	18.98	15.05	1	4	4	10	2.112E7	412	44.3	8.02
P54357	Myosin-2 essential light chain OS=Drosophila melanogaster GN=Mlc-c PE=1 SV=1 - [MLC2_DROME]	18.97	38.10	2	6	6	12	9.945E7	147	16.6	4.59
Q8IRG6	FACT complex subunit spt16 OS=Drosophila melanogaster GN=dre4 PE=1 SV=2 - [SPT16_DROME]	18.88	8.96	1	7	7	10	3.734E7	1083	123.5	6.29
Q24154	60S ribosomal protein L29 OS=Drosophila melanogaster GN=RpL29 PE=1 SV=1 - [RL29_DROME]	18.78	32.89	1	4	4	7	1.161E9	76	8.9	11.34
Q94514	Cytochrome c oxidase subunit 5A, mitochondrial OS=Drosophila melanogaster GN=CoVa PE=1 SV=2 - [COX5A_DROME]	18.77	51.01	1	8	8	20	2.286E8	149	16.6	5.68
Q95RA9	CG9796 OS=Drosophila melanogaster GN=GILT1 PE=2 SV=1 - [Q95RA9_DROME]	18.68	34.00	1	5	5	12	6.365E7	250	27.7	7.33

Accession	Description	Score	Coverage	# Proteins	# Unique Peptides	# Peptides	# PSMs	Area	# AAs	MW [kDa]	calc. pI
P35421	Phosphoribosylformylglycinamidine synthase OS=Drosophila melanogaster GN=ade2 PE=1 SV=2 - [PUR4_DROME]	18.57	8.12	1	6	6	7	1.934E7	1354	148.0	5.68
Q9VRD9	Cystathionine beta-synthase OS=Drosophila melanogaster GN=Cbs PE=1 SV=1 - [Q9VRD9_DROME]	18.44	10.54	1	4	4	9	4.113E7	522	56.8	7.06
Q59E30	Failed axon connections, isoform C OS=Drosophila melanogaster GN=fax PE=4 SV=1 - [Q59E30_DROME]	18.43	25.54	6	8	8	9	1.102E8	415	46.6	5.25
P18053	Proteasome subunit alpha type-4 OS=Drosophila melanogaster GN=Prosalpha3 PE=1 SV=2 - [PSA4_DROME]	18.42	29.92	2	6	6	11	4.130E8	264	29.4	7.15
Q95RF6	CG7654-PA OS=Drosophila melanogaster GN=Tom20 PE=2 SV=1 - [Q95RF6_DROME]	18.35	13.45	1	1	1	5	1.485E7	171	18.8	7.24
Q24572	Probable histone-binding protein Caf1 OS=Drosophila melanogaster GN=Caf1 PE=1 SV=1 - [CAF1_DROME]	18.34	13.49	3	5	5	8	1.531E8	430	48.6	4.89
Q9VIE7	Burgundy, isoform B OS=Drosophila melanogaster GN=bur PE=2 SV=3 - [Q9VIE7_DROME]	18.33	11.13	1	5	5	9	3.291E7	683	76.7	6.80
P20240	Otefin OS=Drosophila melanogaster GN=Ote PE=1 SV=2 - [OTE_DROME]	18.30	25.71	1	7	7	8	5.587E7	424	46.6	9.55
Q9VYW3	CG15220-PA OS=Drosophila melanogaster GN=RPA3 PE=1 SV=1 - [Q9VYW3_DROME]	18.20	26.79	1	2	2	6	1.354E8	112	12.0	5.43
Q9VTU4	Eukaryotic translation initiation factor 3 subunit L OS=Drosophila melanogaster GN=CG5642 PE=2 SV=1 - [EIF3L_DROME]	18.16	13.73	1	7	7	13	6.743E7	539	63.2	5.96
Q9U915	Adenylate kinase OS=Drosophila melanogaster GN=Adk2 PE=1 SV=1 - [KAD2_DROME]	18.16	36.67	1	7	7	9	4.538E7	240	26.5	7.88
A1Z7J7	Sec31, isoform A OS=Drosophila melanogaster GN=Sec31 PE=4 SV=1 - [A1Z7J7_DROME]	18.14	4.52	2	3	3	6	2.117E7	1240	135.9	6.14
Q9V3W7	LD40489p OS=Drosophila melanogaster GN=SF2 PE=2 SV=1 - [Q9V3W7_DROME]	17.96	21.18	1	5	5	15	8.696E7	255	28.3	9.91
Q7JRC0	CG12384 OS=Drosophila melanogaster GN=CG12384 PE=2 SV=1 - [Q7JRC0_DROME]	17.71	26.04	1	1	1	6	4.432E7	96	9.9	9.31
Q9V405	26S proteasome regulatory complex subunit p48A OS=Drosophila melanogaster GN=Rpt3 PE=2 SV=1 - [Q9V405_DROME]	17.67	19.13	2	7	7	12	1.041E8	413	47.0	5.38
Q7K7G0	Coat protein (Coatomer) delta, isoform B OS=Drosophila melanogaster GN=deltaCOP PE=4 SV=1 - [Q7K7G0_DROME]	17.64	14.31	2	5	5	7	2.327E7	531	57.8	6.20
Q9VMV9	Reticulon-like protein OS=Drosophila melanogaster GN=Rtnl1 PE=4 SV=2 - [Q9VMV9_DROME]	17.52	20.17	6	5	8	11	4.703E7	595	63.9	4.83
Q7K3B7	CG11208 OS=Drosophila melanogaster GN=CG11208 PE=2 SV=1 - [Q7K3B7_DROME]	17.48	10.92	1	4	4	8	5.879E7	568	62.2	7.47
A4V4J0	Shibire, isoform C OS=Drosophila melanogaster GN=shi PE=3 SV=1 - [A4V4J0_DROME]	17.47	5.66	7	4	4	6	4.069E7	830	93.0	8.24
Q9VW68	CG7433, isoform A OS=Drosophila melanogaster GN=CG7433 PE=3 SV=1 - [Q9VW68_DROME]	17.32	15.43	1	6	6	9	1.618E8	486	54.6	8.62
Q9VWA1	Clathrin light chain OS=Drosophila melanogaster GN=Clc PE=2 SV=1 - [CLC_DROME]	17.18	25.11	1	5	5	7	6.039E7	219	23.8	4.78
Q8IQZ8	CG8649-PC, isoform C OS=Drosophila melanogaster GN=Fim PE=2 SV=1 - [Q8IQZ8_DROME]	17.16	10.55	3	4	4	6	2.834E7	616	69.1	5.77
Q9VWT1	GH22474p OS=Drosophila melanogaster GN=Aats-his PE=2 SV=1 - [Q9VWT1_DROME]	17.15	17.43	3	7	7	8	6.189E7	522	57.7	6.95
O18333	GH01619p OS=Drosophila melanogaster GN=Rab2 PE=2 SV=1 - [O18333_DROME]	17.10	18.31	1	3	3	6	1.653E7	213	23.5	6.39
P47938	Thioredoxin-1 OS=Drosophila melanogaster GN=dhd PE=1 SV=1 - [THIO1_DROME]	17.10	22.43	1	3	3	8	2.414E8	107	12.4	9.41
Q7YU05	Pyruvate dehydrogenase E1 component subunit alpha OS=Drosophila melanogaster GN=l(1)G0334 PE=2 SV=1 - [Q7YU05_DROME]	17.08	14.63	3	4	4	9	9.443E7	328	35.9	6.54
Q9VH07	RuvB-like helicase 1 OS=Drosophila melanogaster GN=pont PE=1 SV=1 - [RUVB1_DROME]	17.08	27.63	1	9	9	15	3.749E7	456	50.2	7.34
Q9VP57	CG7752-PA OS=Drosophila melanogaster GN=pzg PE=2 SV=1 - [Q9VP57_DROME]	17.07	12.95	1	7	7	9	2.843E7	996	105.0	4.89
Q24319	Dolichyl-diphosphooligosaccharide--protein glycosyltransferase 48 kDa subunit OS=Drosophila melanogaster GN=Ost48 PE=2 SV=2 - [OST48_DROME]	17.06	15.59	1	4	4	9	3.819E7	449	49.9	5.59
Q9XZ32	CG10254, isoform A OS=Drosophila melanogaster GN=CG10254 PE=2 SV=1 - [Q9XZ32_DROME]	17.06	5.58	1	5	5	7	2.336E7	1398	156.5	5.20
Q9VKY2	Cullin-associated NEDD8-dissociated protein 1 OS=Drosophila melanogaster GN=Cand1 PE=2 SV=1 - [CAND1_DROME]	17.02	5.85	1	5	5	9	2.534E7	1248	139.3	5.87
Q9VKM8	CG33129, isoform B OS=Drosophila melanogaster GN=CG33129 PE=2 SV=1 - [Q9VKM8_DROME]	17.00	19.41	2	4	4	11	1.674E8	237	25.8	9.74
P48591	Ribonucleoside-diphosphate reductase large subunit OS=Drosophila melanogaster GN=RnrL PE=1 SV=2 - [RIR1_DROME]	16.93	7.27	1	5	5	8	3.523E7	812	91.9	7.46
Q8I0G5	Coatomer subunit gamma OS=Drosophila melanogaster GN=gammaCop PE=2 SV=1 - [COPG_DROME]	16.88	10.08	1	6	6	13	6.410E7	883	97.6	5.67
Q9VBN5	60S ribosomal protein L27 OS=Drosophila melanogaster GN=RpL27 PE=1 SV=1 - [Q9VBN5_DROME]	16.87	29.63	1	5	5	13	9.151E8	135	15.9	10.61
P26686	Serine-arginine protein 55 OS=Drosophila melanogaster GN=B52 PE=1 SV=4 - [SRR55_DROME]	16.68	17.02	1	7	7	13	1.781E8	376	42.8	11.37
Q9VC94	CG5728 OS=Drosophila melanogaster GN=CG5728 PE=2 SV=2 - [Q9VC94_DROME]	16.60	5.17	1	6	6	9	2.369E7	1430	159.7	8.56
A1ZB80	CG5174, isoform A OS=Drosophila melanogaster GN=CG5174-RA PE=2 SV=1 - [A1ZB80_DROME]	16.54	35.10	8	5	5	13	8.269E7	208	22.5	5.33
Q9V597	60S ribosomal protein L31 OS=Drosophila melanogaster GN=RpL31 PE=1 SV=1 - [RL31_DROME]	16.40	38.71	1	5	5	10	9.747E8	124	14.5	10.20
Q9VDK7	Signal recognition particle subunit SRP72 OS=Drosophila melanogaster GN=Srp72 PE=2 SV=1 - [Q9VDK7_DROME]	16.40	14.62	1	6	6	12	3.118E7	650	72.8	9.06
Q03334	40S ribosomal protein S13 OS=Drosophila melanogaster GN=RpS13 PE=1 SV=3 - [RS13_DROME]	16.26	35.10	1	8	8	17	1.377E9	151	17.2	10.55
Q9VZI1	CG14996-PB OS=Drosophila melanogaster GN=Chd64 PE=2 SV=2 - [Q9VZI1_DROME]	16.25	56.38	2	8	8	13	1.858E8	188	20.6	8.43
Q9VWI2	NAT1 ortholog, isoform A OS=Drosophila melanogaster GN=Nat1 PE=2 SV=1 - [Q9VWI2_DROME]	16.19	9.33	1	7	7	11	8.280E7	890	103.0	7.27
Q7K3W2	CG8728 OS=Drosophila melanogaster GN=CG8728-RA PE=2 SV=1 - [Q7K3W2_DROME]	16.17	11.33	1	4	4	8	1.274E7	556	61.0	7.12
Q9VIQ8	CG10664-PA, isoform A OS=Drosophila melanogaster GN=CoIV PE=2 SV=1 - [Q9VIQ8_DROME]	16.14	10.99	1	3	3	6	2.500E8	182	20.5	8.25
P41092	60S ribosomal protein L27a OS=Drosophila melanogaster GN=RpL27A PE=1 SV=2 - [RL27A_DROME]	16.13	24.16	1	5	5	14	5.645E8	149	17.0	10.71
A1Z843	CG1371 OS=Drosophila melanogaster GN=CG1371 PE=4 SV=1 - [A1Z843_DROME]	16.09	8.42	1	9	9	12	4.345E7	1199	130.8	7.23
P28750	Maternal protein exuperantia OS=Drosophila melanogaster GN=exu PE=1 SV=2 - [EXU_DROME]	16.08	20.11	1	7	7	7	1.674E8	532	57.9	9.54
Q7K221	Glutamate oxaloacetate transaminase 1, isoform A OS=Drosophila melanogaster GN=Got1 PE=2 SV=1 - [Q7K221_DROME]	16.00	12.02	2	4	4	8	7.487E7	416	46.1	7.53
A1Z877	LP19846p OS=Drosophila melanogaster GN=Ndg PE=2 SV=1 - [A1Z877_DROME]	15.99	7.04	2	8	8	8	5.239E7	1350	149.0	4.59

Accession	Description	Score	Coverage	# Proteins	# Unique Peptides	# Peptides	# PSMs	Area	# AAs	MW [kDa]	calc. pI
Q9VZS3	Protein translation factor SUI1 homolog OS=Drosophila melanogaster GN=CG17737 PE=1 SV=1 - [SUI1_DROME]	15.95	62.73	1	6	6	11	5.450E7	110	12.4	7.34
O18373	Selenide, water dikinase OS=Drosophila melanogaster GN=SelD PE=1 SV=1 - [SPS1_DROME]	15.88	9.80	1	4	4	10	5.352E7	398	43.4	6.93
Q9VJD4	LD24721p OS=Drosophila melanogaster GN=Sgt PE=2 SV=1 - [Q9VJD4_DROME]	15.87	24.47	1	8	8	11	1.113E8	331	36.2	4.64
M9PD27	Eukaryotic release factor 1, isoform H OS=Drosophila melanogaster GN=eRF1 PE=4 SV=1 - [M9PD27_DROME]	15.81	17.62	3	6	6	13	5.787E7	437	49.0	6.09
Q9VF51	CG6045 OS=Drosophila melanogaster GN=CG6045-RA PE=2 SV=1 - [Q9VF51_DROME]	15.80	6.86	1	6	6	8	1.595E7	1254	137.8	6.57
E1JJD6	Twenty-four, isoform F OS=Drosophila melanogaster GN=tyf PE=4 SV=2 - [E1JJD6_DROME]	15.71	6.66	2	9	9	13	4.456E7	1667	173.6	6.70
Q9VNX4	Delta-1-Pyrroline-5-carboxylate dehydrogenase 1, isoform A OS=Drosophila melanogaster GN=P5CDh1 PE=2 SV=1 - [Q9VNX4_DROME]	15.67	16.72	1	7	7	12	6.607E7	574	63.6	8.22
Q9VS02	CG9953 OS=Drosophila melanogaster GN=CG9953 PE=4 SV=1 - [Q9VS02_DROME]	15.57	5.12	1	2	2	7	2.022E8	508	56.8	6.60
Q9Y105	Probable glutamine--tRNA ligase OS=Drosophila melanogaster GN=Aats-gln PE=1 SV=1 - [SYQ_DROME]	15.56	8.10	1	4	4	5	2.843E7	778	87.5	7.21
Q9W0H6	CG9149 OS=Drosophila melanogaster GN=CG9149 PE=3 SV=2 - [Q9W0H6_DROME]	15.56	15.31	1	3	3	5	1.539E7	392	41.1	7.05
P49735	DNA replication licensing factor Mcm2 OS=Drosophila melanogaster GN=Mcm2 PE=1 SV=1 - [MCM2_DROME]	15.54	9.36	1	6	6	8	3.845E7	887	100.4	5.11
P22464	Annexin B9 OS=Drosophila melanogaster GN=AnxB9 PE=2 SV=2 - [ANXB9_DROME]	15.52	20.99	1	6	6	10	6.580E7	324	36.0	4.88
Q7JV23	CG11198, isoform B OS=Drosophila melanogaster GN=ACC PE=2 SV=1 - [Q7JV23_DROME]	15.51	4.43	3	7	7	9	2.725E7	2323	261.8	6.07
Q8MMC4	Protein CDV3 homolog OS=Drosophila melanogaster GN=CG3760 PE=1 SV=1 - [CDV3_DROME]	15.45	39.85	1	7	7	8	1.307E8	271	29.6	5.53
Q9VMK3	CG9050-PA OS=Drosophila melanogaster GN=psd PE=4 SV=2 - [Q9VMK3_DROME]	15.41	8.66	1	3	3	11	9.046E7	381	40.5	4.70
Q00963	Spectrin beta chain OS=Drosophila melanogaster GN=beta-Spec PE=1 SV=2 - [SPTCB_DROME]	15.40	5.06	3	8	8	9	2.962E7	2291	265.6	5.34
Q9VFR0	Protein BCCIP homolog OS=Drosophila melanogaster GN=CG9286 PE=2 SV=2 - [BCCIP_DROME]	15.36	17.17	1	4	4	7	3.852E7	297	33.4	4.65
Q9V9Q4	CG1416, isoform A OS=Drosophila melanogaster GN=CG1416 PE=2 SV=2 - [Q9V9Q4_DROME]	15.30	14.41	1	4	4	6	1.010E8	354	40.1	6.57
Q9V438	Calcium-binding protein 1, isoform A OS=Drosophila melanogaster GN=CaBP1 PE=2 SV=1 - [Q9V438_DROME]	15.23	16.86	1	6	6	13	8.038E7	433	46.7	5.69
Q9VU68	Actin-interacting protein 1 OS=Drosophila melanogaster GN=flr PE=2 SV=1 - [WDR1_DROME]	15.17	22.70	1	9	9	13	3.260E7	608	66.5	6.73
O76922	Protein aubergine OS=Drosophila melanogaster GN=aub PE=1 SV=1 - [AUB_DROME]	15.10	10.39	1	6	6	9	3.450E7	866	98.5	9.28
Q9VIV8	CG13084-PA OS=Drosophila melanogaster GN=CG13084 PE=4 SV=1 - [Q9VIV8_DROME]	15.07	15.86	1	7	7	14	6.553E7	454	51.3	6.62
Q9VHS8	FI20117p1 OS=Drosophila melanogaster GN=eIF4AIII PE=2 SV=1 - [Q9VHS8_DROME]	15.03	6.27	1	1	3	5	5.519E8	399	45.6	6.02
Q9W0E4	Puromycin sensitive aminopeptidase, isoform A OS=Drosophila melanogaster GN=Psa PE=4 SV=1 - [Q9W0E4_DROME]	14.95	12.12	3	10	10	14	1.093E8	866	99.3	5.30
Q9V3H2	26S proteasome non-ATPase regulatory subunit 14 OS=Drosophila melanogaster GN=Rpn11 PE=1 SV=1 - [PSDE_DROME]	14.95	24.35	1	5	5	12	1.115E8	308	34.4	6.13
P61209	ADP-ribosylation factor 1 OS=Drosophila melanogaster GN=Arf79F PE=2 SV=2 - [ARF1_DROME]	14.93	14.29	1	1	2	7	4.727E7	182	20.7	6.58
P35128	Ubiquitin-conjugating enzyme E2 N OS=Drosophila melanogaster GN=ben PE=2 SV=1 - [UBE2N_DROME]	14.93	17.88	1	2	2	6	7.792E7	151	17.2	5.60
O97418	CG3621, isoform A OS=Drosophila melanogaster GN=CG3621-RA PE=2 SV=1 - [O97418_DROME]	14.91	34.95	1	2	2	9	8.287E7	103	11.5	7.34
P91928	MICOS complex subunit Mic60 OS=Drosophila melanogaster GN=CG6455 PE=2 SV=4 - [MIC60_DROME]	14.87	12.99	1	8	8	9	5.154E8	739	82.0	8.94
P55828	40S ribosomal protein S20 OS=Drosophila melanogaster GN=RpS20 PE=1 SV=1 - [RS20_DROME]	14.83	35.00	1	5	5	17	7.201E8	120	13.5	10.33
Q6NN28	RE21974p OS=Drosophila melanogaster GN=Tm1 PE=2 SV=1 - [Q6NN28_DROME]	14.81	46.32	1	1	13	15	2.686E8	285	32.7	4.74
Q7KN97	Pyruvate carboxylase OS=Drosophila melanogaster GN=CG1516 PE=2 SV=1 - [Q7KN97_DROME]	14.81	5.33	2	6	6	7	4.022E7	1181	130.8	6.81
Q9V3Z4	GH11341p OS=Drosophila melanogaster GN=Rpn5 PE=2 SV=1 - [Q9V3Z4_DROME]	14.77	19.12	1	7	7	12	1.751E8	502	57.7	5.80
Q7K4H4	Bifunctional lysine-specific demethylase and histidyl-hydroxylase NO66 OS=Drosophila melanogaster GN=CG2982 PE=1 SV=1 - [NO66_DROME]	14.77	20.06	1	8	8	9	6.935E7	653	73.1	7.84
Q9VWI0	CG12203-PA OS=Drosophila melanogaster GN=CG12203 PE=2 SV=2 - [Q9VWI0_DROME]	14.67	16.94	1	2	2	5	3.586E7	183	20.7	9.80
Q9V3W9	DNA repair protein Rad23 OS=Drosophila melanogaster GN=Rad23 PE=2 SV=1 - [Q9V3W9_DROME]	14.60	23.91	2	5	5	9	5.883E7	414	45.8	4.67
Q7K5K3	CG11876, isoform A OS=Drosophila melanogaster GN=CG11876 PE=2 SV=1 - [Q7K5K3_DROME]	14.58	10.14	1	3	3	11	6.147E7	365	39.3	7.80
Q9VCI7	CG10225 OS=Drosophila melanogaster GN=RanBP3 PE=2 SV=1 - [Q9VCI7_DROME]	14.54	9.58	1	3	3	6	5.839E7	449	47.5	4.59
Q8T4G5	CG6512-PA, isoform A OS=Drosophila melanogaster GN=CG6512 PE=2 SV=1 - [Q8T4G5_DROME]	14.48	9.69	2	5	5	7	5.430E7	826	89.6	8.95
Q9VYQ9	LD24662p OS=Drosophila melanogaster GN=Tango4 PE=2 SV=1 - [Q9VYQ9_DROME]	14.45	6.02	1	1	2	9	7.792E7	482	52.7	9.31
Q05783	High mobility group protein D OS=Drosophila melanogaster GN=HmgD PE=1 SV=1 - [HMGD_DROME]	14.30	30.36	1	3	4	5	1.032E8	112	12.4	9.20
Q9VDH8	Ribosomal protein S30, isoform A OS=Drosophila melanogaster GN=RpS30 PE=1 SV=1 - [Q9VDH8_DROME]	14.30	7.58	1	1	1	7	7.336E7	132	14.6	10.05
Q9VNF8	Sec23, isoform A OS=Drosophila melanogaster GN=Sec23 PE=4 SV=2 - [Q9VNF8_DROME]	14.28	9.18	1	4	4	6	3.917E7	773	86.7	6.79
P36951	Putative hydroxypyruvate isomerase OS=Drosophila melanogaster GN=Gip PE=1 SV=1 - [HYI_DROME]	14.22	16.29	1	3	3	8	4.266E7	264	29.1	6.54
Q9VA83	Ferritin OS=Drosophila melanogaster GN=Fer2LCH PE=2 SV=1 - [Q9VA83_DROME]	14.15	18.06	1	4	4	12	1.021E8	227	25.2	6.34
Q9VYW4	CG1703, isoform A OS=Drosophila melanogaster GN=CG1703 PE=2 SV=1 - [Q9VYW4_DROME]	14.10	6.77	1	3	3	7	4.629E7	901	101.5	5.82
Q9VEZ3	LP04448p OS=Drosophila melanogaster GN=msps PE=1 SV=3 - [Q9VEZ3_DROME]	14.08	4.46	2	6	6	10	6.415E7	2042	225.8	8.06
Q7K0Y1	Ribosome biogenesis protein BOP1 homolog OS=Drosophila melanogaster GN=CG5033 PE=2 SV=1 - [BOP1_DROME]	14.07	10.84	1	7	7	8	2.629E7	784	90.4	5.88
Q9V7N5	V-type proton ATPase subunit C OS=Drosophila melanogaster GN=Vha44 PE=2 SV=5 - [VATC_DROME]	14.06	3.59	1	3	3	6	2.782E7	836	92.3	7.56
Q07152	Inosine-5'-monophosphate dehydrogenase OS=Drosophila melanogaster GN=ras PE=1 SV=1 - [IMDH_DROME]	14.05	23.84	1	9	9	17	4.243E7	537	57.8	7.43

Accession	Description	Score	Coverage	# Proteins	# Unique Peptides	# Peptides	# PSMs	Area	# AAs	MW [kDa]	calc. pI
Q9VVA6	Nuclear migration protein NudC OS=Drosophila melanogaster GN=nudC PE=2 SV=2 - [Q9VVA6_DROME]	14.03	22.89	1	8	8	12	4.539E8	332	37.8	5.64
P21914	Succinate dehydrogenase [ubiquinone] iron-sulfur subunit, mitochondrial OS=Drosophila melanogaster GN=SdhB PE=2 SV=2 - [SDHB_DROME]	13.94	23.57	2	5	5	9	2.572E7	297	33.7	8.66
M9PIS3	6-phosphogluconate dehydrogenase, decarboxylating OS=Drosophila melanogaster GN=Pgd PE=3 SV=1 - [M9PIS3_DROME]	13.84	12.27	2	5	5	8	1.009E8	481	52.5	6.32
Q7K0D8	CG2158 OS=Drosophila melanogaster GN=Nup50 PE=2 SV=1 - [Q7K0D8_DROME]	13.81	13.83	1	6	6	7	3.137E7	564	59.4	8.43
Q9W5E4	FI21448p1 OS=Drosophila melanogaster GN=l(1)1Bi PE=2 SV=1 - [Q9W5E4_DROME]	13.76	5.30	1	4	4	6	1.663E7	1133	127.8	7.55
Q9VRL1	CG10640-PA, isoform A OS=Drosophila melanogaster GN=Uev1A PE=2 SV=1 - [Q9VRL1_DROME]	13.73	28.28	2	3	3	6	3.242E8	145	16.7	6.14
Q9VWG1	CG14207, isoform D OS=Drosophila melanogaster GN=HspB8 PE=2 SV=1 - [Q9VWG1_DROME]	13.69	48.63	3	5	5	7	3.184E7	183	20.8	5.94
C1C3E7	MIP09364p OS=Drosophila melanogaster GN=Zasp52 PE=2 SV=1 - [C1C3E7_DROME]	13.67	16.26	2	3	3	7	3.557E7	449	48.8	8.79
Q9W0L7	CG32479, isoform A OS=Drosophila melanogaster GN=CG13903 PE=2 SV=2 - [Q9W0L7_DROME]	13.67	3.30	2	4	4	6	1.678E7	1517	164.6	7.28
P23696	Serine/threonine-protein phosphatase PP2A OS=Drosophila melanogaster GN=mts PE=2 SV=1 - [PP2A_DROME]	13.45	18.45	1	3	3	7	1.027E7	309	35.4	5.34
P54611	V-type proton ATPase subunit E OS=Drosophila melanogaster GN=Vha26 PE=2 SV=1 - [VATE_DROME]	13.39	26.99	1	6	6	11	1.722E8	226	26.1	6.15
P18489	Synaptobrevin OS=Drosophila melanogaster GN=Syb PE=2 SV=3 - [SYB_DROME]	13.35	17.11	1	2	2	5	1.819E7	152	16.7	6.57
Q09332	UDP-glucose:glycoprotein glucosyltransferase OS=Drosophila melanogaster GN=Ugt PE=1 SV=2 - [UGGG_DROME]	13.35	6.91	1	7	7	9	3.565E7	1548	174.2	6.15
Q8IPP8	CG31548 OS=Drosophila melanogaster GN=CG31548 PE=3 SV=1 - [Q8IPP8_DROME]	13.32	25.78	1	4	6	8	4.331E7	256	27.1	8.72
Q7K485	CathD OS=Drosophila melanogaster GN=cathD PE=2 SV=1 - [Q7K485_DROME]	13.29	20.15	1	6	6	11	1.505E8	392	42.4	6.32
Q9VJD1	CG6453, isoform A OS=Drosophila melanogaster GN=CG6453 PE=2 SV=1 - [Q9VJD1_DROME]	13.27	13.50	1	4	4	11	5.063E7	548	61.5	4.46
Q7JVI3	Eukaryotic translation initiation factor 3 subunit M OS=Drosophila melanogaster GN=Tango7 PE=2 SV=1 - [EIF3M_DROME]	13.26	13.70	1	5	5	9	8.953E7	387	44.1	5.71
Q9VYV4	Amun, isoform A OS=Drosophila melanogaster GN=Amun PE=2 SV=1 - [Q9VYV4_DROME]	13.17	11.64	1	4	4	7	6.804E7	550	58.4	4.58
M9PET0	Glycerol 3 phosphate dehydrogenase, isoform E OS=Drosophila melanogaster GN=Gpdh PE=3 SV=1 - [M9PET0_DROME]	13.16	12.00	4	3	3	8	2.401E7	350	38.2	6.61
X2JAI4	Minichromosome maintenance 3, isoform B OS=Drosophila melanogaster GN=Mcm3 PE=3 SV=1 - [X2JAI4_DROME]	13.16	10.88	2	7	7	7	6.516E7	818	90.7	6.33
Q9V411	Probable nucleolar GTP-binding protein 1 OS=Drosophila melanogaster GN=CG8801 PE=2 SV=1 - [NOG1_DROME]	13.14	5.83	1	4	4	8	1.466E7	652	75.3	9.54
Q9VXQ0	CG8288-PA OS=Drosophila melanogaster GN=mRpL3 PE=4 SV=2 - [Q9VXQ0_DROME]	13.13	17.40	1	4	4	7	1.215E7	362	40.5	10.07
Q9VZU4	CG12079-PA OS=Drosophila melanogaster GN=NDUFS3 PE=2 SV=1 - [Q9VZU4_DROME]	13.07	26.42	1	6	6	10	3.897E7	265	30.0	7.84
Q7KN90	Cysteine--tRNA ligase, cytoplasmic OS=Drosophila melanogaster GN=Aats-cys PE=1 SV=1 - [SYCC_DROME]	12.99	6.21	2	4	4	6	1.027E7	741	84.2	6.30
Q8IFW6	DEK protein OS=Drosophila melanogaster GN=Dek PE=2 SV=1 - [Q8IFW6_DROME]	12.93	10.78	4	4	4	4	4.027E7	612	65.9	4.83
Q9VCA5	Probable phenylalanine--tRNA ligase beta subunit OS=Drosophila melanogaster GN=CG5706 PE=2 SV=1 - [SYFB_DROME]	12.88	7.64	1	5	5	10	2.209E7	589	65.7	6.04
Q9VKZ8	Ubiquitin carboxyl-terminal hydrolase OS=Drosophila melanogaster GN=CG5384 PE=2 SV=1 - [Q9VKZ8_DROME]	12.84	16.21	1	7	7	9	6.420E7	475	53.7	6.25
Q9VB96	CG31075 OS=Drosophila melanogaster GN=CG31075 PE=3 SV=2 - [Q9VB96_DROME]	12.84	10.72	1	3	3	5	1.497E7	485	52.6	6.67
Q9VTE5	Probable prefoldin subunit 2 OS=Drosophila melanogaster GN=l(3)01239 PE=2 SV=1 - [PFD2_DROME]	12.81	36.36	1	5	5	8	6.179E7	143	16.2	5.64
Q24117	Dynein light chain 1, cytoplasmic OS=Drosophila melanogaster GN=ctp PE=1 SV=1 - [DYL1_DROME]	12.78	32.58	2	2	2	8	2.358E7	89	10.4	7.37
A4V193	Poly U binding factor 68kD, isoform D OS=Drosophila melanogaster GN=pUf68 PE=4 SV=1 - [A4V193_DROME]	12.77	17.80	2	4	4	6	3.436E7	545	58.0	5.30
Q9VXW3	CG12608 OS=Drosophila melanogaster GN=CG12608 PE=2 SV=2 - [Q9VXW3_DROME]	12.57	9.71	2	3	3	4	1.062E7	443	49.1	8.03
Q9VUJ1	Proteasome subunit beta type OS=Drosophila melanogaster GN=Prosbeta2 PE=2 SV=1 - [Q9VUJ1_DROME]	12.54	20.59	1	4	4	5	1.601E8	272	29.8	8.66
Q9W334	40S ribosomal protein S28 OS=Drosophila melanogaster GN=RpS28b PE=1 SV=2 - [RS28_DROME]	12.50	49.23	1	3	3	5	3.012E8	65	7.5	10.37
Q9V3I2	Drab5 OS=Drosophila melanogaster GN=Rab5 PE=2 SV=1 - [Q9V3I2_DROME]	12.46	15.53	1	2	2	7	2.585E7	219	23.9	8.41
Q9VXN4	Probable arginine--tRNA ligase, cytoplasmic OS=Drosophila melanogaster GN=Aats-arg PE=2 SV=1 - [SYRC_DROME]	12.45	14.59	1	8	8	8	3.714E7	665	75.5	7.44
O61345	Protein penguin OS=Drosophila melanogaster GN=pen PE=2 SV=1 - [PEN_DROME]	12.43	6.78	1	4	4	5	5.404E7	737	81.3	8.94
Q9VK59	CG5787, isoform A OS=Drosophila melanogaster GN=CG5787 PE=2 SV=2 - [Q9VK59_DROME]	12.42	11.26	1	6	6	6	2.051E7	950	100.2	9.74
Q9VZL1	LP07226p OS=Drosophila melanogaster GN=mge PE=2 SV=1 - [Q9VZL1_DROME]	12.41	52.03	3	4	4	6	1.489E8	148	16.1	4.60
P25823	Maternal protein tudor OS=Drosophila melanogaster GN=tud PE=1 SV=2 - [TUD_DROME]	12.34	1.71	1	3	3	5	1.477E7	2515	285.1	6.46
Q0E940	Eukaryotic translation initiation factor 3 subunit B OS=Drosophila melanogaster GN=eIF3-S9 PE=1 SV=1 - [EIF3B_DROME]	12.31	8.84	1	6	6	10	7.452E7	690	80.4	6.28
P13008	40S ribosomal protein S26 OS=Drosophila melanogaster GN=RpS26 PE=1 SV=1 - [RS26_DROME]	12.30	37.72	1	4	4	10	8.002E8	114	13.3	11.15
Q7K4Q5	Probable protein phosphatase CG10417 OS=Drosophila melanogaster GN=CG10417 PE=1 SV=1 - [Y0417_DROME]	12.20	5.59	1	3	3	4	2.047E7	662	72.3	4.58
Q9VY87	Cathepsin B1, isoform A OS=Drosophila melanogaster GN=CtsB1 PE=2 SV=1 - [Q9VY87_DROME]	12.18	18.24	1	5	5	9	6.193E7	340	37.4	6.61
Q9VXK6	Eukaryotic translation initiation factor 5 OS=Drosophila melanogaster GN=eIF5 PE=1 SV=1 - [IF5_DROME]	12.15	9.27	1	3	3	5	7.530E7	464	51.7	5.26
Q9V8R9	Protein 4.1 homolog OS=Drosophila melanogaster GN=cora PE=1 SV=1 - [41_DROME]	11.93	4.12	1	5	5	5	2.223E7	1698	184.1	8.70
A1Z8P9	Nucleoprotein TPR OS=Drosophila melanogaster GN=Mtor PE=1 SV=1 - [TPR_DROME]	11.92	3.71	1	5	5	5	2.897E7	2346	262.2	5.10
A1Z8I8	CG42336, isoform E OS=Drosophila melanogaster GN=CG13211 PE=4 SV=2 - [A1Z8I8_DROME]	11.90	11.43	1	1	1	4	1.983E7	140	15.6	6.33
Q9VHX9	CD98 heavy chain OS=Drosophila melanogaster GN=CD98hc PE=2 SV=1 - [Q9VHX9_DROME]	11.87	5.49	1	2	2	5	1.012E8	565	62.6	5.69
Q9VD58	CG6439, isoform A OS=Drosophila melanogaster GN=CG6439 PE=2 SV=1 - [Q9VD58_DROME]	11.83	18.92	1	4	4	10	4.708E7	370	40.4	8.54

Accession	Description	Score	Coverage	# Proteins	# Unique Peptides	# Peptides	# PSMs	Area	# AAs	MW [kDa]	calc. pI
P62152	Calmodulin OS=Drosophila melanogaster GN=Cam PE=1 SV=2 - [CALM_DROME]	11.82	18.79	1	2	2	4	6.733E7	149	16.8	4.22
Q9V426	LD07162p OS=Drosophila melanogaster GN=vig PE=2 SV=1 - [Q9V426_DROME]	11.80	16.73	1	5	5	6	2.443E7	490	52.7	9.74
Q9VG16	Granny smith, isoform D OS=Drosophila melanogaster GN=grsm PE=4 SV=3 - [Q9VG16_DROME]	11.78	12.95	2	6	6	8	9.754E7	533	57.2	6.21
Q9V436	26S proteasome regulatory complex subunit p30 OS=Drosophila melanogaster GN=Rpn12 PE=2 SV=1 - [Q9V436_DROME]	11.74	10.98	1	2	2	5	1.677E7	264	30.2	6.06
P91926	AP-2 complex subunit alpha OS=Drosophila melanogaster GN=AP-2alpha PE=1 SV=1 - [AP2A_DROME]	11.69	7.55	1	5	5	6	3.429E7	940	105.6	7.18
A1ZB71	Glutathione S transferase E6 OS=Drosophila melanogaster GN=GstE6 PE=3 SV=1 - [A1ZB71_DROME]	11.64	20.72	1	4	4	6	1.563E8	222	25.0	6.23
Q9VXK7	CG9172, isoform A OS=Drosophila melanogaster GN=CG9172 PE=2 SV=1 - [Q9VXK7_DROME]	11.63	8.60	2	2	2	5	9.270E6	221	24.6	9.74
O15971	CG17060-PA OS=Drosophila melanogaster GN=Rab10 PE=2 SV=1 - [O15971_DROME]	11.61	16.67	22	1	3	6	3.530E7	204	23.3	8.05
Q9V3P3	LD45860p OS=Drosophila melanogaster GN=REG PE=2 SV=1 - [Q9V3P3_DROME]	11.56	28.57	1	6	6	9	5.872E7	245	28.1	6.05
Q9GQR5	RH02809p OS=Drosophila melanogaster GN=stai PE=2 SV=1 - [Q9GQR5_DROME]	11.53	18.29	4	5	5	6	5.180E7	257	29.6	8.25
Q8SWU7	Obg-like ATPase 1 OS=Drosophila melanogaster GN=CG1354 PE=1 SV=1 - [OLA1_DROME]	11.52	8.82	1	4	4	7	2.951E8	397	44.9	6.71
E1JIK9	Myofilin, isoform I OS=Drosophila melanogaster GN=Mf PE=4 SV=1 - [E1JIK9_DROME]	11.46	16.09	8	3	3	7	5.407E7	230	26.6	9.44
P20432	Glutathione S-transferase 1-1 OS=Drosophila melanogaster GN=GstD1 PE=1 SV=1 - [GSTT1_DROME]	11.45	25.84	6	7	8	9	5.148E8	209	23.9	7.23
Q9VEP3	CG4009 OS=Drosophila melanogaster GN=CG4009 PE=4 SV=1 - [Q9VEP3_DROME]	11.39	14.77	1	7	7	7	5.454E7	623	70.7	8.02
Q9VQ29	Cytochrome b-c1 complex subunit Rieske, mitochondrial OS=Drosophila melanogaster GN=RFeSP PE=2 SV=3 - [Q9VQ29_DROME]	11.38	10.87	1	2	2	5	2.716E8	230	24.9	8.68
Q9VSU6	Dihydropteridine reductase OS=Drosophila melanogaster GN=Dhpr PE=2 SV=1 - [Q9VSU6_DROME]	11.37	16.60	1	3	3	4	3.785E7	235	24.5	6.34
Q8STG9	DSec61alpha OS=Drosophila melanogaster GN=Sec61alpha PE=2 SV=1 - [Q8STG9_DROME]	11.36	7.77	1	4	4	7	3.545E8	476	52.2	8.24
Q9VTF9	Ubiquitin fusion degradation protein 1 homolog OS=Drosophila melanogaster GN=Ufd1-like PE=2 SV=1 - [UFD1_DROME]	11.32	12.66	1	2	2	4	2.856E7	316	34.8	6.25
M9PD65	Ran GTPase activating protein, isoform C OS=Drosophila melanogaster GN=RanGAP PE=4 SV=1 - [M9PD65_DROME]	11.29	12.07	2	5	5	8	4.609E7	588	65.1	4.65
Q9VIK1	CG9318, isoform A OS=Drosophila melanogaster GN=CG9318 PE=2 SV=1 - [Q9VIK1_DROME]	11.27	5.77	1	2	2	6	4.382E7	659	74.6	7.77
Q9V3V9	Endonuclease G inhibitor, isoform A OS=Drosophila melanogaster GN=EndoGI PE=1 SV=1 - [Q9V3V9_DROME]	11.26	12.81	1	3	3	6	1.867E7	359	40.6	4.60
Q7JWF1	CG12140 OS=Drosophila melanogaster GN=Etf-QO PE=2 SV=1 - [Q7JWF1_DROME]	11.19	11.09	1	5	5	9	2.461E7	604	65.9	6.64
Q9W2D9	Eukaryotic translation initiation factor 3 subunit K OS=Drosophila melanogaster GN=CG10306 PE=2 SV=1 - [EIF3K_DROME]	11.17	19.37	1	3	3	7	1.669E8	222	25.6	6.43
Q24276	Hsp90 co-chaperone Cdc37 OS=Drosophila melanogaster GN=Cdc37 PE=1 SV=1 - [CDC37_DROME]	11.10	15.42	1	4	4	6	3.325E7	389	45.1	5.06
Q9XZ63	Mesencephalic astrocyte-derived neurotrophic factor homolog OS=Drosophila melanogaster GN=Manf PE=1 SV=2 - [ARMET_DROME]	11.07	8.67	1	1	1	6	1.525E7	173	20.1	8.24
Q9VSC5	CG8209 OS=Drosophila melanogaster GN=CG8209 PE=2 SV=1 - [Q9VSC5_DROME]	11.07	11.48	1	2	2	4	1.782E7	331	36.7	6.10
Q9VGP4	LP08082p OS=Drosophila melanogaster GN=Ranbp9 PE=2 SV=1 - [Q9VGP4_DROME]	11.05	4.52	1	4	4	6	2.231E7	1018	114.3	4.87
Q9VAM6	CDGSH iron-sulfur domain-containing protein 2 homolog OS=Drosophila melanogaster GN=CG1458 PE=2 SV=1 - [CISD2_DROME]	11.02	35.34	1	5	5	8	8.670E7	133	14.6	8.46
Q02645	Protein hu-li tai shao OS=Drosophila melanogaster GN=hts PE=1 SV=2 - [HTS_DROME]	11.02	7.09	1	6	6	8	3.058E7	1156	127.9	6.39
Q7K581	CG8963, isoform A OS=Drosophila melanogaster GN=CG8963 PE=2 SV=1 - [Q7K581_DROME]	11.00	10.55	1	4	4	5	5.957E7	559	63.2	6.23
Q04448	Bifunctional methylenetetrahydrofolate dehydrogenase/cyclohydrolase, mitochondrial OS=Drosophila melanogaster GN=Nmdmc PE=2 SV=2 - [MTDC_DROME]	11.00	10.36	1	2	2	4	3.067E7	309	33.5	7.52
Q8INH5	CG32473, isoform B OS=Drosophila melanogaster GN=CG32473-RB PE=2 SV=1 - [Q8INH5_DROME]	11.00	6.76	4	5	5	8	1.120E8	903	102.6	5.12
Q9VSS1	Pixie, isoform A OS=Drosophila melanogaster GN=pix PE=2 SV=1 - [Q9VSS1_DROME]	10.97	5.89	1	4	4	4	4.385E7	611	69.3	8.13
P45437	Coatomer subunit beta OS=Drosophila melanogaster GN=betaCop PE=2 SV=2 - [COPB_DROME]	10.94	7.05	1	4	4	6	2.545E7	964	107.3	6.32
Q9VG69	CG5844 OS=Drosophila melanogaster GN=CG5844 PE=2 SV=1 - [Q9VG69_DROME]	10.84	13.23	1	3	3	4	7.102E7	378	41.6	5.68
Q26454	DNA replication licensing factor MCM4 OS=Drosophila melanogaster GN=dpa PE=1 SV=2 - [MCM4_DROME]	10.77	13.16	1	7	7	9	4.091E7	866	96.5	7.52
O62621	Coatomer subunit beta' OS=Drosophila melanogaster GN=beta'Cop PE=2 SV=2 - [COPB2_DROME]	10.76	4.27	1	3	3	6	4.194E7	914	102.6	5.26
Q9VJC7	Elongation factor Ts, mitochondrial OS=Drosophila melanogaster GN=CG6412 PE=2 SV=1 - [EFTS_DROME]	10.74	13.21	1	3	3	4	4.019E7	318	35.4	6.87
P43332	U1 small nuclear ribonucleoprotein A OS=Drosophila melanogaster GN=snf PE=1 SV=1 - [SNRPA_DROME]	10.71	10.19	1	3	3	5	2.669E7	216	24.5	9.64
P02299	Histone H3 OS=Drosophila melanogaster GN=His3 PE=1 SV=4 - [H3_DROME]	10.68	16.91	2	3	3	5	1.275E9	136	15.4	11.27
Q9VJZ6	CG6523-PA OS=Drosophila melanogaster GN=CG6523 PE=2 SV=1 - [Q9VJZ6_DROME]	10.67	14.81	1	2	2	5	2.458E7	216	23.6	4.86
Q9VZQ8	Glutathione peroxidase OS=Drosophila melanogaster GN=PHGPx PE=2 SV=1 - [Q9VZQ8_DROME]	10.56	18.93	3	3	3	6	5.064E7	169	18.7	7.83
Q9V6U9	Probable trans-2-enoyl-CoA reductase, mitochondrial OS=Drosophila melanogaster GN=CG16935 PE=3 SV=2 - [MECR_DROME]	10.46	19.89	1	6	6	10	3.707E7	357	39.1	9.11
Q8T0Q4	GH13992p OS=Drosophila melanogaster GN=shrb PE=2 SV=1 - [Q8T0Q4_DROME]	10.43	12.39	1	2	2	4	2.603E7	226	25.4	4.81
Q27237	Protein tumorous imaginal discs, mitochondrial OS=Drosophila melanogaster GN=l(2)tid PE=1 SV=2 - [TID_DROME]	10.34	14.42	2	4	4	5	1.285E8	520	56.1	9.10
Q9NHD5	Probable N-acetyltransferase san OS=Drosophila melanogaster GN=san PE=1 SV=1 - [SAN_DROME]	10.28	18.48	1	3	3	5	2.572E7	184	21.0	8.78
P91929	NADH dehydrogenase [ubiquinone] 1 alpha subcomplex subunit 10, mitochondrial OS=Drosophila melanogaster GN=ND42 PE=2 SV=2 - [NDUAA_DROME]	10.26	4.42	1	2	2	6	1.364E7	407	46.9	6.80
Q9W022	CG8993-PA OS=Drosophila melanogaster GN=CG8993 PE=2 SV=1 - [Q9W022_DROME]	10.25	16.20	1	2	2	4	8.770E7	142	15.8	8.46
P40945	ADP-ribosylation factor 2 OS=Drosophila melanogaster GN=Arf102F PE=2 SV=2 - [ARF2_DROME]	10.24	17.78	1	3	4	6	3.301E7	180	20.6	7.39
P23226	205 kDa microtubule-associated protein OS=Drosophila melanogaster GN=Map205 PE=1 SV=2 - [MA205_DROME]	10.23	5.49	2	4	4	4	4.668E7	1185	126.6	4.86

Accession	Description	Score	Coverage	# Proteins	# Unique Peptides	# Peptides	# PSMs	Area	# AAs	MW [kDa]	calc. pI
Q9VXF9	AT13091p OS=Drosophila melanogaster GN=rngo PE=2 SV=1 - [Q9VXF9_DROME]	10.22	12.23	1	4	4	4	8.695E6	458	50.5	5.16
Q9VDT5	Carboxypeptidase OS=Drosophila melanogaster GN=CG4572-RC PE=2 SV=1 - [Q9VDT5_DROME]	10.19	11.00	1	4	4	8	1.571E7	482	54.4	6.42
Q03427	Lamin-C OS=Drosophila melanogaster GN=LamC PE=1 SV=2 - [LAMC_DROME]	10.16	10.14	1	5	5	5	1.945E8	621	69.8	6.84
A4V3J6	Heterogeneous nuclear ribonucleoprotein at 98DE, isoform F OS=Drosophila melanogaster GN=Hrb98DE PE=4 SV=1 - [A4V3J6_DROME]	10.13	13.02	2	3	3	4	1.181E8	361	38.5	9.16
P48148	Ras-like GTP-binding protein Rho1 OS=Drosophila melanogaster GN=Rho1 PE=1 SV=1 - [RHO1_DROME]	10.11	18.23	1	3	3	5	7.430E7	192	21.7	7.01
Q8T079	Putative oxidoreductase GLYR1 homolog OS=Drosophila melanogaster GN=CG4747 PE=1 SV=1 - [GLYR1_DROME]	10.11	13.79	2	6	6	6	3.622E7	602	65.2	6.10
Q04047	Protein no-on-transient A OS=Drosophila melanogaster GN=nonA PE=1 SV=2 - [NONA_DROME]	10.01	7.86	2	3	3	4	1.912E7	700	76.9	9.35
Q0E8V7	CG34132 OS=Drosophila melanogaster GN=Tim13 PE=2 SV=1 - [Q0E8V7_DROME]	10.01	19.05	1	2	2	4	7.491E7	84	9.7	8.16
A8DYH2	Ubiquitin-fold modifier 1 OS=Drosophila melanogaster GN=CG34191 PE=3 SV=1 - [UFM1_DROME]	10.01	31.03	1	2	2	6	3.057E7	87	9.4	8.54
Q9V3J4	Sec13 OS=Drosophila melanogaster GN=Sec13 PE=4 SV=1 - [Q9V3J4_DROME]	10.00	9.55	1	3	3	6	4.727E7	356	39.5	6.25
A1Z909	CG8545 OS=Drosophila melanogaster GN=CG8545-RA PE=2 SV=1 - [A1Z909_DROME]	9.97	9.99	1	6	6	8	3.910E7	891	99.8	5.50
Q7KLX3	GM12291p OS=Drosophila melanogaster GN=Tapdelta PE=2 SV=1 - [Q7KLX3_DROME]	9.92	24.10	1	4	4	9	5.535E7	166	17.6	8.54
Q8IMI8	Myosin light chain 2, isoform B OS=Drosophila melanogaster GN=Mlc2 PE=4 SV=2 - [Q8IMI8_DROME]	9.89	31.34	2	3	3	9	7.278E7	201	21.5	4.74
Q7PLL6	CG17514, isoform A OS=Drosophila melanogaster GN=CG17514 PE=4 SV=2 - [Q7PLL6_DROME]	9.89	4.79	1	9	9	10	7.262E7	2630	293.7	7.03
Q9VX36	CG5703, isoform A OS=Drosophila melanogaster GN=CG5703 PE=4 SV=1 - [Q9VX36_DROME]	9.87	36.36	1	8	8	13	8.930E7	242	26.8	6.90
Q59DP8	Calcium-transporting ATPase OS=Drosophila melanogaster GN=PMCA PE=3 SV=3 - [Q59DP8_DROME]	9.87	4.91	7	3	3	3	2.965E7	1120	123.3	6.70
Q24253	Adaptor protein complex 1/2, beta subunit OS=Drosophila melanogaster GN=AP-1-2beta PE=2 SV=1 - [Q24253_DROME]	9.84	4.99	1	3	3	7	3.694E7	921	101.1	5.11
Q9VJG2	CG12288 OS=Drosophila melanogaster GN=CG12288 PE=4 SV=1 - [Q9VJG2_DROME]	9.83	6.21	1	2	2	4	2.825E7	435	47.9	10.04
Q9VPY2	Phospholipase A2 activator protein, isoform A OS=Drosophila melanogaster GN=Plap PE=2 SV=1 - [Q9VPY2_DROME]	9.82	4.96	1	3	3	5	2.694E7	787	85.7	5.83
Q9VEP6	Adenylosuccinate lyase OS=Drosophila melanogaster GN=AdSL PE=2 SV=1 - [Q9VEP6_DROME]	9.79	10.19	1	3	3	6	6.405E7	481	53.8	7.49
Q9W3N7	CG18624, isoform B OS=Drosophila melanogaster GN=CG18624-RA PE=2 SV=1 - [Q9W3N7_DROME]	9.72	26.79	1	2	2	3	3.499E7	56	6.2	9.70
P48810	Heterogeneous nuclear ribonucleoprotein 87F OS=Drosophila melanogaster GN=Hrb87F PE=2 SV=2 - [RB87F_DROME]	9.71	18.96	1	4	4	6	1.340E8	385	39.5	9.03
Q9VX77	CG5010-PA OS=Drosophila melanogaster GN=CG5010 PE=2 SV=1 - [Q9VX77_DROME]	9.70	7.06	1	1	1	3	1.887E8	170	17.3	8.87
Q9XZ61	Ubiquitin carboxyl-terminal hydrolase OS=Drosophila melanogaster GN=Uch-L5 PE=2 SV=1 - [Q9XZ61_DROME]	9.68	15.74	1	4	4	8	1.149E8	324	37.6	5.21
Q9VU36	LP04985p OS=Drosophila melanogaster GN=mRpL20 PE=2 SV=1 - [Q9VU36_DROME]	9.62	16.00	1	2	2	4	2.014E7	150	17.4	10.56
P11450	Follicle cell protein 3C-1 OS=Drosophila melanogaster GN=Fcp3C PE=2 SV=3 - [FCP3C_DROME]	9.62	5.32	1	1	1	3	2.345E7	282	30.3	7.08
Q7K0D3	CG12909 OS=Drosophila melanogaster GN=CG12909 PE=2 SV=1 - [Q7K0D3_DROME]	9.62	13.88	1	3	3	9	1.856E7	281	32.1	8.95
Q9VGQ1	CG5214 OS=Drosophila melanogaster GN=CG5214 PE=2 SV=1 - [Q9VGQ1_DROME]	9.56	12.61	1	4	4	6	1.171E8	468	49.9	9.47
Q9W1I7	CG5554 OS=Drosophila melanogaster GN=CG5554 PE=2 SV=1 - [Q9W1I7_DROME]	9.55	17.34	1	4	4	5	4.568E7	323	36.3	4.59
Q9VNA5	Proteasome subunit beta type-4 OS=Drosophila melanogaster GN=Prosbeta7 PE=2 SV=1 - [PSB4_DROME]	9.50	25.37	1	5	5	9	7.532E7	268	30.0	6.58
Q9W4M9	tRNA (cytosine(34)-C(5))-methyltransferase OS=Drosophila melanogaster GN=Nsun2 PE=2 SV=1 - [NSUN2_DROME]	9.47	13.27	1	6	6	7	1.257E7	746	84.1	6.83
Q9VJG0	Aminopeptidase P OS=Drosophila melanogaster GN=ApepP PE=2 SV=1 - [Q9VJG0_DROME]	9.44	20.55	1	6	6	8	4.572E7	613	68.5	5.95
Q7KMP8	26S proteasome regulatory complex subunit p39A OS=Drosophila melanogaster GN=Rpn9 PE=1 SV=1 - [Q7KMP8_DROME]	9.39	5.24	1	2	2	7	4.567E7	382	43.7	5.26
Q9VFP5	CG9925 OS=Drosophila melanogaster GN=CG9925 PE=2 SV=1 - [Q9VFP5_DROME]	9.39	7.17	1	4	4	6	3.048E7	892	99.9	8.54
Q9VFN9	CG9922 OS=Drosophila melanogaster GN=CG9922 PE=2 SV=1 - [Q9VFN9_DROME]	9.38	20.97	1	1	1	3	2.468E7	124	14.0	4.74
M9PJQ5	Wings up A, isoform K OS=Drosophila melanogaster GN=wupA PE=4 SV=1 - [M9PJQ5_DROME]	9.38	21.74	6	4	4	4	4.551E7	184	21.6	9.69
Q9W5C2	Translocating chain-associated membrane protein OS=Drosophila melanogaster GN=TRAM PE=2 SV=1 - [Q9W5C2_DROME]	9.37	4.35	1	1	1	3	1.358E7	368	41.8	9.57
A1ZAK9	P-element somatic inhibitor, isoform A OS=Drosophila melanogaster GN=Psi PE=4 SV=1 - [A1ZAK9_DROME]	9.36	7.40	3	4	4	4	2.628E7	716	73.4	7.96
Q8MRW1	CG40042 OS=Drosophila melanogaster GN=CG40042 PE=2 SV=1 - [Q8MRW1_DROME]	9.34	27.67	1	3	3	6	1.398E7	206	22.1	9.00
Q9V3E7	Aly OS=Drosophila melanogaster GN=Ref1 PE=2 SV=1 - [Q9V3E7_DROME]	9.34	15.79	1	4	4	6	3.599E7	266	27.8	10.55
Q0KHZ6	CG7834, isoform A OS=Drosophila melanogaster GN=CG7834 PE=4 SV=1 - [Q0KHZ6_DROME]	9.32	20.95	1	5	5	5	4.535E7	253	27.2	8.05
Q9VI10	Probable small nuclear ribonucleoprotein Sm D2 OS=Drosophila melanogaster GN=SmD2 PE=1 SV=1 - [SMD2_DROME]	9.27	40.34	1	4	4	5	1.612E8	119	13.5	9.95
O61613	Glycylpeptide N-tetradecanoyltransferase OS=Drosophila melanogaster GN=Nmt PE=2 SV=2 - [NMT_DROME]	9.23	10.81	1	4	4	7	2.799E7	472	53.8	8.44
A8JRB8	CG5028, isoform C OS=Drosophila melanogaster GN=CG5028-RC PE=2 SV=1 - [A8JRB8_DROME]	9.20	14.54	4	4	4	6	2.292E7	392	43.2	6.76
Q9VUY9	Phosphoglucomutase OS=Drosophila melanogaster GN=Pgm PE=1 SV=1 - [PGM_DROME]	9.13	5.89	1	3	3	4	3.173E7	560	60.7	6.70
Q7K110	CG6370 OS=Drosophila melanogaster GN=OstDelta PE=2 SV=1 - [Q7K110_DROME]	9.09	13.25	1	6	6	7	2.219E7	634	69.2	9.17
Q9VMW4	Reticulon-like protein OS=Drosophila melanogaster GN=Rtnl1 PE=2 SV=1 - [Q9VMW4_DROME]	9.06	19.82	5	1	4	5	2.113E7	222	24.7	9.19
Q8SWS3	CG13049 OS=Drosophila melanogaster GN=CG13049 PE=2 SV=1 - [Q8SWS3_DROME]	9.06	28.73	1	2	2	3	1.100E7	181	17.3	9.89
P06742	Myosin light chain alkali OS=Drosophila melanogaster GN=Mlc1 PE=1 SV=4 - [MLC1_DROME]	9.05	8.39	1	1	1	5	3.581E7	155	17.5	4.39
O46037	Vinculin OS=Drosophila melanogaster GN=Vinc PE=1 SV=1 - [VINC_DROME]	9.02	8.84	1	5	5	6	2.680E7	961	106.2	6.70

Accession	Description	Score	Coverage	# Proteins	# Unique Peptides	# Peptides	# PSMs	Area	# AAs	MW [kDa]	calc. pI
Q9VRJ4	CG10672 OS=Drosophila melanogaster GN=CG10672 PE=2 SV=1 - [Q9VRJ4_DROME]	8.96	15.46	1	4	4	6	2.177E7	317	33.6	9.33
Q9V3W2	GM23292p OS=Drosophila melanogaster GN=l(2)35Di PE=2 SV=1 - [Q9V3W2_DROME]	8.88	13.17	1	2	2	4	9.424E6	167	19.0	9.82
Q24439	ATP synthase subunit O, mitochondrial OS=Drosophila melanogaster GN=Oscp PE=2 SV=2 - [ATPO_DROME]	8.86	22.49	2	4	4	6	2.534E8	209	22.4	9.63
Q9VEX6	AAA family protein Bor OS=Drosophila melanogaster GN=bor PE=2 SV=2 - [Q9VEX6_DROME]	8.85	13.74	1	8	8	8	5.868E7	604	68.3	9.17
Q9VNX8	Eukaryotic translation initiation factor 2A OS=Drosophila melanogaster GN=CG7414 PE=1 SV=1 - [EIF2A_DROME]	8.84	6.27	1	2	2	3	2.622E7	638	70.0	9.10
Q9VH69	40S ribosomal protein S29 OS=Drosophila melanogaster GN=RpS29 PE=1 SV=1 - [RS29_DROME]	8.77	30.36	1	3	3	7	2.114E8	56	6.6	9.61
Q9VLT7	CG7424-PA OS=Drosophila melanogaster GN=RpL36A PE=1 SV=2 - [Q9VLT7_DROME]	8.76	19.23	1	2	2	4	3.922E8	104	12.5	10.80
Q9VS57	RE14391p OS=Drosophila melanogaster GN=Sec63 PE=2 SV=1 - [Q9VS57_DROME]	8.75	8.50	1	4	4	5	1.863E7	753	85.8	5.99
Q9V3S9	Very long-chain-fatty-acid--CoA ligase bubblegum OS=Drosophila melanogaster GN=bgm PE=2 SV=1 - [BGM_DROME]	8.71	14.71	1	6	6	7	2.735E8	666	73.6	8.24
P23380	V-type proton ATPase 16 kDa proteolipid subunit OS=Drosophila melanogaster GN=Vha16-1 PE=2 SV=1 - [VATL_DROME]	8.70	11.32	1	1	1	4	3.144E7	159	16.3	8.40
Q95SI7	CG6028 OS=Drosophila melanogaster GN=CG6028 PE=2 SV=1 - [Q95SI7_DROME]	8.69	19.11	1	3	3	5	1.053E8	293	32.3	6.57
Q0E8X6	eIF2B-delta, isoform B OS=Drosophila melanogaster GN=eIF2B-delta PE=3 SV=1 - [Q0E8X6_DROME]	8.69	11.63	2	4	4	4	2.055E7	576	61.8	9.25
Q8IN02	BcDNA.LD12153 OS=Drosophila melanogaster GN=Dph5 PE=2 SV=2 - [Q8IN02_DROME]	8.68	5.69	1	1	1	6	4.886E7	281	31.6	5.52
A9UN61	CG13114 OS=Drosophila melanogaster GN=CG13114-RB PE=2 SV=1 - [A9UN61_DROME]	8.66	9.15	1	3	3	4	6.380E7	426	45.9	5.74
Q9VEA1	Eukaryotic initiation factor 1A, isoform A OS=Drosophila melanogaster GN=eIF-1A PE=2 SV=1 - [Q9VEA1_DROME]	8.59	19.59	1	3	3	5	6.353E7	148	17.1	5.22
Q24400	Muscle LIM protein Mlp84B OS=Drosophila melanogaster GN=Mlp84B PE=1 SV=1 - [MLP2_DROME]	8.56	9.29	1	2	3	3	2.009E7	495	53.5	8.28
Q9VT61	CG8108, isoform A OS=Drosophila melanogaster GN=CG8108 PE=2 SV=1 - [Q9VT61_DROME]	8.53	8.81	1	6	6	7	2.518E7	919	102.9	6.21
Q8T6I0	EH domain containing protein OS=Drosophila melanogaster GN=Past1 PE=2 SV=1 - [Q8T6I0_DROME]	8.53	6.18	2	2	2	4	3.792E7	534	61.0	6.51
Q9VA18	Lethal (3) 03670 OS=Drosophila melanogaster GN=l(3)03670 PE=2 SV=1 - [Q9VA18_DROME]	8.52	27.80	1	4	4	6	4.637E7	223	24.2	8.97
Q9VQB4	CG3609, isoform B OS=Drosophila melanogaster GN=CG3609 PE=2 SV=1 - [Q9VQB4_DROME]	8.50	7.46	1	3	3	4	5.417E7	335	37.3	7.52
Q6IDF5	CG12859 OS=Drosophila melanogaster GN=CG12859 PE=2 SV=1 - [Q6IDF5_DROME]	8.46	24.78	1	2	2	4	5.588E7	113	13.1	9.82
Q9VAX8	CG4849 OS=Drosophila melanogaster GN=CG4849 PE=2 SV=1 - [Q9VAX8_DROME]	8.46	5.95	1	4	4	5	2.312E7	975	110.6	5.03
Q9V3A8	Ergic53, isoform A OS=Drosophila melanogaster GN=ergic53 PE=2 SV=1 - [Q9V3A8_DROME]	8.45	10.74	1	3	3	4	3.068E7	512	57.5	6.07
Q06943	High mobility group protein Z OS=Drosophila melanogaster GN=HmgZ PE=1 SV=1 - [HMGZ_DROME]	8.45	35.14	1	4	5	5	3.757E7	111	12.6	9.29
Q9XYU0	DNA replication licensing factor Mcm7 OS=Drosophila melanogaster GN=Mcm7 PE=1 SV=1 - [MCM7_DROME]	8.44	5.83	1	4	4	5	2.440E7	720	81.2	6.99
Q7KUA4	LD22577p OS=Drosophila melanogaster GN=Uba2 PE=2 SV=1 - [Q7KUA4_DROME]	8.43	10.86	1	5	5	9	3.473E7	700	77.6	5.02
P15348	DNA topoisomerase 2 OS=Drosophila melanogaster GN=Top2 PE=1 SV=1 - [TOP2_DROME]	8.41	6.01	1	6	6	8	2.532E7	1447	164.3	8.22
Q7KMM4	BcDNA.GH04962 OS=Drosophila melanogaster GN=BcDNA.GH04962 PE=2 SV=1 - [Q7KMM4_DROME]	8.40	6.28	1	3	3	6	4.407E7	924	105.7	6.51
M9ND61	Upstream of N-ras, isoform C OS=Drosophila melanogaster GN=Unr PE=4 SV=1 - [M9ND61_DROME]	8.40	2.62	3	2	2	3	4.119E6	1032	115.8	6.55
Q95SY7	LD39266p OS=Drosophila melanogaster GN=Sar1 PE=2 SV=1 - [Q95SY7_DROME]	8.35	28.39	2	3	3	4	1.066E8	155	17.5	5.92
P49963	Signal recognition particle 19 kDa protein OS=Drosophila melanogaster GN=Srp19 PE=2 SV=2 - [SRP19_DROME]	8.34	24.38	1	2	2	3	1.045E7	160	18.4	10.04
Q8IMV6	CG6995, isoform B OS=Drosophila melanogaster GN=Saf-B PE=4 SV=2 - [Q8IMV6_DROME]	8.34	4.74	3	3	3	3	1.945E7	928	102.0	7.47
Q7KSM5	Aos1 OS=Drosophila melanogaster GN=Aos1 PE=2 SV=1 - [Q7KSM5_DROME]	8.28	15.43	1	4	4	6	1.223E8	337	37.6	5.44
Q9VD02	CG7054 OS=Drosophila melanogaster GN=CG7054 PE=1 SV=1 - [Q9VD02_DROME]	8.24	13.41	1	2	2	3	1.120E8	179	19.8	5.21
Q9VKW8	CG17768, isoform B OS=Drosophila melanogaster GN=LSm-4-RB PE=1 SV=3 - [Q9VKW8_DROME]	8.22	24.68	1	3	3	5	2.388E7	154	16.9	10.05
Q7JVK8	CG5323 OS=Drosophila melanogaster GN=CG5323 PE=2 SV=1 - [Q7JVK8_DROME]	7.99	33.56	1	4	4	5	3.884E7	146	16.6	7.24
Q9VHN6	39S ribosomal protein L19, mitochondrial OS=Drosophila melanogaster GN=mRpL19 PE=2 SV=1 - [RM19_DROME]	7.98	8.17	1	2	2	4	1.608E7	306	36.2	8.82
Q9W5N2	60S ribosomal protein L38 OS=Drosophila melanogaster GN=RpL38 PE=1 SV=1 - [RL38_DROME]	7.98	25.71	1	2	2	5	8.356E8	70	8.2	10.29
O18335	Drab11 OS=Drosophila melanogaster GN=Rab11 PE=2 SV=1 - [O18335_DROME]	7.93	22.90	1	5	5	7	1.668E8	214	24.2	5.73
P54353	Putative peptidyl-prolyl cis-trans isomerase dodo OS=Drosophila melanogaster GN=dod PE=2 SV=3 - [DOD_DROME]	7.92	23.49	1	3	3	3	3.330E7	166	18.4	7.44
Q9W4K0	CHOp24, isoform A OS=Drosophila melanogaster GN=CHOp24 PE=2 SV=1 - [Q9W4K0_DROME]	7.90	24.04	1	5	5	6	7.408E7	208	23.2	5.76
P41043	Glutathione S-transferase S1 OS=Drosophila melanogaster GN=GstS1 PE=1 SV=2 - [GST1_DROME]	7.90	12.85	1	3	3	5	6.399E7	249	27.6	4.65
Q8T0J5	CG7675, isoform A OS=Drosophila melanogaster GN=CG7675 PE=2 SV=1 - [Q8T0J5_DROME]	7.87	13.94	2	3	3	4	6.995E7	287	31.4	9.32
P48604	GrpE protein homolog, mitochondrial OS=Drosophila melanogaster GN=Roe1 PE=2 SV=2 - [GRPE_DROME]	7.86	18.78	1	3	3	4	2.545E7	213	23.9	8.50
Q9VYY2	Signal peptidase complex subunit 2 OS=Drosophila melanogaster GN=Spase25 PE=2 SV=1 - [SPCS2_DROME]	7.83	14.57	1	3	3	4	5.684E7	199	22.2	8.78
P20028	DNA-directed RNA polymerase I subunit RPA2 OS=Drosophila melanogaster GN=RpI135 PE=2 SV=2 - [RPA2_DROME]	7.82	3.81	1	3	3	3	1.541E7	1129	128.4	8.41
O77134	9 kD basic protein OS=Drosophila melanogaster GN=c550 PE=2 SV=1 - [O77134_DROME]	7.70	24.69	1	2	2	3	3.296E8	81	9.0	10.07
Q9VEZ6	CG10407 OS=Drosophila melanogaster GN=CG10407 PE=4 SV=1 - [Q9VEZ6_DROME]	7.68	20.08	1	4	4	6	7.198E7	259	29.5	7.21
A4V4C1	Casein kinase II beta subunit, isoform C OS=Drosophila melanogaster GN=CkIIbeta PE=2 SV=1 - [A4V4C1_DROME]	7.68	20.00	5	3	3	4	8.534E7	215	24.8	5.55
Q9VXI6	Cytochrome b-c1 complex subunit 7 OS=Drosophila melanogaster GN=CG3560 PE=2 SV=1 - [Q9VXI6_DROME]	7.67	33.33	2	4	4	5	6.284E7	111	13.6	8.10

Accession	Description	Score	Coverage	# Proteins	# Unique Peptides	# Peptides	# PSMs	Area	# AAs	MW [kDa]	calc. pI
Q9VGZ3	Iron regulatory protein 1B OS=Drosophila melanogaster GN=Irp-1B PE=4 SV=1 - [Q9VGZ3_DROME]	7.67	7.68	1	5	5	6	8.280E6	899	98.5	5.95
Q9VRP3	AT08565p OS=Drosophila melanogaster GN=Txl PE=2 SV=1 - [Q9VRP3_DROME]	7.66	16.72	1	4	4	6	1.095E8	287	31.7	5.83
M9NFL1	eIF5B, isoform D OS=Drosophila melanogaster GN=eIF5B PE=4 SV=1 - [M9NFL1_DROME]	7.61	6.15	3	4	4	4	3.763E7	1090	121.0	5.50
Q9U4L6	Mitochondrial import receptor subunit TOM40 homolog 1 OS=Drosophila melanogaster GN=Tom40 PE=2 SV=2 - [TO401_DROME]	7.61	15.99	1	4	4	5	3.358E7	344	36.3	7.09
Q9W5A7	CG11381 OS=Drosophila melanogaster GN=CG11381-RA PE=2 SV=2 - [Q9W5A7_DROME]	7.56	11.70	1	3	3	5	1.503E7	436	49.9	6.73
P25867	Ubiquitin-conjugating enzyme E2-17 kDa OS=Drosophila melanogaster GN=eff PE=2 SV=1 - [UBCD1_DROME]	7.52	17.69	1	3	3	6	1.466E8	147	16.7	7.30
P26270	26S proteasome non-ATPase regulatory subunit 7 OS=Drosophila melanogaster GN=Rpn8 PE=1 SV=6 - [PSMD7_DROME]	7.52	23.37	1	5	5	8	6.087E7	338	38.1	6.71
Q9W1H5	Decapping protein 1, isoform A OS=Drosophila melanogaster GN=Dcp1 PE=1 SV=1 - [Q9W1H5_DROME]	7.43	10.75	1	3	3	5	9.425E7	372	41.3	7.17
Q9VBQ5	CG5112 OS=Drosophila melanogaster GN=CG5112 PE=2 SV=1 - [Q9VBQ5_DROME]	7.40	4.40	1	2	2	3	4.210E7	523	58.4	8.34
Q9W4J4	CG11444, isoform A OS=Drosophila melanogaster GN=CG11444 PE=2 SV=1 - [Q9W4J4_DROME]	7.25	8.37	1	1	1	2	4.146E8	215	22.9	9.38
Q9VB04	Barentsz, isoform A OS=Drosophila melanogaster GN=btz PE=2 SV=1 - [Q9VB04_DROME]	7.23	11.04	2	4	4	5	1.166E7	761	83.6	5.49
Q9VJJ0	Proteasome subunit beta type OS=Drosophila melanogaster GN=Prosbeta4 PE=1 SV=1 - [Q9VJJ0_DROME]	7.21	23.38	1	3	3	4	1.991E7	201	22.5	6.39
Q9W0M7	CG13900-PA, isoform A OS=Drosophila melanogaster GN=CG13900-RA PE=2 SV=2 - [Q9W0M7_DROME]	7.16	4.40	2	4	4	4	1.057E7	1227	136.5	5.47
Q9VMI5	CG9135 protein OS=Drosophila melanogaster GN=CG9135 PE=2 SV=1 - [Q9VMI5_DROME]	7.15	7.19	1	2	2	5	1.522E8	487	53.4	7.18
Q9VKJ3	Nuclear pore complex protein Nup160 homolog OS=Drosophila melanogaster GN=Nup160 PE=1 SV=1 - [NU160_DROME]	7.13	2.91	1	3	3	3	1.894E7	1411	160.2	5.36
Q9VQE0	Dynamin related protein 1, isoform A OS=Drosophila melanogaster GN=Drp1 PE=2 SV=1 - [Q9VQE0_DROME]	7.11	7.48	1	5	5	6	3.004E7	735	82.5	6.98
Q9VUH8	RE01471p OS=Drosophila melanogaster GN=Tdrd3 PE=2 SV=1 - [Q9VUH8_DROME]	7.05	4.67	1	2	2	3	4.001E6	836	91.7	9.58
Q9W4X7	Eukaryotic translation initiation factor 3 subunit G-1 OS=Drosophila melanogaster GN=eIF3-S4-1 PE=1 SV=1 - [EI3G1_DROME]	7.05	20.07	1	5	5	9	8.054E7	269	29.9	8.79
Q9VSC3	Ribonuclease X25 OS=Drosophila melanogaster GN=RNaseX25 PE=3 SV=1 - [Q9VSC3_DROME]	7.03	4.31	1	1	1	2	1.756E7	325	37.5	5.25
O97067	Probable peptidyl-tRNA hydrolase 2 OS=Drosophila melanogaster GN=CG1307 PE=2 SV=2 - [PTH2_DROME]	7.03	20.97	1	3	3	3	2.318E7	186	19.9	8.90
Q9Y134	L.2.35Df OS=Drosophila melanogaster GN=l(2)35Df PE=2 SV=1 - [Q9Y134_DROME]	7.01	2.94	1	3	3	3	1.176E7	1055	118.9	6.77
A1Z8D0	CG6751 OS=Drosophila melanogaster GN=nclb PE=4 SV=1 - [A1Z8D0_DROME]	6.94	16.12	1	4	4	4	1.858E7	459	51.4	4.39
A1Z9M2	CG8331 OS=Drosophila melanogaster GN=CG8331 PE=4 SV=1 - [A1Z9M2_DROME]	6.93	15.73	2	3	3	5	7.097E7	178	20.3	7.80
Q9VTF8	CG7636-PA OS=Drosophila melanogaster GN=mRpL2 PE=4 SV=1 - [Q9VTF8_DROME]	6.89	11.90	1	2	2	2	1.352E7	294	32.8	10.36
Q9W1F7	CG2970 OS=Drosophila melanogaster GN=CG2970 PE=2 SV=2 - [Q9W1F7_DROME]	6.89	10.38	1	3	3	4	2.553E7	366	40.5	8.84
Q8IQP2	Coat protein (Coatomer) zeta, isoform C OS=Drosophila melanogaster GN=zetaCOP PE=4 SV=1 - [Q8IQP2_DROME]	6.87	11.66	2	1	1	2	3.148E7	163	18.7	5.10
Q9W499	CG4111-PB, isoform B OS=Drosophila melanogaster GN=RpL35 PE=1 SV=2 - [Q9W499_DROME]	6.86	15.45	1	2	2	5	7.984E6	123	14.4	11.18
Q9VG32	Malic enzyme OS=Drosophila melanogaster GN=Men PE=2 SV=1 - [Q9VG32_DROME]	6.86	6.98	2	4	4	5	2.698E7	759	84.3	7.21
Q9VTB0	CG8003, isoform A OS=Drosophila melanogaster GN=CG8003 PE=2 SV=1 - [Q9VTB0_DROME]	6.78	11.79	1	3	3	4	1.118E7	407	45.7	7.62
Q9VMI3	CG9140, isoform B OS=Drosophila melanogaster GN=CG9140 PE=2 SV=1 - [Q9VMI3_DROME]	6.75	14.14	1	4	4	4	2.606E7	474	51.8	8.47
Q7KNF2	Polyadenylate-binding protein 2 OS=Drosophila melanogaster GN=Pabp2 PE=2 SV=1 - [PABP2_DROME]	6.75	13.39	1	2	2	3	1.520E7	224	25.0	5.20
Q9XYN7	Proteasome subunit beta type-3 OS=Drosophila melanogaster GN=Prosbeta3 PE=2 SV=1 - [PSB3_DROME]	6.74	23.41	1	4	4	6	1.103E8	205	23.2	5.44
Q9V9K7	Serrate RNA effector molecule homolog OS=Drosophila melanogaster GN=Ars2 PE=1 SV=2 - [SRRT_DROME]	6.74	7.10	1	5	5	5	2.733E7	943	107.2	5.55
Q9VHS2	Probable cytochrome c oxidase subunit 7A, mitochondrial OS=Drosophila melanogaster GN=CG9603 PE=1 SV=1 - [COX7A_DROME]	6.70	28.09	1	4	4	5	3.287E7	89	9.9	10.05
Q9VUL1	CTP synthase OS=Drosophila melanogaster GN=CTPsyn PE=1 SV=2 - [PYRG_DROME]	6.68	7.81	1	4	4	4	5.733E7	627	69.4	7.24
P42271	Tubulin gamma-2 chain OS=Drosophila melanogaster GN=gammaTub37C PE=2 SV=3 - [TBG2_DROME]	6.67	9.85	1	3	3	4	1.263E7	457	51.3	6.32
P41375	Eukaryotic translation initiation factor 2 subunit 2 OS=Drosophila melanogaster GN=eIF-2beta PE=1 SV=1 - [IF2B_DROME]	6.65	11.54	1	4	4	6	1.042E8	312	35.2	5.77
Q9Y0Y5	BcDNA.LD29885 OS=Drosophila melanogaster GN=epsilonCOP PE=2 SV=1 - [Q9Y0Y5_DROME]	6.65	4.90	1	1	1	3	1.373E7	306	34.6	4.87
P32748	Dihydroorotate dehydrogenase (quinone), mitochondrial OS=Drosophila melanogaster GN=Dhod PE=2 SV=2 - [PYRD_DROME]	6.64	7.41	1	2	2	4	2.769E7	405	44.3	9.25
Q7K0B6	CG30000 OS=Drosophila melanogaster GN=GstT1 PE=2 SV=1 - [Q7K0B6_DROME]	6.64	8.33	1	2	2	3	1.653E7	228	26.6	8.97
Q7KJ73	Tetraspanin OS=Drosophila melanogaster GN=Tsp42Ee PE=2 SV=1 - [Q7KJ73_DROME]	6.63	21.49	1	3	3	4	9.819E7	228	24.8	7.11
Q9VKC1	IP16805p OS=Drosophila melanogaster GN=RpL7-like PE=2 SV=2 - [Q9VKC1_DROME]	6.61	17.12	1	3	3	4	1.049E7	257	29.1	10.10
Q9VG79	Dipeptidase C OS=Drosophila melanogaster GN=Dip-C PE=2 SV=2 - [Q9VG79_DROME]	6.55	2.24	1	1	1	3	1.013E7	491	54.6	5.57
Q960Z0	Kinesin-like protein Klp10A OS=Drosophila melanogaster GN=Klp10A PE=1 SV=1 - [KI10A_DROME]	6.51	3.98	3	3	3	4	1.670E7	805	88.6	7.21
Q9W1Y1	ER membrane protein complex subunit 8/9 homolog OS=Drosophila melanogaster GN=CG3501 PE=1 SV=1 - [EMC89_DROME]	6.51	7.39	1	1	1	2	2.240E7	203	22.8	6.05
Q9VAD0	Acid phosphatase 1, isoform A OS=Drosophila melanogaster GN=Acph-1 PE=2 SV=1 - [Q9VAD0_DROME]	6.50	7.99	2	2	2	3	8.869E6	438	50.1	6.68
Q9VVA0	Cold shock domain-containing protein CG9705 OS=Drosophila melanogaster GN=CG9705 PE=1 SV=1 - [Y9705_DROME]	6.48	17.48	1	2	2	5	1.014E8	143	15.8	8.47
Q9VD51	Probable ATP-dependent RNA helicase pitchoune OS=Drosophila melanogaster GN=pit PE=2 SV=2 - [DDX18_DROME]	6.43	5.15	1	3	3	3	2.565E7	680	76.9	7.97
Q9VF27	GM02062p OS=Drosophila melanogaster GN=ND23 PE=2 SV=1 - [Q9VF27_DROME]	6.42	11.98	1	2	2	2	2.934E6	217	24.6	5.88
Q9VCC6	CG6178 OS=Drosophila melanogaster GN=CG6178 PE=2 SV=1 - [Q9VCC6_DROME]	6.41	6.62	1	3	3	3	2.690E7	544	59.9	8.10

Accession	Description	Score	Coverage	# Proteins	# Unique Peptides	# Peptides	# PSMs	Area	# AAs	MW [kDa]	calc. pI
Q9VVB8	CG32171-PA, isoform A OS=Drosophila melanogaster GN=Lmpt PE=2 SV=1 - [Q9VVB8_DROME]	6.41	12.65	7	2	2	4		245	27.7	8.24
Q9VZL3	Sc2 OS=Drosophila melanogaster GN=Sc2 PE=4 SV=1 - [Q9VZL3_DROME]	6.38	12.25	1	3	3	7	3.019E7	302	34.2	9.61
Q9VGA3	CG4115 OS=Drosophila melanogaster GN=CG4115 PE=2 SV=2 - [Q9VGA3_DROME]	6.38	8.64	1	1	1	2	9.680E6	220	25.3	7.58
Q7KRI2	Longitudinals lacking protein-like OS=Drosophila melanogaster GN=lolal PE=1 SV=1 - [LOLAL_DROME]	6.37	11.81	1	1	1	2	1.597E7	127	14.5	7.40
Q9VBH7	CG14544 OS=Drosophila melanogaster GN=CG14544 PE=2 SV=1 - [Q9VBH7_DROME]	6.34	4.42	1	1	1	3	1.341E7	317	35.1	7.03
A4UZR3	Trehalase OS=Drosophila melanogaster GN=Treh PE=3 SV=1 - [A4UZR3_DROME]	6.34	11.25	2	4	4	6	1.460E7	560	63.5	5.20
Q9VUZ0	Translocon-associated protein subunit beta OS=Drosophila melanogaster GN=SsRbeta PE=2 SV=2 - [Q9VUZ0_DROME]	6.32	19.47	1	2	2	8	1.371E8	190	21.2	8.72
A1Z6H7	Gp210 OS=Drosophila melanogaster GN=Gp210 PE=4 SV=1 - [A1Z6H7_DROME]	6.32	3.25	1	4	4	4	1.833E7	1876	209.7	6.47
A1Z934	CG8778 OS=Drosophila melanogaster GN=CG8778 PE=2 SV=1 - [A1Z934_DROME]	6.31	9.36	1	2	2	3	3.302E7	299	31.9	8.22
M9PFG7	Kinesin light chain, isoform C OS=Drosophila melanogaster GN=Klc PE=4 SV=1 - [M9PFG7_DROME]	6.30	4.34	2	2	2	3		507	57.9	6.16
O62602	CG12244-PA OS=Drosophila melanogaster GN=lic PE=2 SV=1 - [O62602_DROME]	6.30	3.89	1	1	1	4	2.830E7	334	38.2	6.39
M9PGX2	Pretaporter, isoform D OS=Drosophila melanogaster GN=prtp PE=4 SV=1 - [M9PGX2_DROME]	6.29	11.90	2	4	4	4	4.410E7	353	39.8	5.43
Q8IRH7	CG9148-PB, isoform B OS=Drosophila melanogaster GN=scf PE=4 SV=1 - [Q8IRH7_DROME]	6.28	8.33	2	1	1	2		192	22.3	4.50
Q9VH81	GH12714p OS=Drosophila melanogaster GN=PpD3 PE=2 SV=1 - [Q9VH81_DROME]	6.28	12.31	1	5	5	6	3.878E7	520	59.2	6.23
Q9VFW4	MIP09035p OS=Drosophila melanogaster GN=poly PE=2 SV=1 - [Q9VFW4_DROME]	6.28	11.55	1	2	2	4	9.826E6	251	27.9	6.90
Q9VQ62	Protein NPC2 homolog OS=Drosophila melanogaster GN=Npc2a PE=1 SV=1 - [NPC2_DROME]	6.28	20.95	1	2	2	3	8.469E7	148	16.0	5.31
P33438	Glutactin OS=Drosophila melanogaster GN=Glt PE=1 SV=2 - [GLT_DROME]	6.23	9.45	1	6	6	8	6.464E7	1026	118.7	4.77
Q7JVK1	HL07956p OS=Drosophila melanogaster GN=mRpL18 PE=2 SV=1 - [Q7JVK1_DROME]	6.23	26.34	1	3	3	3	1.734E7	186	21.2	9.29
Q9VL00	Ubiquitin thioesterase otubain-like OS=Drosophila melanogaster GN=CG4968 PE=2 SV=1 - [OTUBL_DROME]	6.15	11.07	1	2	2	2	4.219E7	262	30.4	4.98
P55035	26S proteasome non-ATPase regulatory subunit 4 OS=Drosophila melanogaster GN=Rpn10 PE=1 SV=2 - [PSMD4_DROME]	6.14	12.63	1	3	3	7	8.361E7	396	42.6	4.83
Q9VY91	LD21074p OS=Drosophila melanogaster GN=Pdcd4 PE=2 SV=2 - [Q9VY91_DROME]	6.12	6.88	1	1	1	3		509	56.3	5.97
Q9VPR5	CG2807, isoform A OS=Drosophila melanogaster GN=CG2807 PE=4 SV=2 - [Q9VPR5_DROME]	6.11	2.54	1	2	2	3		1340	149.5	6.67
Q8IRD0	CG32267 OS=Drosophila melanogaster GN=CG32267 PE=2 SV=1 - [Q8IRD0_DROME]	6.06	28.57	1	1	1	2	5.492E7	49	5.5	9.98
Q24046	Sodium/potassium-transporting ATPase subunit beta-1 OS=Drosophila melanogaster GN=nrv1 PE=1 SV=2 - [ATPB1_DROME]	6.06	9.39	1	4	4	4	6.591E7	309	35.3	6.38
Q9VW56	Probable prefoldin subunit 6 OS=Drosophila melanogaster GN=CG7770 PE=2 SV=1 - [PFD6_DROME]	6.05	21.60	1	3	3	4	1.751E7	125	14.4	8.51
X2JEK3	Wispy, isoform B OS=Drosophila melanogaster GN=wisp PE=4 SV=1 - [X2JEK3_DROME]	6.05	2.26	2	2	2	2	4.979E6	1372	151.1	8.70
Q9VVH5	AT13736p OS=Drosophila melanogaster GN=CG7580 PE=2 SV=1 - [Q9VVH5_DROME]	6.03	40.45	1	5	5	5	1.270E8	89	10.1	10.08
Q0E8X7	CG30415, isoform A OS=Drosophila melanogaster GN=CG30415 PE=4 SV=1 - [Q0E8X7_DROME]	6.02	25.61	1	2	2	3	1.237E7	82	9.5	9.67
Q9VDS6	Surfeit locus protein 6 homolog OS=Drosophila melanogaster GN=Surf6 PE=1 SV=1 - [SURF6_DROME]	5.98	4.94	1	1	1	3	1.514E7	324	38.1	9.85
O62530	AP-50, isoform A OS=Drosophila melanogaster GN=AP-2mu PE=2 SV=1 - [O62530_DROME]	5.97	6.41	1	2	2	3	1.186E7	437	49.8	9.48
Q94901	RNA-binding protein lark OS=Drosophila melanogaster GN=lark PE=1 SV=1 - [LARK_DROME]	5.94	7.39	1	2	2	3	7.522E7	352	39.9	9.07
Q6NP72	CG13220 OS=Drosophila melanogaster GN=CG13220 PE=2 SV=1 - [Q6NP72_DROME]	5.94	22.88	1	2	2	4	1.440E7	153	16.4	9.44
Q6AWN0	1,2-dihydroxy-3-keto-5-methylthiopentene dioxygenase OS=Drosophila melanogaster GN=CG32068 PE=2 SV=2 - [MTND_DROME]	5.90	8.60	1	1	1	2	1.318E7	186	22.2	5.35
Q9VD66	Female-specific independent of transformer OS=Drosophila melanogaster GN=fit PE=2 SV=1 - [Q9VD66_DROME]	5.89	19.83	1	2	2	4	1.066E8	121	13.9	7.58
Q9VVH3	CG7603-PA OS=Drosophila melanogaster GN=CG7603 PE=2 SV=1 - [Q9VVH3_DROME]	5.88	16.39	1	2	2	3	1.186E7	122	13.7	9.33
Q7JVY0	Coronin OS=Drosophila melanogaster GN=coro PE=2 SV=1 - [Q7JVY0_DROME]	5.87	4.55	1	2	2	2	3.075E7	528	57.2	6.38
M9PBM2	Encore, isoform G OS=Drosophila melanogaster GN=enc PE=4 SV=1 - [M9PBM2_DROME]	5.84	1.95	5	1	1	1	1.461E7	1798	187.7	8.37
Q9VES1	ARL-6-interacting protein 1 homolog OS=Drosophila melanogaster GN=CG10326 PE=2 SV=1 - [AR6P1_DROME]	5.83	6.09	1	1	1	3		197	22.2	8.84
P51406	Bystin OS=Drosophila melanogaster GN=bys PE=1 SV=2 - [BYS_DROME]	5.81	8.94	1	3	3	3	5.223E6	436	49.9	8.46
Q9VV74	Survival motor neuron protein OS=Drosophila melanogaster GN=Smn PE=1 SV=1 - [SMN_DROME]	5.81	4.87	1	1	1	2	1.536E7	226	24.6	5.29
Q9V464	Histone chaperone asf1 OS=Drosophila melanogaster GN=asf1 PE=1 SV=1 - [ASF1_DROME]	5.79	11.93	1	2	2	2	3.899E7	218	24.4	4.60
Q9VY28	Probable 28S ribosomal protein S25, mitochondrial OS=Drosophila melanogaster GN=mRpS25 PE=2 SV=1 - [RT25_DROME]	5.76	11.98	1	2	2	2	6.101E6	167	19.1	9.11
Q9VUX1	Mitochondrial ribosomal protein S31 OS=Drosophila melanogaster GN=mRpS31 PE=2 SV=1 - [Q9VUX1_DROME]	5.70	18.62	1	4	4	5	8.748E6	376	42.4	7.18
A4V2S3	C-terminal binding protein, isoform B OS=Drosophila melanogaster GN=CtBP PE=3 SV=1 - [A4V2S3_DROME]	5.64	9.84	2	3	3	6	4.873E7	386	42.2	6.98
Q9Y1A3	Mitochondrial import inner membrane translocase subunit Tim8 OS=Drosophila melanogaster GN=Tim8 PE=3 SV=1 - [TIM8_DROME]	5.64	17.05	1	1	1	2	1.048E8	88	10.1	5.16
P40792	Ras-related protein Rac1 OS=Drosophila melanogaster GN=Rac1 PE=1 SV=2 - [RAC1_DROME]	5.62	15.10	2	2	3	3	9.895E7	192	21.3	8.34
Q9V3E9	FI17138p1 OS=Drosophila melanogaster GN=spag PE=2 SV=1 - [Q9V3E9_DROME]	5.60	5.99	1	2	2	2	3.674E7	534	59.6	8.34
Q9VN39	CG9775, isoform A OS=Drosophila melanogaster GN=CG9775 PE=2 SV=1 - [Q9VN39_DROME]	5.60	6.41	2	2	2	2	7.649E6	421	46.1	12.48
Q9V595	Uroporphyrinogen decarboxylase OS=Drosophila melanogaster GN=Updo PE=3 SV=1 - [DCUP_DROME]	5.60	5.06	2	1	1	2	1.265E7	356	40.0	6.54
Q9VCY1	CG6937 OS=Drosophila melanogaster GN=CG6937 PE=4 SV=1 - [Q9VCY1_DROME]	5.59	11.24	1	4	4	4	1.002E8	356	39.5	9.77

Accession	Description	Score	Coverage	# Proteins	# Unique Peptides	# Peptides	# PSMs	Area	# AAs	MW [kDa]	calc. pI
Q76NQ9	AMP deaminase, isoform F OS=Drosophila melanogaster GN=AMPdeam PE=4 SV=2 - [Q76NQ9_DROME]	5.58	5.24	9	2	2	3	1.626E7	649	75.7	6.42
Q24133	DnaJ protein homolog 1 OS=Drosophila melanogaster GN=DnaJ-1 PE=1 SV=3 - [DNAJ1_DROME]	5.57	9.88	1	2	2	2	1.421E7	334	37.0	8.85
Q9W3E7	Chorion protein c at 7F OS=Drosophila melanogaster GN=Cp7Fc PE=2 SV=2 - [Q9W3E7_DROME]	5.57	19.94	1	3	3	4	9.091E7	321	36.4	6.29
M9PBH8	CG2199, isoform C OS=Drosophila melanogaster GN=CG2199 PE=4 SV=1 - [M9PBH8_DROME]	5.57	4.14	2	2	2	2	1.180E7	724	80.9	8.59
P00967	Trifunctional purine biosynthetic protein adenosine-3 OS=Drosophila melanogaster GN=ade3 PE=1 SV=2 - [PUR2_DROME]	5.54	3.77	3	4	4	6	3.255E7	1353	144.4	7.37
Q9W3J5	Probable phenylalanine--tRNA ligase alpha subunit OS=Drosophila melanogaster GN=CG2263 PE=2 SV=1 - [SYFA_DROME]	5.54	5.82	1	3	3	4	7.847E7	498	55.6	6.76
Q9VD13	GH02671p OS=Drosophila melanogaster GN=lqfR PE=2 SV=4 - [Q9VD13_DROME]	5.53	5.39	2	2	2	2	1.974E7	649	69.3	5.30
P40423	Myosin regulatory light chain sqh OS=Drosophila melanogaster GN=sqh PE=1 SV=1 - [SQH_DROME]	5.52	6.32	1	1	1	3	7.463E6	174	19.9	4.81
Q9VIF6	CG9253 OS=Drosophila melanogaster GN=CG9253-RA PE=2 SV=1 - [Q9VIF6_DROME]	5.51	5.72	1	2	2	3	2.741E7	507	56.5	6.81
Q9V4S8	COP9 signalosome complex subunit 7 OS=Drosophila melanogaster GN=CSN7 PE=1 SV=2 - [CSN7_DROME]	5.50	14.03	1	2	2	3		278	31.0	6.70
Q9VB64	CG31063 OS=Drosophila melanogaster GN=CG31063 PE=4 SV=2 - [Q9VB64_DROME]	5.47	3.71	1	1	1	2		404	44.4	4.87
Q9V429	Thioredoxin-2 OS=Drosophila melanogaster GN=Trx-2 PE=1 SV=2 - [THIO2_DROME]	5.46	12.28	1	2	2	2	2.543E8	114	12.7	5.03
Q7JWR9	Zinc finger CCCH domain-containing protein 15 homolog OS=Drosophila melanogaster GN=CG8635 PE=1 SV=1 - [ZC3HF_DROME]	5.45	6.68	1	3	3	3	9.241E7	404	45.3	5.55
O76454	Pterin-4-alpha-carbinolamine dehydratase OS=Drosophila melanogaster GN=Pcd PE=1 SV=2 - [PHS_DROME]	5.45	6.77	1	1	1	2	5.302E7	192	21.1	9.76
Q9VTW6	CG10418-PA OS=Drosophila melanogaster GN=CG10418 PE=4 SV=1 - [Q9VTW6_DROME]	5.43	20.00	1	1	1	4	1.244E7	95	10.7	6.52
Q9VGH5	AT27789p OS=Drosophila melanogaster GN=glo PE=2 SV=1 - [Q9VGH5_DROME]	5.40	5.29	1	2	2	6	2.600E7	586	61.4	6.04
Q9W3U9	CG14434, isoform B OS=Drosophila melanogaster GN=CG14434 PE=2 SV=1 - [Q9W3U9_DROME]	5.39	22.99	2	3	3	3	4.816E7	187	21.1	5.33
P20232	Transcription elongation factor S-II OS=Drosophila melanogaster GN=TfIIS PE=2 SV=1 - [TFS2_DROME]	5.39	11.82	1	2	2	2	1.006E7	313	34.3	9.06
B7Z0L1	Fasciclin 1, isoform E OS=Drosophila melanogaster GN=Fas1 PE=4 SV=1 - [B7Z0L1_DROME]	5.37	7.85	6	3	3	3	1.054E7	624	69.4	6.92
P40796	La protein homolog OS=Drosophila melanogaster GN=La PE=1 SV=2 - [LA_DROME]	5.37	21.54	2	7	7	7	1.193E8	390	44.9	7.58
P45889	Actin-related protein 1 OS=Drosophila melanogaster GN=Arp1 PE=2 SV=2 - [ACTZ_DROME]	5.37	13.03	1	3	3	6	6.740E7	376	42.7	7.30
Q9Y112	BcDNA.GH10614 OS=Drosophila melanogaster GN=BcDNA.GH10614 PE=2 SV=1 - [Q9Y112_DROME]	5.37	6.01	1	2	2	3	1.590E7	316	36.3	7.08
Q9Y0Y2	Adenylosuccinate synthetase OS=Drosophila melanogaster GN=AdSS PE=2 SV=1 - [PURA_DROME]	5.35	12.98	1	4	4	4	4.153E7	447	48.9	6.84
Q9GNH8	Hexokinase A, isoform B OS=Drosophila melanogaster GN=Hex-A PE=3 SV=1 - [Q9GNH8_DROME]	5.34	3.79	2	1	1	2	1.509E7	448	49.9	5.81
Q9VEQ1	CG17556 OS=Drosophila melanogaster GN=CG17556-RA PE=2 SV=1 - [Q9VEQ1_DROME]	5.34	4.61	2	1	1	2	1.273E7	282	32.7	7.05
Q9VKD3	Probable cysteine desulfurase, mitochondrial OS=Drosophila melanogaster GN=CG12264 PE=2 SV=1 - [NFS1_DROME]	5.34	5.19	1	3	3	4	2.503E6	462	51.0	8.21
Q9XYZ5	DNA damage-binding protein 1 OS=Drosophila melanogaster GN=pic PE=1 SV=1 - [DDB1_DROME]	5.32	1.23	1	1	1	2	4.561E7	1140	126.0	5.36
Q9VTU2	CG9762-PA OS=Drosophila melanogaster GN=l(3)neo18 PE=2 SV=1 - [Q9VTU2_DROME]	5.31	13.98	1	2	2	2	2.043E7	186	22.0	8.51
Q9VDI1	LD46328p OS=Drosophila melanogaster GN=Synd PE=1 SV=2 - [Q9VDI1_DROME]	5.26	8.50	1	3	3	3	9.073E6	494	55.9	5.63
Q9VZ58	Purple acid phosphatase OS=Drosophila melanogaster GN=CG1637 PE=2 SV=1 - [Q9VZ58_DROME]	5.24	5.11	1	2	2	2	3.764E7	450	51.8	5.67
Q9W141	Putative ATP synthase subunit f, mitochondrial OS=Drosophila melanogaster GN=CG4692 PE=1 SV=1 - [ATPK_DROME]	5.23	20.56	1	2	2	2	2.740E8	107	12.5	10.10
Q9V535	RNA-binding protein 8A OS=Drosophila melanogaster GN=tsu PE=1 SV=1 - [RBM8A_DROME]	5.19	13.94	1	2	2	2	4.981E7	165	19.0	5.27
Q86BQ4	CG2862, isoform B OS=Drosophila melanogaster GN=CG2862 PE=4 SV=1 - [Q86BQ4_DROME]	5.19	18.25	2	2	2	4	2.385E8	126	13.8	7.24
Q6NL44	CG33722, isoform B OS=Drosophila melanogaster GN=CG31188 PE=2 SV=1 - [Q6NL44_DROME]	5.19	4.81	1	2	2	2	6.858E6	478	53.6	7.05
Q9XZ03	Multiprotein Bridging Factor 1 OS=Drosophila melanogaster GN=mbf1 PE=2 SV=1 - [Q9XZ03_DROME]	5.16	21.38	2	3	3	3	1.114E8	145	16.0	10.04
Q9W3C2	Uncharacterized protein CG1785 OS=Drosophila melanogaster GN=CG1785 PE=1 SV=1 - [GSCR2_DROME]	5.15	5.65	1	2	2	2	2.587E7	478	55.8	9.76
Q95RI2	CG10973 OS=Drosophila melanogaster GN=CG10973 PE=2 SV=1 - [Q95RI2_DROME]	5.13	8.50	1	2	2	2	7.942E6	306	34.0	5.34
A5XCL5	LD36265p (Fragment) OS=Drosophila melanogaster GN=UGP PE=2 SV=1 - [A5XCL5_DROME]	5.13	4.50	4	2	2	2	1.601E7	511	57.8	7.40
Q9TVM2	Exportin-1 OS=Drosophila melanogaster GN=emb PE=1 SV=1 - [XPO1_DROME]	5.12	5.55	1	4	4	6	1.253E7	1063	122.7	5.87
Q9VM71	5'-3' exoribonuclease 2 homolog OS=Drosophila melanogaster GN=Rat1 PE=1 SV=2 - [XRN2_DROME]	5.12	4.85	1	2	2	2		908	103.9	7.05
Q9VKI3	Vitelline membrane protein Vm32E OS=Drosophila melanogaster GN=Vm32E PE=1 SV=1 - [VTU4_DROME]	5.11	17.24	1	2	2	3	1.298E8	116	12.1	5.67
Q9I7K0	Microtubule-associated protein Jupiter OS=Drosophila melanogaster GN=Jupiter PE=1 SV=2 - [JUPIT_DROME]	5.08	23.08	1	3	3	4	7.956E7	208	22.3	10.14
Q9VEJ0	Peroxiredoxin 5037 OS=Drosophila melanogaster GN=Prx3 PE=2 SV=2 - [Q9VEJ0_DROME]	5.06	10.26	1	2	2	2	4.676E7	234	26.4	7.49
Q9VIM5	Actin-related protein 2/3 complex subunit 2 OS=Drosophila melanogaster GN=Arpc2 PE=2 SV=2 - [ARPC2_DROME]	5.06	8.64	1	2	2	2	1.909E7	301	35.1	7.91
Q9VMU0	CG8680, isoform A OS=Drosophila melanogaster GN=CG8680 PE=2 SV=1 - [Q9VMU0_DROME]	5.06	23.02	1	2	2	2	3.762E7	126	14.3	8.57
Q9VDE2	CG5793 OS=Drosophila melanogaster GN=CG5793 PE=4 SV=2 - [Q9VDE2_DROME]	5.04	10.53	1	2	2	2	8.601E6	228	24.6	6.54
Q9XZ19	CG3909 OS=Drosophila melanogaster GN=CG3909 PE=2 SV=1 - [Q9XZ19_DROME]	5.03	11.18	1	2	2	3	1.078E7	331	36.1	5.25
Q9VPQ2	CG4164 OS=Drosophila melanogaster GN=CG4164 PE=2 SV=1 - [Q9VPQ2_DROME]	5.03	12.43	1	3	3	4	8.851E6	354	40.2	5.67
Q9VP65	Multifunctional methyltransferase subunit TRM112-like protein OS=Drosophila melanogaster GN=CG12975 PE=2 SV=1 - [TR112_DROME]	5.03	11.29	1	1	1	2	7.880E6	124	13.9	4.59
Q7K0Q2	LD41918p OS=Drosophila melanogaster GN=Ranbp11 PE=2 SV=1 - [Q7K0Q2_DROME]	5.01	3.07	1	2	2	2	6.513E6	1075	122.0	5.59

Accession	Description	Score	Coverage	# Proteins	# Unique Peptides	# Peptides	# PSMs	Area	# AAs	MW [kDa]	calc. pI
Q9VQR9	CG17593, isoform A OS=Drosophila melanogaster GN=CG17593-RA PE=2 SV=1 - [Q9VQR9_DROME]	5.00	5.88	1	2	2	2	1.965E7	476	54.1	5.30
Q9V813	S-methyl-5'-thioadenosine phosphorylase OS=Drosophila melanogaster GN=Mtap PE=1 SV=1 - [MTAP_DROME]	5.00	8.65	1	2	2	2	3.918E7	289	32.0	6.43
Q9VAL7	Calnexin 99A, isoform A OS=Drosophila melanogaster GN=Cnx99A PE=2 SV=2 - [Q9VAL7_DROME]	4.99	1.98	2	1	1	2	3.607E7	605	68.0	4.84
Q7K1Q7	CG1381 OS=Drosophila melanogaster GN=RpLP0-like PE=2 SV=1 - [Q7K1Q7_DROME]	4.96	11.72	1	3	3	4	8.295E7	256	29.4	5.12
Q9VDY8	MIP08680p OS=Drosophila melanogaster GN=vib PE=2 SV=1 - [Q9VDY8_DROME]	4.95	8.82	1	2	2	2	5.809E7	272	31.3	6.06
Q9VQI7	CG3083-PA OS=Drosophila melanogaster GN=Prx6005 PE=4 SV=1 - [Q9VQI7_DROME]	4.93	13.51	1	2	2	2	1.857E7	222	24.8	5.36
X2J8I7	Sorting nexin 1, isoform B OS=Drosophila melanogaster GN=Snx1 PE=4 SV=1 - [X2J8I7_DROME]	4.93	8.33	2	3	3	4	3.196E7	456	51.8	5.27
Q9V470	LD44970p OS=Drosophila melanogaster GN=Sucb PE=2 SV=1 - [Q9V470_DROME]	4.92	12.74	1	4	4	4	1.672E7	416	45.0	6.01
Q9VP25	Esterase Q OS=Drosophila melanogaster GN=Est-Q PE=2 SV=1 - [Q9VP25_DROME]	4.91	8.41	1	1	2	5	4.460E8	559	61.4	5.59
Q9VXI3	CG9911, isoform C OS=Drosophila melanogaster GN=CG9911 PE=4 SV=1 - [Q9VXI3_DROME]	4.90	8.80	2	3	3	3	2.660E7	375	43.3	5.87
O97064	Ccp84Ag OS=Drosophila melanogaster GN=Ccp84Ag PE=4 SV=1 - [O97064_DROME]	4.89	21.47	1	2	2	2	8.358E6	191	19.4	5.48
P53034	Replication factor C subunit 2 OS=Drosophila melanogaster GN=RfC4 PE=2 SV=1 - [RFC2_DROME]	4.88	6.34	1	3	3	3	1.690E7	331	37.1	7.72
Q9VCH5	Nup98 OS=Drosophila melanogaster GN=Nup98-96 PE=4 SV=3 - [Q9VCH5_DROME]	4.88	2.55	1	3	3	3	7.760E6	1960	210.0	6.52
Q7JZM8	39S ribosomal protein L41, mitochondrial OS=Drosophila melanogaster GN=mRpL41 PE=2 SV=1 - [RM41_DROME]	4.86	12.65	1	1	1	1	2.091E7	166	18.6	9.39
M9MRR7	Sh3beta, isoform C OS=Drosophila melanogaster GN=Sh3beta PE=4 SV=1 - [M9MRR7_DROME]	4.86	21.23	4	2	2	2	4.264E7	146	16.2	4.50
Q9VSS2	Signal recognition particle subunit SRP68 OS=Drosophila melanogaster GN=Srp68 PE=2 SV=1 - [SRP68_DROME]	4.86	9.93	1	4	4	4	1.774E7	604	69.0	8.47
Q9VW26	Ornithine aminotransferase, mitochondrial OS=Drosophila melanogaster GN=Oat PE=2 SV=1 - [OAT_DROME]	4.85	6.50	1	3	3	3	6.661E6	431	47.3	7.05
Q9VVU5	COP9 signalosome complex subunit 1b OS=Drosophila melanogaster GN=CSN1b PE=2 SV=1 - [CSN1_DROME]	4.83	2.48	1	1	1	2	4.958E7	525	58.2	6.83
Q9VXX8	Probable 60S ribosomal protein L37-A OS=Drosophila melanogaster GN=RpL37a PE=1 SV=1 - [RL371_DROME]	4.82	26.88	2	3	3	4	2.695E8	93	10.6	11.69
Q95TK5	CG1635 OS=Drosophila melanogaster GN=CG1635 PE=2 SV=1 - [Q95TK5_DROME]	4.81	9.38	1	2	2	2		448	51.1	9.06
Q7K148	Proteasome subunit beta type OS=Drosophila melanogaster GN=Prosbeta5 PE=2 SV=1 - [Q7K148_DROME]	4.80	11.35	1	3	3	4	2.382E8	282	31.1	6.93
Q9VDI5	CG17271, isoform A OS=Drosophila melanogaster GN=CG17271 PE=4 SV=2 - [Q9VDI5_DROME]	4.80	13.88	2	3	3	3	6.383E6	281	32.5	6.37
Q058X8	Chorion protein b at 7F, isoform B OS=Drosophila melanogaster GN=Cp7Fb PE=2 SV=1 - [Q058X8_DROME]	4.78	29.50	2	3	3	3	2.173E7	139	15.1	8.91
Q9W2N0	F-actin-capping protein subunit alpha OS=Drosophila melanogaster GN=cpa PE=2 SV=1 - [CAPZA_DROME]	4.78	11.19	1	2	2	2	3.494E7	286	32.7	5.94
Q7KLW9	BcDNA.LD02793 OS=Drosophila melanogaster GN=Prp19 PE=2 SV=1 - [Q7KLW9_DROME]	4.77	9.70	1	4	4	4	2.002E7	505	55.2	6.90
Q7K550	Eukaryotic translation initiation factor 3 subunit J OS=Drosophila melanogaster GN=Adam PE=1 SV=1 - [EIF3J_DROME]	4.77	18.22	1	3	3	4	2.799E6	236	26.6	4.86
Q9V3W0	CG15261-PA OS=Drosophila melanogaster GN=UK114 PE=2 SV=1 - [Q9V3W0_DROME]	4.76	23.19	1	2	2	3	3.506E7	138	14.9	7.81
P25439	ATP-dependent helicase brm OS=Drosophila melanogaster GN=brm PE=1 SV=2 - [BRM_DROME]	4.75	1.28	3	2	2	2	9.332E6	1638	185.0	7.56
Q9VH64	CG8507 OS=Drosophila melanogaster GN=CG8507 PE=2 SV=1 - [Q9VH64_DROME]	4.74	4.75	1	2	2	3	9.877E7	379	44.6	8.15
Q9VHC8	CG8149 OS=Drosophila melanogaster GN=CG8149 PE=2 SV=1 - [Q9VHC8_DROME]	4.73	9.09	1	2	2	2	5.425E7	308	32.9	6.24
Q9W3X7	CG3192, isoform A OS=Drosophila melanogaster GN=CG3192-RA PE=2 SV=1 - [Q9W3X7_DROME]	4.73	16.57	1	2	2	2		175	20.2	8.09
Q9W4C3	Ubiquitin carboxyl-terminal hydrolase OS=Drosophila melanogaster GN=CG4165 PE=2 SV=1 - [Q9W4C3_DROME]	4.71	3.29	1	2	2	2	2.727E7	1126	121.4	6.58
Q9VAK1	Alphabet, isoform A OS=Drosophila melanogaster GN=alph PE=3 SV=1 - [Q9VAK1_DROME]	4.70	11.68	3	3	3	4	3.720E7	368	40.9	5.30
Q9V6Y3	Probable 28S ribosomal protein S16, mitochondrial OS=Drosophila melanogaster GN=mRpS16 PE=2 SV=1 - [RT16_DROME]	4.70	17.83	1	1	1	2	1.549E7	129	14.5	9.98
P12982	Serine/threonine-protein phosphatase alpha-2 isoform OS=Drosophila melanogaster GN=Pp1-87B PE=1 SV=1 - [PP12_DROME]	4.70	12.25	5	3	3	6	3.354E7	302	34.5	5.59
Q27601	Amidophosphoribosyltransferase OS=Drosophila melanogaster GN=Prat PE=1 SV=2 - [PUR1_DROME]	4.69	6.78	1	2	2	2	9.236E7	546	59.5	6.67
A1Z6S6	RE54322p OS=Drosophila melanogaster GN=vimar PE=2 SV=1 - [A1Z6S6_DROME]	4.67	4.73	2	1	2	2	2.291E7	634	70.2	5.16
Q7K3Z3	GH01724p OS=Drosophila melanogaster GN=p47 PE=2 SV=1 - [Q7K3Z3_DROME]	4.66	28.99	1	7	7	9	5.817E7	407	43.4	5.62
P32392	Actin-related protein 3 OS=Drosophila melanogaster GN=Arp3 PE=2 SV=3 - [ARP3_DROME]	4.63	5.74	1	2	2	2	5.460E7	418	47.0	5.99
Q9VU58	Neuropeptide-like 2 OS=Drosophila melanogaster GN=Nplp2 PE=1 SV=1 - [NPLP2_DROME]	4.62	13.95	1	1	1	2	3.592E7	86	9.4	5.40
P52486	Ubiquitin-conjugating enzyme E2-22 kDa OS=Drosophila melanogaster GN=UbcD4 PE=1 SV=2 - [UBCD4_DROME]	4.60	10.55	1	2	2	5	4.813E7	199	22.5	5.47
Q9VN93	Putative cysteine proteinase CG12163 OS=Drosophila melanogaster GN=CG12163 PE=2 SV=2 - [CPR1_DROME]	4.60	4.56	1	2	2	4	1.431E7	614	68.9	7.01
Q9VW13	CG9330 OS=Drosophila melanogaster GN=CG9330-RA PE=2 SV=1 - [Q9VW13_DROME]	4.60	3.39	1	2	2	2	1.296E7	708	79.4	6.30
Q9VI56	CG1943, isoform A OS=Drosophila melanogaster GN=CG1943 PE=2 SV=1 - [Q9VI56_DROME]	4.59	40.68	1	3	3	4	4.092E7	118	12.4	9.44
Q9VHA1	Spermidine synthase, isoform A OS=Drosophila melanogaster GN=SpdS PE=3 SV=1 - [Q9VHA1_DROME]	4.58	11.85	2	3	3	3	1.135E7	287	32.3	5.78
Q86BS3	Chromator, isoform A OS=Drosophila melanogaster GN=Chro PE=2 SV=1 - [Q86BS3_DROME]	4.57	3.24	1	2	2	2	1.529E7	926	101.0	7.17
Q9VMH2	CG5972-PA OS=Drosophila melanogaster GN=Arpc4 PE=1 SV=1 - [Q9VMH2_DROME]	4.54	6.55	1	1	1	2		168	19.6	8.46
P53997	Protein SET OS=Drosophila melanogaster GN=Set PE=1 SV=2 - [SET_DROME]	4.53	12.64	1	2	2	6	2.908E7	269	31.0	4.36
Q9VMQ7	Putative elongator complex protein 4 OS=Drosophila melanogaster GN=CG6907 PE=1 SV=1 - [ELP4_DROME]	4.52	11.44	1	3	3	3	1.207E7	437	48.7	5.88
Q9VBU6	CG11857, isoform A OS=Drosophila melanogaster GN=CG11857 PE=2 SV=1 - [Q9VBU6_DROME]	4.51	13.79	2	2	2	2	2.961E7	203	23.7	9.41

Accession	Description	Score	Coverage	# Proteins	# Unique Peptides	# Peptides	# PSMs	Area	# AAs	MW [kDa]	calc. pI
Q9VSI1	CG7182, isoform A OS=Drosophila melanogaster GN=CG7182 PE=2 SV=2 - [Q9VSI1_DROME]	4.51	7.02	1	2	2	3	2.877E7	513	56.8	5.33
O18388	Importin subunit beta OS=Drosophila melanogaster GN=Fs(2)Ket PE=2 SV=2 - [IMB_DROME]	4.49	3.28	1	2	2	4	4.620E7	884	98.6	5.03
Q9VVZ6	CG12519, isoform A OS=Drosophila melanogaster GN=CG18294 PE=2 SV=2 - [Q9VVZ6_DROME]	4.48	16.03	2	1	1	2		131	12.7	9.50
P54367	Casein kinase I isoform alpha OS=Drosophila melanogaster GN=CkIalpha PE=1 SV=1 - [KC1A_DROME]	4.46	13.06	1	4	4	4	4.714E7	337	39.5	9.38
Q9VL68	CG4598, isoform B OS=Drosophila melanogaster GN=CG4598 PE=2 SV=2 - [Q9VL68_DROME]	4.46	9.96	1	2	2	2	4.303E7	281	31.0	9.23
Q9XZ06	Nucleoporin 93kD-1 OS=Drosophila melanogaster GN=Nup93-1 PE=2 SV=1 - [Q9XZ06_DROME]	4.45	1.34	1	1	1	2	1.375E7	823	93.8	6.27
Q9VKC8	CG6756-PA, isoform A OS=Drosophila melanogaster GN=Tom70 PE=2 SV=2 - [Q9VKC8_DROME]	4.45	9.17	1	4	4	4	3.462E8	589	66.3	6.21
Q8MLS2	CG30410 OS=Drosophila melanogaster GN=Rpi PE=4 SV=3 - [Q8MLS2_DROME]	4.45	19.92	1	4	4	6	7.989E7	241	26.5	5.88
Q9VX08	CG6769, isoform A OS=Drosophila melanogaster GN=CG6769 PE=2 SV=1 - [Q9VX08_DROME]	4.45	9.05	1	3	3	4	1.897E7	409	47.0	6.44
Q9VXP3	GH05406p OS=Drosophila melanogaster GN=mRpS30 PE=2 SV=1 - [Q9VXP3_DROME]	4.40	5.21	1	2	2	2	7.952E7	557	65.1	8.47
Q9VCX3	Probable 39S ribosomal protein L45, mitochondrial OS=Drosophila melanogaster GN=mRpL45 PE=2 SV=1 - [RM45_DROME]	4.39	8.31	1	2	2	2	3.000E7	361	41.6	9.23
Q9V460	Transcription elongation factor SPT5 OS=Drosophila melanogaster GN=Spt5 PE=1 SV=1 - [SPT5H_DROME]	4.38	5.01	1	3	3	3		1078	119.4	6.25
Q9VYD7	Mitochondrial import inner membrane translocase subunit Tim9 OS=Drosophila melanogaster GN=Tim9a PE=3 SV=1 - [TIM9_DROME]	4.38	16.84	1	1	1	1	1.092E8	95	11.1	6.09
Q961V3	CG2246, isoform D OS=Drosophila melanogaster GN=CG2246 PE=2 SV=1 - [Q961V3_DROME]	4.36	11.17	4	3	3	3	2.190E7	367	40.5	7.52
Q8IQW2	CG14235-PB, isoform B OS=Drosophila melanogaster GN=CoVIb PE=2 SV=1 - [Q8IQW2_DROME]	4.36	37.66	2	3	3	8	5.816E7	77	9.3	8.47
Q9VCB9	CG5515, isoform A OS=Drosophila melanogaster GN=CG5515-RA PE=2 SV=1 - [Q9VCB9_DROME]	4.34	15.16	1	2	2	3		244	28.5	4.34
Q8I937	CG32409 OS=Drosophila melanogaster GN=CG32409 PE=4 SV=2 - [Q8I937_DROME]	4.34	12.61	1	3	3	3	4.437E6	349	39.4	9.92
Q9VCE3	Rox8, isoform B OS=Drosophila melanogaster GN=Rox8 PE=4 SV=1 - [Q9VCE3_DROME]	4.34	4.96	2	2	2	2	1.070E7	464	49.5	7.85
Q7KV27	CG1640, isoform A OS=Drosophila melanogaster GN=CG1640 PE=4 SV=1 - [Q7KV27_DROME]	4.33	3.87	2	2	2	3	5.208E7	568	63.1	8.94
A1Z892	LD11278p OS=Drosophila melanogaster GN=Prx2540-1 PE=2 SV=1 - [A1Z892_DROME]	4.32	29.55	3	4	4	4	1.571E7	220	24.8	6.43
Q9VHK6	CG9836 OS=Drosophila melanogaster GN=CG9836 PE=2 SV=1 - [Q9VHK6_DROME]	4.30	20.13	1	2	2	2	1.141E7	154	16.7	9.39
Q9W277	Vacuolar protein sorting-associated protein 35 OS=Drosophila melanogaster GN=Vps35 PE=2 SV=4 - [Q9W277_DROME]	4.29	4.62	2	3	3	3	2.206E7	822	93.1	5.47
Q7KKH3	Protein SDA1 homolog OS=Drosophila melanogaster GN=Mys45A PE=1 SV=1 - [SDA1_DROME]	4.27	5.34	1	3	3	3	1.360E7	712	81.9	6.76
Q9VHY6	CG2943 OS=Drosophila melanogaster GN=CG2943 PE=2 SV=1 - [Q9VHY6_DROME]	4.27	2.30	1	2	2	2		915	100.9	8.13
Q9VBP9	Nuclear protein localization protein 4 homolog OS=Drosophila melanogaster GN=Npl4 PE=1 SV=3 - [NPL4_DROME]	4.26	3.99	1	2	2	2	2.148E7	652	73.3	7.24
Q9VWB8	Nucleoporin 205kD, isoform A OS=Drosophila melanogaster GN=Nup205 PE=4 SV=2 - [Q9VWB8_DROME]	4.25	1.06	2	2	2	4	1.750E7	2067	232.6	6.67
M9PBQ4	Apoptosis inducing factor, isoform C OS=Drosophila melanogaster GN=AIF PE=4 SV=1 - [M9PBQ4_DROME]	4.23	3.12	2	2	2	2	3.856E7	738	81.1	6.20
P18431	Protein kinase shaggy OS=Drosophila melanogaster GN=sgg PE=1 SV=3 - [SGG_DROME]	4.22	8.17	8	3	3	4	2.095E7	514	53.8	8.75
Q9VN51	Transforming acidic coiled-coil protein, isoform B OS=Drosophila melanogaster GN=tacc PE=4 SV=2 - [Q9VN51_DROME]	4.22	5.04	5	4	4	5	2.645E7	1190	132.3	5.02
A1Z992	CG33138 OS=Drosophila melanogaster GN=AGBE PE=4 SV=1 - [A1Z992_DROME]	4.21	3.94	1	2	2	2	1.087E7	685	79.1	6.25
Q8IP62	Mitochondrial ribosomal protein S23 OS=Drosophila melanogaster GN=mRpS23 PE=4 SV=1 - [Q8IP62_DROME]	4.17	8.99	1	1	1	2	1.169E7	189	21.5	5.73
P54622	Single-stranded DNA-binding protein, mitochondrial OS=Drosophila melanogaster GN=mtSSB PE=1 SV=2 - [SSBP_DROME]	4.11	9.59	1	1	1	3	1.586E8	146	16.4	9.85
Q8SX57	CG31549 OS=Drosophila melanogaster GN=CG11005 PE=2 SV=1 - [Q8SX57_DROME]	4.10	10.12	1	2	3	4	1.843E7	257	26.9	7.09
Q9VE85	CG7671 OS=Drosophila melanogaster GN=Nup43 PE=4 SV=1 - [Q9VE85_DROME]	4.10	8.38	1	2	2	2		358	40.1	5.22
Q8IP18	RH52721p OS=Drosophila melanogaster GN=Tpr2 PE=2 SV=1 - [Q8IP18_DROME]	4.09	10.13	3	3	3	3	7.745E6	464	53.2	8.38
Q9V3F8	Pyrroline-5-carboxylate reductase OS=Drosophila melanogaster GN=P5cr PE=3 SV=1 - [Q9V3F8_DROME]	4.08	5.71	1	1	1	1	2.319E7	280	29.6	7.44
O44226	Elongin B OS=Drosophila melanogaster GN=EloB PE=1 SV=1 - [O44226_DROME]	4.08	15.25	1	2	2	2	4.249E7	118	13.2	4.50
Q9VL96	Pescadillo homolog OS=Drosophila melanogaster GN=CG4364 PE=1 SV=1 - [PESC_DROME]	4.07	3.35	1	2	2	2	4.518E7	627	73.8	7.08
Q5KTT4	NAT1, isoform C (Fragment) OS=Drosophila melanogaster GN=NAT1 PE=2 SV=1 - [Q5KTT4_DROME]	4.05	3.01	2	3	3	4	2.168E7	1526	166.8	8.81
Q9VDV2	AT06125p OS=Drosophila melanogaster GN=CG6195 PE=2 SV=1 - [Q9VDV2_DROME]	4.04	5.23	1	2	2	4	1.639E7	363	40.6	8.57
Q9V9U7	CG1890 OS=Drosophila melanogaster GN=CG1890 PE=2 SV=1 - [Q9V9U7_DROME]	4.04	14.55	1	1	1	1	1.545E7	110	12.9	5.71
Q9VV72	CG4123-PA, isoform A OS=Drosophila melanogaster GN=Mipp1 PE=2 SV=2 - [Q9VV72_DROME]	4.04	9.85	1	3	3	4	1.057E7	467	53.5	6.74
Q9W402	CG3446, isoform B OS=Drosophila melanogaster GN=CG3446 PE=2 SV=1 - [Q9W402_DROME]	4.01	17.53	1	2	2	4	1.551E7	154	17.7	9.39
Q9VL71	LD29830p OS=Drosophila melanogaster GN=Srp54 PE=2 SV=1 - [Q9VL71_DROME]	3.98	4.87	1	2	2	2	1.355E7	513	58.3	11.33
Q9VUZ8	CG13072-PA OS=Drosophila melanogaster GN=PDCD-5 PE=2 SV=1 - [Q9VUZ8_DROME]	3.96	18.05	1	2	2	2	3.553E7	133	15.1	5.36
Q9VDP9	CG4390, isoform A OS=Drosophila melanogaster GN=CG4390 PE=2 SV=1 - [Q9VDP9_DROME]	3.96	4.55	2	1	1	1	5.993E7	286	31.4	5.83
M9PBA6	CG5446, isoform B OS=Drosophila melanogaster GN=CG5446 PE=4 SV=1 - [M9PBA6_DROME]	3.94	22.50	2	1	1	1	1.809E7	80	9.1	4.26
Q8T060	CG12304, isoform B OS=Drosophila melanogaster GN=CG12304-RB PE=2 SV=3 - [Q8T060_DROME]	3.91	18.94	2	5	5	5	2.615E7	301	33.2	8.62
Q9VRV8	FI21453p1 OS=Drosophila melanogaster GN=Trn PE=2 SV=1 - [Q9VRV8_DROME]	3.88	2.35	1	2	2	3	7.326E6	893	101.5	5.07
A4V4U5	Sluggish A, isoform F OS=Drosophila melanogaster GN=slgA PE=4 SV=1 - [A4V4U5_DROME]	3.87	1.79	4	1	1	2		669	75.9	8.91

Accession	Description	Score	Coverage	# Proteins	# Unique Peptides	# Peptides	# PSMs	Area	# AAs	MW [kDa]	calc. pI
Q8ML70	Immune-induced peptides OS=Drosophila melanogaster GN=IM10 PE=1 SV=2 - [IM10_DROME]	3.87	6.72	1	1	1	1	1.054E7	268	29.1	10.92
Q9V431	Apoptosis inhibitor 5 homolog OS=Drosophila melanogaster GN=Aac11 PE=2 SV=1 - [API5_DROME]	3.86	4.48	1	2	2	2	6.198E7	536	59.9	6.76
Q9VI15	CG10286 OS=Drosophila melanogaster GN=CG10286 PE=2 SV=1 - [Q9VI15_DROME]	3.85	2.24	1	1	1	4	1.896E7	625	69.1	4.83
Q7KVX5	VAMP-associated protein of 33kDa ortholog A, isoform A OS=Drosophila melanogaster GN=Vap-33A PE=4 SV=1 - [Q7KVX5_DROME]	3.84	15.32	2	3	3	6	7.120E7	235	26.8	8.38
Q9W266	FI21560p1 OS=Drosophila melanogaster GN=wdp PE=2 SV=1 - [Q9W266_DROME]	3.84	2.81	1	1	1	1		677	74.7	5.39
Q9VG33	Sulfurtransferase OS=Drosophila melanogaster GN=CG12279 PE=2 SV=2 - [Q9VG33_DROME]	3.84	26.36	1	2	2	2	2.840E7	110	12.1	5.21
Q7K738	EG:52C10.4 protein OS=Drosophila melanogaster GN=Ubc10 PE=2 SV=1 - [Q7K738_DROME]	3.82	14.94	1	2	2	3	3.251E7	154	17.9	8.56
Q9VPE2	CG6020, isoform A OS=Drosophila melanogaster GN=CG6020-RA PE=2 SV=1 - [Q9VPE2_DROME]	3.80	9.86	1	4	4	5	3.748E7	416	46.8	9.39
Q8IPB9	CG31716-PG, isoform G OS=Drosophila melanogaster GN=Cnot4 PE=4 SV=1 - [Q8IPB9_DROME]	3.79	2.58	6	2	2	2	1.745E7	1047	116.1	6.67
Q5BI50	Cullin-4, isoform A OS=Drosophila melanogaster GN=Cul4 PE=2 SV=1 - [Q5BI50_DROME]	3.78	2.56	1	2	2	2	4.719E7	821	94.3	8.66
Q9U5L1	Signal recognition particle receptor subunit alpha homolog OS=Drosophila melanogaster GN=Gtp-bp PE=1 SV=2 - [SRPR_DROME]	3.78	3.58	1	2	2	2	2.386E7	614	67.8	6.83
Q9VF03	Brahma associated protein 155 kDa OS=Drosophila melanogaster GN=mor PE=2 SV=3 - [Q9VF03_DROME]	3.71	1.32	1	1	1	1	2.219E7	1209	131.3	5.73
Q9VPL0	CG3436, isoform A OS=Drosophila melanogaster GN=CG3436 PE=2 SV=1 - [Q9VPL0_DROME]	3.69	3.75	1	1	1	1	2.011E7	347	38.8	6.92
Q7KVQ7	Transport and golgi organization 5, isoform B OS=Drosophila melanogaster GN=Tango5 PE=4 SV=1 - [Q7KVQ7_DROME]	3.68	3.04	2	1	1	1	3.485E7	428	47.9	9.69
Q7KIN0	Toll-7 OS=Drosophila melanogaster GN=Toll-7 PE=2 SV=1 - [Q7KIN0_DROME]	3.67	2.63	1	1	1	1	1.980E7	1446	160.8	6.61
Q95RX5	CG11984, isoform D OS=Drosophila melanogaster GN=CG11984 PE=2 SV=1 - [Q95RX5_DROME]	3.66	1.84	1	1	1	1	1.132E7	599	62.4	5.72
Q9VXP4	Platelet-activating factor acetylhydrolase IB subunit beta homolog OS=Drosophila melanogaster GN=Paf-AHalpha PE=1 SV=1 - [PA1B2_DROME]	3.66	16.89	1	3	3	3	5.619E7	225	25.4	5.81
Q9VMS1	CG14028-PA OS=Drosophila melanogaster GN=cype PE=4 SV=3 - [Q9VMS1_DROME]	3.65	11.69	1	1	1	3	9.092E7	77	8.3	9.92
Q9VGK3	Peptidyl-prolyl cis-trans isomerase OS=Drosophila melanogaster GN=CG14715 PE=2 SV=1 - [Q9VGK3_DROME]	3.64	13.77	1	1	1	2	5.149E6	138	14.8	8.59
M9NF33	CG7185, isoform B OS=Drosophila melanogaster GN=CG7185 PE=4 SV=1 - [M9NF33_DROME]	3.63	2.48	2	1	1	1	5.622E6	605	66.1	9.50
O77430	HL01263p OS=Drosophila melanogaster GN=skpA PE=1 SV=1 - [O77430_DROME]	3.62	11.11	1	2	2	2	4.849E6	162	18.6	4.65
Q9VTB4	CG6463-PA OS=Drosophila melanogaster GN=CG6463 PE=2 SV=2 - [Q9VTB4_DROME]	3.62	10.48	1	1	1	2	9.023E6	124	13.8	9.39
Q24583	V-type proton ATPase subunit F 1 OS=Drosophila melanogaster GN=Vha14-1 PE=1 SV=1 - [VATF1_DROME]	3.61	8.06	1	1	1	2	1.052E7	124	13.8	6.38
Q9V6X7	GDP-fucose protein O-fucosyltransferase 1 OS=Drosophila melanogaster GN=O-fut1 PE=1 SV=1 - [OFUT1_DROME]	3.60	6.47	1	1	1	1	1.024E7	402	46.8	7.72
Q9V9S0	CG3305-PA OS=Drosophila melanogaster GN=Lamp1 PE=2 SV=3 - [Q9V9S0_DROME]	3.56	3.46	1	1	1	1	5.561E7	318	34.8	6.11
P26308	Guanine nucleotide-binding protein subunit beta-1 OS=Drosophila melanogaster GN=Gbeta13F PE=1 SV=1 - [GBB1_DROME]	3.54	15.29	1	2	2	2	8.701E6	340	37.1	6.44
Q8SYL1	Lethal (2) 09851 OS=Drosophila melanogaster GN=l(2)09851 PE=2 SV=1 - [Q8SYL1_DROME]	3.54	3.73	1	2	2	2	9.951E6	456	50.7	4.69
Q9V7D2	V-type proton ATPase subunit D 1 OS=Drosophila melanogaster GN=Vha36-1 PE=2 SV=1 - [VATD1_DROME]	3.53	16.26	1	2	2	2	3.849E7	246	27.6	9.54
Q9VKU3	CG6094 protein OS=Drosophila melanogaster GN=CG6094 PE=2 SV=1 - [Q9VKU3_DROME]	3.52	7.39	1	1	1	1	8.166E6	203	22.8	10.33
Q9VDE5	Protein Peter pan OS=Drosophila melanogaster GN=ppan PE=1 SV=1 - [PPAN_DROME]	3.51	1.52	1	1	1	2	2.231E6	460	53.1	9.76
Q9VLN6	CG7627, isoform B OS=Drosophila melanogaster GN=CG7627-RA PE=2 SV=2 - [Q9VLN6_DROME]	3.48	1.99	1	2	2	2		1355	152.2	7.34
Q8MSC4	CG3509 OS=Drosophila melanogaster GN=BigH1 PE=2 SV=1 - [Q8MSC4_DROME]	3.47	13.03	1	3	3	3	2.306E7	353	38.6	9.41
Q7JZK1	CG7712 OS=Drosophila melanogaster GN=CG7712 PE=2 SV=1 - [Q7JZK1_DROME]	3.47	10.48	1	1	1	2	3.666E7	124	14.9	9.50
Q9VEY5	CG5903 OS=Drosophila melanogaster GN=CG5903 PE=2 SV=1 - [Q9VEY5_DROME]	3.45	7.52	1	1	1	1	1.045E7	226	24.5	8.78
Q9VRX6	Innexin inx4 OS=Drosophila melanogaster GN=zpg PE=2 SV=1 - [INX4_DROME]	3.41	5.72	1	2	2	2	7.708E6	367	42.8	8.97
Q8SY77	CG3566-PC, isoform C OS=Drosophila melanogaster GN=CG3566 PE=2 SV=1 - [Q8SY77_DROME]	3.40	13.48	3	1	1	1	2.185E7	89	10.0	6.80
Q9XZS3	Translation machinery-associated protein 7 homolog OS=Drosophila melanogaster GN=CG13364 PE=4 SV=1 - [TMA7_DROME]	3.40	15.63	1	1	1	4	1.375E7	64	6.9	9.99
Q9VA73	Calcium-binding mitochondrial carrier protein Aralar1 OS=Drosophila melanogaster GN=aralar1 PE=2 SV=1 - [CMC_DROME]	3.39	5.04	1	3	3	4	3.889E7	695	76.7	8.72
E8NH77	SD15168p OS=Drosophila melanogaster GN=sw-RA PE=2 SV=1 - [E8NH77_DROME]	3.38	3.79	10	2	2	2	2.267E7	634	70.2	5.38
Q9VV39	Mitochondrial ribosomal protein S34 OS=Drosophila melanogaster GN=mRpS34 PE=2 SV=2 - [Q9VV39_DROME]	3.37	7.98	1	1	1	2		188	21.6	9.52
Q9VZ34	CG2076, isoform A OS=Drosophila melanogaster GN=CG2076 PE=2 SV=1 - [Q9VZ34_DROME]	3.36	2.64	1	1	1	1	3.356E7	341	35.8	9.96
Q9VDZ4	CG42358 OS=Drosophila melanogaster GN=CG42358 PE=2 SV=1 - [Q9VDZ4_DROME]	3.35	3.00	1	1	1	1	1.122E7	433	49.2	8.78
Q9VN56	CG14655 OS=Drosophila melanogaster GN=CG14655 PE=2 SV=1 - [Q9VN56_DROME]	3.33	8.57	1	1	1	1		525	58.0	8.29
Q9VGZ2	FI06805p OS=Drosophila melanogaster GN=Rrp46 PE=2 SV=3 - [Q9VGZ2_DROME]	3.33	7.73	1	1	1	1	6.067E6	233	25.7	5.33
Q9VZD8	THUMP domain-containing protein 1 homolog OS=Drosophila melanogaster GN=CG15014 PE=1 SV=2 - [THUM1_DROME]	3.33	4.63	1	1	1	1		324	36.2	5.83
M9PBI9	Sallimus, isoform S OS=Drosophila melanogaster GN=sls PE=4 SV=1 - [M9PBI9_DROME]	3.33	2.29	12	2	2	3	1.118E8	###	1758.8	5.00
Q9VB23	BcDNA.LD34343 OS=Drosophila melanogaster GN=wdb PE=2 SV=2 - [Q9VB23_DROME]	3.31	7.44	8	3	3	3	2.294E7	524	59.7	7.49
Q9VUB8	CG6513-PA, isoform A OS=Drosophila melanogaster GN=endos PE=2 SV=1 - [Q9VUB8_DROME]	3.28	22.69	1	2	2	4	2.856E7	119	12.9	8.63
Q9VWS2	Nucleoporin NUP53 OS=Drosophila melanogaster GN=CG6540 PE=2 SV=1 - [Q9VWS2_DROME]	3.27	5.74	1	1	1	1		331	35.1	9.00
Q9VN95	Enolase-phosphatase E1 OS=Drosophila melanogaster GN=CG12173 PE=1 SV=2 - [ENOPH_DROME]	3.25	4.69	1	1	1	1	1.568E7	256	28.3	6.07

Accession	Description	Score	Coverage	# Proteins	# Unique Peptides	# Peptides	# PSMs	Area	# AAs	MW [kDa]	calc. pI
Q9W4D2	RNA-binding protein 4F OS=Drosophila melanogaster GN=Rnp4F PE=1 SV=1 - [RNP4F_DROME]	3.24	1.91	2	2	2	2	1.320E7	941	106.7	5.11
Q7JQN4	LD15481p OS=Drosophila melanogaster GN=Rs1 PE=2 SV=1 - [Q7JQN4_DROME]	3.23	1.53	1	1	1	1		782	87.8	9.47
Q9VCA8	Ankyrin repeat and KH domain-containing protein mask OS=Drosophila melanogaster GN=mask PE=1 SV=2 - [ANKHM_DROME]	3.23	1.82	1	3	3	3		4001	422.9	5.68
Q94529	Probable pseudouridine-5'-monophosphatase OS=Drosophila melanogaster GN=Gs1l PE=2 SV=2 - [GS1_DROME]	3.22	5.63	1	1	1	1	4.018E7	231	25.8	6.00
P14199	Protein ref(2)P OS=Drosophila melanogaster GN=ref(2)P PE=1 SV=2 - [REF2P_DROME]	3.20	1.67	1	1	1	1	9.459E6	599	65.3	4.98
P11584	Integrin beta-PS OS=Drosophila melanogaster GN=mys PE=1 SV=3 - [ITBX_DROME]	3.19	1.89	2	1	1	1	7.058E6	846	92.6	5.97
Q9W4Z1	EG:BACH48C10.5 protein OS=Drosophila melanogaster GN=ptr PE=2 SV=2 - [Q9W4Z1_DROME]	3.18	1.86	2	1	1	1		1234	132.8	8.69
Q9VS52	AT11423p OS=Drosophila melanogaster GN=CG14834 PE=2 SV=1 - [Q9VS52_DROME]	3.18	3.44	1	1	1	1	1.341E8	407	46.0	10.24
Q9VK89	Probable tRNA (guanine(26)-N(2))-dimethyltransferase OS=Drosophila melanogaster GN=CG6388 PE=2 SV=1 - [TRM1_DROME]	3.18	1.90	1	1	1	1	1.665E7	578	64.9	7.21
Q9W124	CG4806 OS=Drosophila melanogaster GN=CG4806 PE=2 SV=2 - [Q9W124_DROME]	3.16	2.74	1	1	1	1	1.303E7	657	75.0	9.51
Q7JUY7	PTB domain-containing adapter protein ced-6 OS=Drosophila melanogaster GN=ced-6 PE=1 SV=1 - [CED6_DROME]	3.14	3.09	1	1	1	1	1.357E7	517	56.1	6.87
Q24311	Cullin homolog 1 OS=Drosophila melanogaster GN=lin19 PE=1 SV=2 - [CUL1_DROME]	3.10	1.94	1	1	1	1		774	89.5	7.90
Q9W255	GH05812p OS=Drosophila melanogaster GN=qkr58E-1 PE=2 SV=1 - [Q9W255_DROME]	3.09	6.31	1	1	1	1	7.270E6	396	45.4	9.07
Q9VGJ9	Heme oxygenase OS=Drosophila melanogaster GN=Ho PE=2 SV=1 - [Q9VGJ9_DROME]	3.07	6.76	1	1	1	1	9.031E6	296	34.1	5.80
P08266	DNA-directed RNA polymerase II subunit RPB2 OS=Drosophila melanogaster GN=RpII140 PE=2 SV=2 - [RPB2_DROME]	3.06	2.21	1	1	1	1		1176	134.0	7.05
Q9W1G0	Probable transaldolase OS=Drosophila melanogaster GN=Tal PE=2 SV=2 - [TALDO_DROME]	3.06	7.55	1	2	2	4	2.533E7	331	36.7	7.71
Q9V6B9	Probable nucleoporin Nup54 OS=Drosophila melanogaster GN=Nup54 PE=2 SV=1 - [NUP54_DROME]	3.04	1.64	1	1	1	1	4.062E7	610	64.4	8.59
Q9VIH1	CG9273 protein OS=Drosophila melanogaster GN=RPA2 PE=1 SV=2 - [Q9VIH1_DROME]	3.03	22.36	1	3	3	3	6.781E7	246	26.4	5.08
Q7K2D2	Probable dynactin subunit 2 OS=Drosophila melanogaster GN=Dmn PE=1 SV=1 - [DCTN2_DROME]	3.01	9.21	1	2	2	2	1.983E6	380	42.0	5.26
Q9VKK1	Enhancer of mRNA-decapping protein 4 homolog OS=Drosophila melanogaster GN=Ge-1 PE=1 SV=2 - [EDC4_DROME]	3.01	1.03	1	1	1	1		1354	149.2	5.90
Q9W415	tRNA (guanine-N(7)-)-methyltransferase non-catalytic subunit wuho OS=Drosophila melanogaster GN=wuho PE=2 SV=1 - [WUHO_DROME]	3.00	7.08	1	1	1	1	2.107E7	424	46.5	5.34
Q8IR93	CG1938-PB, isoform B OS=Drosophila melanogaster GN=Dlic PE=4 SV=1 - [Q8IR93_DROME]	3.00	8.79	2	2	2	2	1.916E7	398	44.2	6.13
X2JD07	Lethal (1) G0020, isoform B OS=Drosophila melanogaster GN=l(1)G0020 PE=4 SV=1 - [X2JD07_DROME]	2.98	2.69	2	2	2	2	1.212E7	967	108.3	8.13
Q9VMB6	CG31911-PA OS=Drosophila melanogaster GN=Ent2 PE=2 SV=1 - [Q9VMB6_DROME]	2.98	3.06	1	1	1	1	2.292E7	458	51.3	8.70
M9PDP6	CG11377, isoform B OS=Drosophila melanogaster GN=CG11377 PE=4 SV=1 - [M9PDP6_DROME]	2.97	6.75	2	1	1	1		311	34.2	5.48
Q9W021	39S ribosomal protein L23, mitochondrial OS=Drosophila melanogaster GN=mRpL23 PE=2 SV=1 - [RM23_DROME]	2.97	6.67	1	1	1	1	2.568E7	150	17.9	9.61
Q9VXM4	LD12946p OS=Drosophila melanogaster GN=MSBP PE=2 SV=1 - [Q9VXM4_DROME]	2.97	10.08	1	1	1	2		248	27.9	4.74
Q9V447	Krueppel homolog 2 OS=Drosophila melanogaster GN=Kr-h2 PE=1 SV=1 - [KRH2_DROME]	2.95	6.88	1	1	1	1	8.225E7	276	31.2	9.77
A8Y589	Apoptosis-linked gene-2 OS=Drosophila melanogaster GN=Alg-2 PE=4 SV=2 - [A8Y589_DROME]	2.95	6.74	1	1	1	1		178	20.8	5.58
Q9W2V9	CG2221-PA OS=Drosophila melanogaster GN=l(1)G0289 PE=2 SV=1 - [Q9W2V9_DROME]	2.92	2.40	1	1	1	1	1.553E7	625	67.2	7.02
P40797	Protein peanut OS=Drosophila melanogaster GN=pnut PE=1 SV=2 - [PNUT_DROME]	2.92	10.39	1	2	2	2	2.171E7	539	60.1	8.75
Q9VKW3	CG5313-PA OS=Drosophila melanogaster GN=RfC3 PE=2 SV=2 - [Q9VKW3_DROME]	2.91	3.61	1	1	1	1	1.275E7	332	37.4	7.20
Q9I7T7	La-related protein CG11505 OS=Drosophila melanogaster GN=CG11505 PE=1 SV=2 - [Y1505_DROME]	2.91	1.76	1	1	1	1	2.332E7	1531	161.9	8.75
Q9VHD3	Probable maleylacetoacetate isomerase 1 OS=Drosophila melanogaster GN=GstZ1 PE=1 SV=1 - [MAAI1_DROME]	2.87	6.50	1	1	1	1		246	27.9	8.90
Q9VEC8	CG7215, isoform A OS=Drosophila melanogaster GN=Prx5-RA PE=2 SV=1 - [Q9VEC8_DROME]	2.86	9.23	1	1	1	1		130	14.7	7.28
Q9VX91	E3 ubiquitin-protein ligase UBR1 OS=Drosophila melanogaster GN=UBR1 PE=2 SV=2 - [UBR1_DROME]	2.86	1.48	1	1	1	1	2.225E7	1824	208.2	6.43
Q9VE75	V-type proton ATPase subunit a OS=Drosophila melanogaster GN=Vha100-2 PE=2 SV=2 - [Q9VE75_DROME]	2.86	3.72	1	1	1	1	1.065E7	834	94.8	6.68
Q9V3T8	CG5442-PB, isoform B OS=Drosophila melanogaster GN=SC35 PE=2 SV=1 - [Q9V3T8_DROME]	2.84	5.64	1	1	1	1	8.914E6	195	21.4	11.75
O76752	CG12117-PA OS=Drosophila melanogaster GN=Sptr PE=2 SV=1 - [O76752_DROME]	2.84	5.75	1	1	1	1		261	29.2	7.03
Q9VHX4	CG2767, isoform A OS=Drosophila melanogaster GN=CG2767 PE=2 SV=1 - [Q9VHX4_DROME]	2.84	14.89	2	4	4	5	1.812E8	329	36.8	5.99
O97182	Actin related complex p41 subunit OS=Drosophila melanogaster GN=Arpc1 PE=2 SV=1 - [O97182_DROME]	2.83	5.31	1	1	1	1	3.680E7	377	41.6	8.35
Q9VIG1	CG12050 OS=Drosophila melanogaster GN=CG12050-RB PE=2 SV=2 - [Q9VIG1_DROME]	2.83	1.68	1	1	1	1		1071	119.9	8.72
Q9VL69	CG5885-PA OS=Drosophila melanogaster GN=BEST:CK01296 PE=2 SV=1 - [Q9VL69_DROME]	2.81	3.68	1	1	1	1	2.099E6	190	21.6	9.57
Q9VIV2	LD45403p OS=Drosophila melanogaster GN=swm PE=2 SV=1 - [Q9VIV2_DROME]	2.81	3.86	2	2	2	4	2.758E7	1062	115.6	9.26
Q9VZ64	Probable 6-phosphogluconolactonase OS=Drosophila melanogaster GN=CG17333 PE=2 SV=1 - [6PGL_DROME]	2.80	4.94	1	1	1	1		243	26.7	8.21
Q9VK57	LD15349p OS=Drosophila melanogaster GN=Pih1D1 PE=2 SV=1 - [Q9VK57_DROME]	2.80	8.36	2	1	1	1		335	38.0	7.84
Q9VL65	TbCMF46 OS=Drosophila melanogaster GN=TbCMF46 PE=4 SV=2 - [Q9VL65_DROME]	2.79	3.36	1	1	1	2	8.259E6	566	66.6	4.72
P54351	Vesicle-fusing ATPase 2 OS=Drosophila melanogaster GN=Nsf2 PE=2 SV=2 - [NSF2_DROME]	2.78	3.72	1	2	2	2	1.428E7	752	83.4	6.74
Q9W5W8	CG9577 OS=Drosophila melanogaster GN=CG9577 PE=2 SV=2 - [Q9W5W8_DROME]	2.78	4.17	1	1	1	1		312	33.8	7.46
A1ZBF1	CG18190 OS=Drosophila melanogaster GN=CG18190-RB PE=2 SV=2 - [A1ZBF1_DROME]	2.78	5.65	1	1	1	1	8.473E6	248	28.7	5.55

Accession	Description	Score	Coverage	# Proteins	# Unique Peptides	# Peptides	# PSMs	Area	# AAs	MW [kDa]	calc. pI
Q9VVN2	Probable 28S ribosomal protein S26, mitochondrial OS=Drosophila melanogaster GN=mRpS26 PE=2 SV=1 - [RT26_DROME]	2.77	12.89	1	2	2	2	1.553E7	225	26.2	8.41
Q9VY49	CG11674 OS=Drosophila melanogaster GN=CG11674 PE=2 SV=2 - [Q9VY49_DROME]	2.77	6.40	1	2	2	2	1.972E7	344	38.0	6.84
Q9VQQ0	Serine/threonine-protein phosphatase 2A activator OS=Drosophila melanogaster GN=Ptpa PE=2 SV=1 - [Q9VQQ0_DROME]	2.75	3.27	1	1	1	1	3.589E7	398	44.2	5.90
L7EGI4	Dead box protein 80, isoform F OS=Drosophila melanogaster GN=Dbp80 PE=4 SV=1 - [L7EGI4_DROME]	2.75	6.96	5	2	2	2	2.817E7	316	35.7	6.93
Q9VX44	Lethal (1) G0222, isoform F OS=Drosophila melanogaster GN=l(1)G0222 PE=4 SV=2 - [Q9VX44_DROME]	2.75	1.71	3	1	1	1	1.254E7	1110	123.0	8.25
Q8IH00	Nucleolar protein 6 OS=Drosophila melanogaster GN=Mat89Ba PE=1 SV=1 - [NOL6_DROME]	2.75	1.59	1	1	1	1	1.587E7	1193	136.3	9.00
Q9VLR5	RNA polymerase II transcriptional coactivator OS=Drosophila melanogaster GN=Ssb-c31a PE=2 SV=1 - [TCP4_DROME]	2.74	14.55	1	1	1	1	5.807E6	110	12.2	9.17
Q9VZE4	CG1316 OS=Drosophila melanogaster GN=CG1316 PE=2 SV=2 - [Q9VZE4_DROME]	2.74	2.34	1	1	1	1	8.017E6	470	52.4	6.71
Q9VUI8	CG5114, isoform A OS=Drosophila melanogaster GN=CG5114 PE=2 SV=1 - [Q9VUI8_DROME]	2.73	5.41	2	2	2	3	3.092E7	425	47.5	4.64
P04388	Ras-like protein 2 OS=Drosophila melanogaster GN=Ras64B PE=1 SV=2 - [RAS2_DROME]	2.73	7.81	1	1	1	1		192	22.2	6.77
Q9VZC8	CG15019, isoform B OS=Drosophila melanogaster GN=CG15019 PE=2 SV=1 - [Q9VZC8_DROME]	2.73	10.27	1	1	1	1	3.940E7	146	17.3	9.94
Q9V3J1	V-type proton ATPase subunit H OS=Drosophila melanogaster GN=VhaSFD PE=2 SV=2 - [VATH_DROME]	2.72	6.20	1	2	2	2	5.383E7	468	53.7	6.61
P36872	Protein phosphatase PP2A 55 kDa regulatory subunit OS=Drosophila melanogaster GN=tws PE=2 SV=1 - [2ABA_DROME]	2.72	7.41	1	3	3	6	3.131E7	499	56.9	7.09
Q9V3Y2	Anamorsin homolog OS=Drosophila melanogaster GN=CIAPIN1 PE=2 SV=1 - [DRE2_DROME]	2.71	12.10	1	2	2	2	4.337E7	248	27.1	5.21
Q8SXY6	Transmembrane emp24 domain-containing protein bai OS=Drosophila melanogaster GN=bai PE=2 SV=1 - [TMEDA_DROME]	2.69	4.85	1	1	1	1		206	23.7	8.22
P51592	E3 ubiquitin-protein ligase hyd OS=Drosophila melanogaster GN=hyd PE=1 SV=3 - [HYD_DROME]	2.68	0.73	1	1	1	1	1.525E7	2885	318.7	5.94
Q9VLJ9	39S ribosomal protein L51, mitochondrial OS=Drosophila melanogaster GN=mRpL51 PE=2 SV=2 - [RM51_DROME]	2.67	6.90	1	1	1	1		174	20.2	10.54
M9NE97	Copper transporter 1A, isoform C OS=Drosophila melanogaster GN=Ctr1A PE=4 SV=1 - [M9NE97_DROME]	2.66	8.71	1	1	1	1	2.141E7	241	26.8	6.70
Q9VYN1	Protein kinase C delta, isoform E OS=Drosophila melanogaster GN=Pkcdelta PE=4 SV=3 - [Q9VYN1_DROME]	2.66	1.21	1	1	1	1		1894	206.5	8.03
Q9VBI2	UMP-CMP kinase OS=Drosophila melanogaster GN=Dak1 PE=2 SV=1 - [Q9VBI2_DROME]	2.66	9.09	1	2	2	2	3.194E7	253	27.8	8.00
Q8IPJ7	CG11567-PB, isoform B OS=Drosophila melanogaster GN=Cpr PE=4 SV=1 - [Q8IPJ7_DROME]	2.65	2.18	2	1	1	1	1.323E7	550	62.8	8.24
M9PGG0	ADP-ribosylation factor GTPase activating protein 3, isoform E OS=Drosophila melanogaster GN=ArfGAP3 PE=4 SV=1 - [M9PGG0_DROME]	2.65	4.87	5	1	1	1	2.502E7	513	54.4	8.94
Q7KQM6	PERQ amino acid-rich with GYF domain-containing protein CG11148 OS=Drosophila melanogaster GN=CG11148 PE=1 SV=2 - [PERQ1_DROME]	2.64	1.08	1	1	1	1	9.430E6	1574	173.6	7.11
Q7K4B3	Probable elongator complex protein 2 OS=Drosophila melanogaster GN=Elp2 PE=1 SV=1 - [ELP2_DROME]	2.63	5.04	1	3	3	3	2.616E7	794	88.9	5.60
Q9VHR5	CG9684 OS=Drosophila melanogaster GN=CG9684 PE=4 SV=2 - [Q9VHR5_DROME]	2.63	4.36	1	2	2	2	4.803E7	642	71.9	7.99
A1ZBU8	CG18067 OS=Drosophila melanogaster GN=CG18067 PE=4 SV=1 - [A1ZBU8_DROME]	2.62	7.35	1	2	2	2	5.236E7	245	26.2	5.29
Q9VAJ9	CG1907 OS=Drosophila melanogaster GN=CG1907 PE=2 SV=1 - [Q9VAJ9_DROME]	2.62	3.79	1	1	1	1	1.007E8	317	34.6	9.79
Q9VP77	CG10565, isoform A OS=Drosophila melanogaster GN=CG10565 PE=2 SV=1 - [Q9VP77_DROME]	2.62	3.41	1	2	2	3	3.834E7	646	73.6	8.97
P98159	Serine protease nudel OS=Drosophila melanogaster GN=ndl PE=1 SV=2 - [NUDEL_DROME]	2.62	1.91	1	2	2	2	1.413E7	2616	292.3	5.92
P14318	Muscle-specific protein 20 OS=Drosophila melanogaster GN=Mp20 PE=2 SV=2 - [MP20_DROME]	2.62	7.07	1	1	1	1		184	20.2	8.65
Q8IML2	V-type proton ATPase subunit a OS=Drosophila melanogaster GN=Vha100-1 PE=3 SV=2 - [Q8IML2_DROME]	2.60	1.33	6	1	1	1	2.123E7	825	94.1	7.46
Q9VQD7	CG3214, isoform B OS=Drosophila melanogaster GN=CG3214 PE=4 SV=1 - [Q9VQD7_DROME]	2.60	9.15	1	1	1	1	1.527E7	142	16.8	9.23
Q9VBX1	Nuclear export mediator factor NEMF homolog OS=Drosophila melanogaster GN=Clbn PE=1 SV=2 - [NEMF_DROME]	2.60	7.46	1	3	3	3	1.083E7	992	112.5	7.17
Q8IMR3	CG5484, isoform B OS=Drosophila melanogaster GN=CG5484-RB PE=2 SV=1 - [Q8IMR3_DROME]	2.59	2.54	3	1	1	1	2.015E7	393	43.6	9.23
Q9VRD4	CG1532, isoform B OS=Drosophila melanogaster GN=CG1532 PE=4 SV=1 - [Q9VRD4_DROME]	2.58	6.25	1	1	1	1		288	31.6	5.39
Q9VX15	CG8142 OS=Drosophila melanogaster GN=CG8142 PE=2 SV=2 - [Q9VX15_DROME]	2.57	2.83	1	1	1	1		353	39.5	7.87
Q9U616	Glycine cleavage system H protein, mitochondrial OS=Drosophila melanogaster GN=ppl PE=2 SV=1 - [GCSH_DROME]	2.57	10.91	1	1	1	1	1.138E7	165	18.0	5.20
Q9VWF8	CG14210, isoform B OS=Drosophila melanogaster GN=CG14210 PE=2 SV=1 - [Q9VWF8_DROME]	2.57	7.86	1	2	2	2	8.948E6	140	16.5	10.81
Q9W011	Probable cytochrome P450 4d20 OS=Drosophila melanogaster GN=Cyp4d20 PE=3 SV=1 - [C4D20_DROME]	2.56	4.12	1	1	1	1		510	58.2	8.57
A1ZB29	CG5757 OS=Drosophila melanogaster GN=CG5757 PE=3 SV=1 - [A1ZB29_DROME]	2.56	5.69	1	1	1	1	8.396E6	211	23.8	7.44
Q9VXY3	CG5599 OS=Drosophila melanogaster GN=CG5599 PE=2 SV=1 - [Q9VXY3_DROME]	2.56	4.98	1	1	1	1	2.803E7	462	50.0	6.61
M9PGM3	CG42593, isoform C OS=Drosophila melanogaster GN=CG1530 PE=4 SV=1 - [M9PGM3_DROME]	2.54	1.67	3	1	1	1		2218	246.8	5.87
Q9W4C4	CG3249-PA, isoform A OS=Drosophila melanogaster GN=spoon PE=2 SV=2 - [Q9W4C4_DROME]	2.53	4.10	2	2	2	2	6.441E7	585	64.9	6.34
Q8IPK1	Uncoupling protein 4B, isoform B OS=Drosophila melanogaster GN=Ucp4B PE=3 SV=1 - [Q8IPK1_DROME]	2.53	8.56	3	1	1	1		222	24.6	9.29
Q9VTY6	Ubiquitin-conjugating enzyme E2 C OS=Drosophila melanogaster GN=vih PE=1 SV=1 - [UBE2C_DROME]	2.53	16.29	1	2	2	2	1.429E7	178	19.8	6.68
P52034	ATP-dependent 6-phosphofructokinase OS=Drosophila melanogaster GN=Pfk PE=2 SV=2 - [PFKA_DROME]	2.53	1.52	1	1	1	1		788	86.6	6.83
Q86BI3	CG5215-PB, isoform B OS=Drosophila melanogaster GN=Zn72D PE=4 SV=1 - [Q86BI3_DROME]	2.53	3.96	3	3	3	4	1.706E7	884	96.0	8.44
Q9W088	DNA polymerase delta small subunit OS=Drosophila melanogaster GN=CG12018 PE=2 SV=1 - [DPOD2_DROME]	2.52	2.78	1	1	1	2	8.461E6	431	48.0	6.52
O76511	Thymidylate synthase OS=Drosophila melanogaster GN=Ts PE=1 SV=2 - [TYSY_DROME]	2.51	2.80	1	1	1	1	2.202E7	321	36.6	6.61
Q9W335	LD26546p OS=Drosophila melanogaster GN=l(1)G0320 PE=2 SV=1 - [Q9W335_DROME]	2.51	11.59	1	2	2	2	1.420E7	302	33.0	4.84

Accession	Description	Score	Coverage	# Proteins	# Unique Peptides	# Peptides	# PSMs	Area	# AAs	MW [kDa]	calc. pI
Q8IP52	CG43333 OS=Drosophila melanogaster GN=BG:DS01523.2 PE=2 SV=1 - [Q8IP52_DROME]	2.51	1.54	1	1	1	1	7.548E6	1166	128.8	8.54
P52304	Serine/threonine-protein kinase polo OS=Drosophila melanogaster GN=polo PE=1 SV=2 - [POLO_DROME]	2.51	7.81	1	4	4	4	3.792E7	576	66.9	8.88
Q9VB69	Malic enzyme OS=Drosophila melanogaster GN=Men-b PE=2 SV=1 - [Q9VB69_DROME]	2.50	3.24	3	1	1	1	1.955E7	617	68.5	6.71
Q9VKC5	CG6770-PA OS=Drosophila melanogaster GN=CG6770-RA PE=2 SV=1 - [Q9VKC5_DROME]	2.50	23.19	1	1	1	1	4.112E7	69	8.1	8.76
Q9W592	CG14786 OS=Drosophila melanogaster GN=EG:BACN32G11.3 PE=4 SV=1 - [Q9W592_DROME]	2.50	1.12	1	1	1	1	8.328E6	1072	120.1	8.22
Q9VVJ7	CG7484 OS=Drosophila melanogaster GN=CG7484 PE=1 SV=2 - [Q9VVJ7_DROME]	2.50	11.24	1	2	2	2	2.664E7	178	19.9	5.66
Q7JQH9	LD31742p OS=Drosophila melanogaster GN=whd PE=2 SV=1 - [Q7JQH9_DROME]	2.50	2.56	2	2	2	2	1.017E7	780	89.4	8.46
P91875	DNA-directed RNA polymerase I subunit RPA1 OS=Drosophila melanogaster GN=RpI1 PE=1 SV=2 - [RPA1_DROME]	2.49	1.95	1	2	2	2	2.666E7	1642	185.3	7.64
Q9GYU8	Nuclear pore complex protein Nup88 OS=Drosophila melanogaster GN=mbo PE=1 SV=2 - [NUP88_DROME]	2.49	1.14	1	1	1	1	2.291E7	702	78.6	5.91
A8DY43	CG34372 OS=Drosophila melanogaster GN=CG34372 PE=4 SV=1 - [A8DY43_DROME]	2.47	5.15	1	2	2	2		1010	112.0	8.28
Q9VVC8	CG6664, isoform A OS=Drosophila melanogaster GN=CG6664 PE=2 SV=1 - [Q9VVC8_DROME]	2.47	1.88	1	1	1	1	7.552E6	532	57.8	6.67
A1ZA22	eIF2B-gamma OS=Drosophila melanogaster GN=eIF2B-gamma PE=4 SV=1 - [A1ZA22_DROME]	2.47	3.08	1	1	1	1		455	50.6	7.55
Q9VUJ0	39S ribosomal protein L39, mitochondrial OS=Drosophila melanogaster GN=mRpL39 PE=1 SV=2 - [RM39_DROME]	2.46	8.41	1	2	2	2	1.231E7	333	37.3	8.62
A1YK02	Nup75 OS=Drosophila melanogaster GN=Nup75 PE=4 SV=1 - [A1YK02_DROME]	2.45	3.59	1	2	2	2	4.974E6	668	76.8	6.05
Q8MSS1	Protein lava lamp OS=Drosophila melanogaster GN=lva PE=1 SV=2 - [LVA_DROME]	2.45	0.43	1	1	1	1	3.930E6	2779	315.7	4.86
Q9VNB5	Odorant receptor coreceptor OS=Drosophila melanogaster GN=Orco PE=1 SV=2 - [OR83B_DROME]	2.44	4.12	1	1	1	1		486	54.4	8.50
Q9VV52	LD37257p OS=Drosophila melanogaster GN=RAF2 PE=2 SV=3 - [Q9VV52_DROME]	2.44	6.18	1	3	3	3	1.107E6	1117	125.4	9.26
Q9VXN2	CG9032-PA, isoform A OS=Drosophila melanogaster GN=sun PE=2 SV=1 - [Q9VXN2_DROME]	2.43	19.67	1	1	1	1	5.218E7	61	6.8	10.59
Q7KLE5	Amphiphysin OS=Drosophila melanogaster GN=Amph PE=1 SV=1 - [Q7KLE5_DROME]	2.43	1.99	1	1	1	1	1.796E7	602	65.9	5.25
Q8IN97	Cellular repressor of E1A-stimulated genes, isoform C OS=Drosophila melanogaster GN=CREG PE=4 SV=1 - [Q8IN97_DROME]	2.43	7.51	2	1	1	1	1.208E7	173	19.7	9.23
Q94519	Acyl carrier protein, mitochondrial OS=Drosophila melanogaster GN=mtacp1 PE=2 SV=1 - [ACPM_DROME]	2.43	5.92	2	1	1	1		152	17.2	6.79
Q9VAG9	CG7789 OS=Drosophila melanogaster GN=CG7789 PE=2 SV=1 - [Q9VAG9_DROME]	2.42	3.27	1	1	1	1		306	32.6	5.92
P52654	Transcription initiation factor IIA subunit 1 OS=Drosophila melanogaster GN=TfIIA-L PE=1 SV=2 - [TF2AA_DROME]	2.42	6.28	1	1	1	1	7.888E6	366	39.2	4.73
Q7KQM8	CG11076, isoform D OS=Drosophila melanogaster GN=CG11076 PE=4 SV=2 - [Q7KQM8_DROME]	2.42	6.15	2	1	1	1		260	29.5	9.26
Q9W0R0	Cell division cycle 5 ortholog, isoform A OS=Drosophila melanogaster GN=Cdc5 PE=2 SV=2 - [Q9W0R0_DROME]	2.41	1.35	1	1	1	1		814	93.0	8.19
Q9VYH3	Probable alpha-aspartyl dipeptidase OS=Drosophila melanogaster GN=CG2200 PE=1 SV=1 - [PEPE_DROME]	2.41	6.67	1	1	1	2	6.799E6	240	26.6	7.03
Q7JWD6	Elongin C, isoform A OS=Drosophila melanogaster GN=EloC PE=1 SV=1 - [Q7JWD6_DROME]	2.41	8.55	1	1	1	1	9.763E7	117	13.1	5.05
Q9VAI6	General odorant-binding protein 99b OS=Drosophila melanogaster GN=Obp99b PE=2 SV=1 - [OB99B_DROME]	2.40	12.75	1	2	2	2	6.356E7	149	17.2	6.60
Q9VGX3	Protein anoxia up-regulated OS=Drosophila melanogaster GN=fau PE=1 SV=3 - [FAU_DROME]	2.39	3.23	1	2	2	2		619	68.6	5.06
Q8MKW7	Ribonuclease Z, mitochondrial OS=Drosophila melanogaster GN=JhI-1 PE=1 SV=2 - [RNZ_DROME]	2.39	1.70	1	1	1	1		766	85.4	6.99
Q9W2I2	CG9752 OS=Drosophila melanogaster GN=CG9752 PE=4 SV=1 - [Q9W2I2_DROME]	2.39	6.71	1	1	1	1		343	39.5	5.73
Q8INK9	Methionine-R-sulfoxide reductase B1 OS=Drosophila melanogaster GN=SelR PE=1 SV=3 - [MSRB_DROME]	2.39	11.54	1	1	1	3	2.604E7	208	23.3	8.70
Q9W547	LD21404p OS=Drosophila melanogaster GN=mRpL16 PE=2 SV=1 - [Q9W547_DROME]	2.39	4.12	1	1	1	1	3.872E6	243	28.0	10.17
M9MS86	Mucin related 2B, isoform B OS=Drosophila melanogaster GN=Mur2B PE=4 SV=1 - [M9MS86_DROME]	2.38	0.96	2	1	1	1	4.487E7	1144	124.2	9.25
Q8T045	O-glucosyltransferase rumi OS=Drosophila melanogaster GN=rumi PE=1 SV=1 - [RUMI_DROME]	2.38	2.92	1	1	1	1	1.237E7	411	47.7	8.25
Q9VN68	Cdep, isoform C OS=Drosophila melanogaster GN=Cdep PE=2 SV=2 - [Q9VN68_DROME]	2.38	5.94	2	1	1	1	9.488E6	404	45.9	9.29
Q9VAP0	CG14516, isoform A OS=Drosophila melanogaster GN=CG14516 PE=4 SV=2 - [Q9VAP0_DROME]	2.37	0.81	2	1	1	1	3.842E6	990	114.1	5.88
Q9VE52	Cleavage stimulation factor 64 kilodalton subunit OS=Drosophila melanogaster GN=CstF-64 PE=2 SV=2 - [Q9VE52_DROME]	2.36	3.10	1	1	1	1	1.357E7	419	46.2	5.78
Q9W1K4	Egalitarian OS=Drosophila melanogaster GN=egl PE=4 SV=4 - [Q9W1K4_DROME]	2.35	3.49	1	2	2	3	2.674E7	1004	112.1	7.28
A8DY87	cAMP-dependent protein kinase R2, isoform D OS=Drosophila melanogaster GN=Pka-R2 PE=4 SV=1 - [A8DY87_DROME]	2.34	3.22	3	1	1	1	1.839E7	373	42.4	4.92
Q7KJA9	O-glycosyltransferase OS=Drosophila melanogaster GN=sxc PE=2 SV=1 - [Q7KJA9_DROME]	2.34	1.61	1	1	1	1	4.315E6	1059	118.6	7.15
Q9VHC6	Rlc1, isoform A OS=Drosophila melanogaster GN=mRpL47 PE=4 SV=2 - [Q9VHC6_DROME]	2.34	4.85	2	1	1	1	1.673E7	227	26.4	9.77
Q9W379	LD37736p OS=Drosophila melanogaster GN=Zpr1 PE=2 SV=1 - [Q9W379_DROME]	2.34	7.88	1	2	2	2	2.267E7	457	51.3	4.74
Q9VR89	RNA-binding protein pno1 OS=Drosophila melanogaster GN=l(1)G0004 PE=2 SV=1 - [PNO1_DROME]	2.32	4.17	1	1	1	1	2.273E7	240	26.7	9.95
Q8MSS7	CG2937-PA OS=Drosophila melanogaster GN=mRpS2 PE=2 SV=1 - [Q8MSS7_DROME]	2.32	4.17	1	1	1	1	6.485E6	264	30.2	9.14
Q07327	Protein ROP OS=Drosophila melanogaster GN=Rop PE=2 SV=2 - [ROP_DROME]	2.31	2.18	1	1	1	1	6.338E6	597	67.8	6.70
Q9V5P6	H/ACA ribonucleoprotein complex subunit 3 OS=Drosophila melanogaster GN=CG7637 PE=1 SV=1 - [NOP10_DROME]	2.31	35.94	1	2	2	3	1.607E8	64	7.6	9.92
Q7K2L7	CG17765 OS=Drosophila melanogaster GN=CG17765 PE=1 SV=1 - [Q7K2L7_DROME]	2.31	8.04	1	2	2	2	3.326E7	199	22.5	4.86
A8DYI1	CG30118, isoform B OS=Drosophila melanogaster GN=CG30118 PE=4 SV=1 - [A8DYI1_DROME]	2.30	2.97	3	1	1	1		471	53.5	8.84
Q8INB9	RAC serine/threonine-protein kinase OS=Drosophila melanogaster GN=Akt1 PE=1 SV=3 - [AKT1_DROME]	2.30	3.44	1	1	1	1	2.548E6	611	68.4	6.24

Accession	Description	Score	Coverage	# Proteins	# Unique Peptides	# Peptides	# PSMs	Area	# AAs	MW [kDa]	calc. pI
Q9V3Y4	CG6851-PA, isoform A OS=Drosophila melanogaster GN=Mtch PE=2 SV=1 - [Q9V3Y4_DROME]	2.30	5.06	1	1	1	2	3.652E6	316	35.0	9.06
A1Z7K2	CG30349 OS=Drosophila melanogaster GN=CG30349-RA PE=2 SV=1 - [A1Z7K2_DROME]	2.29	1.77	1	1	1	1	3.295E7	620	68.3	5.94
Q9XZH6	V-type proton ATPase subunit G OS=Drosophila melanogaster GN=Vha13 PE=1 SV=1 - [VATG_DROME]	2.29	15.38	1	2	2	2	6.946E7	117	13.6	9.58
Q9VJH2	CG31739, isoform A OS=Drosophila melanogaster GN=mdy PE=2 SV=3 - [Q9VJH2_DROME]	2.29	1.39	1	1	1	1		1082	121.4	5.82
Q7JVK6	GM27569p OS=Drosophila melanogaster GN=trsn PE=1 SV=1 - [Q7JVK6_DROME]	2.28	5.11	1	1	1	1		235	27.0	5.50
O77266	CG4199, isoform A OS=Drosophila melanogaster GN=CG4199 PE=4 SV=3 - [O77266_DROME]	2.27	1.81	2	1	1	1	1.056E7	552	60.7	5.73
Q9VTN6	CG5964 OS=Drosophila melanogaster GN=CG5964 PE=4 SV=1 - [Q9VTN6_DROME]	2.27	2.48	1	1	1	1		928	106.3	5.47
M9PCS5	CG18304, isoform C OS=Drosophila melanogaster GN=CG18304 PE=4 SV=1 - [M9PCS5_DROME]	2.26	0.83	4	1	1	1		1813	204.3	9.29
Q9VII1	CG9336, isoform A OS=Drosophila melanogaster GN=CG9336-RA PE=2 SV=1 - [Q9VII1_DROME]	2.25	16.22	2	2	2	2	2.721E7	148	15.9	7.71
Q960K8	CG5044, isoform A OS=Drosophila melanogaster GN=CG5044 PE=2 SV=1 - [Q960K8_DROME]	2.25	9.09	2	2	2	2	1.341E7	385	42.7	8.35
Q9VKW1	CG5168, isoform A OS=Drosophila melanogaster GN=CG5168 PE=2 SV=1 - [Q9VKW1_DROME]	2.25	4.17	1	1	1	1	9.728E6	408	46.2	7.37
Q9V463	Nucleoporin 154kD, isoform A OS=Drosophila melanogaster GN=Nup154 PE=2 SV=1 - [Q9V463_DROME]	2.25	5.57	1	4	4	5	3.875E7	1365	153.8	6.58
Q8IR41	CG43737, isoform C OS=Drosophila melanogaster GN=CG15029 PE=4 SV=2 - [Q8IR41_DROME]	2.25	1.62	2	1	1	1	7.486E7	1483	157.0	4.68
Q9W2G8	Acyl-coenzyme A oxidase OS=Drosophila melanogaster GN=Acox57D-d PE=2 SV=1 - [Q9W2G8_DROME]	2.24	3.40	1	1	1	1	1.346E7	677	75.4	8.34
M9PB96	Ribosome biogenesis protein WDR12 homolog OS=Drosophila melanogaster GN=CG6724 PE=3 SV=1 - [M9PB96_DROME]	2.24	7.18	2	2	2	5	2.639E8	418	46.9	5.31
P91623	GATA-binding factor C OS=Drosophila melanogaster GN=grn PE=2 SV=1 - [GATAC_DROME]	2.24	5.35	2	1	1	1		486	50.6	9.36
Q7KT36	CG13083 OS=Drosophila melanogaster GN=CG13083-RB PE=2 SV=1 - [Q7KT36_DROME]	2.24	14.56	1	3	3	4	1.757E8	316	35.4	6.28
Q7K4G8	CG12391 OS=Drosophila melanogaster GN=CG12391 PE=2 SV=1 - [Q7K4G8_DROME]	2.24	2.73	1	1	1	1	4.407E6	587	66.4	4.81
Q01989	Myosin heavy chain 95F OS=Drosophila melanogaster GN=jar PE=2 SV=4 - [MYS9_DROME]	2.24	3.59	1	3	3	3	1.003E7	1253	143.2	8.63
Q9W2U8	Neb-cGP, isoform A OS=Drosophila melanogaster GN=Neb-cGP PE=2 SV=1 - [Q9W2U8_DROME]	2.23	20.83	1	1	1	2	3.061E8	48	5.1	10.10
Q9VU65	POC1 centriolar protein homolog OS=Drosophila melanogaster GN=Poc1 PE=2 SV=1 - [POC1_DROME]	2.23	4.09	2	1	1	1		391	42.8	8.44
Q9Y102	BcDNA.GH11973 OS=Drosophila melanogaster GN=rgn PE=2 SV=1 - [Q9Y102_DROME]	2.23	1.50	2	1	1	2		800	97.3	9.22
Q9W225	CG3746 OS=Drosophila melanogaster GN=CG3746 PE=2 SV=1 - [Q9W225_DROME]	2.23	14.57	1	1	1	1	1.614E7	151	16.6	8.09
Q8SY96	NFU1 iron-sulfur cluster scaffold homolog, mitochondrial OS=Drosophila melanogaster GN=CG32500 PE=2 SV=1 - [NFU1_DROME]	2.22	6.71	1	1	1	1		283	31.3	5.40
Q00637	Superoxide dismutase [Mn], mitochondrial OS=Drosophila melanogaster GN=Sod2 PE=2 SV=3 - [SODM_DROME]	2.22	7.37	1	2	2	2	6.077E7	217	24.7	8.15
P40301	Proteasome subunit alpha type-2 OS=Drosophila melanogaster GN=Prosalpha2 PE=1 SV=1 - [PSA2_DROME]	2.22	5.98	1	2	2	2	5.399E7	234	25.9	6.68
Q9VMU7	CG14036-PA OS=Drosophila melanogaster GN=CG14036-RA PE=2 SV=1 - [Q9VMU7_DROME]	2.21	12.90	1	1	1	1	5.019E7	93	11.0	7.78
Q8IN71	Anoctamin OS=Drosophila melanogaster GN=subdued PE=3 SV=1 - [Q8IN71_DROME]	2.20	3.56	2	2	2	2	8.151E6	926	107.5	6.92
O02002	Caspase-1 OS=Drosophila melanogaster GN=Dcp-1 PE=1 SV=1 - [CASP1_DROME]	2.20	9.91	1	2	2	2	1.017E7	323	35.9	7.74
P49905	Transcription initiation factor TFIID subunit 12 OS=Drosophila melanogaster GN=Taf12 PE=1 SV=1 - [TAF12_DROME]	2.20	10.71	1	1	1	1	4.611E6	196	21.5	7.12
Q9V677	Proteasome-associated protein ECM29 homolog OS=Drosophila melanogaster GN=CG8858 PE=1 SV=1 - [ECM29_DROME]	2.20	1.96	1	2	2	2	9.212E6	1890	212.0	6.86
Q9VLT5	Protein purity of essence OS=Drosophila melanogaster GN=poe PE=1 SV=1 - [POE_DROME]	2.20	1.09	1	3	3	3		5322	590.3	6.38
Q9VVA1	CG9706, isoform B OS=Drosophila melanogaster GN=CG9706-RA PE=2 SV=1 - [Q9VVA1_DROME]	2.20	1.90	1	1	1	1	5.473E6	525	59.1	8.12
Q9VVI9	Charged multivesicular body protein 5 OS=Drosophila melanogaster GN=CG6259 PE=1 SV=2 - [CHMP5_DROME]	2.20	14.60	1	2	2	2	1.168E7	226	25.1	4.75
Q9W4U2	CG3603, isoform B OS=Drosophila melanogaster GN=CG3603 PE=2 SV=2 - [Q9W4U2_DROME]	2.20	10.44	1	2	2	2	7.903E7	249	26.2	7.80
P07183	Chorion protein S38 OS=Drosophila melanogaster GN=Cp38 PE=2 SV=1 - [CH38_DROME]	2.19	26.14	1	3	3	6	1.153E8	306	30.4	9.13
Q9W1I8	Synaptosomal-associated protein OS=Drosophila melanogaster GN=Snap29 PE=2 SV=1 - [Q9W1I8_DROME]	2.19	4.58	1	1	1	1	2.857E7	284	31.6	5.17
O46050	CG3460-PA OS=Drosophila melanogaster GN=Nmd3 PE=2 SV=1 - [O46050_DROME]	2.18	4.61	1	2	2	2	1.826E7	521	59.1	6.06
Q9VKB4	Probable ribosome production factor 1 OS=Drosophila melanogaster GN=CG6712 PE=2 SV=1 - [RPF1_DROME]	2.17	3.81	1	1	1	1	1.891E7	394	46.0	6.70
Q9VLU0	Barrier-to-autointegration factor OS=Drosophila melanogaster GN=baf PE=3 SV=1 - [BAF_DROME]	2.17	27.78	1	2	2	3	7.985E7	90	10.1	5.16
Q9VQ58	CG15356 OS=Drosophila melanogaster GN=CG15356-RA PE=2 SV=1 - [Q9VQ58_DROME]	2.16	1.96	1	1	1	1		1023	108.1	9.44
Q0KI00	FI20034p1 OS=Drosophila melanogaster GN=SP1029 PE=2 SV=1 - [Q0KI00_DROME]	2.16	1.72	1	1	1	1		932	108.0	6.60
Q9VT75	CG11989-PA, isoform A OS=Drosophila melanogaster GN=vnc PE=2 SV=1 - [Q9VT75_DROME]	2.16	15.31	1	3	3	4	1.791E7	196	22.3	6.95
P22465	Annexin B10 OS=Drosophila melanogaster GN=AnxB10 PE=2 SV=3 - [ANX10_DROME]	2.16	7.48	1	2	2	3	7.228E7	321	35.7	4.74
Q9VLK1	CG13097 OS=Drosophila melanogaster GN=CG13097 PE=2 SV=1 - [Q9VLK1_DROME]	2.15	3.32	1	1	1	1	2.254E7	662	76.1	4.77
M9PH72	CG42324, isoform I OS=Drosophila melanogaster GN=CG14972 PE=4 SV=1 - [M9PH72_DROME]	2.15	2.17	4	1	1	1		923	100.2	6.00
Q9VYG8	CG15717, isoform A OS=Drosophila melanogaster GN=CG15717 PE=2 SV=1 - [Q9VYG8_DROME]	2.15	3.97	1	1	1	1	4.399E7	252	27.7	6.29
Q8IPJ2	CG17378, isoform C OS=Drosophila melanogaster GN=CG17378 PE=4 SV=1 - [Q8IPJ2_DROME]	2.15	4.81	2	1	1	1		561	62.3	8.18
Q9VJ59	Jwa ortholog OS=Drosophila melanogaster GN=Jwa PE=4 SV=5 - [Q9VJ59_DROME]	2.15	4.42	1	1	1	1		249	27.8	9.99
M9PC31	CG3714, isoform G OS=Drosophila melanogaster GN=CG3714 PE=4 SV=1 - [M9PC31_DROME]	2.14	2.00	3	1	1	1	2.998E7	550	61.5	6.71

Accession	Description	Score	Coverage	# Proteins	# Unique Peptides	# Peptides	# PSMs	Area	# AAs	MW [kDa]	calc. pI
Q9VRJ9	Ubiquitin thioesterase OTU1 OS=Drosophila melanogaster GN=CG4603 PE=2 SV=1 - [OTU1_DROME]	2.14	3.46	2	1	1	1		347	37.8	4.82
Q8MS38	CG33217-PA OS=Drosophila melanogaster GN=CG33217 PE=2 SV=1 - [Q8MS38_DROME]	2.14	7.44	1	3	3	3		699	79.9	8.10
P40688	Protein swallow OS=Drosophila melanogaster GN=swa PE=1 SV=1 - [SWA_DROME]	2.14	1.82	1	1	1	1	1.997E7	548	62.0	6.14
Q7JZF5	RE34924p OS=Drosophila melanogaster GN=Rpb5 PE=2 SV=1 - [Q7JZF5_DROME]	2.14	4.29	1	1	1	1	1.367E7	210	24.5	6.15
Q9VZW7	CG2107 OS=Drosophila melanogaster GN=CG2107 PE=3 SV=1 - [Q9VZW7_DROME]	2.14	1.80	1	1	1	1	1.020E7	667	75.1	8.35
Q7KVP4	CG9485, isoform A OS=Drosophila melanogaster GN=CG9485 PE=4 SV=1 - [Q7KVP4_DROME]	2.13	1.04	4	1	1	1	1.193E7	1542	173.5	6.47
Q27884	Serine/threonine-protein phosphatase 6 catalytic subunit OS=Drosophila melanogaster GN=PpV PE=2 SV=1 - [PPP6_DROME]	2.12	3.30	1	1	1	1	1.353E7	303	34.7	5.71
P27864	Recombination repair protein 1 OS=Drosophila melanogaster GN=Rrp1 PE=1 SV=2 - [RRP1_DROME]	2.12	3.39	2	1	1	1	5.219E6	679	74.6	8.98
Q24050	Anon-i1 protein OS=Drosophila melanogaster GN=anon-i1 PE=2 SV=1 - [Q24050_DROME]	2.12	3.44	1	1	1	1	1.620E7	262	28.9	4.82
A1Z8L8	CG7777, isoform A OS=Drosophila melanogaster GN=CG7777 PE=3 SV=1 - [A1Z8L8_DROME]	2.12	3.79	2	1	1	1	6.643E7	264	28.4	7.15
Q9W0M4	CG13887, isoform C OS=Drosophila melanogaster GN=CG13887 PE=2 SV=1 - [Q9W0M4_DROME]	2.12	10.96	1	2	2	3	2.186E7	228	26.1	9.32
Q9VSC1	Signal recognition particle 9 kDa protein OS=Drosophila melanogaster GN=Srp9 PE=3 SV=1 - [SRP09_DROME]	2.12	16.88	1	1	1	1	2.895E7	77	9.0	8.46
Q9V9W7	Rough deal OS=Drosophila melanogaster GN=rod PE=4 SV=2 - [Q9V9W7_DROME]	2.11	0.48	1	1	1	1		2089	239.5	6.15
Q9W197	7-methylguanosine phosphate-specific 5'-nucleotidase OS=Drosophila melanogaster GN=CG3362 PE=1 SV=1 - [5NT3B_DROME]	2.11	2.82	1	1	1	1		319	36.3	5.44
A1Z6I8	Maleless OS=Drosophila melanogaster GN=mle PE=4 SV=1 - [A1Z6I8_DROME]	2.11	15.04	2	2	2	2	3.666E6	226	25.0	7.27
Q9VD26	Zinc finger protein-like 1 homolog OS=Drosophila melanogaster GN=CG5382 PE=1 SV=1 - [ZFPL1_DROME]	2.11	4.68	1	1	1	1	3.520E6	299	33.5	8.53
Q9VT68	CG6707, isoform B OS=Drosophila melanogaster GN=CG6707 PE=4 SV=2 - [Q9VT68_DROME]	2.11	4.33	2	1	1	1	7.287E7	231	25.2	8.95
P48603	F-actin-capping protein subunit beta OS=Drosophila melanogaster GN=cpb PE=2 SV=1 - [CAPZB_DROME]	2.10	7.25	1	1	1	1		276	31.3	5.44
A1ZAW5	Methylosome subunit pICln OS=Drosophila melanogaster GN=icln PE=1 SV=1 - [ICLN_DROME]	2.10	5.58	1	1	1	1	5.498E7	215	23.7	4.12
Q02427	RNA-binding protein 1 OS=Drosophila melanogaster GN=Rbp1 PE=2 SV=3 - [RBP1_DROME]	2.09	14.58	3	2	2	2	1.747E8	144	16.0	9.98
Q9VE86	Werner Syndrome-like exonuclease OS=Drosophila melanogaster GN=WRNexo PE=1 SV=2 - [WRNXO_DROME]	2.09	3.68	2	1	1	1	4.497E6	353	40.3	9.09
Q24151	Signal transducer and transcription activator OS=Drosophila melanogaster GN=Stat92E PE=1 SV=1 - [STAT_DROME]	2.09	2.10	1	1	1	1		761	86.4	6.14
Q94524	Dynein light chain Tctex-type OS=Drosophila melanogaster GN=Dlc90F PE=1 SV=1 - [DYLT_DROME]	2.09	16.22	1	1	1	1		111	12.5	5.16
Q9VJE3	CG15141 OS=Drosophila melanogaster GN=CG15141 PE=2 SV=1 - [Q9VJE3_DROME]	2.09	3.22	1	1	1	1		404	45.9	4.93
Q9V3D9	BcDNA.GM09489 OS=Drosophila melanogaster GN=Srp54k PE=2 SV=1 - [Q9V3D9_DROME]	2.09	1.97	1	1	1	1		508	55.8	8.70
Q9VF08	Mitochondrial import inner membrane translocase subunit Tim16 OS=Drosophila melanogaster GN=blp PE=2 SV=1 - [TIM16_DROME]	2.08	12.06	1	1	1	1	2.225E7	141	15.7	9.60
Q7K1C3	CG6984 OS=Drosophila melanogaster GN=CG6984 PE=2 SV=1 - [Q7K1C3_DROME]	2.08	5.61	1	1	1	1	3.559E6	285	31.1	8.35
Q9VJ74	CG10333 OS=Drosophila melanogaster GN=CG10333 PE=2 SV=2 - [Q9VJ74_DROME]	2.08	4.14	1	2	2	2	1.241E8	822	94.5	9.47
Q9W3Y0	Zinc finger protein 593 homolog OS=Drosophila melanogaster GN=CG3224 PE=2 SV=1 - [ZN593_DROME]	2.08	12.35	1	1	1	1		162	18.9	9.67
E1JHY0	CG5946, isoform E OS=Drosophila melanogaster GN=CG5946 PE=4 SV=1 - [E1JHY0_DROME]	2.08	4.94	6	1	1	1	4.101E7	263	29.7	9.55
Q9VTH0	Procollagen lysyl hydroxylase, isoform A OS=Drosophila melanogaster GN=Plod PE=4 SV=1 - [Q9VTH0_DROME]	2.07	3.61	1	2	2	2	1.520E7	721	82.5	5.68
Q9VC93	Dis3 OS=Drosophila melanogaster GN=Dis3 PE=3 SV=1 - [Q9VC93_DROME]	2.07	1.83	1	1	1	1		982	112.1	6.68
Q9I7J0	CG5023 OS=Drosophila melanogaster GN=CG5023 PE=2 SV=1 - [Q9I7J0_DROME]	2.07	7.10	1	1	1	1	1.965E7	169	19.1	8.13
Q9VW12	UPF0389 protein CG9231 OS=Drosophila melanogaster GN=CG9231 PE=1 SV=1 - [U389_DROME]	2.07	32.80	1	3	3	3	4.280E7	125	14.1	10.07
Q9VCG4	Nucleoporin Ndc1 OS=Drosophila melanogaster GN=Ndc1 PE=1 SV=1 - [NDC1_DROME]	2.07	3.98	1	2	2	2	5.405E7	578	65.3	8.37
Q8I0J3	Mitochondrial ribosomal protein S21 OS=Drosophila melanogaster GN=mRpS21 PE=2 SV=1 - [Q8I0J3_DROME]	2.07	16.09	1	1	1	1	1.881E7	87	10.6	10.08
Q960T2	Synapse-associated protein of 47 kDa OS=Drosophila melanogaster GN=Sap47 PE=1 SV=1 - [SAP47_DROME]	2.07	2.90	1	1	1	1		551	56.9	4.56
A1Z9A2	UPF0587 protein CG4646 OS=Drosophila melanogaster GN=CG4646 PE=2 SV=1 - [U587_DROME]	2.06	7.98	1	1	1	1	7.986E6	163	18.6	4.96
Q9VYT3	CG2025 OS=Drosophila melanogaster GN=CG2025 PE=3 SV=2 - [Q9VYT3_DROME]	2.05	2.70	1	3	3	4	2.204E7	1147	132.7	5.15
Q9VWE6	Protein ELYS homolog OS=Drosophila melanogaster GN=CG14215 PE=1 SV=2 - [ELYS_DROME]	2.05	0.76	1	1	1	1		2111	235.0	5.33
Q8MT06	Guanine nucleotide-binding protein-like 3 homolog OS=Drosophila melanogaster GN=Ns1 PE=1 SV=2 - [GNL3_DROME]	2.05	7.57	1	3	3	5	3.144E7	581	65.9	9.36
M9PGB6	CG7519, isoform B OS=Drosophila melanogaster GN=CG7519 PE=4 SV=1 - [M9PGB6_DROME]	2.05	8.61	2	1	1	1		151	17.6	5.27
Q9W114	CG12848 OS=Drosophila melanogaster GN=CG12848 PE=2 SV=2 - [Q9W114_DROME]	2.04	13.33	1	1	1	1		120	13.7	9.11
Q09147	Fibroblast growth factor receptor homolog 2 OS=Drosophila melanogaster GN=btl PE=2 SV=3 - [FGFR2_DROME]	2.04	0.76	1	1	1	2	2.241E8	1052	117.7	6.28
A1Z7K8	CG8235 OS=Drosophila melanogaster GN=CG8235 PE=4 SV=1 - [A1Z7K8_DROME]	2.04	2.79	1	1	1	1	5.218E7	323	34.4	8.91
Q8INE8	Arginine methyltransferase 3, isoform B OS=Drosophila melanogaster GN=Art3 PE=2 SV=1 - [Q8INE8_DROME]	2.04	3.59	2	1	1	2	5.533E6	474	54.2	5.08
Q9W3B2	CG10648-PA OS=Drosophila melanogaster GN=Rbm13 PE=2 SV=1 - [Q9W3B2_DROME]	2.04	3.79	1	1	1	1		343	40.6	4.89
Q7K4N3	Nuclear cap-binding protein subunit 1 OS=Drosophila melanogaster GN=Cbp80 PE=1 SV=1 - [NCBP1_DROME]	2.03	2.63	1	1	1	1	2.200E7	800	93.2	6.55
Q7KSQ0	CG6782, isoform A OS=Drosophila melanogaster GN=sea PE=2 SV=1 - [Q7KSQ0_DROME]	2.03	2.52	1	1	1	1	7.831E7	317	34.1	9.42
M9NE07	CG9132, isoform C OS=Drosophila melanogaster GN=CG9132 PE=4 SV=1 - [M9NE07_DROME]	2.03	7.35	2	1	1	1	1.614E7	204	21.6	8.09

Accession	Description	Score	Coverage	# Proteins	# Unique Peptides	# Peptides	# PSMs	Area	# AAs	MW [kDa]	calc. pI
Q9V3Q4	CG1274-PA, isoform A OS=Drosophila melanogaster GN=Jafrac2 PE=2 SV=1 - [Q9V3Q4_DROME]	2.03	7.44	1	1	1	2	1.823E7	242	26.7	6.80
M9NEW0	CG43346, isoform A OS=Drosophila melanogaster GN=CG43346 PE=2 SV=1 - [M9NEW0_DROME]	2.03	8.00	1	1	1	1		150	17.6	8.95
Q9VSL6	Glutathione S transferase O1 OS=Drosophila melanogaster GN=GstO1 PE=2 SV=1 - [Q9VSL6_DROME]	2.03	4.33	1	1	1	1	2.233E7	254	30.0	7.44
E1JHV7	Enhancer of bithorax, isoform F OS=Drosophila melanogaster GN=E(bx) PE=4 SV=1 - [E1JHV7_DROME]	2.03	0.65	5	1	1	1	5.022E6	2139	239.3	9.09
Q9VYY3	Ubiquitin-like modifier-activating enzyme 5 OS=Drosophila melanogaster GN=CG1749 PE=2 SV=1 - [UBA5_DROME]	2.02	4.70	1	1	1	1	1.245E7	404	44.1	4.73
Q86D34	CG33203, isoform B OS=Drosophila melanogaster GN=CG14522 PE=2 SV=1 - [Q86D34_DROME]	2.02	6.44	2	1	1	1		450	47.8	6.96
Q95U20	GH13793p OS=Drosophila melanogaster GN=Opa1 PE=2 SV=1 - [Q95U20_DROME]	2.02	1.50	2	1	1	1	8.040E7	933	107.1	8.94
Q95T12	Calcium channel flower OS=Drosophila melanogaster GN=fwe PE=1 SV=1 - [FLOWR_DROME]	2.02	9.28	1	1	1	1	1.337E7	194	20.6	6.51
O18405	Surfeit locus protein 4 homolog OS=Drosophila melanogaster GN=Surf4 PE=2 SV=1 - [SURF4_DROME]	2.02	4.07	1	1	1	1	1.991E7	270	30.5	8.97
Q9VW09	E3 ubiquitin-protein ligase listerin OS=Drosophila melanogaster GN=l(3)76BDr PE=1 SV=2 - [LTN1_DROME]	2.01	0.74	1	1	1	1		1747	198.5	5.94
A1Z9J6	Mitochondrial ribosomal protein L53 OS=Drosophila melanogaster GN=mRpL53 PE=2 SV=1 - [A1Z9J6_DROME]	2.01	5.16	1	1	1	1	1.547E7	155	16.9	10.01
Q8T481	AT11343p OS=Drosophila melanogaster GN=CG32022 PE=2 SV=1 - [Q8T481_DROME]	2.01	9.55	1	1	1	1		178	19.8	9.85
Q960Z4	Arrest, isoform E OS=Drosophila melanogaster GN=aret PE=2 SV=1 - [Q960Z4_DROME]	2.01	7.62	5	3	3	3	1.751E7	604	64.1	7.65
Q9V3F9	BcDNA.LD28120 OS=Drosophila melanogaster GN=out PE=2 SV=1 - [Q9V3F9_DROME]	2.00	1.98	1	1	1	1	2.469E6	655	72.9	8.91
Q9VE94	CG18600 OS=Drosophila melanogaster GN=CG18600 PE=2 SV=1 - [Q9VE94_DROME]	2.00	2.34	1	1	1	1		384	43.7	8.24
Q9W3T2	CG4593, isoform A OS=Drosophila melanogaster GN=CG4593-RA PE=2 SV=1 - [Q9W3T2_DROME]	2.00	14.83	1	2	2	4	5.752E7	209	24.8	6.57
Q9VAW3	GH12731p OS=Drosophila melanogaster GN=Gfat2 PE=2 SV=1 - [Q9VAW3_DROME]	1.99	1.46	1	1	1	1	7.974E6	683	76.5	6.62
A1Z6H4	RE52822p OS=Drosophila melanogaster GN=vlc PE=2 SV=1 - [A1Z6H4_DROME]	1.98	2.57	3	1	1	1	1.630E7	583	63.6	6.21
X2JF15	Radish, isoform L OS=Drosophila melanogaster GN=rad PE=4 SV=1 - [X2JF15_DROME]	1.98	0.80	5	1	1	1	1.224E7	1630	183.8	8.31
Q9VVU6	CG6841-PA OS=Drosophila melanogaster GN=CG6841 PE=2 SV=2 - [Q9VVU6_DROME]	1.98	1.83	1	1	1	1		931	105.2	7.44
A8Y535	Cytochrome b-c1 complex subunit 6 OS=Drosophila melanogaster GN=Ucrh PE=3 SV=2 - [A8Y535_DROME]	1.98	10.59	1	1	1	1	5.696E7	85	9.9	5.21
O44437	Small nuclear ribonucleoprotein Sm D3 OS=Drosophila melanogaster GN=SmD3 PE=1 SV=1 - [SMD3_DROME]	1.97	6.62	1	1	1	1	1.783E8	151	15.6	10.54
Q7KMJ6	BcDNA.GH11110 OS=Drosophila melanogaster GN=nito PE=2 SV=1 - [Q7KMJ6_DROME]	1.96	1.51	1	1	1	1	9.659E6	793	89.0	9.38
Q9VC06	CG11771 OS=Drosophila melanogaster GN=CG11771 PE=2 SV=1 - [Q9VC06_DROME]	1.96	1.37	1	1	1	1	2.565E7	730	81.3	7.08
Q9VQ35	CG17642-PA OS=Drosophila melanogaster GN=mRpL48 PE=2 SV=1 - [Q9VQ35_DROME]	1.96	6.63	1	1	1	1	6.126E6	181	20.9	5.47
Q9VBG6	Protein HGH1 homolog OS=Drosophila melanogaster GN=CG6073 PE=2 SV=2 - [HGH1_DROME]	1.96	2.17	1	1	1	1	1.131E8	369	41.8	4.97
M9PC65	CG34126, isoform D OS=Drosophila melanogaster GN=CG34126 PE=4 SV=1 - [M9PC65_DROME]	1.95	0.54	5	1	1	1	1.142E8	1475	161.8	6.81
Q7KTW9	Asparagine synthetase OS=Drosophila melanogaster GN=asparagine-synthetase PE=2 SV=1 - [Q7KTW9_DROME]	1.95	3.05	1	1	1	1		558	63.2	6.49
Q9VG76	C-Myc-binding protein homolog OS=Drosophila melanogaster GN=CG17202 PE=2 SV=2 - [MYCBP_DROME]	1.95	3.87	1	1	1	1	1.832E7	181	20.1	4.68
Q7JVG6	CG1550 OS=Drosophila melanogaster GN=CG1550 PE=2 SV=1 - [Q7JVG6_DROME]	1.95	1.92	1	1	1	1	8.447E6	626	72.5	5.16
Q9VA81	IP02765p OS=Drosophila melanogaster GN=mRpS18C PE=2 SV=2 - [Q9VA81_DROME]	1.95	5.00	1	1	1	1	8.683E6	140	16.2	9.07
Q8MSU4	Symplekin OS=Drosophila melanogaster GN=Sym PE=1 SV=1 - [SYMPK_DROME]	1.95	0.86	1	1	1	1		1165	132.0	6.49
Q9VVV7	CG3808 OS=Drosophila melanogaster GN=CG3808 PE=4 SV=3 - [Q9VVV7_DROME]	1.95	1.95	1	1	1	1		615	68.6	6.23
Q7K2B0	Ribosomal RNA-processing protein 8 OS=Drosophila melanogaster GN=CG7137 PE=1 SV=1 - [RRP8_DROME]	1.94	3.35	1	1	1	1	2.371E7	358	40.3	9.57
Q9VSL4	Glutathione S transferase O2, isoform B OS=Drosophila melanogaster GN=GstO2 PE=4 SV=2 - [Q9VSL4_DROME]	1.94	6.80	1	2	2	2		250	28.7	7.05
P91927	LETM1 and EF-hand domain-containing protein anon-60Da, mitochondrial OS=Drosophila melanogaster GN=Letm1 PE=2 SV=2 - [A60DA_DROME]	1.93	1.48	1	1	1	1		1013	113.5	6.84
Q8WTC1	28S ribosomal protein S15, mitochondrial OS=Drosophila melanogaster GN=bonsai PE=2 SV=2 - [RT15_DROME]	1.93	3.93	1	1	1	1	5.954E7	280	33.5	9.20
Q9VVM8	CG5290-PA OS=Drosophila melanogaster GN=CG5290-RA PE=2 SV=1 - [Q9VVM8_DROME]	1.93	1.75	1	2	2	2	3.740E7	798	92.1	6.06
M9PE02	Female sterile (1) homeotic, isoform H OS=Drosophila melanogaster GN=fs(1)h PE=4 SV=1 - [M9PE02_DROME]	1.92	2.17	5	1	1	1		1105	109.9	9.29
O77434	EG:34F3.8 protein OS=Drosophila melanogaster GN=Sec22 PE=2 SV=2 - [O77434_DROME]	1.91	6.64	2	1	1	1	6.299E6	211	24.6	9.29
A4V1Y7	Trithorax-like, isoform C OS=Drosophila melanogaster GN=Trl PE=4 SV=1 - [A4V1Y7_DROME]	1.91	2.89	6	1	1	1		519	54.8	8.03
Q03042	cGMP-dependent protein kinase, isozyme 1 OS=Drosophila melanogaster GN=Pkg21D PE=1 SV=2 - [KGP1_DROME]	1.91	2.21	1	1	1	1		768	86.7	6.42
Q9VVK7	NUCB1 OS=Drosophila melanogaster GN=NUCB1 PE=4 SV=3 - [Q9VVK7_DROME]	1.90	4.57	1	2	2	2	5.796E7	569	67.3	5.30
Q9VGW4	CG14692 OS=Drosophila melanogaster GN=CG14692 PE=4 SV=3 - [Q9VGW4_DROME]	1.89	0.98	1	2	2	2	1.955E7	2743	306.8	4.93
Q23973	Protein matrimony OS=Drosophila melanogaster GN=mtrm PE=1 SV=2 - [MTRM_DROME]	1.89	5.99	1	1	1	1	7.872E6	217	24.8	8.56
Q9W127	CG3663 OS=Drosophila melanogaster GN=CG3663 PE=2 SV=1 - [Q9W127_DROME]	1.89	5.29	1	1	1	1	6.045E7	208	23.3	7.69
Q9W328	Protein LST8 homolog OS=Drosophila melanogaster GN=Lst8 PE=2 SV=2 - [LST8_DROME]	1.89	3.19	1	1	1	1	3.646E7	313	35.3	7.17
Q9VJ11	CG10470-PA OS=Drosophila melanogaster GN=CG10470 PE=2 SV=1 - [Q9VJ11_DROME]	1.88	4.37	1	1	1	1		183	20.8	9.50
Q9VTT2	CG6801-PA OS=Drosophila melanogaster GN=l(3)j2D3 PE=2 SV=1 - [Q9VTT2_DROME]	1.88	2.30	1	1	1	1	3.650E9	391	44.8	5.81
Q7KNS3	Lissencephaly-1 homolog OS=Drosophila melanogaster GN=Lis-1 PE=1 SV=2 - [LIS1_DROME]	1.88	2.68	1	1	1	1	3.157E7	411	46.4	7.56

Accession	Description	Score	Coverage	# Proteins	# Unique Peptides	# Peptides	# PSMs	Area	# AAs	MW [kDa]	calc. pI
Q9VVZ4	CG14095-PA OS=Drosophila melanogaster GN=CG14095 PE=4 SV=1 - [Q9VVZ4_DROME]	1.88	44.44	1	3	3	5	9.140E7	162	15.9	9.92
Q9Y0I1	NuA4 complex subunit EAF3 homolog OS=Drosophila melanogaster GN=MRG15 PE=1 SV=1 - [EAF3_DROME]	1.88	3.07	1	1	1	1	3.642E7	424	47.2	8.13
Q9VH85	CG12950 OS=Drosophila melanogaster GN=CG12950 PE=1 SV=2 - [Q9VH85_DROME]	1.87	1.17	1	1	1	1	6.774E7	939	102.8	6.64
P40793	Cdc42 homolog OS=Drosophila melanogaster GN=Cdc42 PE=1 SV=1 - [CDC42_DROME]	1.87	11.52	1	1	2	2	1.422E8	191	21.4	7.14
Q9VJ58	Fas-associated factor, isoform A OS=Drosophila melanogaster GN=Faf PE=2 SV=1 - [Q9VJ58_DROME]	1.87	3.45	1	1	1	1	6.426E6	464	52.9	5.38
Q7K010	Tetraspanin OS=Drosophila melanogaster GN=Tsp42Ef PE=2 SV=1 - [Q7K010_DROME]	1.87	5.45	1	1	1	1		220	24.7	5.86
O01939	Protein misato OS=Drosophila melanogaster GN=mst PE=2 SV=1 - [MST_DROME]	1.87	1.74	1	1	1	1	8.913E6	574	64.7	5.03
Q9V998	Ubiquitin-like protein 5 OS=Drosophila melanogaster GN=ubl PE=3 SV=1 - [UBL5_DROME]	1.87	16.44	1	1	1	1	4.208E7	73	8.6	8.43
Q9VM18	Trehalose 6-phosphate phosphatase OS=Drosophila melanogaster GN=CG5177 PE=2 SV=1 - [Q9VM18_DROME]	1.86	9.06	1	2	2	2	2.399E7	276	31.2	7.12
Q9V3U2	H/ACA ribonucleoprotein complex subunit 2-like protein OS=Drosophila melanogaster GN=NHP2 PE=1 SV=1 - [NHP2_DROME]	1.86	6.25	1	1	1	2	3.464E8	160	17.7	8.10
Q7K486	Armadillo repeat-containing protein 6 homolog OS=Drosophila melanogaster GN=CG5721 PE=1 SV=1 - [ARMC6_DROME]	1.85	2.16	1	1	1	1	3.235E7	464	51.3	5.55
D2A6L6	CG41056 (Fragment) OS=Drosophila melanogaster GN=CG41056 PE=4 SV=1 - [D2A6L6_DROME]	1.85	1.06	1	1	1	1	1.849E7	755	87.7	9.14
Q9VDC9	CG5871 OS=Drosophila melanogaster GN=Oga PE=2 SV=1 - [Q9VDC9_DROME]	1.85	1.18	1	1	1	1		1019	113.8	4.88
Q9VC48	Methionine aminopeptidase 1 OS=Drosophila melanogaster GN=CG13630 PE=2 SV=1 - [Q9VC48_DROME]	1.84	2.41	1	1	1	1	4.478E7	374	41.7	6.93
M9PBS9	CG10103, isoform F OS=Drosophila melanogaster GN=CG10103 PE=4 SV=1 - [M9PBS9_DROME]	1.83	3.06	6	1	1	1		327	35.6	5.88
P82711	Probable cytochrome P450 6a19 OS=Drosophila melanogaster GN=Cyp6a19 PE=3 SV=1 - [C6A19_DROME]	1.83	2.39	1	1	1	1	3.582E6	503	57.7	8.94
Q8T007	CG6791, isoform B OS=Drosophila melanogaster GN=CG6791 PE=2 SV=1 - [Q8T007_DROME]	1.83	0.88	1	1	1	1		1243	143.1	6.39
A8JQT5	CG34306 OS=Drosophila melanogaster GN=Cdep PE=4 SV=1 - [A8JQT5_DROME]	1.82	0.46	1	2	2	2	3.793E7	3671	405.9	5.78
Q8I0S9	CG2118, isoform B OS=Drosophila melanogaster GN=CG2118 PE=2 SV=1 - [Q8I0S9_DROME]	1.82	1.74	2	1	1	1		634	69.4	5.52
P54358	DNA polymerase delta catalytic subunit OS=Drosophila melanogaster GN=DNApol-delta PE=2 SV=2 - [DPOD1_DROME]	1.82	1.10	1	1	1	1	4.760E6	1092	124.8	6.92
Q7K2P3	CG1648, isoform A OS=Drosophila melanogaster GN=CG1648 PE=2 SV=1 - [Q7K2P3_DROME]	1.82	12.61	2	2	2	2	9.051E8	230	23.8	8.91
Q0E9F9	CG2915, isoform A OS=Drosophila melanogaster GN=CG2915-RB PE=2 SV=1 - [Q0E9F9_DROME]	1.82	1.99	1	1	1	1		453	51.0	6.13
A1Z9K8	LD13733p OS=Drosophila melanogaster GN=Prosap PE=2 SV=2 - [A1Z9K8_DROME]	1.81	0.37	1	1	1	1	5.980E7	1871	198.9	8.44
Q9W149	CG11414 OS=Drosophila melanogaster GN=CG11414-RA PE=2 SV=1 - [Q9W149_DROME]	1.81	1.38	1	1	1	1		867	95.9	8.82
Q9V3D0	LD33485p OS=Drosophila melanogaster GN=mRpL4 PE=2 SV=1 - [Q9V3D0_DROME]	1.81	5.74	1	1	1	2		296	32.8	9.22
Q9W3U0	Acyl-coenzyme A oxidase OS=Drosophila melanogaster GN=CG4586 PE=2 SV=1 - [Q9W3U0_DROME]	1.81	4.58	1	2	2	2		677	76.6	8.91
Q9VFC8	Glycogen [starch] synthase OS=Drosophila melanogaster GN=GlyS PE=1 SV=2 - [GYS_DROME]	1.81	1.69	1	1	1	1	4.883E6	709	81.7	6.65
P23647	Serine/threonine-protein kinase fused OS=Drosophila melanogaster GN=fu PE=1 SV=2 - [FUSED_DROME]	1.81	2.73	17	2	2	2	1.206E7	805	90.3	6.67
B7YZH7	CG8446, isoform D OS=Drosophila melanogaster GN=CG8446-RD PE=2 SV=1 - [B7YZH7_DROME]	1.81	25.71	1	1	1	1		70	7.4	9.70
Q59DZ1	CG42313, isoform A OS=Drosophila melanogaster GN=CG42313 PE=1 SV=3 - [Q59DZ1_DROME]	1.81	1.41	1	1	1	2	2.000E7	1064	114.0	7.33
Q9W2E7	Rae1 OS=Drosophila melanogaster GN=Rae1 PE=4 SV=1 - [Q9W2E7_DROME]	1.81	3.47	1	1	1	1	9.016E7	346	38.6	7.58
Q9VJ12	Acinus, isoform A OS=Drosophila melanogaster GN=Acn PE=1 SV=1 - [Q9VJ12_DROME]	1.80	1.76	1	1	1	1	1.099E8	739	83.7	6.70
Q9VQY9	Probable DNA replication complex GINS protein PSF2 OS=Drosophila melanogaster GN=Psf2 PE=2 SV=1 - [PSF2_DROME]	1.80	7.88	1	1	1	1		203	23.1	6.39
Q9VJ31	CG10623 protein OS=Drosophila melanogaster GN=CG10623 PE=2 SV=1 - [Q9VJ31_DROME]	1.79	9.06	1	2	2	2	8.363E6	331	36.7	5.11
Q9VIM0	CG2493, isoform A OS=Drosophila melanogaster GN=CG2493 PE=2 SV=1 - [Q9VIM0_DROME]	1.79	2.32	1	1	1	1	3.230E7	475	53.7	5.71
Q9VM75	HEAT repeat-containing protein 1 homolog OS=Drosophila melanogaster GN=l(2)k09022 PE=2 SV=2 - [HEAT1_DROME]	1.78	1.81	1	3	3	3	1.645E6	2096	237.1	6.87
Q9VGP6	Prefoldin subunit 3 OS=Drosophila melanogaster GN=mgr PE=1 SV=3 - [PFD3_DROME]	1.78	4.64	1	1	1	1	9.124E7	194	22.3	5.11
P55824	Probable ubiquitin carboxyl-terminal hydrolase FAF OS=Drosophila melanogaster GN=faf PE=1 SV=2 - [FAF_DROME]	1.78	0.43	1	1	1	1	3.464E7	2778	310.9	6.05
Q9W394	CG32708-PA OS=Drosophila melanogaster GN=CG32708 PE=4 SV=1 - [Q9W394_DROME]	1.78	3.77	1	1	1	1		239	27.5	9.77
Q9VS83	CG7546, isoform B OS=Drosophila melanogaster GN=CG7546 PE=2 SV=3 - [Q9VS83_DROME]	1.77	1.72	8	2	2	2	3.253E7	1166	124.3	5.14
Q9VYA7	RRP12-like protein OS=Drosophila melanogaster GN=CG2691 PE=1 SV=2 - [RRP12_DROME]	1.77	3.25	1	3	3	3	3.378E7	1384	153.5	8.78
Q9VHX2	CG3223 OS=Drosophila melanogaster GN=CG3223 PE=2 SV=1 - [Q9VHX2_DROME]	1.77	3.13	1	1	1	1	9.890E6	415	45.1	5.25
B7Z107	CG42376, isoform A OS=Drosophila melanogaster GN=cin-RB PE=2 SV=1 - [B7Z107_DROME]	1.77	9.78	1	1	1	1		92	11.0	8.69
Q9VXK5	Putative rRNA methyltransferase OS=Drosophila melanogaster GN=CG8939 PE=2 SV=1 - [Q9VXK5_DROME]	1.75	1.84	1	1	1	1		817	93.3	9.04
Q9VGK7	Putative elongator complex protein 1 OS=Drosophila melanogaster GN=Elp1 PE=1 SV=2 - [ELP1_DROME]	1.74	0.88	1	1	1	1	9.191E6	1252	142.7	6.43
Q7K533	CG11395 OS=Drosophila melanogaster GN=CG11395 PE=2 SV=1 - [Q7K533_DROME]	1.74	3.07	1	1	1	1	1.731E7	456	48.8	6.73
Q9W270	Organic anion transporting polypeptide 58Db OS=Drosophila melanogaster GN=Oatp58Db PE=4 SV=2 - [Q9W270_DROME]	1.74	0.97	1	1	1	1	2.842E7	722	80.1	7.90
A1Z803	CG1665 OS=Drosophila melanogaster GN=CG1665-RA PE=2 SV=1 - [A1Z803_DROME]	1.74	2.94	1	1	1	1	1.535E7	340	38.1	8.54
P32866	G protein-coupled receptor kinase 2 OS=Drosophila melanogaster GN=Gprk2 PE=1 SV=3 - [GPRK2_DROME]	1.73	4.48	1	2	2	2	3.037E7	714	80.6	6.35
Q9U9Q1	Replication factor C 38kD subunit, isoform A OS=Drosophila melanogaster GN=RfC38 PE=4 SV=1 - [Q9U9Q1_DROME]	1.73	3.93	1	1	1	1	3.366E7	356	40.8	8.40

Accession	Description	Score	Coverage	# Proteins	# Unique Peptides	# Peptides	# PSMs	Area	# AAs	MW [kDa]	calc. pI
F2FBA6	Dorsal switch protein 1, isoform G OS=Drosophila melanogaster GN=Dsp1-RC PE=2 SV=1 - [F2FBA6_DROME]	1.73	3.37	3	1	1	2	4.617E7	386	44.0	8.43
Q9W4Z5	CG2854-PA OS=Drosophila melanogaster GN=EG:BACH48C10.2 PE=2 SV=2 - [Q9W4Z5_DROME]	1.73	2.24	1	1	1	1	1.238E7	491	55.5	8.63
Q9W4P5	V-type proton ATPase subunit d 1 OS=Drosophila melanogaster GN=VhaAC39-1 PE=2 SV=1 - [VA0D1_DROME]	1.73	3.14	1	1	1	1		350	39.8	4.91
Q8MYL1	Fancd2 OS=Drosophila melanogaster GN=Fancd2 PE=2 SV=1 - [Q8MYL1_DROME]	1.73	0.47	1	1	1	1	4.592E6	1478	167.4	6.89
Q9VAJ1	Condensin complex subunit 1 OS=Drosophila melanogaster GN=Cap-D2 PE=2 SV=1 - [Q9VAJ1_DROME]	1.73	0.51	1	1	1	1		1380	157.4	5.86
Q9VHB8	CG8199 OS=Drosophila melanogaster GN=CG8199 PE=2 SV=1 - [Q9VHB8_DROME]	1.73	12.98	1	3	3	3	2.919E6	439	49.7	6.65
Q86B87	Modifier of mdg4 OS=Drosophila melanogaster GN=mod(mdg4) PE=1 SV=1 - [MMD4_DROME]	1.72	1.64	1	1	1	1	5.511E6	610	67.1	5.07
Q9VB05	ALG-2 interacting protein X OS=Drosophila melanogaster GN=ALiX PE=1 SV=1 - [Q9VB05_DROME]	1.72	5.02	1	3	3	3	1.257E7	836	92.5	5.45
Q961F9	CG4729-PD, isoform D OS=Drosophila melanogaster GN=CG4729 PE=2 SV=1 - [Q961F9_DROME]	1.72	6.29	3	1	1	1	1.452E7	143	16.1	8.85
Q9VBX4	CG31510 OS=Drosophila melanogaster GN=CG31510-RA PE=2 SV=2 - [Q9VBX4_DROME]	1.72	1.30	1	1	1	1	7.030E6	1150	129.2	9.57
Q6NLM8	RE56731p OS=Drosophila melanogaster GN=sano PE=2 SV=1 - [Q6NLM8_DROME]	1.72	1.67	1	1	1	2	9.458E6	778	84.8	9.72
Q8SZW5	Alpha-Esterase-3, isoform A OS=Drosophila melanogaster GN=alpha-Est3 PE=2 SV=1 - [Q8SZW5_DROME]	1.72	2.39	1	1	1	1		543	61.4	6.80
Q9VDE9	FI04035p OS=Drosophila melanogaster GN=RhoGAP93B PE=2 SV=2 - [Q9VDE9_DROME]	1.72	1.43	1	1	1	1	2.919E7	1330	146.2	8.34
Q9VMY1	Probable 39S ribosomal protein L24, mitochondrial OS=Drosophila melanogaster GN=mRpL24 PE=1 SV=1 - [RM24_DROME]	1.72	3.24	1	1	1	1		247	29.2	9.38
Q960X8	Hepatocyte growth factor-regulated tyrosine kinase substrate OS=Drosophila melanogaster GN=Hrs PE=1 SV=1 - [HRS_DROME]	1.71	1.71	1	1	1	1		760	85.4	6.98
Q2PDP2	AT24894p OS=Drosophila melanogaster GN=CG31693 PE=2 SV=1 - [Q2PDP2_DROME]	1.71	1.65	2	1	1	1		607	67.0	6.16
Q9VBK9	CG5913 OS=Drosophila melanogaster GN=CG5913 PE=4 SV=1 - [Q9VBK9_DROME]	1.70	2.20	1	1	1	1	2.003E6	454	49.9	9.70
Q9VW53	CG8025-PA OS=Drosophila melanogaster GN=Mtr3 PE=2 SV=1 - [Q9VW53_DROME]	1.69	3.07	1	1	1	1	6.154E8	326	36.6	5.21
Q9VM58	CG10399, isoform A OS=Drosophila melanogaster GN=CG10399 PE=2 SV=2 - [Q9VM58_DROME]	1.69	4.02	1	1	1	1	1.251E7	323	34.2	8.40
Q9W1Y0	CG3499 OS=Drosophila melanogaster GN=CG3499 PE=2 SV=1 - [Q9W1Y0_DROME]	1.69	5.03	1	3	3	3	1.587E7	736	80.6	8.57
Q8IMG3	CG1815, isoform B OS=Drosophila melanogaster GN=CG1815 PE=4 SV=1 - [Q8IMG3_DROME]	1.69	1.16	3	1	1	1		1121	124.9	7.69
Q8MR62	Viral IAP-associated factor homolog OS=Drosophila melanogaster GN=viaf PE=1 SV=1 - [VIAF1_DROME]	1.68	5.42	1	1	1	1		240	27.4	4.86
Q9VK44	CG9934, isoform A OS=Drosophila melanogaster GN=CG9934 PE=2 SV=1 - [Q9VK44_DROME]	1.68	1.23	1	1	1	1		1217	138.5	5.87
Q8SY19	CG1742-PA, isoform A OS=Drosophila melanogaster GN=Mgstl PE=2 SV=1 - [Q8SY19_DROME]	1.68	7.89	1	1	1	2	3.901E7	152	16.9	9.99
Q9VK28	CG16974, isoform A OS=Drosophila melanogaster GN=CG16974 PE=2 SV=1 - [Q9VK28_DROME]	1.68	2.07	1	2	2	2		1257	143.0	7.14
Q9W2Y4	CG2972, isoform B OS=Drosophila melanogaster GN=CG2972 PE=2 SV=2 - [Q9W2Y4_DROME]	1.68	1.69	1	1	1	1	9.139E6	472	53.7	4.92
A1ZBR2	18 wheeler OS=Drosophila melanogaster GN=18w PE=4 SV=1 - [A1ZBR2_DROME]	1.68	4.69	1	3	3	3	5.368E7	1385	154.7	6.46
Q8MLW2	Domino, isoform D OS=Drosophila melanogaster GN=dom PE=4 SV=1 - [Q8MLW2_DROME]	1.68	0.50	2	1	1	2	5.884E6	3183	348.2	8.87
A4V2A4	Mushroom-body expressed, isoform G OS=Drosophila melanogaster GN=mub PE=4 SV=1 - [A4V2A4_DROME]	1.68	14.48	9	3	3	5	5.195E7	359	37.9	8.51
D0Z726	MIP14975p OS=Drosophila melanogaster GN=Ntf-2 PE=2 SV=1 - [D0Z726_DROME]	1.67	8.99	4	1	1	2	2.115E8	89	9.9	6.93
Q9VCH8	LD23303p OS=Drosophila melanogaster GN=tst PE=2 SV=2 - [Q9VCH8_DROME]	1.66	1.34	1	1	1	1	1.306E8	1197	135.7	7.01
Q9Y162	BcDNA.GH02678 OS=Drosophila melanogaster GN=Vps4 PE=2 SV=1 - [Q9Y162_DROME]	1.66	2.49	1	1	1	1	3.076E7	442	49.6	6.96
Q9VES8	CG10340 OS=Drosophila melanogaster GN=CG10340 PE=2 SV=3 - [Q9VES8_DROME]	1.66	3.60	1	1	1	1	2.191E7	278	32.0	6.55
Q9VAJ2	Bub3 OS=Drosophila melanogaster GN=Bub3 PE=2 SV=1 - [Q9VAJ2_DROME]	1.66	4.29	1	1	1	1		326	37.4	6.54
P47949	Troponin C, isoform 3 OS=Drosophila melanogaster GN=TpnC73F PE=2 SV=2 - [TNNC3_DROME]	1.65	47.74	2	6	6	7	1.088E8	155	17.7	4.18
Q94546	Another transcription unit protein OS=Drosophila melanogaster GN=Atu PE=1 SV=2 - [ATU_DROME]	1.65	0.97	1	1	1	1		725	77.4	10.21
Q8IPT1	CG32441, isoform D OS=Drosophila melanogaster GN=CG32441 PE=4 SV=2 - [Q8IPT1_DROME]	1.65	3.74	2	1	1	1	1.298E7	214	23.7	5.26
Q9W2K8	CG9418 OS=Drosophila melanogaster GN=Hmg-2 PE=2 SV=1 - [Q9W2K8_DROME]	1.65	2.39	1	1	1	1	2.853E7	376	41.7	9.38
Q7KVA1	Xylosyltransferase oxt OS=Drosophila melanogaster GN=oxt PE=2 SV=1 - [XYLT_DROME]	1.64	1.26	1	1	1	1		876	99.0	8.10
E1JJF9	Neuroglian, isoform D OS=Drosophila melanogaster GN=Nrg PE=4 SV=1 - [E1JJF9_DROME]	1.64	1.94	3	2	2	2	1.105E7	1239	138.3	5.49
Q9VEP9	CG16941 OS=Drosophila melanogaster GN=CG16941 PE=2 SV=1 - [Q9VEP9_DROME]	1.64	1.40	1	1	1	1	2.962E7	784	88.0	6.37
A8DY82	Not1, isoform E OS=Drosophila melanogaster GN=Not1 PE=4 SV=1 - [A8DY82_DROME]	1.64	0.99	4	2	2	2	1.292E7	2220	249.6	6.96
Q9W199	Mitochondrial ribosomal protein S17 OS=Drosophila melanogaster GN=mRpS17 PE=2 SV=1 - [Q9W199_DROME]	1.64	5.16	1	1	1	1	1.506E7	155	18.0	8.75
Q9VG73	Interleukin enhancer-binding factor 2 homolog OS=Drosophila melanogaster GN=CG5641 PE=2 SV=1 - [ILF2_DROME]	1.63	4.55	1	1	1	2	7.338E6	396	43.6	6.93
Q7K0H4	SD07723p OS=Drosophila melanogaster GN=stj PE=2 SV=1 - [Q7K0H4_DROME]	1.63	0.90	1	1	1	1	8.390E6	1218	140.0	6.64
Q6IGW6	CG34200 OS=Drosophila melanogaster GN=CG34200 PE=4 SV=1 - [Q6IGW6_DROME]	1.63	17.31	1	1	1	1	5.254E7	52	5.9	9.42
Q9W0M1	CG13889, isoform A OS=Drosophila melanogaster GN=CG13889 PE=4 SV=2 - [Q9W0M1_DROME]	1.63	0.35	1	1	1	1		1978	227.8	5.54
Q94517	Histone deacetylase Rpd3 OS=Drosophila melanogaster GN=Rpd3 PE=1 SV=2 - [HDAC1_DROME]	1.63	1.54	1	1	1	1	2.046E7	521	58.3	5.76
Q9W1C5	CG3173 OS=Drosophila melanogaster GN=IntS1 PE=4 SV=2 - [Q9W1C5_DROME]	1.62	1.12	1	2	2	2	1.035E8	2053	234.9	6.61
Q9V3Y5	BcDNA.LD23634 OS=Drosophila melanogaster GN=BcDNA.LD23634 PE=2 SV=1 - [Q9V3Y5_DROME]	1.62	1.20	1	1	1	1		998	112.6	6.49

Accession	Description	Score	Coverage	# Proteins	# Unique Peptides	# Peptides	# PSMs	Area	# AAs	MW [kDa]	calc. pI
Q9VXJ8	CG8958 OS=Drosophila melanogaster GN=CG8958 PE=4 SV=2 - [Q9VXJ8_DROME]	1.62	4.19	1	2	2	2	2.100E7	621	71.0	5.74
Q9VL63	UPF0430 protein CG31712 OS=Drosophila melanogaster GN=CG31712 PE=1 SV=3 - [U430_DROME]	1.62	2.41	1	1	1	1	1.374E6	290	35.5	9.91
X2JE40	Rho kinase, isoform C OS=Drosophila melanogaster GN=Rok PE=4 SV=1 - [X2JE40_DROME]	1.62	1.02	4	1	1	1	2.759E7	1374	158.5	6.47
Q9VPL3	39S ribosomal protein L10, mitochondrial OS=Drosophila melanogaster GN=mRpL10 PE=2 SV=1 - [RM10_DROME]	1.62	6.05	1	1	1	1	1.156E7	248	28.3	10.07
Q8MSW9	CG33505 OS=Drosophila melanogaster GN=U3-55K PE=2 SV=1 - [Q8MSW9_DROME]	1.62	2.48	1	1	1	1	2.166E7	484	53.3	8.73
P92208	Stress-activated protein kinase JNK OS=Drosophila melanogaster GN=bsk PE=1 SV=1 - [JNK_DROME]	1.61	2.15	3	1	1	1		372	43.0	6.42
Q7K2S6	CG11509-PA OS=Drosophila melanogaster GN=CG11509 PE=2 SV=1 - [Q7K2S6_DROME]	1.61	5.13	1	1	1	1	3.083E8	117	13.5	10.14
Q9W252	DNA repair protein RAD50 OS=Drosophila melanogaster GN=rad50 PE=2 SV=4 - [RAD50_DROME]	1.61	4.02	1	2	2	2	3.642E6	1318	152.1	7.58
Q9VYI3	Probable 39S ribosomal protein L49, mitochondrial OS=Drosophila melanogaster GN=mRpL49 PE=3 SV=1 - [RM49_DROME]	1.61	5.59	1	1	1	1	3.236E7	179	20.6	9.63
Q0KHT4	CG2371, isoform A OS=Drosophila melanogaster GN=CG2371 PE=4 SV=1 - [Q0KHT4_DROME]	1.61	2.99	4	1	1	1	3.304E7	201	22.6	4.82
Q9VZ45	Myosin 10A, isoform C OS=Drosophila melanogaster GN=Myo10A PE=4 SV=3 - [Q9VZ45_DROME]	1.60	0.49	2	1	1	1		3054	339.0	8.13
Q9VZI2	Activated Cdc42 kinase OS=Drosophila melanogaster GN=Ack PE=2 SV=2 - [Q9VZI2_DROME]	1.60	0.75	1	1	1	1	1.459E7	1073	118.3	7.23
Q9VRN4	Blimp-1, isoform A OS=Drosophila melanogaster GN=Blimp-1 PE=2 SV=1 - [Q9VRN4_DROME]	1.60	1.50	2	1	1	1		1203	133.2	7.39
P12426	Adenine phosphoribosyltransferase OS=Drosophila melanogaster GN=Aprt PE=2 SV=3 - [APT_DROME]	0.00	4.95	1	1	1	1	1.089E7	182	19.7	5.69
P45888	Actin-related protein 2 OS=Drosophila melanogaster GN=Arp2 PE=2 SV=3 - [ARP2_DROME]	0.00	3.01	1	1	1	2	3.189E7	399	45.3	6.02
Q7JWD3	ATPase ASNA1 homolog OS=Drosophila melanogaster GN=CG1598 PE=2 SV=1 - [ASNA_DROME]	0.00	5.65	1	2	2	3	1.345E7	336	37.6	5.02
Q5EAK6	Serine/threonine-protein kinase ATM OS=Drosophila melanogaster GN=tefu PE=2 SV=1 - [ATM_DROME]	0.00	0.54	1	1	1	1		2767	317.8	7.36
Q9VKN7	Aurora kinase B OS=Drosophila melanogaster GN=ial PE=1 SV=1 - [AURKB_DROME]	0.00	6.08	1	1	1	1		329	38.3	8.78
P29746	Protein bangles and beads OS=Drosophila melanogaster GN=bnb PE=1 SV=1 - [BNB_DROME]	0.00	2.71	1	1	1	1	5.928E7	442	45.8	4.63
Q9VZE6	Ribosome biogenesis protein BRX1 homolog OS=Drosophila melanogaster GN=CG11583 PE=2 SV=2 - [BRX1_DROME]	0.00	7.52	1	2	2	6	1.536E7	359	41.6	9.89
Q9VE01	Probable cytochrome P450 12a5, mitochondrial OS=Drosophila melanogaster GN=Cyp12a5 PE=2 SV=1 - [C12A5_DROME]	0.00	2.99	1	1	1	1		536	61.3	9.20
Q03017	NF-kappa-B inhibitor cactus OS=Drosophila melanogaster GN=cact PE=1 SV=2 - [CACT_DROME]	0.00	2.60	1	1	1	1	1.435E6	500	53.8	4.84
P09085	Homeotic protein caudal OS=Drosophila melanogaster GN=cad PE=1 SV=4 - [CAD_DROME]	0.00	5.62	2	1	1	1		427	45.7	9.32
Q9VT65	Calpain-B OS=Drosophila melanogaster GN=CalpB PE=1 SV=2 - [CANB_DROME]	0.00	3.57	1	1	1	1		925	103.7	5.02
P39205	Molybdenum cofactor synthesis protein cinnamon OS=Drosophila melanogaster GN=cin PE=1 SV=3 - [CIN_DROME]	0.00	2.16	1	1	1	1	9.752E6	601	65.7	6.24
Q01819	Connectin OS=Drosophila melanogaster GN=Con PE=2 SV=2 - [CONN_DROME]	0.00	3.96	2	1	1	1		682	75.9	6.77
P00408	Cytochrome c oxidase subunit 2 OS=Drosophila melanogaster GN=mt:CoII PE=3 SV=1 - [COX2_DROME]	0.00	4.39	1	1	1	1	3.113E8	228	26.2	5.10
Q24478	Centrosome-associated zinc finger protein CP190 OS=Drosophila melanogaster GN=Cp190 PE=1 SV=2 - [CP190_DROME]	0.00	2.28	1	1	1	1		1096	121.6	4.67
Q9VYQ5	Probable cytochrome P450 318a1 OS=Drosophila melanogaster GN=Cyp318a1 PE=2 SV=4 - [CP318_DROME]	0.00	4.29	1	1	1	1		536	61.7	8.76
P82713	Probable cytochrome P450 309a2 OS=Drosophila melanogaster GN=Cyp309a2 PE=2 SV=2 - [CP392_DROME]	0.00	5.02	1	1	1	1		538	62.3	9.22
Q8MZC4	Probable cardiolipin synthase OS=Drosophila melanogaster GN=CLS PE=2 SV=1 - [CRLS1_DROME]	0.00	1.86	1	1	1	1		322	36.4	9.77
Q8SYG2	COP9 signalosome complex subunit 3 OS=Drosophila melanogaster GN=CSN3 PE=2 SV=2 - [CSN3_DROME]	0.00	2.02	1	1	1	1	2.851E7	445	50.7	6.25
Q9VIV3	Cytoplasmic tRNA 2-thiolation protein 2 OS=Drosophila melanogaster GN=CG10189 PE=1 SV=1 - [CTU2_DROME]	0.00	2.47	1	1	1	1	7.773E7	405	44.7	6.33
Q9V629	Protein cutoff OS=Drosophila melanogaster GN=cuff PE=1 SV=1 - [CUFF_DROME]	0.00	6.25	1	1	1	2		384	44.8	7.43
Q9V3B6	Protein CWC15 homolog OS=Drosophila melanogaster GN=c12.1 PE=2 SV=1 - [CWC15_DROME]	0.00	2.32	1	1	1	1	8.925E5	259	28.4	7.43
Q9VF87	Cytoplasmic FMR1-interacting protein OS=Drosophila melanogaster GN=Sra-1 PE=1 SV=1 - [CYFIP_DROME]	0.00	0.54	1	1	1	1	9.756E7	1291	149.2	7.12
Q9VH89	Protein dalmatian OS=Drosophila melanogaster GN=dmt PE=1 SV=1 - [DALMA_DROME]	0.00	1.17	1	1	1	1	3.747E7	857	96.5	9.39
P91931	S-adenosylmethionine decarboxylase proenzyme OS=Drosophila melanogaster GN=SamDC PE=2 SV=1 - [DCAM_DROME]	0.00	8.36	1	1	1	1	1.545E7	347	39.8	4.91
P13496	Dynactin subunit 1 OS=Drosophila melanogaster GN=Gl PE=1 SV=2 - [DCTN1_DROME]	0.00	0.87	1	1	1	1	4.664E6	1265	141.1	5.62
P18173	Glucose dehydrogenase [FAD, quinone] OS=Drosophila melanogaster GN=Gld PE=3 SV=3 - [DHGL_DROME]	0.00	3.68	1	1	1	1		625	68.4	7.23
Q8T626	Protein disks lost OS=Drosophila melanogaster GN=dlt PE=1 SV=2 - [DLT_DROME]	0.00	3.31	1	2	2	2		1240	139.4	6.93
Q9W1H4	DNA ligase 1 OS=Drosophila melanogaster GN=DNA-ligI PE=1 SV=2 - [DNLI1_DROME]	0.00	2.54	1	1	1	2	1.888E7	747	84.7	6.62
Q9V9U4	Deoxyhypusine hydroxylase OS=Drosophila melanogaster GN=nero PE=2 SV=1 - [DOHH_DROME]	0.00	6.62	1	1	1	2	8.007E6	302	33.5	4.73
P26019	DNA polymerase alpha catalytic subunit OS=Drosophila melanogaster GN=DNApol-alpha180 PE=1 SV=2 - [DPOLA_DROME]	0.00	1.88	1	2	2	2		1488	169.8	8.02
Q24292	Protein dachsous OS=Drosophila melanogaster GN=ds PE=1 SV=3 - [DS_DROME]	0.00	0.31	2	1	1	1	7.227E7	3503	379.5	5.03
Q9V9Z9	Probable rRNA-processing protein EBP2 homolog OS=Drosophila melanogaster GN=CG1542 PE=2 SV=1 - [EBP2_DROME]	0.00	6.51	1	1	1	1	5.743E6	307	35.1	10.10
Q9VE34	Ectopic P granules protein 5 homolog OS=Drosophila melanogaster GN=CG14299 PE=1 SV=2 - [EPG5_DROME]	0.00	1.55	1	1	1	1		2455	280.5	6.57
Q07436	Protein expanded OS=Drosophila melanogaster GN=ex PE=1 SV=3 - [EXPA_DROME]	0.00	1.33	1	1	1	1		1427	153.5	8.34
Q8T3L6	Probable malonyl-CoA-acyl carrier protein transacylase, mitochondrial OS=Drosophila melanogaster GN=beg PE=2 SV=2 - [FABD_DROME]	0.00	5.80	1	1	1	1		379	42.1	9.16
Q960W6	Putative fatty acyl-CoA reductase CG8306 OS=Drosophila melanogaster GN=CG8306 PE=2 SV=1 - [FACR3_DROME]	0.00	2.71	1	1	1	1	5.028E6	516	58.4	9.32

Accession	Description	Score	Coverage	# Proteins	# Unique Peptides	# Peptides	# PSMs	Area	# AAs	MW [kDa]	calc. pI
P33450	Cadherin-related tumor suppressor OS=Drosophila melanogaster GN=ft PE=1 SV=3 - [FAT_DROME]	0.00	0.49	1	1	1	1		5147	564.4	5.08
Q8SWV6	Adenosine monophosphate-protein transferase FICD homolog OS=Drosophila melanogaster GN=Fic PE=1 SV=1 - [FICD_DROME]	0.00	5.28	1	1	1	1	8.112E6	492	55.7	6.70
O61491	Flotillin-1 OS=Drosophila melanogaster GN=Flo-1 PE=2 SV=1 - [FLOT1_DROME]	0.00	7.28	1	1	1	2	1.895E7	426	47.1	5.64
P40795	Protein folded gastrulation OS=Drosophila melanogaster GN=fog PE=2 SV=1 - [FOG_DROME]	0.00	3.42	1	1	1	1		730	78.2	4.49
Q9W596	Microtubule-associated protein futsch OS=Drosophila melanogaster GN=futsch PE=1 SV=4 - [FUTSC_DROME]	0.00	0.22	1	1	1	1	6.313E7	5495	591.7	4.97
Q9W0P5	UDP-glucose 4-epimerase OS=Drosophila melanogaster GN=Gale PE=1 SV=1 - [GALE_DROME]	0.00	10.29	1	2	2	3	1.790E8	350	38.7	7.46
Q6WV20	Polypeptide N-acetylgalactosaminyltransferase 1 OS=Drosophila melanogaster GN=GalNAc-T1 PE=2 SV=2 - [GALT1_DROME]	0.00	4.99	1	1	1	1		601	68.9	6.92
Q6WV17	Polypeptide N-acetylgalactosaminyltransferase 5 OS=Drosophila melanogaster GN=pgant5 PE=2 SV=2 - [GALT5_DROME]	0.00	4.13	1	1	1	1		630	72.1	7.83
Q8SY33	Protein Gawky OS=Drosophila melanogaster GN=gw PE=1 SV=1 - [GAWKY_DROME]	0.00	3.54	1	2	2	2	6.955E6	1384	142.9	6.74
Q8INF0	Soluble guanylate cyclase 88E OS=Drosophila melanogaster GN=Gyc88E PE=1 SV=3 - [GCY8E_DROME]	0.00	2.01	2	1	1	1	1.211E7	947	105.0	6.79
Q9VNL4	Glutaredoxin domain-containing cysteine-rich protein CG31559 OS=Drosophila melanogaster GN=CG31559 PE=1 SV=2 - [GRCR1_DROME]	0.00	4.19	1	1	1	1		454	50.1	6.40
Q9W3K5	Glutamate--cysteine ligase OS=Drosophila melanogaster GN=Gclc PE=2 SV=1 - [GSH1_DROME]	0.00	3.21	1	1	1	1	8.549E6	717	80.7	6.15
Q9V3D2	Oxygen-dependent coproporphyrinogen-III oxidase OS=Drosophila melanogaster GN=Coprox PE=1 SV=1 - [HEM6_DROME]	0.00	5.38	1	1	1	1		390	44.4	8.72
Q9V9S8	Ferrochelatase, mitochondrial OS=Drosophila melanogaster GN=FeCh PE=2 SV=1 - [HEMH_DROME]	0.00	4.17	1	1	1	1		384	43.6	8.40
P55162	Membrane-associated protein Hem OS=Drosophila melanogaster GN=Hem PE=2 SV=1 - [HEM_DROME]	0.00	2.22	1	2	2	2	1.847E7	1126	129.3	6.81
Q9NFT9	Hexokinase type 1 OS=Drosophila melanogaster GN=Hex-t1 PE=2 SV=1 - [HXK1_DROME]	0.00	4.52	1	1	1	1	3.469E5	465	52.2	6.39
Q08180	Irregular chiasm C-roughest protein OS=Drosophila melanogaster GN=rst PE=1 SV=2 - [ICCR_DROME]	0.00	2.09	1	1	1	1		764	83.0	6.99
Q8MLZ7	Chitinase-like protein Idgf3 OS=Drosophila melanogaster GN=Idgf3 PE=1 SV=3 - [IDGF3_DROME]	0.00	4.08	2	1	1	1		441	49.2	7.55
P09208	Insulin-like receptor OS=Drosophila melanogaster GN=InR PE=1 SV=3 - [INSR_DROME]	0.00	0.65	1	1	1	1		2144	239.6	6.90
Q9VWL5	Innexin inx5 OS=Drosophila melanogaster GN=Inx5 PE=2 SV=2 - [INX5_DROME]	0.00	4.30	1	1	1	1		419	48.9	8.65
Q24368	Chromatin-remodeling complex ATPase chain Iswi OS=Drosophila melanogaster GN=Iswi PE=1 SV=1 - [ISWI_DROME]	0.00	0.97	1	1	1	1		1027	118.8	8.29
P29993	Inositol 1,4,5-trisphosphate receptor OS=Drosophila melanogaster GN=Itp-r83A PE=2 SV=3 - [ITPR_DROME]	0.00	0.35	1	1	1	1		2838	318.9	5.83
P20348	Sex-regulated protein janus-A OS=Drosophila melanogaster GN=janA PE=2 SV=2 - [JANA_DROME]	0.00	7.41	1	1	1	1		135	15.2	7.49
P11346	Raf homolog serine/threonine-protein kinase phl OS=Drosophila melanogaster GN=phl PE=1 SV=6 - [KRAF1_DROME]	0.00	2.84	1	1	1	1		739	83.7	8.73
Q9V4Z9	Protein lines OS=Drosophila melanogaster GN=lin PE=1 SV=1 - [LINES_DROME]	0.00	0.82	1	1	1	2	1.025E9	858	96.2	5.31
P42284	Longitudinals lacking protein, isoforms H/M/V OS=Drosophila melanogaster GN=lola PE=1 SV=2 - [LOLA2_DROME]	0.00	2.91	6	1	1	1	2.838E6	549	59.3	5.52
P11995	Larval serum protein 1 alpha chain OS=Drosophila melanogaster GN=Lsp1alpha PE=2 SV=3 - [LSP1A_DROME]	0.00	1.23	1	1	1	1	7.418E8	816	98.8	6.14
Q9VZL6	Ragulator complex protein LAMTOR4 homolog OS=Drosophila melanogaster GN=CG14977 PE=1 SV=2 - [LTOR4_DROME]	0.00	22.50	2	1	1	1	4.113E7	120	13.3	5.03
Q9VNS0	Protein maelstrom OS=Drosophila melanogaster GN=mael PE=1 SV=1 - [MAEL_DROME]	0.00	2.18	1	1	1	1		459	51.6	8.18
Q7K3L1	Guanylate kinase-associated protein mars OS=Drosophila melanogaster GN=mars PE=1 SV=1 - [MARS_DROME]	0.00	0.87	1	1	1	1		921	101.9	9.98
Q9VVX5	Lysophospholipid acyltransferase 5 OS=Drosophila melanogaster GN=nes PE=1 SV=1 - [MBOA5_DROME]	0.00	1.61	1	1	1	1		497	57.4	8.19
Q9VGW6	DNA replication licensing factor Mcm5 OS=Drosophila melanogaster GN=Mcm5 PE=1 SV=1 - [MCM5_DROME]	0.00	1.77	1	1	1	1		733	82.2	7.84
Q9V461	DNA replication licensing factor Mcm6 OS=Drosophila melanogaster GN=Mcm6 PE=1 SV=1 - [MCM6_DROME]	0.00	2.08	1	2	2	3	4.874E7	817	92.3	5.38
P49028	Protein mago nashi OS=Drosophila melanogaster GN=mago PE=1 SV=1 - [MGN_DROME]	0.00	5.44	1	1	1	1	4.321E6	147	17.3	6.11
Q86BA1	Protein-methionine sulfoxide oxidase Mical OS=Drosophila melanogaster GN=Mical PE=1 SV=1 - [MICAL_DROME]	0.00	0.28	1	1	1	1	7.301E6	4723	524.7	7.09
Q9VUM0	Probable DNA mismatch repair protein Msh6 OS=Drosophila melanogaster GN=Msh6 PE=1 SV=2 - [MSH6_DROME]	0.00	2.27	1	1	1	1	5.609E8	1190	133.1	7.28
Q24134	Negative elongation factor D OS=Drosophila melanogaster GN=TH1 PE=1 SV=2 - [NELFD_DROME]	0.00	2.60	1	1	1	2	1.958E8	578	65.7	5.07
Q9VXE6	Nuclear pore complex protein Nup153 OS=Drosophila melanogaster GN=Nup153 PE=1 SV=4 - [NU153_DROME]	0.00	3.93	2	3	3	3	7.955E6	1883	196.5	8.13
Q9VDV3	Probable nucleoporin Nup58 OS=Drosophila melanogaster GN=Nup58 PE=2 SV=1 - [NUPL1_DROME]	0.00	2.20	1	1	1	1	1.830E7	546	55.5	9.44
Q9V931	General odorant-binding protein 57c OS=Drosophila melanogaster GN=Obp57c PE=2 SV=1 - [OB57C_DROME]	0.00	13.42	1	1	1	1		149	17.2	4.31
Q9W589	GDP-fucose protein O-fucosyltransferase 2 OS=Drosophila melanogaster GN=O-fut2 PE=1 SV=1 - [OFUT2_DROME]	0.00	4.49	1	1	1	1		490	56.0	8.12
P08255	Opsin Rh4 OS=Drosophila melanogaster GN=Rh4 PE=1 SV=2 - [OPS4_DROME]	0.00	8.99	1	1	1	1	2.646E7	378	42.6	7.75
P91679	Peptide transporter family 1 OS=Drosophila melanogaster GN=yin PE=1 SV=2 - [PEPT1_DROME]	0.00	5.11	2	1	1	1	9.193E6	743	82.2	6.30
Q9VWK6	Post-GPI attachment to proteins factor 2-like OS=Drosophila melanogaster GN=CG7990 PE=2 SV=4 - [PGP2L_DROME]	0.00	5.54	1	1	1	1	1.270E6	325	36.1	8.07
P39769	Polyhomeotic-proximal chromatin protein OS=Drosophila melanogaster GN=ph-p PE=1 SV=2 - [PHP_DROME]	0.00	2.20	1	1	1	1		1589	167.2	9.06
Q9U9P7	Cytoplasmic phosphatidylinositol transfer protein 1 OS=Drosophila melanogaster GN=rdgBbeta PE=2 SV=1 - [PITC1_DROME]	0.00	4.76	2	2	2	2	1.704E7	273	31.7	5.45
Q9W3C7	Palmitoyl-protein thioesterase 1 OS=Drosophila melanogaster GN=Ppt1 PE=1 SV=2 - [PPT1_DROME]	0.00	9.24	1	1	1	1		314	35.8	7.14
Q9V9E3	Presequence protease, mitochondrial OS=Drosophila melanogaster GN=CG3107 PE=2 SV=2 - [PREP_DROME]	0.00	2.13	1	2	2	2	8.056E7	1034	119.2	6.81
Q9VFS8	26S proteasome non-ATPase regulatory subunit 9 OS=Drosophila melanogaster GN=CG9588 PE=1 SV=1 - [PSMD9_DROME]	0.00	4.55	1	1	1	1		220	23.9	5.66
A1Z9A8	Protein PTCD3 homolog, mitochondrial OS=Drosophila melanogaster GN=CG4679 PE=2 SV=1 - [PTCD3_DROME]	0.00	1.23	1	1	1	1	1.328E7	652	74.2	5.92

Accession	Description	Score	Coverage	# Proteins	# Unique Peptides	# Peptides	# PSMs	Area	# AAs	MW [kDa]	calc. pI
Q9VPK7	Putative tRNA pseudouridine synthase Pus10 OS=Drosophila melanogaster GN=Pus10 PE=2 SV=2 - [PUS10_DROME]	0.00	2.80	1	1	1	3	5.300E6	500	56.7	8.35
Q24472	Retinoblastoma family protein OS=Drosophila melanogaster GN=Rbf PE=1 SV=2 - [RBF_DROME]	0.00	2.60	1	2	2	2	1.558E7	845	96.8	6.57
Q9VYS3	Regulator of nonsense transcripts 1 homolog OS=Drosophila melanogaster GN=Upf1 PE=1 SV=2 - [RENT1_DROME]	0.00	2.12	1	1	1	1		1180	129.8	7.33
P35600	Replication factor C subunit 1 OS=Drosophila melanogaster GN=Gnf1 PE=1 SV=2 - [RFC1_DROME]	0.00	2.54	1	2	2	2	3.588E6	986	108.5	9.31
Q86BY9	Protein rigor mortis OS=Drosophila melanogaster GN=rig PE=1 SV=1 - [RIG_DROME]	0.00	1.05	1	1	1	1	2.534E7	1235	137.7	6.77
O16130	60S ribosomal protein L39 OS=Drosophila melanogaster GN=RpL39 PE=1 SV=2 - [RL39_DROME]	0.00	13.73	1	1	1	1	1.017E9	51	6.3	12.56
Q9VMX0	39S ribosomal protein L28, mitochondrial OS=Drosophila melanogaster GN=mRpL28 PE=2 SV=1 - [RM28_DROME]	0.00	1.99	1	1	1	1	5.527E7	302	34.9	8.95
Q9VC49	DNA-directed RNA polymerases I, II, and III subunit RPABC5 OS=Drosophila melanogaster GN=Rpb10 PE=1 SV=1 - [RPAB5_DROME]	0.00	8.96	1	1	1	1		67	7.7	7.77
P04052	DNA-directed RNA polymerase II subunit RPB1 OS=Drosophila melanogaster GN=RpII215 PE=3 SV=4 - [RPB1_DROME]	0.00	0.69	1	1	1	1	1.453E7	1887	209.0	7.81
Q9V4Q8	Probable U2 small nuclear ribonucleoprotein A' OS=Drosophila melanogaster GN=U2A PE=1 SV=1 - [RU2A_DROME]	0.00	6.42	1	1	1	1		265	29.7	8.51
Q9VXE0	Probable small nuclear ribonucleoprotein G OS=Drosophila melanogaster GN=SmG PE=1 SV=1 - [RUXG_DROME]	0.00	11.84	1	1	1	1	2.017E8	76	8.5	8.79
Q9VWF2	Supporter of activation of yellow protein OS=Drosophila melanogaster GN=e(y)3 PE=2 SV=3 - [SAYP_DROME]	0.00	2.39	3	1	1	1	2.335E7	2006	212.6	7.52
Q9W0Y8	Sodium channel protein 60E OS=Drosophila melanogaster GN=NaCP60E PE=2 SV=5 - [SCN60_DROME]	0.00	0.32	1	1	1	1		2821	318.4	6.93
Q94523	Succinate dehydrogenase [ubiquinone] flavoprotein subunit, mitochondrial OS=Drosophila melanogaster GN=SdhA PE=2 SV=3 - [SDHA_DROME]	0.00	5.45	1	2	2	2	3.013E7	661	72.3	7.12
P54359	Septin-2 OS=Drosophila melanogaster GN=Sep2 PE=2 SV=2 - [SEPT2_DROME]	0.00	2.15	2	1	1	1	1.681E7	419	48.4	7.61
Q9VHI4	Probable splicing factor 3B subunit 5 OS=Drosophila melanogaster GN=CG11985 PE=1 SV=1 - [SF3B5_DROME]	0.00	23.53	1	1	1	1	1.763E7	85	10.0	6.35
Q9VAQ7	Pre-mRNA-splicing factor Slu7 OS=Drosophila melanogaster GN=Slu7 PE=1 SV=2 - [SLU7_DROME]	0.00	2.44	1	1	1	1	4.006E7	574	65.9	7.06
Q9VS48	Structure-specific endonuclease subunit SLX4 OS=Drosophila melanogaster GN=mus312 PE=1 SV=2 - [SLX4_DROME]	0.00	2.18	2	1	1	1	3.812E6	1145	128.1	5.45
Q70PP2	Serine/threonine-protein kinase Smg1 OS=Drosophila melanogaster GN=nonC PE=1 SV=2 - [SMG1_DROME]	0.00	0.68	1	1	1	1		3218	361.4	6.86
Q23972	Protein Smaug OS=Drosophila melanogaster GN=smg PE=1 SV=2 - [SMG_DROME]	0.00	1.20	1	1	1	1		999	109.0	7.43
Q23983	Soluble NSF attachment protein OS=Drosophila melanogaster GN=Snap PE=1 SV=1 - [SNAP_DROME]	0.00	4.45	1	1	1	1	3.058E7	292	33.0	5.45
Q9V9J3	Tyrosine-protein kinase Src42A OS=Drosophila melanogaster GN=Src42A PE=2 SV=1 - [SRC42_DROME]	0.00	2.13	1	1	1	1		517	59.0	6.68
Q05344	FACT complex subunit Ssrp1 OS=Drosophila melanogaster GN=Ssrp PE=1 SV=2 - [SSRP1_DROME]	0.00	1.66	1	1	1	1		723	81.5	5.63
Q9V877	Kinesin-like protein subito OS=Drosophila melanogaster GN=sub PE=1 SV=1 - [SUB_DROME]	0.00	2.39	1	1	1	1	5.831E6	628	71.3	5.48
P25172	Protein suppressor 2 of zeste OS=Drosophila melanogaster GN=Su(z)2 PE=2 SV=2 - [SUZ2_DROME]	0.00	0.88	1	1	1	1		1368	146.3	9.33
Q9VFL5	Methionine--tRNA ligase, mitochondrial OS=Drosophila melanogaster GN=Aats-met PE=2 SV=2 - [SYMM_DROME]	0.00	3.26	1	1	1	1		582	66.0	8.00
Q24325	Transcription initiation factor TFIID subunit 2 OS=Drosophila melanogaster GN=Taf2 PE=1 SV=2 - [TAF2_DROME]	0.00	1.15	1	1	1	1	2.875E7	1221	139.4	7.23
Q9VWB0	Tectonin beta-propeller repeat-containing protein OS=Drosophila melanogaster GN=Pex23 PE=2 SV=2 - [TECPR_DROME]	0.00	1.26	1	1	1	1		1350	149.4	6.99
Q9VMH0	Tectonic OS=Drosophila melanogaster GN=tectonic PE=1 SV=4 - [TECT_DROME]	0.00	6.39	1	1	1	1		657	73.9	6.27
Q9V3N8	Probable tRNA(His) guanylyltransferase OS=Drosophila melanogaster GN=l(2)35Bc PE=2 SV=1 - [THG1_DROME]	0.00	6.99	1	1	1	1	6.319E7	286	33.1	7.47
P49021	Protein timeless OS=Drosophila melanogaster GN=tim PE=1 SV=3 - [TIM_DROME]	0.00	2.72	1	2	2	2		1398	156.3	5.25
P30974	Tachykinin-like peptides receptor 86C OS=Drosophila melanogaster GN=TkR86C PE=2 SV=2 - [TLR1_DROME]	0.00	6.55	2	1	1	1		504	58.0	9.16
Q9VQE9	Transmembrane and TPR repeat-containing protein CG31690 OS=Drosophila melanogaster GN=CG31690 PE=2 SV=3 - [TMTC1_DROME]	0.00	2.56	1	1	1	1	2.622E7	859	94.8	9.03
Q8IN41	Protein Turandot X OS=Drosophila melanogaster GN=TotX PE=2 SV=1 - [TOTX_DROME]	0.00	19.01	1	1	1	1		142	16.4	7.55
Q9VP47	Pre-rRNA-processing protein TSR1 homolog OS=Drosophila melanogaster GN=CG7338 PE=1 SV=1 - [TSR1_DROME]	0.00	2.21	1	1	1	1		814	93.7	6.87
P34739	Transcription termination factor 2 OS=Drosophila melanogaster GN=lds PE=1 SV=2 - [TTF2_DROME]	0.00	0.94	1	1	1	1		1061	118.3	6.65
Q9VUV9	Putative U5 small nuclear ribonucleoprotein 200 kDa helicase OS=Drosophila melanogaster GN=l(3)72Ab PE=2 SV=4 - [U520_DROME]	0.00	1.77	1	4	4	4	3.270E7	2142	244.4	6.04
Q9VNI3	UPF0609 protein CG1218 OS=Drosophila melanogaster GN=CG1218 PE=1 SV=2 - [U609_DROME]	0.00	4.45	1	1	1	1	1.929E6	449	51.1	7.03
Q9VSF3	Nedd8-conjugating enzyme Ubc12 OS=Drosophila melanogaster GN=Ubc12 PE=1 SV=1 - [UBC12_DROME]	0.00	13.81	1	1	1	1	1.104E7	181	20.7	7.15
Q9VRP5	Ubiquitin carboxyl-terminal hydrolase 36 OS=Drosophila melanogaster GN=scny PE=1 SV=3 - [UBP36_DROME]	0.00	3.08	1	2	2	2		1038	114.0	9.09
Q9VUR0	Probable Ufm1-specific protease 2 OS=Drosophila melanogaster GN=UFSP2 PE=2 SV=1 - [UFSP2_DROME]	0.00	1.81	1	1	1	1	7.009E6	607	68.2	6.76
Q9VB11	Protein unc-80 homolog OS=Drosophila melanogaster GN=CG18437 PE=3 SV=4 - [UNC80_DROME]	0.00	0.46	1	1	1	1		3295	366.6	8.12
Q9W552	Vacuolar protein sorting-associated protein 26 OS=Drosophila melanogaster GN=Vps26 PE=2 SV=1 - [VPS26_DROME]	0.00	3.56	1	1	1	1	3.405E7	478	52.6	6.39
Q8MSY4	Vacuolar protein sorting-associated protein 51 homolog OS=Drosophila melanogaster GN=CG15087 PE=2 SV=1 - [VPS51_DROME]	0.00	2.70	1	1	1	1	6.931E6	740	83.9	5.40
Q06521	Vitelline membrane protein Vm34Ca OS=Drosophila melanogaster GN=Vm34Ca PE=1 SV=2 - [VTU3_DROME]	0.00	35.29	1	1	1	2	3.575E7	119	11.9	8.05
O44342	Protein windbeutel OS=Drosophila melanogaster GN=wbl PE=1 SV=1 - [WBL_DROME]	0.00	2.72	1	1	1	1		257	29.4	6.44
Q7K0L4	WD repeat-containing protein 26 homolog OS=Drosophila melanogaster GN=CG7611 PE=1 SV=1 - [WDR26_DROME]	0.00	5.08	1	2	2	4	3.761E6	630	69.6	5.63
Q8T088	WD repeat-containing protein 55 homolog OS=Drosophila melanogaster GN=CG14722 PE=2 SV=1 - [WDR55_DROME]	0.00	2.41	1	1	1	1	1.415E7	498	55.3	4.53
Q9VJN5	Zinc finger protein weckle OS=Drosophila melanogaster GN=wek PE=1 SV=1 - [WEK_DROME]	0.00	5.96	1	1	1	1	1.136E6	470	53.2	7.05
Q3LHL9	Protein winged eye OS=Drosophila melanogaster GN=wge PE=1 SV=1 - [WGE_DROME]	0.00	0.72	1	1	1	1		1658	178.2	6.99

Accession	Description	Score	Coverage	# Proteins	# Unique Peptides	# Peptides	# PSMs	Area	# AAs	MW [kDa]	calc. pI
P82804	Partner of Y14 and mago OS=Drosophila melanogaster GN=wibg PE=1 SV=1 - [WIBG_DROME]	0.00	8.70	1	1	1	1		207	23.4	9.83
Q8SX68	Uncharacterized protein CG10915 OS=Drosophila melanogaster GN=CG10915 PE=1 SV=1 - [Y0915_DROME]	0.00	1.48	1	1	1	1	1.174E7	609	65.4	7.06
Q6NLL1	WD repeat-containing protein CG11141 OS=Drosophila melanogaster GN=CG11141 PE=1 SV=1 - [Y1411_DROME]	0.00	1.69	1	1	1	1	7.206E7	710	79.0	5.91
Q7YZA2	Uncharacterized protein CG7065 OS=Drosophila melanogaster GN=CG7065 PE=1 SV=1 - [Y7065_DROME]	0.00	1.87	1	1	1	1		1231	136.7	9.23
Q9VA00	Protein zwilch OS=Drosophila melanogaster GN=zwilch PE=1 SV=1 - [ZWILC_DROME]	0.00	4.21	1	1	1	1		641	72.4	6.65
Q9VLW9	Homocysteine-induced endoplasmic reticulum protein, isoform A OS=Drosophila melanogaster GN=Herp PE=2 SV=1 - [Q9VLW9_DROME]	0.00	1.29	2	1	1	1	5.479E7	465	50.0	6.60
Q9VE12	CG6005 OS=Drosophila melanogaster GN=CG6005 PE=2 SV=2 - [Q9VE12_DROME]	0.00	6.48	1	1	1	1		324	36.7	8.51
Q9XYZ2	Excision repair protein ERCC2/XPD OS=Drosophila melanogaster GN=Xpd PE=2 SV=1 - [Q9XYZ2_DROME]	0.00	2.15	2	1	1	1		745	85.0	6.93
Q9VB90	CG6283 OS=Drosophila melanogaster GN=CG6283 PE=2 SV=1 - [Q9VB90_DROME]	0.00	7.67	1	1	1	1	5.223E5	339	36.8	6.15
Q8IPX7	CG31938-PA OS=Drosophila melanogaster GN=Rrp40 PE=2 SV=1 - [Q8IPX7_DROME]	0.00	3.88	1	1	1	1	1.556E7	232	25.0	8.31
Q9VG11	CG14383 OS=Drosophila melanogaster GN=CG14383 PE=2 SV=1 - [Q9VG11_DROME]	0.00	3.01	1	1	1	1	1.036E9	332	38.7	8.51
Q9W3W1	Coronin OS=Drosophila melanogaster GN=pod1 PE=2 SV=1 - [Q9W3W1_DROME]	0.00	2.89	3	1	1	1		1074	119.3	6.77
A1ZBL2	CG7744 OS=Drosophila melanogaster GN=CG7744-RA PE=2 SV=1 - [A1ZBL2_DROME]	0.00	4.12	1	1	1	1		680	76.6	8.95
Q9VT31	CG16719-PA OS=Drosophila melanogaster GN=CG16719 PE=2 SV=1 - [Q9VT31_DROME]	0.00	8.86	1	1	1	1	2.381E6	271	30.6	9.41
Q9VBV8	CG11851 OS=Drosophila melanogaster GN=CG11851 PE=2 SV=1 - [Q9VBV8_DROME]	0.00	1.61	1	1	1	1	5.511E7	623	72.0	9.25
Q9VG04	CG8031 OS=Drosophila melanogaster GN=CG8031 PE=2 SV=1 - [Q9VG04_DROME]	0.00	3.73	1	1	1	1	6.144E7	295	33.2	6.93
Q8SYF5	CG32373, isoform A OS=Drosophila melanogaster GN=CG32373 PE=2 SV=1 - [Q8SYF5_DROME]	0.00	3.29	1	1	1	1	4.211E6	486	54.0	6.05
Q0E8N9	CG9339-PD, isoform D OS=Drosophila melanogaster GN=sky PE=4 SV=1 - [Q0E8N9_DROME]	0.00	1.06	7	1	1	1	4.968E7	568	65.0	7.64
Q9VIP3	CG10747, isoform B OS=Drosophila melanogaster GN=CG10747 PE=2 SV=2 - [Q9VIP3_DROME]	0.00	4.88	1	1	1	1	1.101E8	328	38.3	7.03
Q9VPM7	CG17078, isoform B OS=Drosophila melanogaster GN=CG17078-RA PE=2 SV=2 - [Q9VPM7_DROME]	0.00	3.77	1	1	1	2	7.660E6	610	67.5	7.49
Q9VU75	CG14109, isoform A OS=Drosophila melanogaster GN=CG14109 PE=2 SV=1 - [Q9VU75_DROME]	0.00	4.12	1	1	1	1		243	27.2	4.78
Q9VJZ1	CG9302 OS=Drosophila melanogaster GN=CG9302-RA PE=2 SV=1 - [Q9VJZ1_DROME]	0.00	2.35	1	1	1	1	6.712E6	510	57.9	8.78
Q9VUU7	CG18081, isoform B OS=Drosophila melanogaster GN=CG18081 PE=2 SV=1 - [Q9VUU7_DROME]	0.00	9.09	2	1	1	1		77	8.8	9.64
Q8IQ27	Ubiquitin carboxyl-terminal hydrolase OS=Drosophila melanogaster GN=CG14619-RC PE=2 SV=1 - [Q8IQ27_DROME]	0.00	4.58	2	1	1	1		938	101.6	9.45
Q9VVH9	GH24467p OS=Drosophila melanogaster GN=Oatp74D PE=2 SV=2 - [Q9VVH9_DROME]	0.00	4.15	1	1	1	1		819	88.4	7.27
Q9VG81	CG5167 OS=Drosophila melanogaster GN=CG5167 PE=2 SV=1 - [Q9VG81_DROME]	0.00	4.64	1	1	1	1		431	47.2	9.44
Q9VCR2	Aminoacylase-1 OS=Drosophila melanogaster GN=CG6726 PE=3 SV=2 - [Q9VCR2_DROME]	0.00	2.24	1	1	1	1		401	45.0	5.62
O77424	CG32812, isoform B OS=Drosophila melanogaster GN=EG:114D9.1 PE=2 SV=1 - [O77424_DROME]	0.00	13.33	1	1	1	1		225	25.8	6.79
Q9W2U5	AT19485p OS=Drosophila melanogaster GN=CG2887 PE=2 SV=1 - [Q9W2U5_DROME]	0.00	7.89	1	1	1	1		342	37.4	9.06
Q9VDD5	CG10824 OS=Drosophila melanogaster GN=CG10824 PE=1 SV=2 - [Q9VDD5_DROME]	0.00	1.99	1	1	1	1	7.003E7	554	62.1	4.98
Q9W5Y4	CG17018, isoform A OS=Drosophila melanogaster GN=CG17018-RC PE=2 SV=2 - [Q9W5Y4_DROME]	0.00	1.26	3	1	1	1	8.435E6	1272	143.5	8.28
Q9VQ94	LP05220p OS=Drosophila melanogaster GN=Sec24CD PE=2 SV=2 - [Q9VQ94_DROME]	0.00	0.92	2	1	1	1	1.761E6	1193	129.2	7.25
Q9W074	FI04779p OS=Drosophila melanogaster GN=HBS1 PE=2 SV=2 - [Q9W074_DROME]	0.00	1.49	1	1	1	1		670	74.1	6.70
Q9VA95	Bet5 OS=Drosophila melanogaster GN=Bet5 PE=2 SV=1 - [Q9VA95_DROME]	0.00	15.86	1	1	1	1	1.046E8	145	17.0	9.07
Q9VA51	CG2267 OS=Drosophila melanogaster GN=CG2267 PE=2 SV=2 - [Q9VA51_DROME]	0.00	7.95	1	1	1	2	6.366E5	390	44.1	9.50
Q7KNM2	CG3018-PA, isoform A OS=Drosophila melanogaster GN=lwr PE=1 SV=1 - [Q7KNM2_DROME]	0.00	14.47	1	2	2	3	2.057E7	159	18.1	8.41
Q8SX92	CG8001, isoform B OS=Drosophila melanogaster GN=CG8001 PE=2 SV=1 - [Q8SX92_DROME]	0.00	0.94	2	1	1	1	8.059E7	743	82.7	5.03
Q7KUZ1	FI18406p1 OS=Drosophila melanogaster GN=Stim PE=2 SV=2 - [Q7KUZ1_DROME]	0.00	1.78	2	1	1	1	1.595E7	561	63.7	6.49
Q9VGD2	Dpr17, isoform B OS=Drosophila melanogaster GN=dpr17 PE=4 SV=2 - [Q9VGD2_DROME]	0.00	7.09	2	1	1	1	7.240E6	508	54.0	6.13
Q7K2W2	CG11060 OS=Drosophila melanogaster GN=CG11060 PE=2 SV=1 - [Q7K2W2_DROME]	0.00	2.97	1	1	1	1	3.179E6	706	81.9	6.60
Q9W409	CG12219 OS=Drosophila melanogaster GN=CG12219 PE=2 SV=1 - [Q9W409_DROME]	0.00	4.45	1	1	1	1		562	60.6	7.56
Q7KTU3	CG7369 OS=Drosophila melanogaster GN=CG7369-RA PE=2 SV=1 - [Q7KTU3_DROME]	0.00	5.00	1	1	1	1		680	75.7	7.65
E1JIY8	CG42498 OS=Drosophila melanogaster GN=CG42498-RA PE=2 SV=1 - [E1JIY8_DROME]	0.00	6.31	1	1	1	1	1.452E7	111	12.5	5.92
Q9VFK2	Crossveinless c, isoform A OS=Drosophila melanogaster GN=cv-c PE=2 SV=2 - [Q9VFK2_DROME]	0.00	1.47	2	1	1	1		1017	115.1	9.41
Q9VHT5	LD31571p OS=Drosophila melanogaster GN=mRpL1 PE=2 SV=2 - [Q9VHT5_DROME]	0.00	3.95	1	1	1	2	6.954E6	354	40.3	9.42
Q0E965	SRPK, isoform A OS=Drosophila melanogaster GN=SRPK PE=3 SV=1 - [Q0E965_DROME]	0.00	3.93	1	1	1	1	7.854E6	764	85.1	8.98
Q9VTU7	GH14208p OS=Drosophila melanogaster GN=Rh7 PE=2 SV=2 - [Q9VTU7_DROME]	0.00	4.14	1	1	1	1	4.082E7	483	53.7	8.19
Q0IGY0	CG34411, isoform B OS=Drosophila melanogaster GN=CG4187 PE=2 SV=1 - [Q0IGY0_DROME]	0.00	1.99	2	1	1	1		806	90.4	6.55
Q9V428	Icarus, isoform A OS=Drosophila melanogaster GN=ics PE=2 SV=2 - [Q9V428_DROME]	0.00	2.12	2	1	1	1		283	31.8	7.55
Q9VQT8	CG16712, isoform B OS=Drosophila melanogaster GN=CG16712 PE=2 SV=1 - [Q9VQT8_DROME]	0.00	14.63	1	1	1	1	1.924E7	82	8.7	5.92

Accession	Description	Score	Coverage	# Proteins	# Unique Peptides	# Peptides	# PSMs	Area	# AAs	MW [kDa]	calc. pI
A4V1K1	AT21853p1 OS=Drosophila melanogaster GN=CG8398-RA PE=2 SV=1 - [A4V1K1_DROME]	0.00	8.51	2	1	1	1		435	49.3	7.33
Q9VRG3	CG1494, isoform B OS=Drosophila melanogaster GN=CG1494 PE=4 SV=3 - [Q9VRG3_DROME]	0.00	1.89	1	1	1	1	2.893E6	1695	194.1	7.17
Q9W047	CG5704, isoform A OS=Drosophila melanogaster GN=BEST:LD30049 PE=2 SV=1 - [Q9W047_DROME]	0.00	2.99	1	1	1	1	3.634E7	335	38.4	9.00
Q7JW61	Ubiquitin carboxyl-terminal hydrolase OS=Drosophila melanogaster GN=CG8494 PE=2 SV=1 - [Q7JW61_DROME]	0.00	2.05	1	1	1	2	2.610E7	975	109.6	5.62
Q961H1	Katanin p60 ATPase-containing subunit A1 OS=Drosophila melanogaster GN=kat-60L1 PE=2 SV=1 - [Q961H1_DROME]	0.00	4.79	2	1	1	1	1.914E6	605	66.6	9.55
E1JIB5	CG42494, isoform A OS=Drosophila melanogaster GN=CG42494 PE=4 SV=1 - [E1JIB5_DROME]	0.00	14.13	1	1	1	1		283	30.9	4.61
Q59E33	LD21442p OS=Drosophila melanogaster GN=scaf6 PE=2 SV=1 - [Q59E33_DROME]	0.00	1.35	1	1	1	1		960	107.5	9.22
Q9GNC8	PASILLA splice variant 1 OS=Drosophila melanogaster GN=ps PE=2 SV=1 - [Q9GNC8_DROME]	0.00	2.95	7	1	1	1	4.508E6	475	49.3	6.79
Q9VUL8	LD41491p OS=Drosophila melanogaster GN=Pex3 PE=2 SV=1 - [Q9VUL8_DROME]	0.00	3.38	1	1	1	1		385	43.7	8.50
Q9VBA3	AT30002p OS=Drosophila melanogaster GN=sda PE=2 SV=2 - [Q9VBA3_DROME]	0.00	0.84	2	1	1	1	1.881E7	1071	121.6	6.10
Q9I7V0	CG33988 OS=Drosophila melanogaster GN=Mid1 PE=2 SV=3 - [Q9I7V0_DROME]	0.00	1.45	1	1	1	1	3.269E8	1241	135.4	5.95
Q9VMQ8	CG7371 OS=Drosophila melanogaster GN=CG7371-RA PE=2 SV=1 - [Q9VMQ8_DROME]	0.00	4.23	1	1	1	1	6.234E5	662	77.4	6.62
O61444	MAP kinase kinase 4, isoform A OS=Drosophila melanogaster GN=Mkk4 PE=2 SV=1 - [O61444_DROME]	0.00	6.84	1	1	1	1	6.778E6	424	47.5	7.43
Q8SXU3	CG8207 OS=Drosophila melanogaster GN=CG8207 PE=2 SV=1 - [Q8SXU3_DROME]	0.00	2.51	1	1	1	1		438	48.1	7.72
Q9W1H6	CG5597 OS=Drosophila melanogaster GN=CG5597 PE=1 SV=1 - [Q9W1H6_DROME]	0.00	2.69	1	1	1	2	7.225E6	260	29.6	4.65
C1C560	MIP02960p OS=Drosophila melanogaster GN=RhoGAP1A PE=2 SV=1 - [C1C560_DROME]	0.00	1.44	4	1	1	1	1.012E8	903	100.0	8.41
Q9VF82	CG6196 OS=Drosophila melanogaster GN=Trs33 PE=2 SV=1 - [Q9VF82_DROME]	0.00	7.89	1	1	1	1	9.969E6	152	17.6	6.99
Q7KNR7	Photoreceptor dehydrogenase, isoform C OS=Drosophila melanogaster GN=Pdh PE=2 SV=1 - [Q7KNR7_DROME]	0.00	11.11	4	2	2	3	7.087E6	261	28.3	8.69
Q9VP79	FI21431p1 OS=Drosophila melanogaster GN=ko PE=2 SV=1 - [Q9VP79_DROME]	0.00	1.14	1	1	1	1	2.663E7	1049	113.5	8.57
Q2PDM3	CG33978, isoform C OS=Drosophila melanogaster GN=CG33978 PE=4 SV=3 - [Q2PDM3_DROME]	0.00	0.66	1	1	1	2	7.030E7	2289	256.4	6.60
Q9W3D2	Calmodulin-binding protein related to a Rab3 GDP/GTP exchange protein, isoform B OS=Drosophila melanogaster GN=Crag PE=2 SV=3 - [Q9W3D2_DROME]	0.00	1.64	2	1	1	2		1644	183.9	6.32
Q9W351	CG16892, isoform A OS=Drosophila melanogaster GN=CG16892-RA PE=2 SV=1 - [Q9W351_DROME]	0.00	5.79	1	2	2	2		466	52.2	6.71
Q9W363	Multi sex combs, isoform A OS=Drosophila melanogaster GN=mxc PE=4 SV=2 - [Q9W363_DROME]	0.00	0.87	1	1	1	1		1837	197.4	6.46
Q5U0X8	CG12325 OS=Drosophila melanogaster GN=CG12325 PE=2 SV=1 - [Q5U0X8_DROME]	0.00	1.69	1	1	1	1		949	105.9	5.34
A1ZAW0	Dicer-2 OS=Drosophila melanogaster GN=Dcr-2 PE=1 SV=1 - [A1ZAW0_DROME]	0.00	0.70	1	1	1	1		1722	197.6	6.86
Q9VRG8	CG1486, isoform A OS=Drosophila melanogaster GN=CG1486 PE=2 SV=1 - [Q9VRG8_DROME]	0.00	2.35	1	1	1	1		852	91.5	5.27
Q7JWW6	Ady43A OS=Drosophila melanogaster GN=Ady43A PE=2 SV=1 - [Q7JWW6_DROME]	0.00	2.19	1	1	1	2	8.124E7	366	40.3	5.83
Q8T0K8	Aldehyde dehydrogenase type III, isoform J OS=Drosophila melanogaster GN=Aldh-III PE=2 SV=1 - [Q8T0K8_DROME]	0.00	7.72	5	2	2	2	1.217E7	337	37.7	5.10
Q9VKB2	CG5336-PA OS=Drosophila melanogaster GN=Ced-12 PE=1 SV=1 - [Q9VKB2_DROME]	0.00	2.49	1	2	2	2	7.834E7	724	82.9	6.19
Q9VLE1	CG13108-PA OS=Drosophila melanogaster GN=CG13108 PE=2 SV=1 - [Q9VLE1_DROME]	0.00	3.86	1	1	1	1	1.333E7	233	26.9	4.94
Q9VBQ3	CG5111 OS=Drosophila melanogaster GN=CG5111-RA PE=2 SV=1 - [Q9VBQ3_DROME]	0.00	1.95	1	1	1	1	6.991E6	614	69.9	5.27
Q9VS66	CG8562 OS=Drosophila melanogaster GN=CG8562 PE=2 SV=1 - [Q9VS66_DROME]	0.00	7.08	1	1	1	1		424	47.7	4.97
Q9W2L2	LD27118p OS=Drosophila melanogaster GN=Magi PE=2 SV=2 - [Q9W2L2_DROME]	0.00	1.83	1	1	1	1		1202	130.5	6.51
Q7K0G5	CG8008, isoform A OS=Drosophila melanogaster GN=CG8008 PE=2 SV=1 - [Q7K0G5_DROME]	0.00	4.78	2	1	1	1	1.796E7	439	49.8	8.62
Q6NP69	GST-containing FLYWCH zinc-finger protein OS=Drosophila melanogaster GN=gfzf PE=2 SV=1 - [Q6NP69_DROME]	0.00	2.97	4	2	3	3	9.581E6	1045	119.2	5.99
Q7K1U0	Arc1 OS=Drosophila melanogaster GN=Arc1 PE=2 SV=1 - [Q7K1U0_DROME]	0.00	4.72	1	1	1	1	6.601E7	254	28.9	6.47
A1ZAC0	FI03676p OS=Drosophila melanogaster GN=Tsf3 PE=2 SV=1 - [A1ZAC0_DROME]	0.00	1.82	1	1	1	1	1.153E7	714	81.0	6.89
Q8MKK1	CG30185 OS=Drosophila melanogaster GN=CG30185;Gr59f PE=2 SV=1 - [Q8MKK1_DROME]	0.00	6.15	1	1	1	1		179	20.0	7.03
Q9VBV1	CG11854 (Fragment) OS=Drosophila melanogaster GN=CG11854-RA PE=2 SV=3 - [Q9VBV1_DROME]	0.00	8.70	1	1	1	1	4.098E7	253	28.2	7.23
Q9XYX1	HDAC6 OS=Drosophila melanogaster GN=HDAC6 PE=2 SV=1 - [Q9XYX1_DROME]	0.00	0.79	7	1	1	1	5.868E8	883	98.2	6.48
Q9VVG1	CG13728 OS=Drosophila melanogaster GN=CG13728-RA PE=2 SV=4 - [Q9VVG1_DROME]	0.00	2.66	1	1	1	1		753	85.7	9.48
Q9W3C3	CG2004, isoform A OS=Drosophila melanogaster GN=CG2004 PE=2 SV=1 - [Q9W3C3_DROME]	0.00	2.35	1	1	1	1	8.069E7	425	48.3	5.21
Q4V5A7	CG7229 OS=Drosophila melanogaster GN=CG7229 PE=2 SV=1 - [Q4V5A7_DROME]	0.00	5.93	1	1	1	1		674	74.6	9.52
Q8MY02	FI14518p OS=Drosophila melanogaster GN=pxb PE=2 SV=1 - [Q8MY02_DROME]	0.00	1.73	1	1	1	5		636	69.0	6.79
O46102	CG11418, isoform A OS=Drosophila melanogaster GN=fs(1)N PE=2 SV=2 - [O46102_DROME]	0.00	3.92	1	1	1	1		612	68.5	8.34
M9PCW4	CG44004, isoform B OS=Drosophila melanogaster GN=CG44004 PE=4 SV=2 - [M9PCW4_DROME]	0.00	2.99	1	1	1	1		368	43.3	7.96
Q9W362	LD09531p OS=Drosophila melanogaster GN=Larp7 PE=2 SV=1 - [Q9W362_DROME]	0.00	1.85	1	1	1	1	1.416E7	595	67.3	7.36
H8F4P0	FI18115p1 OS=Drosophila melanogaster GN=MED1 PE=2 SV=1 - [H8F4P0_DROME]	0.00	2.87	3	1	1	1		1080	111.4	9.19
Q3ZAQ1	CG7029, isoform B OS=Drosophila melanogaster GN=CG7029 PE=2 SV=1 - [Q3ZAQ1_DROME]	0.00	3.41	3	1	1	1	1.312E6	909	99.2	9.58
Q9I7M3	CG10237, isoform A OS=Drosophila melanogaster GN=CG10237 PE=4 SV=1 - [Q9I7M3_DROME]	0.00	8.64	3	2	2	2	1.761E7	301	35.1	8.51

Accession	Description	Score	Coverage	# Proteins	# Unique Peptides	# Peptides	# PSMs	Area	# AAs	MW [kDa]	calc. pI
Q8T0I9	CG11326-PC, isoform C OS=Drosophila melanogaster GN=Tsp PE=2 SV=1 - [Q8T0I9_DROME]	0.00	5.73	2	1	1	1		419	47.3	8.10
Q8IRH4	CG9181-PD, isoform D OS=Drosophila melanogaster GN=Ptp61F PE=4 SV=1 - [Q8IRH4_DROME]	0.00	3.02	2	1	1	1	6.665E6	431	48.5	5.20
Q9VTE1	CG32069-PA OS=Drosophila melanogaster GN=CG32069 PE=2 SV=2 - [Q9VTE1_DROME]	0.00	10.00	1	1	1	1		80	9.0	9.11
Q9VZG9	TipE homolog 2, isoform A OS=Drosophila melanogaster GN=Teh2 PE=2 SV=1 - [Q9VZG9_DROME]	0.00	5.83	2	1	1	1		309	34.2	6.13
Q9VBC4	AT02583p OS=Drosophila melanogaster GN=CG6420 PE=2 SV=2 - [Q9VBC4_DROME]	0.00	3.41	1	1	1	3	1.493E7	909	95.6	7.33
Q8SXS7	CG3734 OS=Drosophila melanogaster GN=CG3734 PE=2 SV=1 - [Q8SXS7_DROME]	0.00	3.17	1	1	1	1	1.308E7	473	53.2	5.02
Q9V415	BcDNA.LD23587 OS=Drosophila melanogaster GN=Hrd3 PE=2 SV=1 - [Q9V415_DROME]	0.00	3.17	1	1	1	1	1.460E6	819	89.3	5.60
M9PF73	CG43896, isoform C OS=Drosophila melanogaster GN=CG43896 PE=4 SV=1 - [M9PF73_DROME]	0.00	0.57	1	1	1	1	3.642E7	2113	229.2	4.59
Q9VNA3	CG11999 OS=Drosophila melanogaster GN=CG11999 PE=2 SV=1 - [Q9VNA3_DROME]	0.00	5.09	1	1	1	1		216	23.6	6.80
Q9W3D8	AT17652p OS=Drosophila melanogaster GN=CG12111 PE=2 SV=1 - [Q9W3D8_DROME]	0.00	6.91	1	1	1	1		188	21.8	5.59
Q7KUE8	CG6216 OS=Drosophila melanogaster GN=CG6216-RB PE=2 SV=1 - [Q7KUE8_DROME]	0.00	2.98	1	1	1	1	1.861E6	235	27.0	9.57
Q9VGW9	LD25118p OS=Drosophila melanogaster GN=mRpL37 PE=2 SV=3 - [Q9VGW9_DROME]	0.00	4.17	1	1	1	1	2.949E6	408	45.7	9.52
Q9VFE9	CG7265 OS=Drosophila melanogaster GN=CG7265 PE=2 SV=1 - [Q9VFE9_DROME]	0.00	5.33	1	1	1	1		469	52.1	5.25
Q0E8T7	CG34124 OS=Drosophila melanogaster GN=CG34124 PE=2 SV=1 - [Q0E8T7_DROME]	0.00	1.60	1	1	1	1		2003	232.3	5.38
Q9VM10	Scavenger receptor acting in neural tissue and majority of rhodopsin is absent, isoform A OS=Drosophila melanogaster GN=santa-maria PE=4 SV=2 - [Q9VM10_DROME]	0.00	6.04	1	1	1	1	1.692E6	563	62.9	5.67
Q9W0T7	CG16940, isoform A OS=Drosophila melanogaster GN=CG16940 PE=2 SV=2 - [Q9W0T7_DROME]	0.00	2.03	2	1	1	1		1032	116.8	8.18
Q9W1L1	Mitochondrial ribosomal protein L43 OS=Drosophila melanogaster GN=mRpL43 PE=2 SV=1 - [Q9W1L1_DROME]	0.00	14.06	1	1	1	1	2.888E7	192	22.0	9.26
A1ZAB3	FI18312p1 OS=Drosophila melanogaster GN=Ptp52F PE=2 SV=2 - [A1ZAB3_DROME]	0.00	2.79	1	1	1	1		1433	160.0	5.20
Q0KIE7	Ankyrin, isoform B OS=Drosophila melanogaster GN=Ank PE=2 SV=1 - [Q0KIE7_DROME]	0.00	0.71	1	1	1	1	5.490E7	1549	170.1	7.05
Q9VCX7	CG31156 OS=Drosophila melanogaster GN=CG31156 PE=2 SV=3 - [Q9VCX7_DROME]	0.00	5.06	1	2	2	3		948	106.4	8.87
Q9VF04	RE32990p OS=Drosophila melanogaster GN=Rbf2 PE=2 SV=1 - [Q9VF04_DROME]	0.00	1.40	1	1	1	1		783	89.8	8.00
B9A0M4	CG42355, isoform C OS=Drosophila melanogaster GN=CG42355-RB PE=2 SV=1 - [B9A0M4_DROME]	0.00	12.42	1	1	1	1		161	18.8	10.78
Q9VLY7	GH05679p OS=Drosophila melanogaster GN=Tep3 PE=2 SV=2 - [Q9VLY7_DROME]	0.00	0.41	1	1	1	1	9.714E7	1469	163.8	6.84
Q9VAH2	CG7837 OS=Drosophila melanogaster GN=CG7837 PE=2 SV=2 - [Q9VAH2_DROME]	0.00	3.18	1	1	1	2		975	110.6	5.77
Q9VZX0	CG9970-PA OS=Drosophila melanogaster GN=CG9970-RA PE=2 SV=2 - [Q9VZX0_DROME]	0.00	4.34	1	1	1	1		484	56.1	7.88
Q9VTW3	CG4328 OS=Drosophila melanogaster GN=CG4328-RA PE=2 SV=2 - [Q9VTW3_DROME]	0.00	4.41	1	1	1	1		544	59.6	7.88
Q7KUW6	Chascon, isoform B OS=Drosophila melanogaster GN=chas PE=4 SV=1 - [Q7KUW6_DROME]	0.00	2.91	3	1	1	1	1.201E6	1031	116.4	9.86
Q9W2E6	Poor Imd response upon knock-in OS=Drosophila melanogaster GN=pirk PE=2 SV=1 - [Q9W2E6_DROME]	0.00	6.60	1	1	1	1		197	21.2	9.14
Q9VU51	CG11280-PA OS=Drosophila melanogaster GN=trn PE=2 SV=1 - [Q9VU51_DROME]	0.00	2.04	2	1	1	1	1.624E6	737	81.9	7.58
Q9VK63	CG5776, isoform A OS=Drosophila melanogaster GN=CG5776 PE=2 SV=1 - [Q9VK63_DROME]	0.00	1.88	1	1	1	1	1.079E7	799	89.0	6.79
Q9VYH4	CG12715, isoform A OS=Drosophila melanogaster GN=CG12715-RA PE=2 SV=1 - [Q9VYH4_DROME]	0.00	4.75	1	1	1	1	5.661E8	505	58.7	6.61
Q9V9U9	CG11563 OS=Drosophila melanogaster GN=CG11563-RA PE=2 SV=2 - [Q9V9U9_DROME]	0.00	3.86	1	1	1	1		207	24.7	9.94
Q9VXA0	CG9104-PA OS=Drosophila melanogaster GN=CG9104 PE=2 SV=1 - [Q9VXA0_DROME]	0.00	3.40	1	1	1	1	2.753E6	412	46.3	8.21
Q9VKW2	LD12022p OS=Drosophila melanogaster GN=pie PE=2 SV=1 - [Q9VKW2_DROME]	0.00	1.89	1	1	1	2		582	66.0	5.80
Q9VA37	DJ-1 beta OS=Drosophila melanogaster GN=dj-1beta PE=1 SV=2 - [Q9VA37_DROME]	0.00	10.24	1	1	1	1	8.932E7	205	21.4	7.94
B7YZM9	Syndecan OS=Drosophila melanogaster GN=Sdc PE=2 SV=1 - [B7YZM9_DROME]	0.00	6.53	3	1	1	1		398	42.0	4.48
Q95TJ6	CG8907 OS=Drosophila melanogaster GN=CG8907 PE=2 SV=1 - [Q95TJ6_DROME]	0.00	2.81	1	1	1	2	4.013E7	712	80.1	6.34
Q9VH62	CG9467 OS=Drosophila melanogaster GN=CG9467 PE=2 SV=1 - [Q9VH62_DROME]	0.00	3.56	1	1	1	1	1.875E7	730	80.1	6.61
Q9VSP0	CG6511, isoform A OS=Drosophila melanogaster GN=CG6511 PE=2 SV=2 - [Q9VSP0_DROME]	0.00	2.73	1	2	2	2	3.982E6	1211	137.2	6.42
Q9VYR1	Regucalcin homologue OS=Drosophila melanogaster GN=regucalcin PE=2 SV=1 - [Q9VYR1_DROME]	0.00	3.96	2	1	1	1		303	33.6	6.40
Q9VG51	CG6359 OS=Drosophila melanogaster GN=Snx3 PE=2 SV=1 - [Q9VG51_DROME]	0.00	5.39	1	1	1	2	2.542E7	167	19.4	9.14
Q9VCS7	GH07323p OS=Drosophila melanogaster GN=Nha2 PE=2 SV=2 - [Q9VCS7_DROME]	0.00	1.20	1	1	1	1		584	62.7	8.92
Q9VF37	Crossover suppressor on 3 of Gowen OS=Drosophila melanogaster GN=c(3)G PE=2 SV=1 - [Q9VF37_DROME]	0.00	1.48	1	1	1	1	3.975E8	744	85.7	6.23
A1ZAQ7	Chitin deacetylase-like 9 OS=Drosophila melanogaster GN=Cda9 PE=2 SV=1 - [A1ZAQ7_DROME]	0.00	1.51	1	1	1	1	2.898E7	397	45.1	6.71
O76892	EG:4F1.1 protein OS=Drosophila melanogaster GN=Scgdelta PE=2 SV=1 - [O76892_DROME]	0.00	5.45	1	1	1	1		404	42.6	9.48
Q3YBY4	Virus-induced 1 OS=Drosophila melanogaster GN=vir-1 PE=2 SV=1 - [Q3YBY4_DROME]	0.00	5.16	3	1	1	1	5.969E5	155	18.3	4.13
Q9W2J4	CG4302 OS=Drosophila melanogaster GN=BEST:GH09393 PE=2 SV=2 - [Q9W2J4_DROME]	0.00	2.63	1	1	1	1	2.890E8	532	60.1	9.04
Q9VMK5	CG13997-PA OS=Drosophila melanogaster GN=Vm26Ac PE=2 SV=1 - [Q9VMK5_DROME]	0.00	11.05	1	1	1	2		181	18.5	8.35
Q9VMU2	CG5828, isoform A OS=Drosophila melanogaster GN=CG5828 PE=4 SV=1 - [Q9VMU2_DROME]	0.00	6.93	1	1	1	1		361	40.4	5.53
Q8SZU1	CG43755, isoform D OS=Drosophila melanogaster GN=CG33992 PE=2 SV=1 - [Q8SZU1_DROME]	0.00	3.44	1	1	1	1		407	46.0	8.25

Accession	Description	Score	Coverage	# Proteins	# Unique Peptides	# Peptides	# PSMs	Area	# AAs	MW [kDa]	calc. pI
O61722	CG4993-PA, isoform A OS=Drosophila melanogaster GN=PRL-1 PE=2 SV=2 - [O61722_DROME]	0.00	10.80	1	1	1	1		176	20.0	8.63
Q9VGN7	AT25352p OS=Drosophila melanogaster GN=CG6744 PE=2 SV=2 - [Q9VGN7_DROME]	0.00	2.57	1	1	1	1		583	66.8	8.81
Q9VID9	CG8677, isoform B OS=Drosophila melanogaster GN=CG8677 PE=4 SV=3 - [Q9VID9_DROME]	0.00	0.34	1	1	1	1	8.662E7	2663	292.4	5.27
Q9NCC3	Sorting nexin OS=Drosophila melanogaster GN=SH3PX1 PE=2 SV=1 - [Q9NCC3_DROME]	0.00	6.73	1	2	2	2	1.305E7	565	63.1	6.77
A8JV36	Trf4-1, isoform F OS=Drosophila melanogaster GN=Trf4-1 PE=4 SV=1 - [A8JV36_DROME]	0.00	2.87	2	1	1	1	1.942E6	802	88.0	7.61
Q9VC41	CG42331, isoform C OS=Drosophila melanogaster GN=CG42331-RC PE=2 SV=1 - [Q9VC41_DROME]	0.00	0.42	2	1	1	1	2.088E7	1439	159.9	7.27
Q9VCP5	CG13826 OS=Drosophila melanogaster GN=cnc PE=2 SV=2 - [Q9VCP5_DROME]	0.00	7.92	1	1	1	1	1.266E7	101	11.2	9.99
Q8MRG6	Precursor RNA processing 3, isoform B OS=Drosophila melanogaster GN=Prp3 PE=2 SV=1 - [Q8MRG6_DROME]	0.00	1.33	2	1	1	1	5.148E7	528	60.1	9.73
Q9VAE4	CG31036, isoform A OS=Drosophila melanogaster GN=CG31036 PE=2 SV=2 - [Q9VAE4_DROME]	0.00	1.29	2	1	1	1		1007	112.0	6.87
Q7JXC4	CG6459 OS=Drosophila melanogaster GN=P32 PE=2 SV=1 - [Q7JXC4_DROME]	0.00	4.56	1	1	1	1		263	29.0	5.08
Q8IR81	CG32652-PA OS=Drosophila melanogaster GN=CG32652 PE=2 SV=1 - [Q8IR81_DROME]	0.00	26.51	1	1	1	1		83	9.7	7.25
Q9VUZ6	FI05332p OS=Drosophila melanogaster GN=l(3)72Dn PE=2 SV=1 - [Q9VUZ6_DROME]	0.00	2.16	2	1	1	1	3.363E6	696	78.6	8.76
Q7K231	CG7747 OS=Drosophila melanogaster GN=CG7747 PE=2 SV=1 - [Q7K231_DROME]	0.00	2.13	1	1	1	1	2.885E6	517	58.9	8.13
Q8IR17	Gpi1, isoform B OS=Drosophila melanogaster GN=Gpi1 PE=4 SV=3 - [Q8IR17_DROME]	0.00	3.12	1	1	1	1		481	56.0	8.18
Q7PLT4	Tim17b, isoform A OS=Drosophila melanogaster GN=Tim17b PE=4 SV=1 - [Q7PLT4_DROME]	0.00	9.83	1	1	1	1	9.285E6	173	18.2	8.18
Q9VR29	CG3225 OS=Drosophila melanogaster GN=CG3225 PE=2 SV=2 - [Q9VR29_DROME]	0.00	3.24	1	1	1	1	7.272E5	678	76.1	7.97
Q0E930	CG30116, isoform B OS=Drosophila melanogaster GN=CG30116 PE=4 SV=1 - [Q0E930_DROME]	0.00	1.41	3	1	1	2		1698	190.5	6.68
Q9VGU6	GTP:AMP phosphotransferase, mitochondrial OS=Drosophila melanogaster GN=Adk3 PE=2 SV=1 - [Q9VGU6_DROME]	0.00	4.63	1	1	1	1	1.664E6	216	24.1	9.32
Q9VZ85	Rho guanine nucleotide exchange factor at 64C, isoform A OS=Drosophila melanogaster GN=RhoGEF64C PE=4 SV=2 - [Q9VZ85_DROME]	0.00	0.76	1	1	1	1		1984	214.8	6.80
Q9XYZ4	CG16928-PA OS=Drosophila melanogaster GN=mre11 PE=2 SV=1 - [Q9XYZ4_DROME]	0.00	3.87	1	1	1	1		620	69.2	5.95
Q9W1F6	Fizzy-related 2 OS=Drosophila melanogaster GN=fzr2 PE=2 SV=1 - [Q9W1F6_DROME]	0.00	2.44	1	1	1	5	1.615E7	451	50.9	8.90
Q9W3W4	COQ7 OS=Drosophila melanogaster GN=COQ7 PE=2 SV=2 - [Q9W3W4_DROME]	0.00	5.02	1	1	1	1	6.940E6	219	24.1	6.96
Q9V3S7	BcDNA.GH12326 OS=Drosophila melanogaster GN=BcDNA.GH12326 PE=2 SV=1 - [Q9V3S7_DROME]	0.00	2.80	1	1	1	1		429	47.8	9.58
Q6NR30	CG14478, isoform A OS=Drosophila melanogaster GN=BEST:LD13441 PE=2 SV=1 - [Q6NR30_DROME]	0.00	4.10	1	1	1	1		512	56.0	8.73
Q9VKJ6	CG6230-PA OS=Drosophila melanogaster GN=CG6230-RA PE=2 SV=1 - [Q9VKJ6_DROME]	0.00	1.63	1	2	2	2	1.096E7	1225	136.3	7.74
Q7K4R6	CG1868, isoform A OS=Drosophila melanogaster GN=CG1868 PE=2 SV=1 - [Q7K4R6_DROME]	0.00	1.81	2	1	1	1		718	83.0	6.34
Q9W4I7	Partner of numb OS=Drosophila melanogaster GN=pon PE=2 SV=1 - [Q9W4I7_DROME]	0.00	1.34	1	1	1	1		670	76.1	6.90
Q9VJ82	Kon-tiki, isoform B OS=Drosophila melanogaster GN=kon PE=4 SV=3 - [Q9VJ82_DROME]	0.00	1.13	1	1	1	1		2381	270.5	5.76
Q9VHN4	CG8043 OS=Drosophila melanogaster GN=CG8043 PE=2 SV=1 - [Q9VHN4_DROME]	0.00	5.75	1	1	1	1	9.959E6	348	39.5	8.68
Q9VVT6	CG6852, isoform C OS=Drosophila melanogaster GN=CG6852 PE=2 SV=1 - [Q9VVT6_DROME]	0.00	8.77	1	1	1	2	4.071E7	114	12.4	8.44
Q9W1H0	CG5591 OS=Drosophila melanogaster GN=Lpt PE=2 SV=2 - [Q9W1H0_DROME]	0.00	1.08	1	1	1	1	1.894E7	1482	164.4	6.99
Q9W0Q5	CG13891-PA OS=Drosophila melanogaster GN=CG13891-RA PE=2 SV=1 - [Q9W0Q5_DROME]	0.00	15.19	1	1	1	1		158	18.0	5.39
Q9VVV6	CG18135-PB, isoform B OS=Drosophila melanogaster GN=CG18135 PE=2 SV=2 - [Q9VVV6_DROME]	0.00	2.67	5	1	1	1	9.486E7	636	72.0	6.32
Q9VWC0	Histone gene-specific epigenetic repressor in late S phase, isoform A OS=Drosophila melanogaster GN=Hers PE=4 SV=2 - [Q9VWC0_DROME]	0.00	0.63	1	1	1	1		2529	270.5	9.10
Q7JQT9	CG1671 OS=Drosophila melanogaster GN=CG1671 PE=2 SV=1 - [Q7JQT9_DROME]	0.00	1.40	1	1	1	1	5.545E7	787	88.5	7.44
Q9VXQ2	GRAF ortholog, isoform A OS=Drosophila melanogaster GN=Graf PE=4 SV=3 - [Q9VXQ2_DROME]	0.00	2.36	2	1	1	1		1016	114.6	8.15
Q9VAI7	Odorant-binding protein 99d OS=Drosophila melanogaster GN=Obp99d PE=2 SV=1 - [Q9VAI7_DROME]	0.00	9.49	1	1	1	1		137	15.9	5.52
Q9I7S6	Phosphodiesterase 9, isoform B OS=Drosophila melanogaster GN=Pde9 PE=4 SV=2 - [Q9I7S6_DROME]	0.00	0.98	2	1	1	1	8.164E7	1526	162.7	6.20
Q9VIF2	CG9248, isoform A OS=Drosophila melanogaster GN=CG9248 PE=2 SV=1 - [Q9VIF2_DROME]	0.00	3.64	1	1	1	1		494	55.6	6.14
D0IQA3	CG42570, isoform A OS=Drosophila melanogaster GN=CG42570 PE=2 SV=1 - [D0IQA3_DROME]	0.00	3.44	1	1	1	1		610	70.6	5.44
O18475	FI03732p OS=Drosophila melanogaster GN=mus308 PE=2 SV=1 - [O18475_DROME]	0.00	0.63	1	1	1	1	7.961E7	2059	229.7	7.17
Q9VVQ5	CG18234 OS=Drosophila melanogaster GN=CG18234 PE=2 SV=4 - [Q9VVQ5_DROME]	0.00	4.08	1	1	1	1	4.864E6	515	58.9	5.72
Q6NPB4	CG1092, isoform B OS=Drosophila melanogaster GN=CG1092 PE=2 SV=1 - [Q6NPB4_DROME]	0.00	7.62	1	1	1	1		315	38.0	6.80
B5X533	CG8909, isoform C OS=Drosophila melanogaster GN=CG8909-RB PE=2 SV=1 - [B5X533_DROME]	0.00	1.34	2	1	1	1	1.613E7	2008	225.6	6.25
Q9VI64	CG10098 OS=Drosophila melanogaster GN=CG10098 PE=2 SV=1 - [Q9VI64_DROME]	0.00	4.53	1	1	1	1		353	37.5	4.79
Q9W275	Alkaline phosphatase OS=Drosophila melanogaster GN=CG3292 PE=2 SV=1 - [Q9W275_DROME]	0.00	2.21	1	1	1	1		543	58.2	5.14
Q8MRA3	Hibris, isoform A OS=Drosophila melanogaster GN=hbs PE=2 SV=1 - [Q8MRA3_DROME]	0.00	1.06	2	1	1	1	1.791E8	1228	133.5	7.01
Q9W087	CG2021-PA OS=Drosophila melanogaster GN=CG2021 PE=1 SV=1 - [Q9W087_DROME]	0.00	7.37	2	1	1	1	1.107E7	95	10.3	4.92
Q7JW03	CG2257 OS=Drosophila melanogaster GN=UbcE2H PE=2 SV=1 - [Q7JW03_DROME]	0.00	4.37	1	1	1	1	8.658E6	183	20.9	4.79
M9PBY4	CG11455, isoform H OS=Drosophila melanogaster GN=CG11455 PE=4 SV=1 - [M9PBY4_DROME]	0.00	15.71	2	1	1	1	1.274E7	70	8.5	7.85

Accession	Description	Score	Coverage	# Proteins	# Unique Peptides	# Peptides	# PSMs	Area	# AAs	MW [kDa]	calc. pI
Q9VG46	Lipase OS=Drosophila melanogaster GN=CG6753-RB PE=2 SV=2 - [Q9VG46_DROME]	0.00	2.22	1	1	1	1	6.783E7	405	47.0	9.26
Q9VC90	CG5746, isoform A OS=Drosophila melanogaster GN=CG5746 PE=4 SV=2 - [Q9VC90_DROME]	0.00	4.38	2	2	2	2	1.348E9	457	46.5	9.73
Q8T8S2	Uridine phosphorylase OS=Drosophila melanogaster GN=CG3788 PE=2 SV=1 - [Q8T8S2_DROME]	0.00	3.97	1	1	1	1	3.318E7	378	41.6	5.95
Q8SYP9	Mitochondrial ribosomal protein S5, isoform A OS=Drosophila melanogaster GN=mRpS5 PE=2 SV=1 - [Q8SYP9_DROME]	0.00	5.90	1	1	1	1		407	46.0	10.20
Q9VVJ0	CG7510, isoform A OS=Drosophila melanogaster GN=CG7510 PE=2 SV=2 - [Q9VVJ0_DROME]	0.00	2.74	2	1	1	1		949	105.0	8.32
Q76NQ0	Dolichyl-diphosphooligosaccharide--protein glycosyltransferase subunit 1 OS=Drosophila melanogaster GN=CG33303 PE=3 SV=1 - [Q76NQ0_DROME]	0.00	6.77	1	2	2	2	2.417E7	458	51.7	8.05
Q9VM55	Uninflatable, isoform B OS=Drosophila melanogaster GN=uif PE=4 SV=3 - [Q9VM55_DROME]	0.00	0.82	2	1	1	1	1.091E6	3557	386.9	5.30
Q9VZ84	CG7509 OS=Drosophila melanogaster GN=CG7509 PE=2 SV=2 - [Q9VZ84_DROME]	0.00	4.39	1	1	1	1		615	69.7	6.67
Q9W4Z9	CG3071-PA OS=Drosophila melanogaster GN=EG:25E8.3 PE=2 SV=1 - [Q9W4Z9_DROME]	0.00	3.74	1	2	2	2	3.801E7	535	60.3	9.32
Q7K1M4	CG1902, isoform A OS=Drosophila melanogaster GN=CG1902 PE=2 SV=1 - [Q7K1M4_DROME]	0.00	4.81	1	1	1	1	6.115E6	312	35.8	6.20
Q9VJZ5	LD07294p OS=Drosophila melanogaster GN=Tap42 PE=2 SV=2 - [Q9VJZ5_DROME]	0.00	7.37	1	1	1	1	4.977E7	380	43.3	5.40
Q4V6X9	CG17385 OS=Drosophila melanogaster GN=CG17385 PE=2 SV=1 - [Q4V6X9_DROME]	0.00	10.43	1	1	1	1		278	32.4	8.19
Q86P21	CG31721-PA OS=Drosophila melanogaster GN=Trim9 PE=2 SV=1 - [Q86P21_DROME]	0.00	1.92	2	1	1	2	2.068E6	729	79.0	5.91
Q9VBR0	CG4553 OS=Drosophila melanogaster GN=CG4553 PE=2 SV=1 - [Q9VBR0_DROME]	0.00	4.13	1	1	1	1	2.485E7	363	42.3	9.11
Q8IQG3	Vacuolar H[+] ATPase 16kD subunit 3, isoform A OS=Drosophila melanogaster GN=Vha16-3 PE=3 SV=1 - [Q8IQG3_DROME]	0.00	11.39	1	1	1	2	7.736E7	158	16.1	9.23
Q7JR71	Superoxide dismutase [Cu-Zn] OS=Drosophila melanogaster GN=Sod3 PE=2 SV=1 - [Q7JR71_DROME]	0.00	3.87	3	1	1	1	2.785E8	181	19.2	6.51
Q9VW59	LD16419p OS=Drosophila melanogaster GN=RhoGDI PE=2 SV=1 - [Q9VW59_DROME]	0.00	3.48	1	1	1	1	2.157E7	201	23.2	5.64
Q9VX29	CG12990 OS=Drosophila melanogaster GN=CG12990 PE=2 SV=2 - [Q9VX29_DROME]	0.00	1.96	1	1	1	1		663	76.0	7.72
Q9VUU6	CG12713 OS=Drosophila melanogaster GN=CG12713 PE=2 SV=2 - [Q9VUU6_DROME]	0.00	3.04	1	1	1	1		329	37.9	7.69
Q9VV09	CG4950, isoform B OS=Drosophila melanogaster GN=CG4950 PE=4 SV=2 - [Q9VV09_DROME]	0.00	3.66	1	1	1	1		574	65.1	6.34
Q8T3J1	CG31125 OS=Drosophila melanogaster GN=CG31125 PE=2 SV=1 - [Q8T3J1_DROME]	0.00	3.58	1	1	1	1		391	44.1	6.81
Q9VH45	CG5359, isoform A OS=Drosophila melanogaster GN=Dlc90F PE=2 SV=1 - [Q9VH45_DROME]	0.00	5.78	1	1	1	1		173	19.1	5.39
Q9VKX8	Dpr19, isoform A OS=Drosophila melanogaster GN=dpr19 PE=1 SV=2 - [Q9VKX8_DROME]	0.00	7.01	1	1	1	1		385	42.2	9.33
Q9VDX1	CG11626 OS=Drosophila melanogaster GN=CG11626-RB PE=2 SV=2 - [Q9VDX1_DROME]	0.00	6.60	1	1	1	1		379	42.7	5.31
Q9VY42	CG1461, isoform A OS=Drosophila melanogaster GN=CG1461 PE=2 SV=1 - [Q9VY42_DROME]	0.00	3.39	1	1	1	1		501	55.9	6.25
Q9VYK0	Smrter, isoform D OS=Drosophila melanogaster GN=Smr PE=4 SV=3 - [Q9VYK0_DROME]	0.00	1.00	2	2	2	2		3601	378.9	9.44
Q7K0L5	LP01562p OS=Drosophila melanogaster GN=veil PE=2 SV=1 - [Q7K0L5_DROME]	0.00	2.17	1	1	1	1	3.703E6	599	65.4	4.96
Q7K0P0	Juvenile hormone-inducible protein 26 OS=Drosophila melanogaster GN=JhI-26 PE=2 SV=1 - [Q7K0P0_DROME]	0.00	6.38	1	1	1	1	2.061E7	439	50.9	5.24
Q9VZU8	CG1291-PA OS=Drosophila melanogaster GN=CG1291 PE=2 SV=1 - [Q9VZU8_DROME]	0.00	2.59	1	1	1	1		424	48.6	8.15
Q9VK19	CG15481-PA OS=Drosophila melanogaster GN=Ski6 PE=2 SV=1 - [Q9VK19_DROME]	0.00	4.07	1	1	1	1	3.101E6	246	27.0	6.07
Q9VS47	CG8602, isoform B OS=Drosophila melanogaster GN=CG8602 PE=2 SV=1 - [Q9VS47_DROME]	0.00	2.09	1	1	1	1	4.434E7	478	53.3	6.39
Q9W139	CG3565 OS=Drosophila melanogaster GN=CG3565 PE=2 SV=1 - [Q9W139_DROME]	0.00	5.74	1	1	1	1	8.483E6	244	28.4	5.12
Q8MQQ0	CG10175, isoform B OS=Drosophila melanogaster GN=CG10175 PE=2 SV=1 - [Q8MQQ0_DROME]	0.00	4.77	3	1	1	2		524	58.8	6.23
A1Z7T4	FI18952p1 OS=Drosophila melanogaster GN=Pkn PE=2 SV=1 - [A1Z7T4_DROME]	0.00	1.53	7	1	1	1	2.993E7	590	66.2	5.39
Q7JR73	LD20239p OS=Drosophila melanogaster GN=Rcd6 PE=2 SV=1 - [Q7JR73_DROME]	0.00	3.31	1	1	1	1		513	56.8	8.82
Q9W3N1	CG1444 OS=Drosophila melanogaster GN=CG1444 PE=2 SV=1 - [Q9W3N1_DROME]	0.00	6.85	1	2	2	2	3.538E7	321	35.1	9.47
Q9VDZ1	CG5555 OS=Drosophila melanogaster GN=CG5555 PE=2 SV=2 - [Q9VDZ1_DROME]	0.00	1.62	1	1	1	1		555	63.1	6.14
Q9VF20	Heavy metal tolerance factor 1 OS=Drosophila melanogaster GN=Hmt-1 PE=2 SV=1 - [Q9VF20_DROME]	0.00	3.12	1	1	1	1	5.379E8	866	97.8	8.37
Q9VWP1	CG7288 OS=Drosophila melanogaster GN=CG7288 PE=2 SV=2 - [Q9VWP1_DROME]	0.00	3.64	1	1	1	1	1.258E7	494	57.5	8.46
Q9VHW4	FI21225p1 OS=Drosophila melanogaster GN=unc-45 PE=2 SV=1 - [Q9VHW4_DROME]	0.00	2.75	1	2	2	2	4.890E6	947	105.0	5.78
Q9VUM4	AT16346p OS=Drosophila melanogaster GN=CG6888 PE=2 SV=1 - [Q9VUM4_DROME]	0.00	12.24	1	1	1	1	1.182E8	196	22.0	5.95
M9ND79	CG43064, isoform A OS=Drosophila melanogaster GN=CG43064 PE=4 SV=1 - [M9ND79_DROME]	0.00	4.37	1	1	1	1		481	54.7	7.40
Q9Y171	BcDNA.GH02220 OS=Drosophila melanogaster GN=BcDNA.GH02220 PE=2 SV=1 - [Q9Y171_DROME]	0.00	3.17	1	1	1	1		441	48.7	9.55
Q9VWL0	Mec2 OS=Drosophila melanogaster GN=Mec2 PE=2 SV=1 - [Q9VWL0_DROME]	0.00	7.43	1	1	1	1	7.957E5	350	38.6	5.25
O62531	AP-47 OS=Drosophila melanogaster GN=AP-1mu PE=2 SV=1 - [O62531_DROME]	0.00	2.82	1	1	1	2		426	48.9	7.18
Q7JUP3	Caspar, isoform A OS=Drosophila melanogaster GN=casp PE=2 SV=1 - [Q7JUP3_DROME]	0.00	1.73	1	1	1	1	3.017E7	695	78.4	4.89
Q9W044	CG13810 OS=Drosophila melanogaster GN=CG13810-RA PE=2 SV=2 - [Q9W044_DROME]	0.00	5.31	1	1	1	1		490	55.4	8.02
Q9W1D9	Oxysterol-binding protein OS=Drosophila melanogaster GN=CG3860 PE=2 SV=1 - [Q9W1D9_DROME]	0.00	3.74	1	1	1	1	5.894E6	455	52.2	7.15
Q9VL10	AT10584p OS=Drosophila melanogaster GN=Mulk PE=2 SV=2 - [Q9VL10_DROME]	0.00	6.86	1	1	1	1	5.074E6	408	45.7	8.37
Q8IQZ1	Glutathione synthetase OS=Drosophila melanogaster GN=GS PE=2 SV=2 - [Q8IQZ1_DROME]	0.00	6.52	5	2	2	2	2.442E6	491	54.6	6.01

Accession	Description	Score	Coverage	# Proteins	# Unique Peptides	# Peptides	# PSMs	Area	# AAs	MW [kDa]	calc. pI
Q9VQB6	Ataxin 7, isoform A OS=Drosophila melanogaster GN=Atxn7 PE=2 SV=1 - [Q9VQB6_DROME]	0.00	1.96	1	1	1	1	3.032E6	971	104.1	6.98
Q9VLQ9	LD22082p OS=Drosophila melanogaster GN=Snx6 PE=2 SV=2 - [Q9VLQ9_DROME]	0.00	3.56	2	1	1	1	5.953E6	449	50.1	6.73
Q9VWS4	CG6531-PA OS=Drosophila melanogaster GN=wgn PE=1 SV=2 - [Q9VWS4_DROME]	0.00	4.37	1	1	1	1		343	37.8	8.50
Q7KW02	Enhanced adult sensory threshold, isoform B OS=Drosophila melanogaster GN=east PE=4 SV=1 - [Q7KW02_DROME]	0.00	0.48	3	1	1	1	2.268E7	2301	245.6	6.04
O76863	EG:100G10.3 protein OS=Drosophila melanogaster GN=eIF2B-beta PE=2 SV=1 - [O76863_DROME]	0.00	4.83	1	1	1	1	2.658E6	352	39.1	6.27
Q9W253	LD41023p OS=Drosophila melanogaster GN=mRpS29 PE=2 SV=1 - [Q9W253_DROME]	0.00	2.55	1	1	1	1	1.822E7	392	44.3	8.41
Q9W4R2	CG2875, isoform B OS=Drosophila melanogaster GN=CG2875-RB PE=2 SV=1 - [Q9W4R2_DROME]	0.00	2.22	2	1	1	1	8.984E6	496	56.6	8.21
Q9VZ55	CG1582, isoform A OS=Drosophila melanogaster GN=CG1582 PE=2 SV=2 - [Q9VZ55_DROME]	0.00	1.79	1	2	2	3		1288	146.0	7.40
Q9VLS4	CG8498, isoform B OS=Drosophila melanogaster GN=CG8498 PE=2 SV=1 - [Q9VLS4_DROME]	0.00	21.11	1	1	1	1	1.748E7	90	9.9	5.45
Q9VB22	LD33695p OS=Drosophila melanogaster GN=pins PE=1 SV=1 - [Q9VB22_DROME]	0.00	1.52	1	1	1	1		658	71.4	6.74
Q9W0J9	CG9119 OS=Drosophila melanogaster GN=CG9119 PE=2 SV=1 - [Q9W0J9_DROME]	0.00	9.32	1	1	1	1		322	35.6	6.38
Q1WWD1	IP14862p OS=Drosophila melanogaster GN=nompA PE=2 SV=1 - [Q1WWD1_DROME]	0.00	2.43	3	1	1	1	2.225E7	1027	116.8	6.29
Q9VEB4	CG7175 OS=Drosophila melanogaster GN=mTerf5 PE=2 SV=1 - [Q9VEB4_DROME]	0.00	3.39	1	1	1	2		560	64.8	9.03
O97066	GM04682p OS=Drosophila melanogaster GN=twr PE=2 SV=1 - [O97066_DROME]	0.00	5.41	1	1	1	1		185	21.0	8.85
Q29QP5	CG12914 OS=Drosophila melanogaster GN=CG12914 PE=2 SV=1 - [Q29QP5_DROME]	0.00	10.59	1	1	1	1		255	29.9	9.64
Q5BI31	CG12179, isoform C OS=Drosophila melanogaster GN=CG12179 PE=2 SV=1 - [Q5BI31_DROME]	0.00	1.51	1	1	1	1		1322	148.0	9.31
Q9W0N3	CKII-alpha subunit interactor-3, isoform A OS=Drosophila melanogaster GN=CkIIalpha-i3 PE=2 SV=1 - [Q9W0N3_DROME]	0.00	2.58	2	1	1	1	4.218E6	387	44.3	8.88
Q9VZV2	Cht7 OS=Drosophila melanogaster GN=Cht7 PE=2 SV=3 - [Q9VZV2_DROME]	0.00	1.97	1	1	1	5		1013	113.2	6.33
Q9W1N3	Levy OS=Drosophila melanogaster GN=levy PE=2 SV=1 - [Q9W1N3_DROME]	0.00	7.34	1	1	1	1		109	12.5	9.70
Q86CW5	Hmr OS=Drosophila melanogaster GN=Hmr PE=4 SV=1 - [Q86CW5_DROME]	0.00	0.64	1	1	1	1	2.332E8	1413	158.5	9.55
Q7JR83	CG11055, isoform A OS=Drosophila melanogaster GN=Hsl PE=2 SV=1 - [Q7JR83_DROME]	0.00	1.82	1	1	1	1	2.640E7	881	97.4	5.33
Q9V3A7	Structural maintenance of chromosomes protein OS=Drosophila melanogaster GN=glu PE=2 SV=1 - [Q9V3A7_DROME]	0.00	0.92	1	1	1	1		1409	159.8	6.15
Q9VZN2	CG32264, isoform B OS=Drosophila melanogaster GN=CG12604 PE=2 SV=2 - [Q9VZN2_DROME]	0.00	3.04	1	1	1	1		790	87.9	9.20
Q9VMQ6	CG31648 OS=Drosophila melanogaster GN=CG31648 PE=2 SV=2 - [Q9VMQ6_DROME]	0.00	2.49	1	1	1	2	1.470E8	241	27.3	7.71
Q8IQV2	CG32221 OS=Drosophila melanogaster GN=CG32221-RA PE=2 SV=2 - [Q8IQV2_DROME]	0.00	3.94	1	1	1	1	1.088E7	432	50.4	7.84
Q9VCW3	LP09464p OS=Drosophila melanogaster GN=Nup133 PE=2 SV=2 - [Q9VCW3_DROME]	0.00	1.67	1	2	2	2		1200	135.1	5.63
Q8SZ78	CG11318 OS=Drosophila melanogaster GN=CG11318 PE=2 SV=1 - [Q8SZ78_DROME]	0.00	3.87	1	1	1	1	1.006E8	802	90.4	7.25
Q9VCD3	CG13605 OS=Drosophila melanogaster GN=CG13605 PE=2 SV=2 - [Q9VCD3_DROME]	0.00	2.84	1	1	1	1	4.245E6	669	72.0	6.74
Q9VL16	CG5676-PA OS=Drosophila melanogaster GN=CG5676 PE=2 SV=1 - [Q9VL16_DROME]	0.00	5.13	1	1	1	1	9.354E7	156	17.3	9.20
Q9VD14	CG13850 OS=Drosophila melanogaster GN=CG13850 PE=2 SV=3 - [Q9VD14_DROME]	0.00	2.84	1	1	1	1		564	61.8	9.13
Q86PF5	CG6167-PB, isoform B OS=Drosophila melanogaster GN=PICK1 PE=4 SV=1 - [Q86PF5_DROME]	0.00	1.44	3	1	1	1	5.869E7	487	54.5	5.15
Q9W541	EIF2B-epsilon protein OS=Drosophila melanogaster GN=eIF2B-epsilon PE=2 SV=1 - [Q9W541_DROME]	0.00	6.73	1	1	1	1		669	75.0	5.07
Q9VLZ8	Serpin 28B, isoform A OS=Drosophila melanogaster GN=Spn28B PE=3 SV=2 - [Q9VLZ8_DROME]	0.00	6.08	1	1	1	2	9.802E6	378	43.2	9.48
Q9W1V8	CG9893 protein OS=Drosophila melanogaster GN=l(2)06496 PE=2 SV=1 - [Q9W1V8_DROME]	0.00	15.63	1	2	2	3		192	21.5	4.67
Q9VC63	CG5789 OS=Drosophila melanogaster GN=CG5789-RA PE=2 SV=2 - [Q9VC63_DROME]	0.00	1.14	1	1	1	1		1402	157.0	6.79
Q9VFE7	CG7262 OS=Drosophila melanogaster GN=Nup93-2 PE=2 SV=1 - [Q9VFE7_DROME]	0.00	0.88	1	1	1	1		796	90.5	7.15
Q9XYZ9	CG16936 OS=Drosophila melanogaster GN=GstE12 PE=2 SV=1 - [Q9XYZ9_DROME]	0.00	7.62	1	2	2	2	4.953E7	223	25.2	6.29
A4V0X6	CG10947, isoform B OS=Drosophila melanogaster GN=CG10947-RB PE=2 SV=1 - [A4V0X6_DROME]	0.00	4.46	1	1	1	1	4.969E5	404	45.7	5.45
Q9VE77	V-type proton ATPase subunit a OS=Drosophila melanogaster GN=Vha100-4 PE=2 SV=1 - [Q9VE77_DROME]	0.00	2.73	1	1	1	1	1.828E6	844	96.6	6.51
Q9VTV2	CG11529 OS=Drosophila melanogaster GN=CG11529 PE=2 SV=1 - [Q9VTV2_DROME]	0.00	11.15	1	1	1	1		287	31.8	7.36
Q9W3H4	CG10964-PA OS=Drosophila melanogaster GN=sni PE=1 SV=1 - [Q9W3H4_DROME]	0.00	8.10	1	1	1	1		247	26.5	8.65
Q9VQS1	CG8851, isoform A OS=Drosophila melanogaster GN=CG8851 PE=2 SV=2 - [Q9VQS1_DROME]	0.00	3.42	2	2	2	2	7.875E6	644	74.4	9.54
Q9VVP2	CG7320 OS=Drosophila melanogaster GN=CG7320-RA PE=2 SV=3 - [Q9VVP2_DROME]	0.00	2.63	1	1	1	1		646	77.1	6.07
Q7KIS4	SP2637 OS=Drosophila melanogaster GN=SP2637 PE=2 SV=1 - [Q7KIS4_DROME]	0.00	6.45	2	1	1	1	3.762E7	310	34.5	5.36
Q86LG7	Pickpocket 16 OS=Drosophila melanogaster GN=ppk16 PE=2 SV=1 - [Q86LG7_DROME]	0.00	8.10	1	1	1	1		531	61.4	8.38
P91634	PI-3 kinase OS=Drosophila melanogaster GN=Pi3K92E PE=2 SV=1 - [P91634_DROME]	0.00	1.47	1	1	1	1		1088	126.9	7.58
Q6XK20	Bitesize isoform 1 OS=Drosophila melanogaster GN=btsz PE=2 SV=1 - [Q6XK20_DROME]	0.00	1.54	7	2	2	2	9.858E7	1102	121.4	8.91
Q7K509	CG8708, isoform A OS=Drosophila melanogaster GN=CG8708 PE=2 SV=1 - [Q7K509_DROME]	0.00	9.68	2	1	1	1	1.149E6	310	35.5	8.60
Q9W4J6	CG11436 OS=Drosophila melanogaster GN=CG11436 PE=2 SV=1 - [Q9W4J6_DROME]	0.00	7.53	1	1	1	1		465	53.8	8.63
Q7JVP4	CG1845 OS=Drosophila melanogaster GN=Br140 PE=2 SV=1 - [Q7JVP4_DROME]	0.00	2.31	1	1	1	1		1430	157.0	7.14

Accession	Description	Score	Coverage	# Proteins	# Unique Peptides	# Peptides	# PSMs	Area	# AAs	MW [kDa]	calc. pI
Q7JWR4	CG13319, isoform A OS=Drosophila melanogaster GN=CG13319 PE=2 SV=1 - [Q7JWR4_DROME]	0.00	20.45	1	1	1	2		132	14.3	8.50
Q9VXZ0	CG5548, isoform B OS=Drosophila melanogaster GN=CG5548 PE=2 SV=1 - [Q9VXZ0_DROME]	0.00	12.82	1	2	2	3	8.488E6	117	13.9	7.81
Q8SY35	CG8213, isoform B OS=Drosophila melanogaster GN=CG8213 PE=2 SV=1 - [Q8SY35_DROME]	0.00	1.37	2	1	1	1		1674	180.5	6.79
Q00G30	Sno oncogene, isoform B OS=Drosophila melanogaster GN=Snoo PE=2 SV=1 - [Q00G30_DROME]	0.00	0.57	1	1	1	1	3.412E8	1223	135.0	5.96
Q9VUJ2	FI21605p1 OS=Drosophila melanogaster GN=Reck PE=2 SV=2 - [Q9VUJ2_DROME]	0.00	5.14	1	2	2	2		1071	117.7	7.02
Q86BM0	CG9515, isoform B OS=Drosophila melanogaster GN=CG9515 PE=3 SV=1 - [Q86BM0_DROME]	0.00	5.26	1	1	1	1		209	22.9	5.44
Q7K2N0	CG8735 OS=Drosophila melanogaster GN=CG8735 PE=2 SV=1 - [Q7K2N0_DROME]	0.00	4.65	1	1	1	1	1.388E7	387	43.6	9.13
Q9VTZ4	LD02044p OS=Drosophila melanogaster GN=nst PE=2 SV=1 - [Q9VTZ4_DROME]	0.00	3.28	1	1	1	1	1.650E8	549	60.5	6.29
Q9VFX6	CG8449 OS=Drosophila melanogaster GN=CG8449 PE=2 SV=1 - [Q9VFX6_DROME]	0.00	0.92	2	1	1	1		654	74.3	6.39
Q9VZX9	Adenosylhomocysteinase OS=Drosophila melanogaster GN=CG9977-RA PE=2 SV=1 - [Q9VZX9_DROME]	0.00	3.45	1	1	1	1		521	56.9	6.79
Q9VVF0	AT21479p OS=Drosophila melanogaster GN=CG6485 PE=2 SV=1 - [Q9VVF0_DROME]	0.00	7.56	1	1	1	1	1.366E6	238	26.7	8.90
Q7JQ32	Guanylate cyclase OS=Drosophila melanogaster GN=Gyc76C PE=2 SV=1 - [Q7JQ32_DROME]	0.00	0.72	1	1	1	1	1.060E7	1525	170.4	7.23
Q9VFC2	GH14439p OS=Drosophila melanogaster GN=Spn88Ea PE=2 SV=1 - [Q9VFC2_DROME]	0.00	2.11	1	1	1	1	3.377E7	427	48.4	5.55
Q0E908	Hillarin, isoform A OS=Drosophila melanogaster GN=Hil PE=4 SV=1 - [Q0E908_DROME]	0.00	2.20	1	1	1	1		818	94.4	7.28
Q9VB83	CG14257 OS=Drosophila melanogaster GN=CG14257 PE=2 SV=2 - [Q9VB83_DROME]	0.00	3.29	1	1	1	1	1.121E7	547	57.6	8.10
A1Z6H9	Dpr12 OS=Drosophila melanogaster GN=dpr12 PE=1 SV=3 - [A1Z6H9_DROME]	0.00	2.76	1	1	1	1	8.315E6	326	36.9	8.27
Q9VJ27	CG10431, isoform A OS=Drosophila melanogaster GN=CG10431 PE=4 SV=3 - [Q9VJ27_DROME]	0.00	4.33	1	1	1	1		762	87.2	8.91
Q24216	Cyclin-dependent kinase 7 OS=Drosophila melanogaster GN=Cdk7 PE=2 SV=1 - [Q24216_DROME]	0.00	7.37	1	1	1	1		353	39.6	9.45
Q9VEV7	CG17565 OS=Drosophila melanogaster GN=CG17565 PE=2 SV=1 - [Q9VEV7_DROME]	0.00	2.39	1	1	1	1	2.589E7	419	46.7	5.39
Q7JQV9	CG10953 OS=Drosophila melanogaster GN=CG10953 PE=2 SV=1 - [Q7JQV9_DROME]	0.00	8.27	1	1	1	1	2.363E8	278	28.4	9.04
Q9VSM8	FI07835p OS=Drosophila melanogaster GN=GAPsec PE=2 SV=1 - [Q9VSM8_DROME]	0.00	5.96	1	1	1	1	9.282E5	403	45.8	5.73
Q9VUM1	LD41209p OS=Drosophila melanogaster GN=Prp31 PE=2 SV=1 - [Q9VUM1_DROME]	0.00	2.59	1	1	1	1		501	55.5	7.09
Q9VZY0	LD45195p OS=Drosophila melanogaster GN=Non2 PE=2 SV=1 - [Q9VZY0_DROME]	0.00	4.51	1	1	1	2	1.635E7	244	27.5	6.32
Q9W0R7	Ada2a-containing complex component 3, isoform B OS=Drosophila melanogaster GN=Atac3 PE=4 SV=3 - [Q9W0R7_DROME]	0.00	3.30	2	1	1	1	7.692E6	576	63.2	7.99
Q7JVY2	Double parked OS=Drosophila melanogaster GN=dup PE=1 SV=1 - [Q7JVY2_DROME]	0.00	0.81	1	1	1	1	4.685E6	743	83.4	9.76
Q9VR17	Blastoderm-specific gene 25A OS=Drosophila melanogaster GN=Bsg25A PE=2 SV=2 - [Q9VR17_DROME]	0.00	5.51	1	1	1	1		363	41.6	5.16
Q9VJ28	CG10639, isoform A OS=Drosophila melanogaster GN=CG10639-RA PE=2 SV=1 - [Q9VJ28_DROME]	0.00	2.64	1	1	1	1		455	50.1	8.59
Q7KSQ2	C-terminal Src kinase, isoform F OS=Drosophila melanogaster GN=Csk PE=4 SV=1 - [Q7KSQ2_DROME]	0.00	2.77	3	1	1	1	1.161E7	723	78.0	7.47
Q9VNC3	Holocarboxylase synthetase OS=Drosophila melanogaster GN=Hcs PE=2 SV=2 - [Q9VNC3_DROME]	0.00	3.55	1	1	1	1		1041	115.9	7.08
Q9VKC3	CG14945, isoform B OS=Drosophila melanogaster GN=CG14945 PE=4 SV=1 - [Q9VKC3_DROME]	0.00	2.07	1	1	1	1	1.135E9	482	54.8	6.90
Q9VAR6	CG14521 OS=Drosophila melanogaster GN=CG14521 PE=1 SV=1 - [Q9VAR6_DROME]	0.00	7.02	1	1	1	1		413	45.3	8.50
Q9VSL2	Glutathione S transferase O3 OS=Drosophila melanogaster GN=GstO3 PE=2 SV=1 - [Q9VSL2_DROME]	0.00	9.54	1	1	1	1		241	27.7	6.95
Q494G1	CG9864 OS=Drosophila melanogaster GN=CG9864 PE=2 SV=1 - [Q494G1_DROME]	0.00	7.74	1	1	1	1		465	51.4	7.62
Q9VY08	CG9518, isoform A OS=Drosophila melanogaster GN=CG9518-RA PE=2 SV=1 - [Q9VY08_DROME]	0.00	3.27	1	1	1	1		703	78.5	9.33
Q7KT73	CG18477 OS=Drosophila melanogaster GN=CG18477 PE=2 SV=1 - [Q7KT73_DROME]	0.00	2.80	1	1	1	1		464	51.4	5.16
Q9VMC7	CG31638, isoform A OS=Drosophila melanogaster GN=CG9545 PE=2 SV=3 - [Q9VMC7_DROME]	0.00	1.14	1	1	1	1	1.776E7	704	79.7	6.80
Q4ABH5	CG33771 OS=Drosophila melanogaster GN=CG33771 PE=4 SV=3 - [Q4ABH5_DROME]	0.00	12.92	1	1	1	1		178	20.6	9.57
Q8IMP1	CG31064, isoform A OS=Drosophila melanogaster GN=CG31064 PE=4 SV=1 - [Q8IMP1_DROME]	0.00	3.75	3	1	1	1		667	73.4	5.96
A1ZA72	Stretchin-Mlck, isoform D OS=Drosophila melanogaster GN=Strn-Mlck PE=4 SV=1 - [A1ZA72_DROME]	0.00	0.42	2	1	1	1	1.827E5	7210	808.5	5.10
Q9VK52	ACXB OS=Drosophila melanogaster GN=ACXB PE=4 SV=3 - [Q9VK52_DROME]	0.00	3.05	1	1	1	1		1114	127.6	7.14
A1ZAN7	RhoGEF2, isoform E OS=Drosophila melanogaster GN=RhoGEF2 PE=4 SV=1 - [A1ZAN7_DROME]	0.00	0.32	2	1	1	1	9.997E6	2205	242.1	6.40
Q9VXP5	EFHC1 homologue 1 OS=Drosophila melanogaster GN=Efhc1.1 PE=4 SV=2 - [Q9VXP5_DROME]	0.00	4.67	1	1	1	2		793	90.8	7.05
Q9W2B5	CG34369 OS=Drosophila melanogaster GN=ppk9 PE=4 SV=2 - [Q9W2B5_DROME]	0.00	4.51	1	1	1	2		599	68.1	7.56
Q9W347	C12.2, isoform A OS=Drosophila melanogaster GN=c12.2 PE=4 SV=3 - [Q9W347_DROME]	0.00	1.80	2	1	1	1		1386	155.6	7.09
Q9I7S5	CG2209-PA OS=Drosophila melanogaster GN=CG2209 PE=4 SV=2 - [Q9I7S5_DROME]	0.00	4.58	1	1	1	1	1.142E8	153	17.9	9.70
A1Z7C5	CG14760 OS=Drosophila melanogaster GN=CG14760 PE=3 SV=1 - [A1Z7C5_DROME]	0.00	5.10	1	1	1	1		529	57.8	6.61
A1Z8L3	CG7759, isoform A OS=Drosophila melanogaster GN=CG7759 PE=4 SV=1 - [A1Z8L3_DROME]	0.00	1.97	1	1	1	1	3.578E7	660	75.4	7.77
A1Z9C4	CG4744 OS=Drosophila melanogaster GN=CG4744 PE=4 SV=1 - [A1Z9C4_DROME]	0.00	0.73	1	1	1	1	4.408E7	1503	171.3	6.74
M9PDT1	CG2861, isoform E OS=Drosophila melanogaster GN=CG2861 PE=4 SV=1 - [M9PDT1_DROME]	0.00	1.66	3	2	2	2	2.654E7	1873	208.5	9.77
M9PGX7	Succinyl-CoA:3-ketoacid-coenzyme A transferase OS=Drosophila melanogaster GN=SCOT PE=3 SV=1 - [M9PGX7_DROME]	0.00	3.56	3	1	1	1		450	47.9	7.88

Accession	Description	Score	Coverage	# Proteins	# Unique Peptides	# Peptides	# PSMs	Area	# AAs	MW [kDa]	calc. pI
Q9VNN0	Osiris 10, isoform A OS=Drosophila melanogaster GN=Osi10 PE=4 SV=1 - [Q9VNN0_DROME]	0.00	1.39	2	1	1	1		576	63.1	7.24
Q8IM95	CG11155, isoform B OS=Drosophila melanogaster GN=CG11155 PE=4 SV=2 - [Q8IM95_DROME]	0.00	3.74	2	1	1	1		855	98.4	6.07
A8JNR2	CG32082, isoform D OS=Drosophila melanogaster GN=CG32082 PE=4 SV=2 - [A8JNR2_DROME]	0.00	3.50	4	1	1	1	3.485E7	629	69.6	9.17
Q9VZ30	CG11122 OS=Drosophila melanogaster GN=CG11122 PE=4 SV=3 - [Q9VZ30_DROME]	0.00	0.23	1	1	1	1	6.471E7	3539	384.8	9.29
A1Z6I7	Bub1-related kinase OS=Drosophila melanogaster GN=BubR1 PE=4 SV=1 - [A1Z6I7_DROME]	0.00	1.16	1	1	1	1		1460	165.0	6.33
A1Z7J1	Sticks and stones, isoform A OS=Drosophila melanogaster GN=sns PE=4 SV=1 - [A1Z7J1_DROME]	0.00	0.47	2	1	1	1	1.462E7	1479	161.8	7.75
M9PE86	CG43347, isoform D OS=Drosophila melanogaster GN=CG15295 PE=4 SV=1 - [M9PE86_DROME]	0.00	0.34	3	1	1	1	1.664E7	2652	281.8	6.30
M9PF85	Required for cell differentiation 1 ortholog, isoform C OS=Drosophila melanogaster GN=Rcd-1 PE=4 SV=1 - [M9PF85_DROME]	0.00	3.99	2	1	1	1		301	33.8	7.33
Q8INT3	CG31544 OS=Drosophila melanogaster GN=CG31544 PE=4 SV=2 - [Q8INT3_DROME]	0.00	9.51	1	1	1	1		263	29.6	9.14
X2JG22	CG7028, isoform D OS=Drosophila melanogaster GN=CG7028 PE=4 SV=1 - [X2JG22_DROME]	0.00	2.76	2	1	1	1		906	103.9	9.48
X2JDA8	Suppressor of sable, isoform B OS=Drosophila melanogaster GN=su(s) PE=4 SV=1 - [X2JDA8_DROME]	0.00	1.82	2	1	1	2		1321	143.4	5.58
A1Z8Q2	CG13185, isoform B OS=Drosophila melanogaster GN=CG13185 PE=4 SV=1 - [A1Z8Q2_DROME]	0.00	0.70	2	2	2	2	7.381E7	5303	604.8	5.25
X2JAZ3	Downstream of raf1, isoform B OS=Drosophila melanogaster GN=Dsor1 PE=4 SV=1 - [X2JAZ3_DROME]	0.00	4.07	2	1	1	1		393	43.5	6.21
Q9VDR5	CG4783 OS=Drosophila melanogaster GN=CG4783 PE=4 SV=1 - [Q9VDR5_DROME]	0.00	23.26	1	1	1	1		86	9.9	5.19
A1Z9B2	CG4714 OS=Drosophila melanogaster GN=CG4714 PE=4 SV=1 - [A1Z9B2_DROME]	0.00	2.87	1	1	1	1		732	83.6	5.06
Q2MGL8	CG33966 OS=Drosophila melanogaster GN=CG1211 PE=4 SV=1 - [Q2MGL8_DROME]	0.00	2.90	1	1	1	2	1.160E7	310	35.8	6.43
Q9VNF5	3-oxoacyl-[acyl-carrier-protein] synthase OS=Drosophila melanogaster GN=CG12170 PE=3 SV=1 - [Q9VNF5_DROME]	0.00	2.74	1	1	1	1		438	46.3	7.88
Q9VJP6	CG15260 OS=Drosophila melanogaster GN=CG15260 PE=4 SV=3 - [Q9VJP6_DROME]	0.00	9.13	1	1	1	1		230	26.4	8.44
M9NDD0	Rim2 ortholog, isoform D OS=Drosophila melanogaster GN=Rim2 PE=3 SV=1 - [M9NDD0_DROME]	0.00	6.62	3	1	1	1		272	30.7	9.44
M9PC19	CG42268, isoform M OS=Drosophila melanogaster GN=CG42268 PE=4 SV=1 - [M9PC19_DROME]	0.00	2.08	6	1	1	1		1200	129.2	5.67
X2JHS3	CG13366, isoform D OS=Drosophila melanogaster GN=CG13366 PE=4 SV=1 - [X2JHS3_DROME]	0.00	4.53	3	1	1	3	1.796E6	949	105.3	5.15
A8DYJ0	Endophilin B, isoform D OS=Drosophila melanogaster GN=EndoB PE=4 SV=1 - [A8DYJ0_DROME]	0.00	5.74	4	1	1	1		366	41.3	5.38
Q9VG47	Lipase OS=Drosophila melanogaster GN=CG11608 PE=3 SV=3 - [Q9VG47_DROME]	0.00	6.44	1	1	1	3		435	49.5	8.56
M9PID3	CG3680, isoform D OS=Drosophila melanogaster GN=CG3680 PE=4 SV=1 - [M9PID3_DROME]	0.00	2.06	4	1	1	2	1.103E7	923	99.6	6.89
Q9VHB0	CG8273 OS=Drosophila melanogaster GN=CG8273 PE=4 SV=1 - [Q9VHB0_DROME]	0.00	2.40	1	1	1	1		874	97.7	9.69
Q9W2S8	CG9806-PA OS=Drosophila melanogaster GN=CG9806 PE=4 SV=1 - [Q9W2S8_DROME]	0.00	1.54	1	1	1	1	8.019E7	911	103.3	7.43
Q9VXU8	Grip128 OS=Drosophila melanogaster GN=Grip128 PE=4 SV=2 - [Q9VXU8_DROME]	0.00	1.28	1	1	1	1		1092	127.3	6.49
Q9VQM0	Toucan, isoform A OS=Drosophila melanogaster GN=toc PE=4 SV=2 - [Q9VQM0_DROME]	0.00	4.49	10	3	3	3	7.913E6	2162	234.0	8.76
Q9VL48	CG13131 OS=Drosophila melanogaster GN=CG13131 PE=4 SV=2 - [Q9VL48_DROME]	0.00	0.53	1	1	1	3	3.847E8	1317	148.5	8.51
Q9VX86	CG8945 OS=Drosophila melanogaster GN=CG8945 PE=4 SV=1 - [Q9VX86_DROME]	0.00	0.70	1	1	1	1	3.349E7	1430	161.0	8.95
Q9VVG9	CG32176 OS=Drosophila melanogaster GN=CG32176 PE=4 SV=2 - [Q9VVG9_DROME]	0.00	3.10	1	1	1	1	5.572E6	581	65.1	5.26
Q9VB44	CG3339, isoform D OS=Drosophila melanogaster GN=CG3339 PE=4 SV=3 - [Q9VB44_DROME]	0.00	0.53	2	2	2	2	8.689E6	4689	536.6	6.51
M9PC84	Muscle-specific protein 300 kDa, isoform L OS=Drosophila melanogaster GN=Msp300 PE=4 SV=1 - [M9PC84_DROME]	0.00	0.24	9	1	2	4	1.344E8	###	1400.3	5.22
A1ZA45	CG30089 OS=Drosophila melanogaster GN=CG30089 PE=4 SV=1 - [A1ZA45_DROME]	0.00	1.98	1	1	1	1		1263	132.3	7.09
A1ZAT2	CG10764 OS=Drosophila melanogaster GN=CG10764 PE=3 SV=1 - [A1ZAT2_DROME]	0.00	6.50	1	1	1	1	2.035E8	523	57.7	7.59
X2JE64	CG4198, isoform B OS=Drosophila melanogaster GN=CG4198 PE=4 SV=1 - [X2JE64_DROME]	0.00	6.93	2	1	1	1		476	53.3	7.30
Q9VM02	Rap GTPase activating protein 1, isoform E OS=Drosophila melanogaster GN=RapGAP1 PE=4 SV=1 - [Q9VM02_DROME]	0.00	2.35	4	1	1	1	3.560E6	850	92.8	7.12
A8JR82	CG31158, isoform C OS=Drosophila melanogaster GN=Efa6 PE=4 SV=1 - [A8JR82_DROME]	0.00	2.09	3	1	1	1	7.822E5	1387	155.1	6.34
E1JJ04	Dynein heavy chain at 89D, isoform C OS=Drosophila melanogaster GN=Dhc98D PE=4 SV=1 - [E1JJ04_DROME]	0.00	0.75	2	2	2	2	5.364E7	5073	580.1	5.90
M9NE79	CG43055 OS=Drosophila melanogaster GN=CG43055 PE=4 SV=1 - [M9NE79_DROME]	0.00	8.89	1	1	1	1	9.719E7	180	20.7	5.07
Q5LJY8	CG40467 (Fragment) OS=Drosophila melanogaster GN=CG40467 PE=4 SV=3 - [Q5LJY8_DROME]	0.00	10.04	1	1	1	1		279	30.8	5.83
Q9VS86	CG16998 OS=Drosophila melanogaster GN=CG16998 PE=3 SV=2 - [Q9VS86_DROME]	0.00	3.88	1	1	1	1		258	28.2	8.21
A8DYT6	CG15387 OS=Drosophila melanogaster GN=CG15387 PE=4 SV=1 - [A8DYT6_DROME]	0.00	18.97	1	1	1	1		116	13.3	7.34
M9NDR2	Bicaudal D, isoform B OS=Drosophila melanogaster GN=BicD PE=4 SV=1 - [M9NDR2_DROME]	0.00	0.93	3	1	1	1		750	85.5	5.05
E1JIT1	CG31169, isoform C OS=Drosophila melanogaster GN=CG31169 PE=3 SV=1 - [E1JIT1_DROME]	0.00	1.70	3	1	1	1	9.221E6	1291	144.7	5.16
Q9VGC0	CG17404 OS=Drosophila melanogaster GN=CG17404 PE=3 SV=3 - [Q9VGC0_DROME]	0.00	11.27	1	1	1	1	4.654E5	275	29.5	8.70
X2JA59	Spalt major, isoform B OS=Drosophila melanogaster GN=salm PE=4 SV=1 - [X2JA59_DROME]	0.00	0.52	2	1	1	1	7.965E7	1355	148.9	6.51
Q8ING0	CG31326 OS=Drosophila melanogaster GN=CG31326 PE=3 SV=1 - [Q8ING0_DROME]	0.00	6.54	1	1	1	1		520	57.2	6.92
Q9VU38	Adenosine kinase, isoform B OS=Drosophila melanogaster GN=AdenoK PE=4 SV=1 - [Q9VU38_DROME]	0.00	2.03	2	1	1	1		345	37.8	5.38
Q9VU08	CG32113 OS=Drosophila melanogaster GN=CG32113 PE=4 SV=3 - [Q9VU08_DROME]	0.00	0.54	1	2	2	2	3.458E8	3919	444.8	6.83

Accession	Description	Score	Coverage	# Proteins	# Unique Peptides	# Peptides	# PSMs	Area	# AAs	MW [kDa]	calc. pI
M9PEB1	CG10289, isoform B OS=Drosophila melanogaster GN=CG10289 PE=4 SV=1 - [M9PEB1_DROME]	0.00	4.45	3	2	2	2	6.768E6	967	108.4	4.50
E1JIM1	CG4699, isoform J OS=Drosophila melanogaster GN=nsl1 PE=4 SV=1 - [E1JIM1_DROME]	0.00	2.78	4	2	2	2	3.535E7	936	99.0	7.81
A1Z8Z5	CG33626 OS=Drosophila melanogaster GN=CG33626 PE=4 SV=2 - [A1Z8Z5_DROME]	0.00	14.37	1	1	1	1		167	19.5	8.95
A8DQW8	CG42600 OS=Drosophila melanogaster GN=clos PE=4 SV=2 - [A8DQW8_DROME]	0.00	0.84	1	1	1	1	1.673E6	1785	201.0	6.07
M9NDA6	Lasp, isoform C OS=Drosophila melanogaster GN=Lasp PE=4 SV=1 - [M9NDA6_DROME]	0.00	3.77	2	1	1	1		636	71.8	8.41
A1Z9R6	Additional sex combs, isoform B OS=Drosophila melanogaster GN=Asx PE=4 SV=1 - [A1Z9R6_DROME]	0.00	1.28	2	1	1	1	3.785E6	1560	168.6	6.42
Q8IMH9	CG31019 OS=Drosophila melanogaster GN=CG31019 PE=4 SV=1 - [Q8IMH9_DROME]	0.00	2.58	1	1	1	1		659	74.2	9.17
Q9VQ68	CG42296 OS=Drosophila melanogaster GN=lectin-22C PE=4 SV=2 - [Q9VQ68_DROME]	0.00	1.41	1	1	1	1		994	109.4	6.37
Q9VW57	Grasp65 OS=Drosophila melanogaster GN=Grasp65 PE=4 SV=1 - [Q9VW57_DROME]	0.00	6.30	1	1	1	1		460	47.7	4.74
M9PDJ0	Klarsicht, isoform G OS=Drosophila melanogaster GN=klar PE=4 SV=1 - [M9PDJ0_DROME]	0.00	0.85	7	1	1	1	4.634E7	1180	128.8	6.44
Q8IRL1	CG32686-PA, isoform A OS=Drosophila melanogaster GN=CG32686 PE=4 SV=1 - [Q8IRL1_DROME]	0.00	3.20	2	1	1	1		719	79.6	9.89
Q9VNB7	CG14669 OS=Drosophila melanogaster GN=CG14669 PE=4 SV=5 - [Q9VNB7_DROME]	0.00	1.96	1	1	1	1	8.469E6	306	33.1	8.87
M9PB77	Sema-1a, isoform I OS=Drosophila melanogaster GN=Sema-1a PE=4 SV=1 - [M9PB77_DROME]	0.00	2.57	5	1	1	1	3.800E7	817	92.1	6.71
A1Z9C2	CG17050 OS=Drosophila melanogaster GN=CG17050 PE=4 SV=1 - [A1Z9C2_DROME]	0.00	3.36	1	1	1	2		506	58.9	9.25
Q8IRS0	Protein tyrosine phosphatase 4E, isoform B OS=Drosophila melanogaster GN=Ptp4E PE=4 SV=1 - [Q8IRS0_DROME]	0.00	1.43	4	1	1	1	3.967E4	1607	182.2	6.47
Q7PLB8	CG17374 OS=Drosophila melanogaster GN=CG17374 PE=4 SV=3 - [Q7PLB8_DROME]	0.00	1.38	1	1	1	1	1.124E6	2394	266.7	6.65
Q2PDV8	CG4822, isoform H OS=Drosophila melanogaster GN=CG4822 PE=4 SV=1 - [Q2PDV8_DROME]	0.00	19.09	4	1	1	1		110	11.8	5.31
Q9VWJ2	CG8028, isoform D OS=Drosophila melanogaster GN=CG8028 PE=4 SV=3 - [Q9VWJ2_DROME]	0.00	4.94	2	1	1	1	1.556E6	486	54.2	9.41
A1ZBH2	CG15111, isoform A OS=Drosophila melanogaster GN=CG15111 PE=4 SV=1 - [A1ZBH2_DROME]	0.00	9.41	2	1	1	1		393	45.5	9.20
A8DY94	CG34363, isoform E OS=Drosophila melanogaster GN=CG12897 PE=4 SV=2 - [A8DY94_DROME]	0.00	1.01	2	1	1	1	2.597E7	1774	194.5	7.52
E1JIJ7	CG7518, isoform C OS=Drosophila melanogaster GN=CG7518 PE=4 SV=1 - [E1JIJ7_DROME]	0.00	0.30	3	1	1	1		2301	251.9	8.54
M9PIF1	CG7324, isoform B OS=Drosophila melanogaster GN=CG7324 PE=4 SV=1 - [M9PIF1_DROME]	0.00	1.83	2	1	1	1		1256	143.2	5.97
M9PFR5	Suppressor of variegation 3-3, isoform C OS=Drosophila melanogaster GN=Su(var)3-3 PE=4 SV=1 - [M9PFR5_DROME]	0.00	3.56	2	1	1	1		870	95.9	7.31
Q9VCB7	CG6204 OS=Drosophila melanogaster GN=CG6204 PE=4 SV=1 - [Q9VCB7_DROME]	0.00	3.10	1	1	1	1		903	102.7	7.01
Q2PDQ0	CG34027 OS=Drosophila melanogaster GN=CG34027 PE=4 SV=2 - [Q2PDQ0_DROME]	0.00	0.39	1	1	1	1	4.108E7	1519	173.9	7.17
A8DYE4	CG34365, isoform F OS=Drosophila melanogaster GN=CG34365 PE=4 SV=1 - [A8DYE4_DROME]	0.00	2.14	1	1	1	1	2.788E8	1030	113.1	9.39
E1JIL5	Mitochondrial ribosomal protein S10, isoform D OS=Drosophila melanogaster GN=mRpS10 PE=4 SV=1 - [E1JIL5_DROME]	0.00	4.00	4	1	1	1	1.178E8	150	17.2	8.97
V9H0I1	Methuselah-like 13 OS=Drosophila melanogaster GN=mthl13 PE=4 SV=1 - [V9H0I1_DROME]	0.00	9.44	1	1	1	2		413	49.0	5.57
M9NFP9	Formin homology 2 domain containing ortholog, isoform G OS=Drosophila melanogaster GN=Fhos PE=4 SV=1 - [M9NFP9_DROME]	0.00	0.87	7	1	1	1		1152	129.7	7.20
Q9VML2	Blue cheese OS=Drosophila melanogaster GN=bchs PE=4 SV=4 - [Q9VML2_DROME]	0.00	0.20	1	1	1	1	9.575E6	3489	389.6	6.96
X2JEX7	Ubiquitin carboxyl-terminal hydrolase OS=Drosophila melanogaster GN=Usp7 PE=3 SV=1 - [X2JEX7_DROME]	0.00	1.07	2	1	1	1		1125	130.0	5.99
Q8IR47	Hog OS=Drosophila melanogaster GN=hog PE=4 SV=1 - [Q8IR47_DROME]	0.00	27.52	1	1	1	1	7.798E6	149	16.8	4.44
A1Z7Y9	CG33800 OS=Drosophila melanogaster GN=CheA46a PE=4 SV=1 - [A1Z7Y9_DROME]	0.00	15.82	1	1	1	1	1.530E6	177	20.1	7.40
A1Z7T9	CG13739 OS=Drosophila melanogaster GN=CG13739 PE=4 SV=1 - [A1Z7T9_DROME]	0.00	3.77	1	1	1	2		875	94.6	9.69
M9PHE1	CG2750, isoform C OS=Drosophila melanogaster GN=CG2750 PE=4 SV=1 - [M9PHE1_DROME]	0.00	0.58	2	1	1	1		1881	209.2	6.49
M9PBG8	Rho guanine nucleotide exchange factor 3, isoform M OS=Drosophila melanogaster GN=RhoGEF3 PE=4 SV=1 - [M9PBG8_DROME]	0.00	1.25	2	2	2	2	4.724E8	3518	383.6	9.09
Q7KVB1	ABCB7, isoform B OS=Drosophila melanogaster GN=ABCB7 PE=3 SV=1 - [Q7KVB1_DROME]	0.00	1.13	2	1	1	1	1.132E7	709	76.7	9.26
M9NGE4	Mushroom body defect, isoform I OS=Drosophila melanogaster GN=mud PE=4 SV=1 - [M9NGE4_DROME]	0.00	1.61	4	1	1	1		2113	242.7	5.40
Q7KU95	Ankyrin 2, isoform M OS=Drosophila melanogaster GN=Ank2 PE=4 SV=1 - [Q7KU95_DROME]	0.00	2.08	18	2	2	2	1.043E8	2404	264.6	6.19
E1JH06	CG30372, isoform C OS=Drosophila melanogaster GN=Asap PE=4 SV=1 - [E1JH06_DROME]	0.00	1.06	2	1	1	1		1127	124.2	6.89
Q9VIC6	CG34127 OS=Drosophila melanogaster GN=CG32465 PE=4 SV=3 - [Q9VIC6_DROME]	0.00	1.12	1	1	1	1		1159	125.1	8.21
M9PBW8	CG33125, isoform C OS=Drosophila melanogaster GN=CG33125 PE=4 SV=1 - [M9PBW8_DROME]	0.00	4.35	2	1	1	1		736	86.5	9.58
Q9W4J0	CG12184, isoform A OS=Drosophila melanogaster GN=CG12184 PE=4 SV=3 - [Q9W4J0_DROME]	0.00	1.02	2	1	1	1		981	110.2	9.69
Q9VH49	CG12419 OS=Drosophila melanogaster GN=CG12419 PE=4 SV=1 - [Q9VH49_DROME]	0.00	11.45	1	1	1	1	1.182E8	131	14.2	9.74
M9NDG8	CG11236, isoform B OS=Drosophila melanogaster GN=CG11236 PE=4 SV=1 - [M9NDG8_DROME]	0.00	2.96	2	1	1	1	5.112E6	338	37.3	7.36
Q9VUY2	CG13074 OS=Drosophila melanogaster GN=CG13074 PE=4 SV=1 - [Q9VUY2_DROME]	0.00	8.87	1	1	1	1		451	50.4	5.58
Q9VZG1	Cuticular protein 64Ab OS=Drosophila melanogaster GN=Cpr64Ab PE=4 SV=2 - [Q9VZG1_DROME]	0.00	10.83	1	1	1	1		120	12.5	4.83
Q9VWB9	CG15618, isoform B OS=Drosophila melanogaster GN=CG15618 PE=4 SV=3 - [Q9VWB9_DROME]	0.00	0.40	1	1	1	1	2.069E9	1746	197.2	6.84
A8JR35	Dscam3, isoform C OS=Drosophila melanogaster GN=Dscam3 PE=4 SV=1 - [A8JR35_DROME]	0.00	2.64	3	2	2	2		2007	219.6	7.88
M9PEB2	Chromodomain-helicase-DNA-binding protein 1, isoform C OS=Drosophila melanogaster GN=Chd1 PE=4 SV=1 - [M9PEB2_DROME]	0.00	0.48	3	1	1	1	1.491E7	1881	211.4	6.34

Accession	Description	Score	Coverage	# Proteins	# Unique Peptides	# Peptides	# PSMs	Area	# AAs	MW [kDa]	calc. pI
M9PCJ9	Sarcoglycan alpha, isoform B OS=Drosophila melanogaster GN=Scgalpha PE=4 SV=1 - [M9PCJ9_DROME]	0.00	4.19	3	1	1	1	9.174E6	406	46.7	8.38
Q9VV18	CG13047 OS=Drosophila melanogaster GN=CG13047 PE=4 SV=2 - [Q9VV18_DROME]	0.00	8.24	1	1	1	2	2.189E7	170	16.6	8.54
Q9VHZ8	CG18268 OS=Drosophila melanogaster GN=CG18268 PE=4 SV=1 - [Q9VHZ8_DROME]	0.00	4.75	1	1	1	1	3.790E7	358	40.1	8.51
Q9W135	CG42360 OS=Drosophila melanogaster GN=CG42360 PE=4 SV=3 - [Q9W135_DROME]	0.00	2.02	1	1	1	1		496	56.1	8.88
Q9VAU5	CG10011 OS=Drosophila melanogaster GN=CG10011 PE=4 SV=1 - [Q9VAU5_DROME]	0.00	0.90	1	1	1	1	5.451E5	2119	232.9	6.28
A1Z7P0	Phosphomannomutase 45A, isoform B OS=Drosophila melanogaster GN=Pmm45A PE=3 SV=1 - [A1Z7P0_DROME]	0.00	1.88	2	1	1	1	2.835E6	584	65.4	5.35
B7Z0U7	Like-AP180, isoform C OS=Drosophila melanogaster GN=lap PE=4 SV=2 - [B7Z0U7_DROME]	0.00	4.35	3	2	2	2	1.644E7	758	80.8	7.18
Q8IMQ3	CG31077 OS=Drosophila melanogaster GN=CG31077 PE=4 SV=2 - [Q8IMQ3_DROME]	0.00	2.43	1	1	1	1		988	110.3	4.53
M9PG46	Ubiquitin carboxyl-terminal hydrolase OS=Drosophila melanogaster GN=CG8334 PE=3 SV=1 - [M9PG46_DROME]	0.00	1.42	4	1	1	1		1693	189.2	6.40
M9NE59	CG43078, isoform G OS=Drosophila melanogaster GN=CG32355 PE=4 SV=1 - [M9NE59_DROME]	0.00	0.77	1	1	1	1		2455	274.4	5.38
A1ZA27	CG30466 OS=Drosophila melanogaster GN=CG30466 PE=4 SV=2 - [A1ZA27_DROME]	0.00	1.73	1	1	1	1	1.295E8	404	47.5	8.65
M9PFW7	M6, isoform E OS=Drosophila melanogaster GN=M6 PE=4 SV=1 - [M9PFW7_DROME]	0.00	8.08	2	1	1	1		297	34.0	7.87
M9NEA5	DNA-binding protein SATB OS=Drosophila melanogaster GN=ct PE=3 SV=1 - [M9NEA5_DROME]	0.00	0.69	4	1	1	1	5.799E7	2165	233.2	5.58
Q8MLW8	Cht12 OS=Drosophila melanogaster GN=Cht12 PE=3 SV=1 - [Q8MLW8_DROME]	0.00	5.11	1	1	1	1	4.253E6	470	54.4	6.76
Q9VPL6	Kismet, isoform B OS=Drosophila melanogaster GN=kis PE=4 SV=1 - [Q9VPL6_DROME]	0.00	1.91	6	2	2	2	1.070E7	2151	224.6	6.21
A8JUU1	Sec16 ortholog, isoform E OS=Drosophila melanogaster GN=Sec16 PE=4 SV=1 - [A8JUU1_DROME]	0.00	1.04	4	1	1	1		2021	220.0	5.76
Q0KI16	Molting defective, isoform B OS=Drosophila melanogaster GN=mld PE=4 SV=1 - [Q0KI16_DROME]	0.00	1.50	2	1	1	1	6.229E6	1728	194.0	6.19
Q9VNK7	CG1075 OS=Drosophila melanogaster GN=CR1075 PE=3 SV=1 - [Q9VNK7_DROME]	0.00	2.92	1	1	1	1		274	31.6	8.69
Q8MMF3	CG4329, isoform B OS=Drosophila melanogaster GN=CG4329 PE=4 SV=1 - [Q8MMF3_DROME]	0.00	1.95	2	1	1	1	1.877E7	667	78.2	5.53
Q9W198	CG3363 OS=Drosophila melanogaster GN=ocm PE=4 SV=1 - [Q9W198_DROME]	0.00	0.51	1	1	1	1	5.263E9	2175	241.8	9.09
M9NET6	Pigment-dispersing factor receptor, isoform B OS=Drosophila melanogaster GN=Pdfr PE=4 SV=1 - [M9NET6_DROME]	0.00	1.90	1	1	1	1		738	81.9	8.76
X2JF83	CG8184, isoform G OS=Drosophila melanogaster GN=CG8184 PE=4 SV=1 - [X2JF83_DROME]	0.00	1.02	7	2	2	2		5101	552.7	5.50
Q59DZ3	CG7147-PC, isoform C OS=Drosophila melanogaster GN=kuz PE=4 SV=1 - [Q59DZ3_DROME]	0.00	1.38	4	1	1	1		1090	121.1	8.21
M9NE82	Dumpy, isoform M OS=Drosophila melanogaster GN=dp PE=4 SV=1 - [M9NE82_DROME]	0.00	0.65	16	2	2	2	1.126E6	8579	915.3	5.53
M9NFK8	CG12017, isoform B OS=Drosophila melanogaster GN=CG12017 PE=4 SV=1 - [M9NFK8_DROME]	0.00	1.15	4	1	1	1		522	57.3	5.17
X2JIM3	CG15894, isoform D OS=Drosophila melanogaster GN=CG15894 PE=4 SV=1 - [X2JIM3_DROME]	0.00	1.27	4	1	1	1		1493	157.3	8.66
Q9VAS0	CG14527 OS=Drosophila melanogaster GN=CG14527 PE=4 SV=1 - [Q9VAS0_DROME]	0.00	4.29	1	1	1	1	2.439E6	700	81.1	6.35
Q9VCZ9	Neurexin 1, isoform A OS=Drosophila melanogaster GN=Nrx-1 PE=4 SV=1 - [Q9VCZ9_DROME]	0.00	0.38	2	1	1	1		1837	199.2	7.15
Q7KS15	Ubiquitin carboxyl-terminal hydrolase OS=Drosophila melanogaster GN=puf PE=3 SV=1 - [Q7KS15_DROME]	0.00	1.38	3	2	2	2		2832	318.5	5.90
E1JGN7	Bancal, isoform E OS=Drosophila melanogaster GN=bl PE=4 SV=1 - [E1JGN7_DROME]	0.00	6.90	5	2	2	3	6.197E7	493	49.8	8.35
Q7KVK3	Roundabout, isoform A OS=Drosophila melanogaster GN=robo PE=4 SV=1 - [Q7KVK3_DROME]	0.00	1.43	2	1	1	1		1395	151.7	7.24
Q9W1R3	CG3493 OS=Drosophila melanogaster GN=Golgin245 PE=4 SV=2 - [Q9W1R3_DROME]	0.00	1.14	1	1	1	1	4.317E7	1489	170.0	5.20
E1JJG4	Anaphase promoting complex subunit 4, isoform C OS=Drosophila melanogaster GN=APC4 PE=4 SV=1 - [E1JJG4_DROME]	0.00	3.10	2	1	1	1	6.353E6	773	87.1	4.97
M9PDX4	CG13933, isoform B OS=Drosophila melanogaster GN=CG13933 PE=4 SV=1 - [M9PDX4_DROME]	0.00	3.43	2	1	1	1	4.960E7	670	77.0	8.16
X2JCI0	Fasciclin 2, isoform H OS=Drosophila melanogaster GN=Fas2 PE=4 SV=1 - [X2JCI0_DROME]	0.00	1.92	5	1	1	1	3.354E8	832	93.0	6.14
Q9VFV0	Ionotropic receptor 87a OS=Drosophila melanogaster GN=Ir87a PE=4 SV=1 - [Q9VFV0_DROME]	0.00	3.23	1	1	1	1	6.913E5	806	92.6	4.64
Q9VAT1	CG1647 OS=Drosophila melanogaster GN=CG1647 PE=4 SV=1 - [Q9VAT1_DROME]	0.00	1.96	1	1	1	1		1222	136.0	4.89
Q8MLS0	CG13551, isoform C OS=Drosophila melanogaster GN=CG13551 PE=4 SV=1 - [Q8MLS0_DROME]	0.00	13.79	2	1	1	1		87	9.7	6.80
Q9W246	CG4554 OS=Drosophila melanogaster GN=CG4554 PE=4 SV=2 - [Q9W246_DROME]	0.00	0.55	1	1	1	1	1.497E6	2733	311.7	7.74
A1Z9P8	CG10102 OS=Drosophila melanogaster GN=CR10102 PE=4 SV=1 - [A1Z9P8_DROME]	0.00	13.19	1	1	1	1	3.920E6	182	21.7	7.06
Q9VUM6	Bestrophin 4 OS=Drosophila melanogaster GN=Best4 PE=4 SV=3 - [Q9VUM6_DROME]	0.00	2.85	1	1	1	1		526	61.4	6.62
A1Z9I6	CG13344 OS=Drosophila melanogaster GN=CG13344 PE=4 SV=1 - [A1Z9I6_DROME]	0.00	2.94	1	1	1	1		374	42.1	6.33
A8DYM1	CG11206, isoform D OS=Drosophila melanogaster GN=Liprin-gamma PE=4 SV=1 - [A8DYM1_DROME]	0.00	3.05	3	1	1	1		852	93.0	6.99
Q9VCN6	Cdc16 OS=Drosophila melanogaster GN=Cdc16 PE=4 SV=1 - [Q9VCN6_DROME]	0.00	1.95	1	1	1	1		718	81.7	5.52
M9PG56	CG42674, isoform G OS=Drosophila melanogaster GN=CG13811 PE=4 SV=1 - [M9PG56_DROME]	0.00	3.30	6	1	1	1	4.431E6	848	93.2	7.74
Q9VAS9	APC-like OS=Drosophila melanogaster GN=Apc PE=4 SV=1 - [Q9VAS9_DROME]	0.00	0.54	1	1	1	1	9.856E6	2417	261.1	5.64
Q7KUK9	CG17839, isoform B OS=Drosophila melanogaster GN=CG17839 PE=1 SV=2 - [Q7KUK9_DROME]	0.00	1.57	1	1	1	1		1656	178.4	6.35
Q9VDG0	Dynein heavy chain at 93AB OS=Drosophila melanogaster GN=Dhc93AB PE=4 SV=3 - [Q9VDG0_DROME]	0.00	0.18	1	1	1	1	1.306E8	4486	513.7	5.49
Q9VXS2	CG15599 OS=Drosophila melanogaster GN=CG15599 PE=4 SV=2 - [Q9VXS2_DROME]	0.00	3.97	1	1	1	1	1.005E7	1057	116.4	9.92
Q9VK08	CG44085, isoform P OS=Drosophila melanogaster GN=CG44085 PE=4 SV=2 - [Q9VK08_DROME]	0.00	1.20	2	1	1	1		2246	251.5	4.87

Accession	Description	Score	Coverage	# Proteins	# Unique Peptides	# Peptides	# PSMs	Area	# AAs	MW [kDa]	calc. pI
M9NDR1	CG43120 OS=Drosophila melanogaster GN=CG43120 PE=4 SV=1 - [M9NDR1_DROME]	0.00	9.44	1	1	1	1	4.533E6	286	33.4	5.26
Q9VR15	CG3355, isoform A OS=Drosophila melanogaster GN=CG3355 PE=3 SV=1 - [Q9VR15_DROME]	0.00	11.78	1	1	1	1	1.453E7	314	34.4	9.11
Q9VDL0	Srp14, isoform B OS=Drosophila melanogaster GN=Srp14 PE=4 SV=2 - [Q9VDL0_DROME]	0.00	14.68	1	1	1	1	3.079E7	109	12.4	10.17
E1JH95	CG4966, isoform C OS=Drosophila melanogaster GN=HPS4 PE=4 SV=1 - [E1JH95_DROME]	0.00	2.29	3	1	1	1		828	93.1	6.80
Q7KUH2	Mucin 68Ca OS=Drosophila melanogaster GN=Muc68Ca PE=4 SV=1 - [Q7KUH2_DROME]	0.00	1.31	1	2	2	2		3135	308.9	4.11
M9NDN5	CG3280 OS=Drosophila melanogaster GN=CG3280 PE=4 SV=1 - [M9NDN5_DROME]	0.00	0.93	1	1	1	1	9.673E7	1932	216.5	6.57
M9PIA6	Mi-2, isoform D OS=Drosophila melanogaster GN=Mi-2 PE=4 SV=1 - [M9PIA6_DROME]	0.00	1.12	3	1	1	1		1973	223.0	5.76
M9PDL0	CG3630, isoform B OS=Drosophila melanogaster GN=CG3630 PE=4 SV=1 - [M9PDL0_DROME]	0.00	6.63	2	1	1	1		347	36.9	5.01
Q9VBR8	CG11902 OS=Drosophila melanogaster GN=CG11902 PE=4 SV=2 - [Q9VBR8_DROME]	0.00	1.19	1	1	1	1		1426	165.8	7.66
M9NDF0	Poor gastrulation, isoform E OS=Drosophila melanogaster GN=pog PE=4 SV=1 - [M9NDF0_DROME]	0.00	3.24	4	1	1	1	3.884E6	894	98.9	7.99
Q9VV61	CG33158 OS=Drosophila melanogaster GN=CG33158 PE=4 SV=3 - [Q9VV61_DROME]	0.00	1.26	1	1	1	1		1033	115.4	5.90
M9PFE5	CG10732, isoform D OS=Drosophila melanogaster GN=CG10732 PE=4 SV=1 - [M9PFE5_DROME]	0.00	2.52	4	2	2	2		1624	180.3	5.68
Q9VWZ3	CG7092-PA OS=Drosophila melanogaster GN=Dhc16F PE=4 SV=1 - [Q9VWZ3_DROME]	0.00	0.59	1	1	1	1		4081	468.8	6.37
Q9VF50	CG18516 OS=Drosophila melanogaster GN=CG18516 PE=4 SV=1 - [Q9VF50_DROME]	0.00	1.35	1	1	1	1	1.035E7	1256	138.5	6.04
Q59DY2	CG33510 OS=Drosophila melanogaster GN=CG33510 PE=4 SV=2 - [Q59DY2_DROME]	0.00	1.64	1	1	1	1	6.233E7	426	49.8	5.69
Q9VHT6	CG9626 OS=Drosophila melanogaster GN=CG9626 PE=4 SV=2 - [Q9VHT6_DROME]	0.00	0.84	1	1	1	1		1667	184.1	8.84
A1Z6P0	CG9430 OS=Drosophila melanogaster GN=Zip42C.2 PE=4 SV=1 - [A1Z6P0_DROME]	0.00	10.82	1	1	1	1	2.141E6	305	33.9	7.97
Q9VZ77	CG17150, isoform D OS=Drosophila melanogaster GN=CG17150 PE=4 SV=3 - [Q9VZ77_DROME]	0.00	0.82	1	2	2	4	9.187E7	4385	504.3	6.24
X2JCN4	Kugelei, isoform E OS=Drosophila melanogaster GN=kug PE=4 SV=1 - [X2JCN4_DROME]	0.00	0.68	2	1	1	1	1.962E6	4689	522.4	5.07
A1ZAV2	CG18631 OS=Drosophila melanogaster GN=CG18432 PE=4 SV=1 - [A1ZAV2_DROME]	0.00	4.24	1	1	1	1	2.302E5	519	60.0	7.06
Q9VU57	CG17687 OS=Drosophila melanogaster GN=CG17687 PE=4 SV=1 - [Q9VU57_DROME]	0.00	0.97	1	1	1	2	1.716E8	1544	179.3	8.51
M9PE54	CG3597, isoform B OS=Drosophila melanogaster GN=CG3597 PE=4 SV=1 - [M9PE54_DROME]	0.00	6.53	1	1	1	1		337	38.0	6.54
Q7KTG2	Apolipoprotein lipid transfer particle OS=Drosophila melanogaster GN=Apoltp PE=4 SV=2 - [Q7KTG2_DROME]	0.00	0.39	1	1	1	1	1.431E7	4333	494.0	6.33
